# Targeted degradation via direct 26S proteasome recruitment

**DOI:** 10.1038/s41589-022-01218-w

**Published:** 2022-12-28

**Authors:** Charlene Bashore, Sumit Prakash, Matthew C. Johnson, Ryan J. Conrad, Ivy A. Kekessie, Suzie J. Scales, Noriko Ishisoko, Tracy Kleinheinz, Peter S. Liu, Nataliya Popovych, Aaron T. Wecksler, Lijuan Zhou, Christine Tam, Inna Zilberleyb, Rajini Srinivasan, Robert A. Blake, Aimin Song, Steven T. Staben, Yingnan Zhang, David Arnott, Wayne J. Fairbrother, Scott A. Foster, Ingrid E. Wertz, Claudio Ciferri, Erin C. Dueber

**Affiliations:** 1 Department of Early Discovery Biochemistry, Genentech Inc., South San Francisco, CA USA; 2 Department of Discovery Oncology, Genentech Inc., South San Francisco, CA USA; 3 Department of Structural Biology, Genentech Inc., South San Francisco, CA USA; 4 Department of Immunology, Genentech Inc., South San Francisco, CA USA; 5 Department of Biochemical and Cellular Pharmacology, Genentech Inc., South San Francisco, CA USA; 6 Department of Microchemistry, Proteomics, and Lipidomics, Genentech Inc., South San Francisco, CA USA; 7 Department of Protein Analytical Chemistry, Genentech Inc., South San Francisco, CA USA; 8 Department of Biomolecular Resources, Genentech Inc., South San Francisco, CA USA; 9 Department of Molecular Biology, Genentech Inc., South San Francisco, CA USA; 10 Department of Discovery Chemistry, Genentech Inc., South San Francisco, CA USA

**Keywords:** Chemical tools, Peptides, Target identification

## Abstract

Engineered destruction of target proteins by recruitment to the cell’s degradation machinery has emerged as a promising strategy in drug discovery. The majority of molecules that facilitate targeted degradation do so via a select number of ubiquitin ligases, restricting this therapeutic approach to tissue types that express the requisite ligase. Here, we describe a new strategy of targeted protein degradation through direct substrate recruitment to the 26S proteasome. The proteolytic complex is essential and abundantly expressed in all cells; however, proteasomal ligands remain scarce. We identify potent peptidic macrocycles that bind directly to the 26S proteasome subunit PSMD2, with a 2.5-Å-resolution cryo-electron microscopy complex structure revealing a binding site near the 26S pore. Conjugation of this macrocycle to a potent BRD4 ligand enabled generation of chimeric molecules that effectively degrade BRD4 in cells, thus demonstrating that degradation via direct proteasomal recruitment is a viable strategy for targeted protein degradation.

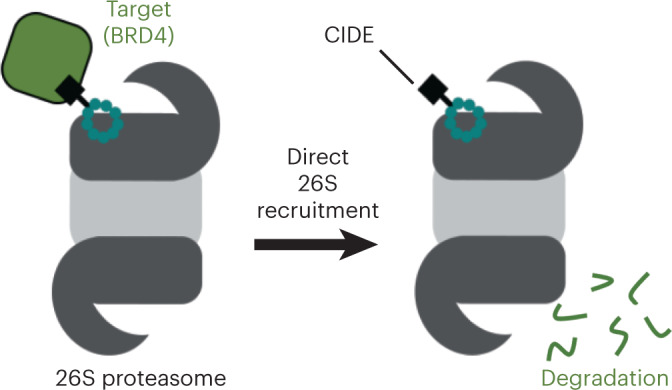

## Abstract (web summary)

Discovery of macrocyclic ligands to the 19S regulatory particle protein PSMD2 enables the synthesis of heterobifunctional molecules that demonstrate proof-of-concept, targeted degradation of BRD4 through direct engagement of the 26S proteasome.

## Main

Protein degradation is an essential function for all living cells^1^. Recently there has been a sizeable effort to engineer the degradation of target proteins for therapeutic benefit^2,3^. At the forefront of this newly emerging field are heterobifunctional molecules that bind to both a target protein and a ubiquitin ligase, thereby mediating the ubiquitination and destruction of a given target. Such targeted protein degradation has a number of putative advantages, including the simultaneous destruction of a target’s scaffolding and enzymatic activities and the ability to catalytically turn over the target protein within a cell. Given these potential therapeutic benefits, work in the field has increased rapidly, and heterobifunctional degrader molecules have recently entered the clinic^4^. One of the limitations of this approach is the requirement for non-native substrates to be efficiently ubiquitinated by a ubiquitin ligase. To date, only a select number of ligases have been identified that can be co-opted to robustly induce degradation of targeted proteins, in particular cullin ligase family members Von–Hippel Lindau and cereblon^5–9^; however, their efficacy depends on the presence of an available lysine on the target protein and expression and subcellular localization of the ligase in the relevant tissue^7,10–12^.

Targeting proteins directly to the 26S proteasome would provide an alternate strategy to induce efficient substrate degradation. Because of its indispensable function, the 26S proteasome is highly expressed in the cytoplasm and nuclei of all cells^13,14^. Moreover, a degrader molecule able to recruit targets directly to the proteasome would not require efficient ubiquitination, providing a universal degradation strategy applicable even to proteins lacking available lysines, and would not be subject to the counteracting effects of deubiquinating enzymes. Despite these advantages, this approach is currently hindered by the limited number of suitable ligands against 26S subunits to properly position a target protein on the complex for substrate processing and degradation.

In this study, we used mRNA display technology to sample a wide range of chemical space and discover highly potent macrocyclic ligands against the 26S subunit PSMD2, which plays a role in endogenous substrate delivery and processing and is consequently ideally located for substrate delivery. A 2.5-Å-resolution cryo-electron microscopy (cryo-EM) structure of a PSMD2–macrocycle complex revealed that the ligand bound to a site of PSMD2 that is accessible in the assembled 26S proteasome and distinct from interfaces used by other PSMD2 binding partners, such as USP14. Conjugation of the macrocycle to a potent BRD4 ligand produced proteasome-targeting, heterobifunctional BRD4 degraders, referred to here as chemical inducers of degradation (CIDEs). These molecules retain affinity for PSMD2 and BRD4 and mediate robust ternary complex formation. Finally, the cell-permeable nature of these chimeric tool compounds enabled study of CIDE-directed cellular BRD4 degradation in cells and confirmation of their dependence on macrocycle binding, proteasome function and BRD4 ligand binding. Thus, we show proof of concept for this unique strategy in targeted protein degradation, potentially expanding the number of target proteins amenable to engineered destruction as a therapeutic strategy.

## Results

### Discovery of ligands against the 26S proteasome subunit PSMD2

**Fig. 1:**
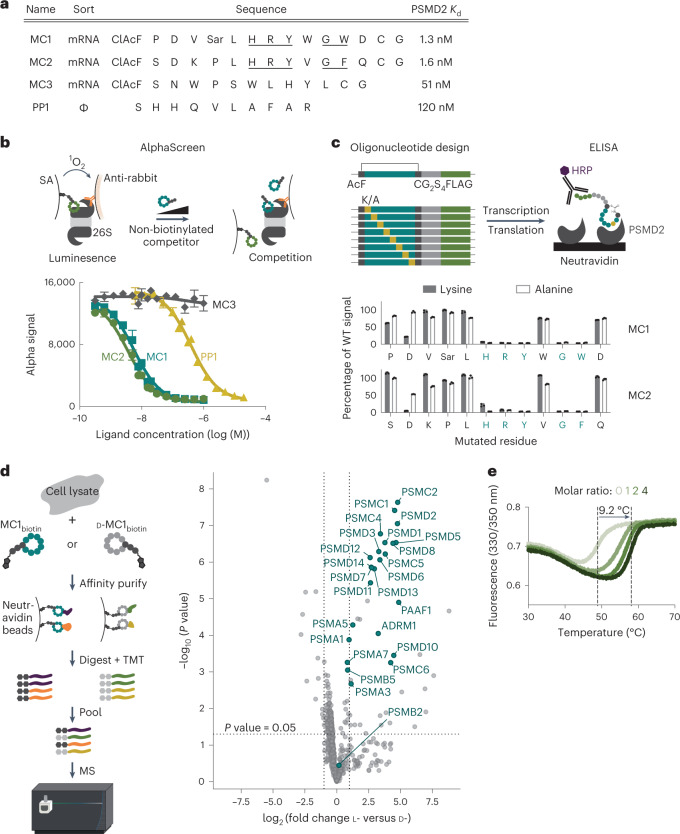
Discovery of potent ligands against proteasome subunit PSMD2. **a**, Phage and mRNA display approaches yield macrocycle ligands with high affinity to PSMD2. ClAc-F, *N*-chloroacetyl l-phenylalanine; Sar, sarcosine (*N*-methyl glycine); ϕ, phage display. **b**, Top macrocycle ligands bind the 26S proteasome with nanomolar affinity. An AlphaScreen assay using a biotinylated analog of macrocycle MC2 and an antibody to PSMD1 shows dose-dependent 26S proteasome binding for three of the four ligands; curves represent the mean ± s.d. from three independent experiments. SA, streptavidin. **c**, Alanine and lysine scanning of MC2 and MC1 using in vitro translation and ELISA reveals that an HRYxGW/F motif is critical for PSMD2 binding. Bars represent the mean ± s.d. of three independent experiments; WT, wild type. **d**, AP–MS and TMT–MS with MC1_biotin_ from HEK293 lysates shows enrichment of proteasome subunits for the active l-enantiomer over the inactive d-enantiomer control in a binary comparison volcano plot. Data represent three biological replicates per condition (l-CIDE, d-CIDE and DMSO control). The horizontal dotted line shows a *P* value of 0.05. The vertical dotted lines show log_2_ (fold change) of –1 or 1. For details of the data and statistical analyses, see the Methods. **e**, Macrocycle MC2 dramatically stabilizes PSMD2, increasing its *T*_m_ by 9.2 °C, as observed by differential scanning fluorimetry.

**Extended Data Fig. 1:**
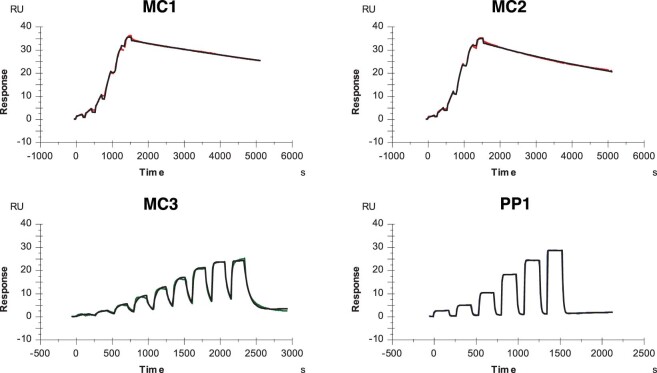
Representative SPR sensorgrams for top four hits binding to PSMD2. SPR sensorgrams of the indicated ligands binding to PSMD2 immobilized on the sensor. The colored lines are measured response units (RUs) with the kinetic fits from the Biacore S200 Evaluation Software shown overlayed in black. The derived K_D_ values and kinetic parameters are reported in Supplementary Table 2. Data were reproduced in at least 3 independent experiments.

Recombinant isolated PSMD2 protein was screened against naive 8- to 16-amino acid-long peptide phage libraries and 10- to 14-residue macrocyclic mRNA display libraries to identify high-affinity peptidic ligands within the nanomolar range of affinities (Fig. 1a and Supplementary Fig. 1). In particular, the latter mRNA display technology allows for reprogramming of the genetic code, resulting in the inclusion of non-standard amino acids^15–18^. This process substantially expands the chemical diversity of the library, including an N-terminal chloroacetyl amino acid that after translation cyclizes with the thiol in a C-terminal cysteine to form a thioether bond. These features resulted in the most potent hits, generating redox-stable macrocycles with low nanomolar affinity to PSMD2 (Supplementary Figs. 1 and 2, Supplementary Tables 1 and 2 and Extended Data Fig. 1).

To determine if the highest-affinity PSMD2-binding macrocycles (MC1, MC2 and MC3) and phage-derived peptide (PP1) bind to a surface of PSMD2 that is accessible in the assembled 26S proteasome, biotinylated versions of these ligands were synthesized and evaluated for their competency to bind the full degradation complex (Supplementary Table 1). The biotinylated analogs of the two most potent macrocycles (MC1_biotin_ and MC2_biotin_, respectively) and the phage-derived peptide (PP1_biotin_) successfully pulled down the 26S proteasome (Supplementary Fig. 3). Despite demonstrating a tenfold higher affinity for isolated PSMD2 than the linear PP1, a biotinylated version of MC3 (MC3_biotin_) failed to associate with the 26S proteasome, suggesting that the binding epitope of this ligand is likely occluded in the complex. Further quantitation of these interactions was achieved through the development of an AlphaScreen assay that used MC2_biotin_ as a tracer and an antibody to an adjacent 26S proteasome subunit, PSMD1, as a handle for a luminescent, proximity-based probe (Fig. 1b, top). Macrocycles MC1 and MC2 bind to 26S in a dose-dependent manner, yielding half-maximum inhibitory concentration (IC_50_) values close to the 5 nM detection limit of the assay (Fig. 1b, bottom). Competition of MC1 and MC2 with the biotinylated tracer agrees with the apparent primary sequence conservation of these macrocycles, with lysine and alanine scanning of both macrocyclic ligands by in vitro translation and enzyme-linked immunosorbent assay (ELISA) revealing a strong consensus for a central HRYxGW/F motif (Fig. 1c). Moreover, cyclization is required for ligand binding, as mutation of the cysteine to a serine, which cannot react to form a thioether bond, abrogated ligand binding to PSMD2 (Supplementary Fig. 4).

**Extended Data Fig. 2:**
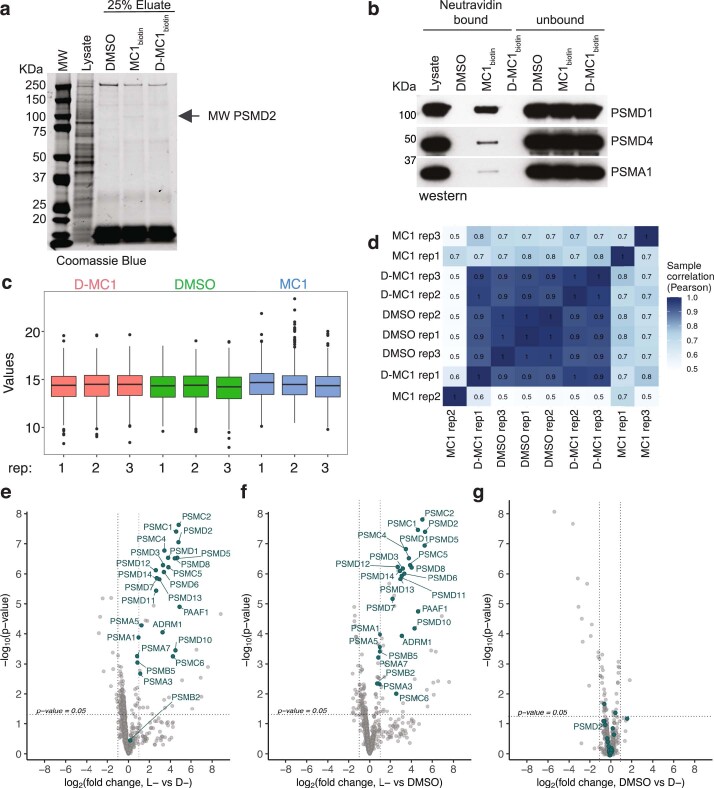
Biotinylated macrocycles pull down proteasome subunits in cell lysates. **a**, Affinity purification of L- and D- versions of MC1_biotin_ incubated with HEK 293 lysates run on SDS-PAGE and stained for total protein elution. **b**, Western blot showing MC1_biotin_, but not the inactive D enantiomer or DMSO, co-purifies with 19S subunits PSMD1, PSMD4, as well as 20S subunit PSMA1. **c**, The distribution of normalized log2 protein intensities across three biological replicates from each condition shown in (**a**) and (**b**) show similar abundance across all samples. Box plots represent data distribution (minimum, first quantile, median, third quantile, and maximum) with all proteins. The lower whisker is the smallest normalized log2 intensities or equal to lower bound of box-1.5*IQR (Interquantile range=75% quantile-25% quantile). The upper whisker is the largest normalized log2 intensities or equal to upper bound of box+1.5*IQR. Outliers are displayed as dots in each sample. **d**, Sample correlation heatmap showing correlation between each biological replicate, represented by Pearson coefficient. **e-g**, Volcano plots showing enrichment for L- vs D- affinity purification (panel **e**, included from Fig. 1d for comparison), L- vs -DMSO (**f**), and DMSO vs D- affinity purification (**g**), showing clear enrichment of proteasome subunits for the L-enantiomer of MC1. Data in **e-g** represent three biological replicates per condition (L-CIDE, D-CIDE and DMSO control). Horizontal dotted line shows p-value=0.05. Vertical dotted lines show log2(fold change)= -1 or 1. For details of data and statistical analysis see methods. Source data

We next assessed the specificity of the macrocycle MC1 by using its biotinylated analog (MC1_biotin_) to perform affinity purification from cell lysates and quantify any co-purified proteins by tandem mass tag–mass spectrometry (TMT–MS). As expected, macrocycle MC1_biotin_ strongly enriched for 26S subunits compared to the enantiomeric control or DMSO (Fig. 1d and Extended Data Fig. 2). Surprisingly, in a label-free affinity purification–MS (AP–MS) experiment, MC1_biotin_ showed a potential interaction with the CUL3 ligase and substrate adapter Kelch-like 15 (KLHL15; Supplementary Fig. 5); however, this interaction was not identified in the quantitative TMT experiment that included more stringent purification (Fig. 1d and Extended Data Fig. 2), indicating a potentially weak interaction. Previous studies reported that KLHL15 recognizes substrates via an ‘FRY’ motif, which is similar to the critical ‘HRY’ motif in our potent macrocyclic ligands MC1 and MC2 (refs. 19,20).

**Extended Data Fig. 3:**
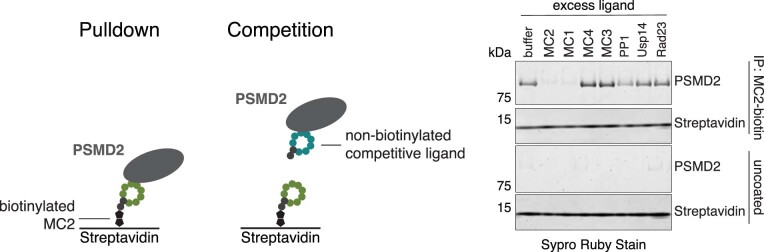
Macrocycles MC1 and MC2 do not compete with USP14 or Rad23b-Ubl for binding to PSMD2. Streptavidin beads coated with MC2_biotin_ pull down PSMD2 in the presence of excess MC3, MC4, USP14, or RAD23b_UBL_, but not in the presence of competing MC1 or MC2. PSMD2 capture is also attenuated in the presence of the phage-derived peptide ligand, PP1. Data were reproduced in two independent experiments. Source data

Unexpectedly, the linear, phage-derived peptide PP1 competes for binding with the biotinylated macrocyclic tracer MC1_biotin_ in the AlphaScreen competition assay, although it shares no obvious sequence similarity with the macrocyclic ligands (Fig. 1c), suggesting a potential peptide binding hot spot on PSMD2. Indeed, PSMD2 mediates the association of many substrate remodeling factors to the proteasome, most notably the deubiquitinase USP14 (refs. 21,22). Thus, we next evaluated whether the macrocyclic ligands altered PSMD2 binding to these endogenous interactors. MC1_biotin_ successfully bound PSMD2 in complex with USP14, providing clear evidence for discrete binding epitopes (Extended Data Fig. 3). Moreover, we performed hydroxyl radical footprinting (HRF) of PSMD2 in the presence of either macrocycle MC1 or USP14. We observed substantial differences in side chain protection for USP14 compared to that of the macrocycle MC1 complex (Supplementary Fig. 6), suggesting that the different binding partners may have distinct impacts on the structural dynamics of PSMD2. The binding of MC1 and MC2 also resulted in strong thermostabilization of PSMD2, increasing the melting temperature (*T*_m_) observed by differential scanning fluorimetry by 9.2 °C (Fig. 1e and Supplementary Fig. 7).

Given the potent affinities and considerable thermal stabilization of PSMD2 observed following macrocycle binding, we next examined MC1 by NMR to structurally characterize this ligand. One-dimensional proton NMR spectra in either DMSO or aqueous buffer revealed that MC1 exists as a dynamic ensemble of structures (Supplementary Fig. 8). Two-dimensional (2D) TOCSY spectra of MC1 confirmed at least three distinct conformers in aqueous solution (Supplementary Fig. 8). Unfortunately, the substantial broadening and spectral overlap due to multiple conformations precluded confident assignment of the unbound MC1 structure.

### Structure of PSMD2 reveals a new ligand binding site

**Fig. 2:**
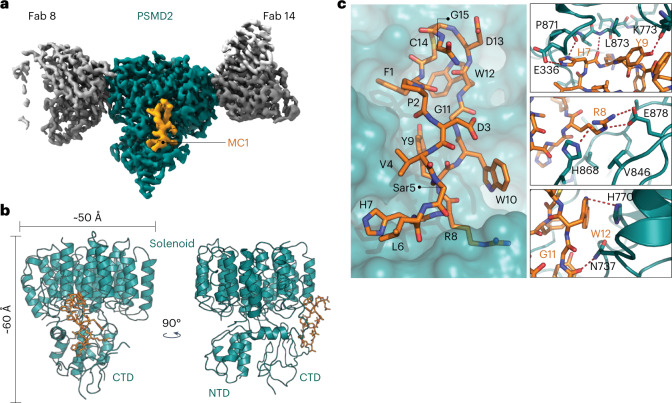
The structure of PSMD2 reveals a new ligand binding site. **a**, Structure of PSMD2 in complex with two Fabs and macrocycle MC1. Macrocycle MC1 (orange) binds between C-terminal and solenoid domains of PSMD2 (teal). PSMD2-interacting Fab chaperones are shown in gray. **b**, Ribbon diagram of PSMD2 (teal) with MC1 shown as orange sticks; CTD, C-terminal domain; NTD, N-terminal domain. **c**, Close-up of the binding site shows residues HRYxGW of macrocycle MC1 interact with K773, L873, E336, H868, E878, V846, H770 and N737 on PSMD2.

**Extended Data Fig. 4:**
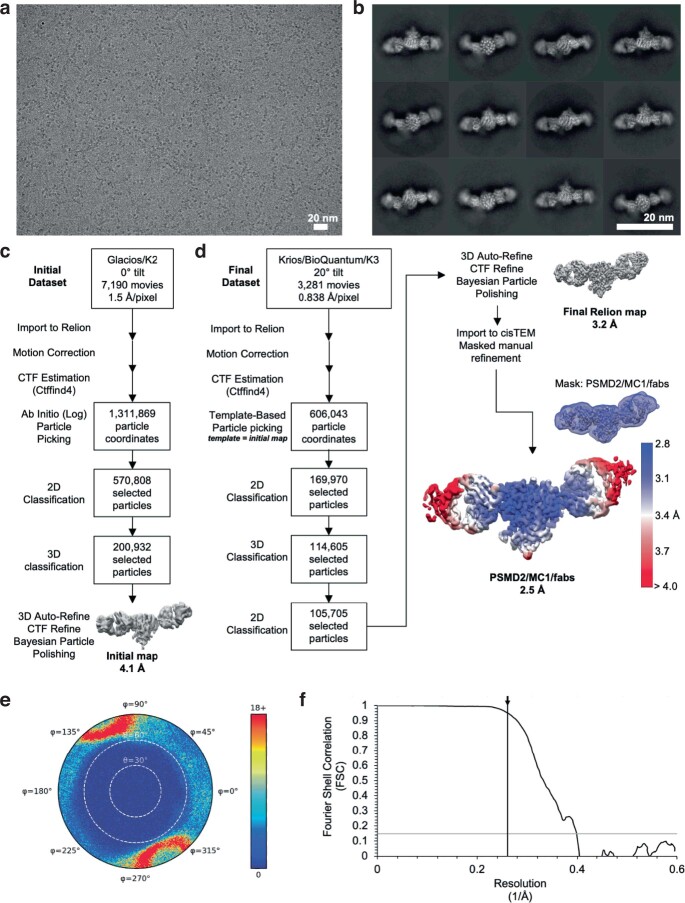
Cryo-EM image processing for the PSMD2:Fabs:MC-1 dataset. **a**, Representative micrograph, Krios data. **b**, Representative 2D class averages, Krios data. **c**, Data processing flowchart for the initial Glacios dataset. **d**, Data processing flow chart for the final Krios dataset. Blue-red color scaling indicates estimated local resolution. **e**, Particle angular (theta/phi) orientation diagram (cisTEM). Scale at right indicates number of particles per angular bin. **f**, Masked Fourier shell correlation (FSC) plots for the two differently masked refinements. Gray horizontal line indicates FSC = 0.143. Vertical line with arrow indicates refinement limit for the final refinement iteration.

To elucidate the molecular basis of the PSMD2–macrocycle interactions, we used cryo-EM and single-particle reconstruction. Determining the structure of PSMD2 at high resolution either alone or within the 26S proteasome has proven challenging due to the relatively small size of this subunit, the preferential orientation of the PSMD2 particles in ice and its intrinsic flexibility within the 26S proteasome. To overcome these limitations, we reconstituted PSMD2 in the presence of MC1 and two fragment antigen-binding (Fab) ‘chaperones’ to determine a structure of the PSMD2–MC1–Fab complex with a resolution of ≈2.5 Å (Fig. 2a, Supplementary Table 3 and Extended Data Fig. 4). Comparison with a published structure of PSMD2 in the context of the 26S proteasome holoenzyme (Protein Data Bank (PDB): 6MSJ; Electron Microscopy Data Bank (EMD): EMD-9221) showed good agreement of the overall topology and fold^23^. Although the majority of the heat repeats in the N-terminal domain of PSMD2 were not resolved in our cryo-EM map, the substantially higher resolution of this map in other regions allowed us to confidently build the structure of PSMD2 (residues S260–N903) and elucidate the details of the PSMD2–MC1 interaction (Supplementary Fig. 9).

**Fig. 3:**
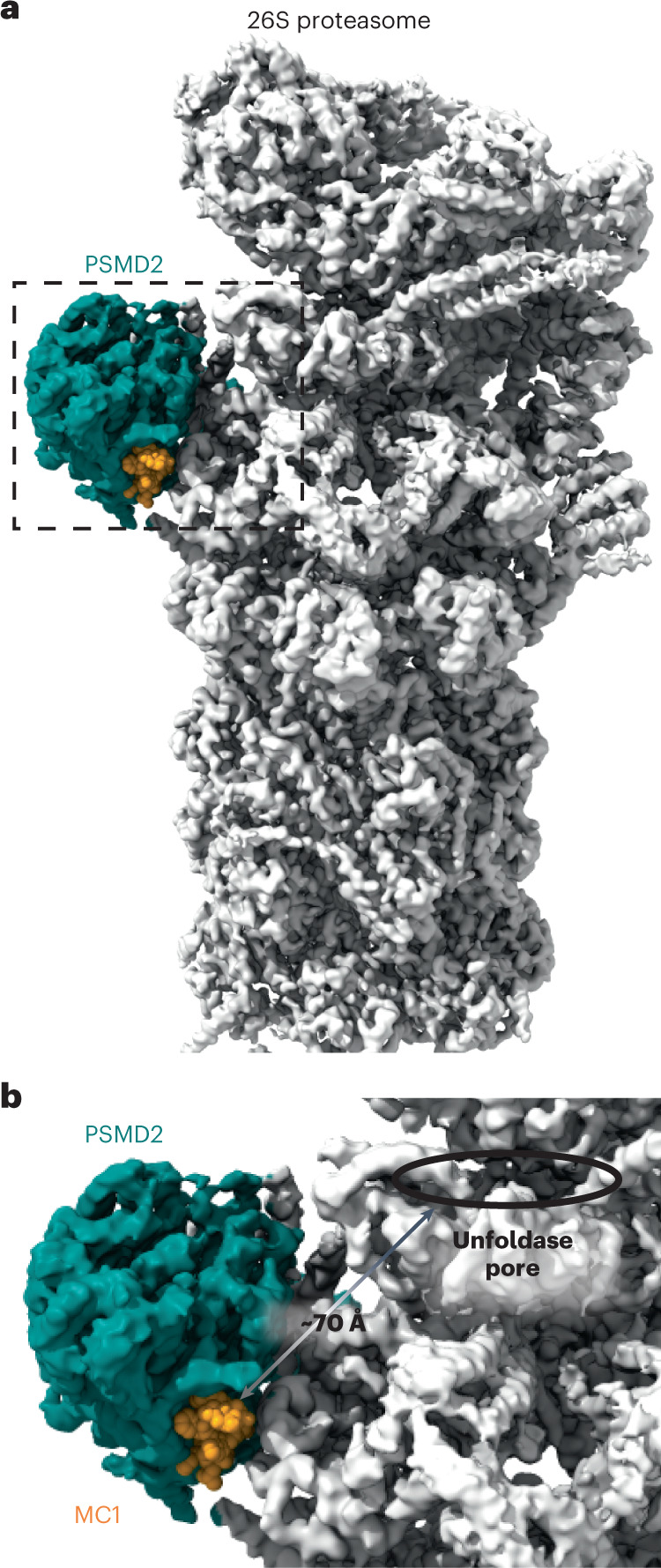
The macrocycle epitope is positioned close to the AAA^+^ unfoldase of the 26S proteasome, making it ideal for target delivery. **a**, Overlay of the PSMD2–MC1 structure with the 26S proteasome structure 6MSJ. **b**, Zoom-in of the binding site shows that MC1 binds PSMD2 near the proteasomal unfoldase and is well positioned for substrate delivery.

**Extended Data Fig. 5:**
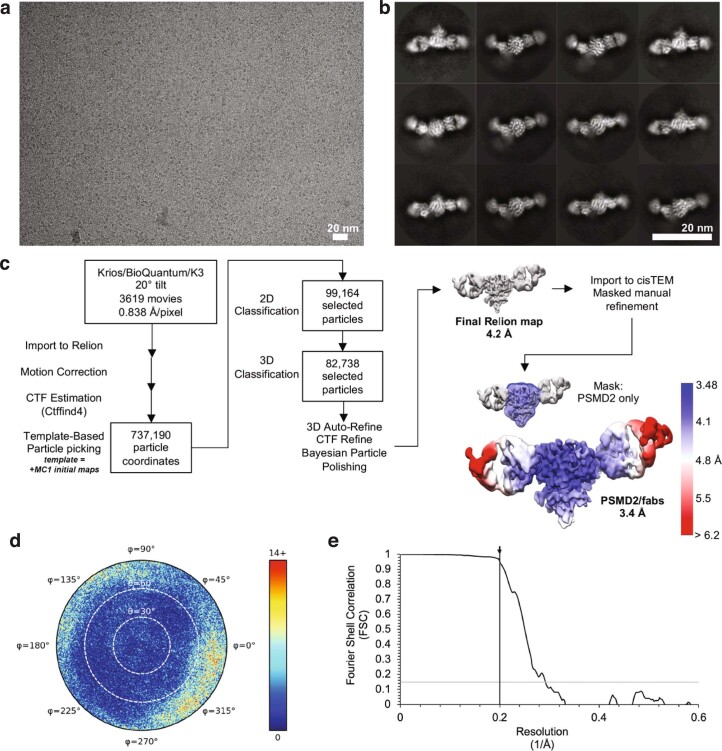
Cryo-EM image processing for the PSMD2:Fab dataset. **a**, Representative micrograph. **b**, Representative 2D class averages. **c**, Image processing flowchart. Blue-red color scaling indicates estimated local resolution. **d**, Particle angular (theta/phi) orientation diagram (cisTEM). Scale at right indicates number of particles per angular bin. **e**, Masked Fourier shell correlation (FSC) plots for final masked refinement. Gray horizontal line indicates FSC = 0.143. Vertical line with arrow indicates refinement limit for the final refinement iterations.

As illustrated in Fig. 2b, MC1 binds between the C-terminal domain and the helical solenoid of PSMD2 in a region that is distinct from the previously identified binding sites for USP14 and RAD23 (ref. 22). Analysis of the binding pocket showed that MC1 uses the HRYxGW motif to associate with PSMD2 (Fig. 2c), consistent with our mutagenesis results (Fig. 1c). Specifically, MC1_H7_ establishes polar contacts with the PSMD2_E336_ side chain and the backbone carbonyls of PSMD2_P871_ and PSMD2_L873_. The neighboring MC1_R8_ residue establishes a charge interaction with PSMD2_E878_ and hydrogen bonds with PSMD2_H868_. Residues Y9 and W10 hydrogen bond with the backbone carbonyl of PSMD2_K773_ and PSMD2_N737_, respectively. Finally, W12 establishes π-stacking interactions with PSMD2_H770_. Together these contacts span the wide and fairly shallow binding pocket with a buried surface area of 720.3 Å^2^, with the majority of interactions mediated by residues at opposite ends of the macrocycle’s long axis (~20 Å apart). We assessed the structural impact of MC1 binding on PSMD2 conformation by solving a PSMD2–Fab apo structure (Extended Data Fig. 5). Comparison between the PSMD2–MC1 complex and PSMD2 apo revealed that association with MC1 does not alter PSMD2 structure, indicating that this binding pocket is preformed and not induced by ligand binding (Supplementary Fig. 10). Moreover, this site is accessible in the 26S proteasome and situated adjacent to the AAA^+^ unfoldase, suggesting that it could serve as an effective location to tether substrates for targeted degradation (Fig. 3).

### Macrocycle-derived CIDEs bind PSMD2 and BRD4

**Fig. 4:**
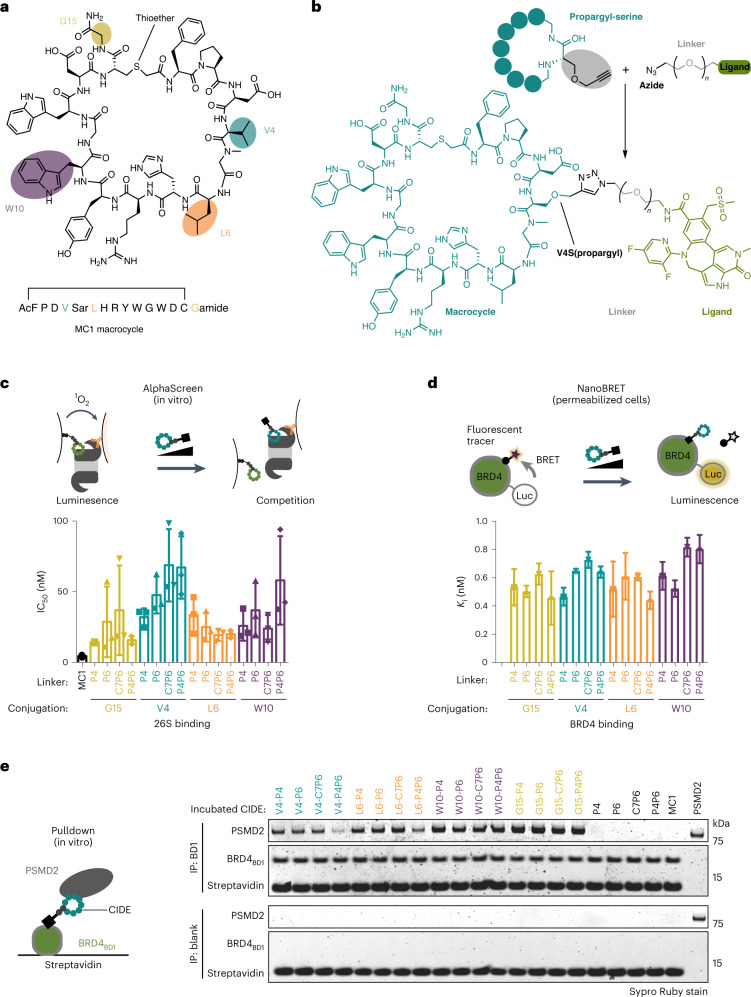
Macrocycle-derived CIDEs bind PSMD2 and BRD4 with high affinity. **a**, Schematic of MC1 and four amino acids chosen as conjugation points. AcF, acetyl l-phenylalanine. **b**, Click-based strategy used to conjugate a BRD4 ligand (BETi) with various linkers to each macrocycle via a propargyl-serine introduced at each of the positions highlighted in **a**. **c**,**d**, PSMD2-based CIDEs (Supplementary Table 3) retain nanomolar binding affinities to purified 26S proteasome (**c**) and BRD4 (**d**) in permeabilized cells; P4, PEG4; P6, PEG6; C7P6, -(CH_2_)_6_CONH-(C7) + PEG6; P4P6, PEG4 + PEG6. Bars represent the mean ± s.d. of at least three independent experiments. **e**, CIDEs mediate formation of the PSMD2–BRD4_BD1_ ternary complex as observed by pulldown assay. Recombinant PSMD2 co-purifies with biotinylated BD1 pulled down with Streptavidin beads in the presence of all 16 CIDE compounds but not in the presence of BETi linker compound without macrocycle or macrocycle alone. Lanes are color coded based on their conjugation point in **a**; IP, immunoprecipitation. Data were reproduced in two independent experiments. Source data

Having shown that MC1 binds PSMD2 with high affinity in close proximity to the 26S proteasome pore, we wanted to test whether this ligand can induce selective protein degradation by attaching the target ligand that recruits the model target protein BRD4. Because the linker attachment site is a critical parameter of heterobifunctional degrader optimization, we explored different exit vectors on MC1. In addition to the mRNA attachment vector located at the C-terminal G15 residue, positions within MC1, such as V4, L6 and W10, that were insensitive to alanine or lysine substitution and solvent accessible in the PSMD2–macrocycle complex provide potential conjugation vectors for the generation of chimeric CIDE molecules (Fig. 4a and Supplementary Fig. 11). To synthesize the MC1 ligand as a heterobifunctional CIDE, we used a click chemistry-based strategy at the aforementioned positions on the macrocycle to conjugate MC1 to a high-affinity BRD4 sulfone ligand (BETi^24^) with PEG-based linkers of differing lengths (Fig. 4b and Supplementary Table 4). This approach allowed the efficient generation of 16 CIDE molecules with different combinations of attachment geometries and linker lengths. We confirmed that these CIDEs still bound to the 26S proteasome using the established AlphaScreen assay (Fig. 4c) and that they maintained binding to BRD4 using a nano-bioluminescence resonance energy transfer (NanoBRET) assay in permeabilized cells expressing BRD4–nanoluciferase to quantify the interaction (Fig. 4d). Finally, the compounds were also able to mediate the critical PSMD2–BRD4 ternary complex required for efficient degradation^25–27^, as observed by pulldown of PSMD2 by biotinylated BRD4 bromodomain 1 (BRD4_BD1_) and BRD4_BD2_ domains in the presence of the heterobifunctional CIDE molecules but not the BETi linker or MC1 building blocks using purified proteins (Fig. 4e and Supplementary Fig. 12a). Moreover, one of the CIDE compounds was also confirmed to mediate ternary complex formation between the 26S proteasome and BRD4_BD1_ in cell lysates (see BetCIDE8 V4-P6 in Supplementary Table 4 (referred to subsequently as l-CIDE) and Supplementary Fig. 12b).

### MC1_Cy5.5_ conjugate is permeable in KPL-4 cells

**Fig. 5:**
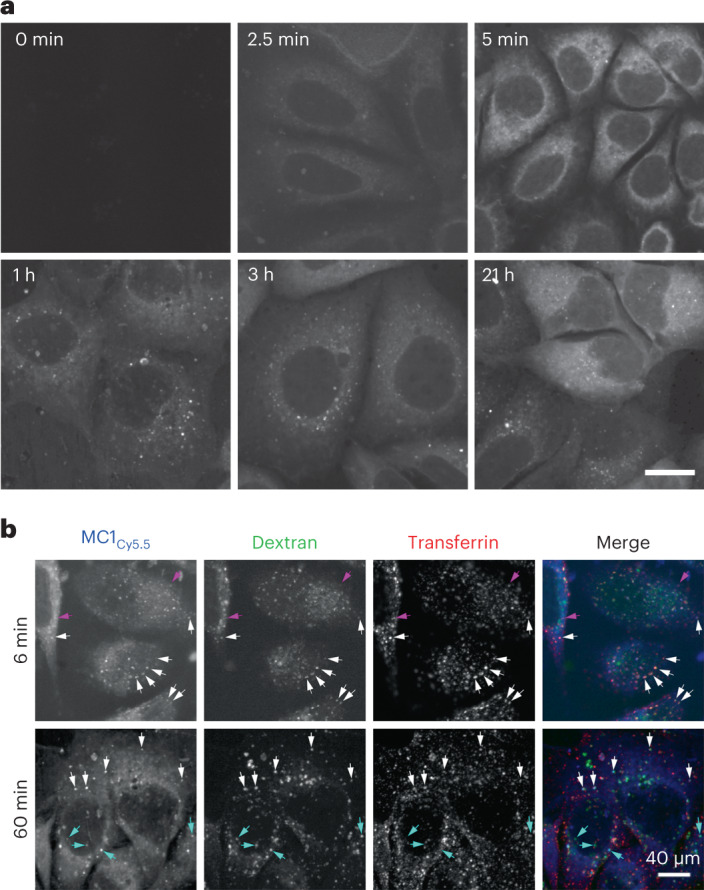
MC1 is delivered to the cytoplasm and endosomes. **a**, KPL-4 cells were incubated with MC1_Cy5.5_ for the indicated times and washed, fixed and imaged by spinning disk confocal microscopy; 1-µm projections (5 × 0.25 µm) are shown. Although the cytoplasmic signal at 2.5 min was faint, it was distinguishable from the vehicle control (0 min). MC1 was first detected in the cytoplasm before endosomal accumulation. Note that the 3-h and 21-h sample timepoints were collected on a different day as the others; scale bar, 40 µm. **b**, At early timepoints (6 min), MC1_Cy5.5_ puncta (blue) colocalized well with two co-incubated endocytic markers, the bulk flow marker Oregon Green Dextran (green) and the clathrin marker transferrin (white arrows for triple positives) or with transferrin alone (pink arrows), whereas by 60 min, there was more robust colocalization with dextran (cyan arrows), indicating that endocytosed MC1_Cy5.5_ takes the bulk flow pathway rather than recycling; scale bar, 40 µm. Data are representative of at least two independent experiments.

**Extended Data Fig. 6:**
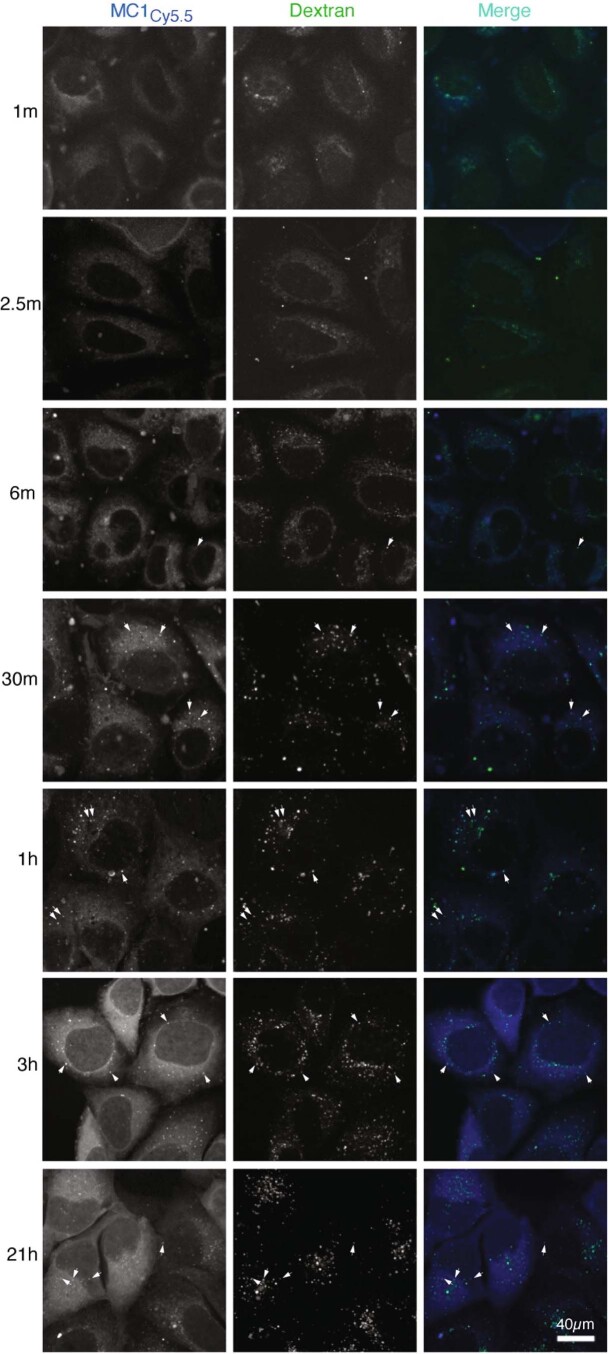
Time-course of MC1_Cy5.5_ uptake versus dextran. Cells were co-incubated as in Fig. 5 with MC1_Cy5.5_ and the fluid phase endocytic marker Oregon Green dextran for the indicated times from one minute to overnight, then washed, fixed and imaged. The MC1_Cy5.5_ puncta (first appearing after 6 minutes) colocalized with dextran at all time points, indicating they are endosomal (arrows). Scale bar is 40 µm. Data were reproduced in at least 2 independent experiments.

**Extended Data Fig. 7:**
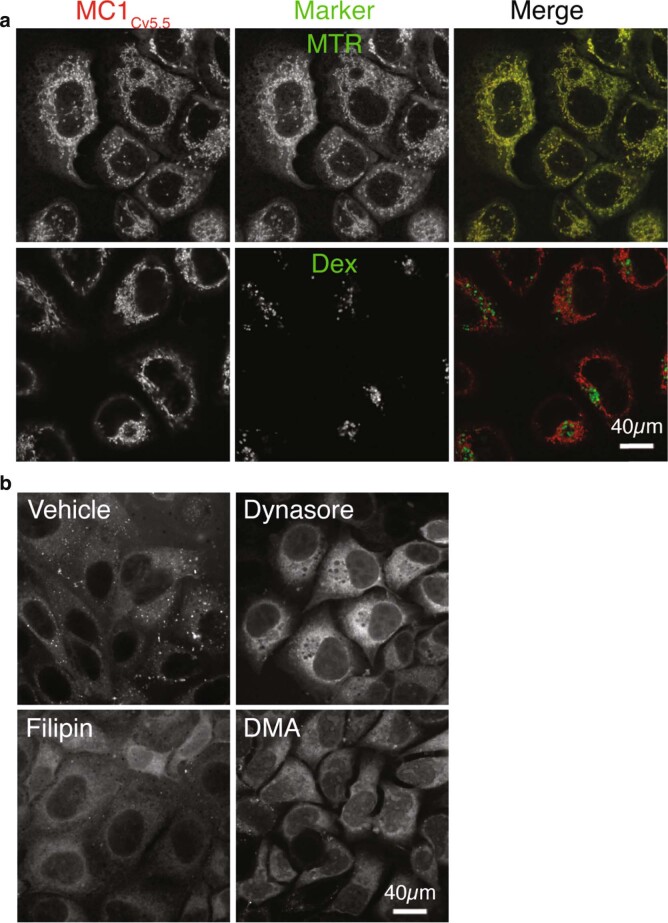
Cytoplasmic delivery of MC1_Cy5.5_ is not mediated by the fluorophore and is independent of endocytosis. **a**, Free Cy5.5 dye accumulates in mitochondria. Upper panels: KPL-4 cells were pre-incubated with Mitotracker^TM^ Red FM (MTR, shown in the green channel) for 20 min, washed twice, then free, capped Cy5.5 dye (red channel) was added for 9 min and imaged. Lower panels: cells were pre-loaded overnight with Oregon Green dextran (Dex, lower panels), washed, then free Cy5.5 dye was added for 6 and imaged. The free Cy5.5 fluorophore colocalizes perfectly with the mitochondrial MitoTracker^TM^, clearly distinct from the fluid phase endocytic marker, dextran, and is absent from the cytoplasm (in contrast to MC1_Cy5.5_). Scale bar is 40 µm. **b**, Three independent endocytic inhibitors prevent endosomal uptake of MC1_Cy5.5_ with no detriment to its cytoplasmic delivery. KPL-4 cells were preincubated for 6 min in serum-free media with the dynamin inhibitor Dynasore (which inhibits clathrin-mediated and caveolar endocytosis); or for 30 min with 2% methanol (vehicle), 5 µg ml^−1^ cholesterol extractor filipin (an inhibitor of lipid raft and caveolar endocytosis), or 200 µg ml^−1^ dimethyl amiloride (DMA), a micropinocytosis inhibitor. Cells were then incubated for a further 30 min with 2 µM MC1_Cy5.5_ and fresh inhibitors before washing, fixing and imaging. Scale bar is 40 µm. Panels are representative of at least 2 experiments. All three inhibitors prevented endocytosis of MC1_Cy5.5_, as evidenced by loss of punctate endosomal signal (confirming that uptake in the vehicle control is mediated by nonspecific fluid phase endocytosis), but none of them prevented MC1_Cy5.5_ accumulation in the cytoplasm. Data are representative of 2 independent experiments.

To evaluate the potential permeability of a macrocycle-based CIDE, we conjugated Cyanine 5.5 (Cy5.5) to the V4 position of MC1 (see MC1_Cy5.5_, Supplementary Table 4) and monitored cellular uptake by fluorescence microscopy. Direct evidence that MC1 is readily cell permeable was observed, as MC1_Cy5.5_ was detected in the cytoplasm as early as 1–2 min after application, increasing in intensity over time (Fig. 5a and Extended Data Fig. 6). This was not an artifact of fluorophore conjugation, because free (capped) Cy5.5 accumulated instead in mitochondria (Extended Data Fig. 7a). MC1_Cy5.5_ was subsequently also visible in endosomes (a few at 5 min, increasing in number for up to 1 h and remaining steady through 21 h). This is likely due to micropinocytosis, because MC1_Cy5.5_ colocalized at all timepoints with the fluid-phase marker dextran (Extended Data Fig. 6) concurrent with the clathrin-mediated cargo transferrin at early timepoints before transferrin is recycled (Fig. 5b). However, cytoplasmic delivery was likely independent of endocytosis because it preceded endosomal delivery and was not prevented by three distinct endocytic inhibitors (Extended Data Fig. 7b). Together, these studies suggest that in spite of the size and peptidic nature of the macrocycle, MC1-based conjugates can achieve cellular entry and access the cytoplasm.

### PSMD2–BRD4 CIDEs mediate degradation of BRD4 in cells

**Fig. 6:**
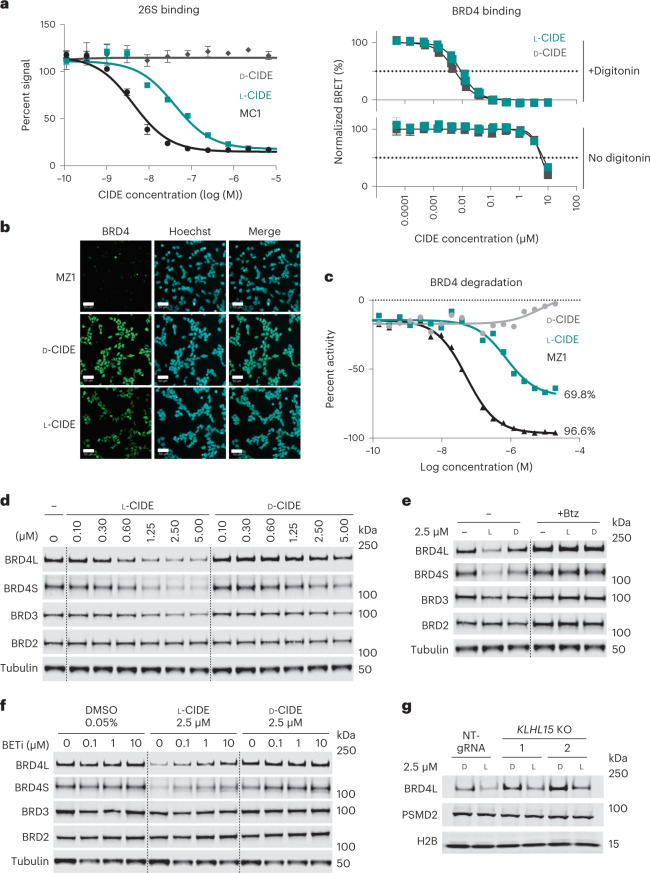
PSMD2-based CIDEs mediate degradation of BRD4 in HEK293 cells. **a**, An all-d-enantiomer macrocycle conjugated to BRD4 ligand provides a negative control CIDE with differential PSMD2 binding (left) but equivalent permeability and BRD4 binding (right). Data represent the mean ± s.d. for three independent experiments. **b**, Degradation of BRD4 observed by immunofluorescence shows an on-target mechanism of action for PSMD2-based CIDEs. Cells were treated with 10 μM MZ1, l-CIDE or d-CIDE; scale bar, 50 µm. **c**, IC_50_ curves based on quantitation of immunofluorescence in **b**, with 0% activity defined by DMSO control. Results were reproduced in two independent experiments. **d**, Degradation of BRD4 observed by western blotting in HEK293 cells treated with the active l-CIDE but not the inactive d-CIDE. Blots are representative of five independent experiments. **e**, BRD4 degradation is abrogated by proteasome inhibition by bortezomib (Btz). Blots are representative of two independent experiments. **f**, BRD4 degradation is competed by co-treatment with BRD4 inhibitor BETi. Data represent a single experiment. **g**, l-CIDE-mediated BRD4 degradation occurs in *KLHL15*-knockout (KO) cell lines. The blot is representative of three independent experiments; NT-gRNA, non-targeting gRNA. Source data

**Extended Data Fig. 8:**
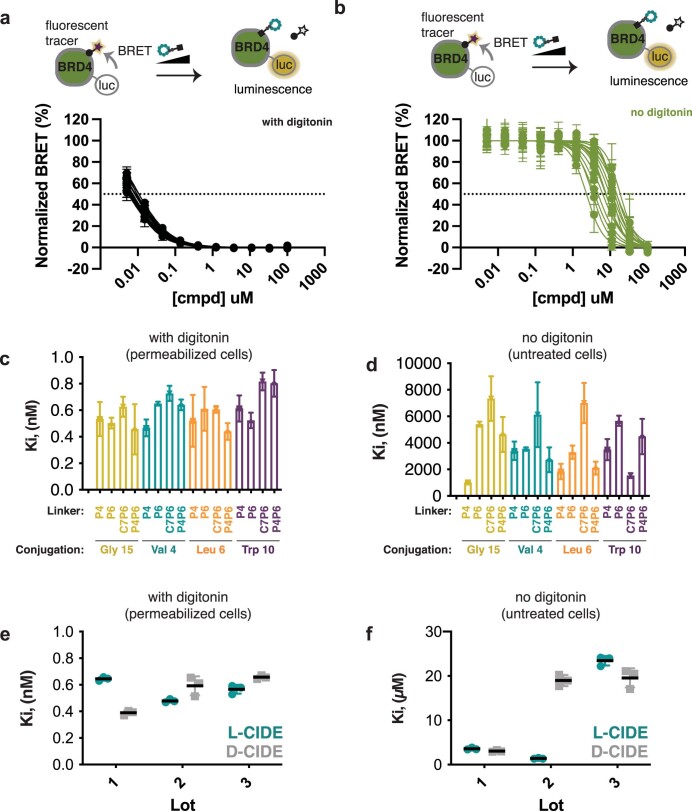
Summary of CIDE binding to BRD4 in HEK293 cells with and without digitonin. Representative curves for BRD4 binding shown in cells with (**a**) digitonin permeabilization (black) and (**b**) without permeabilization (green) for 16 BETcides. Data are presented as mean values ± standard deviation from three independent experiments. **c** and **d**, K_i_ values for 16 CIDEs in HEK293 cells with permeabilization (**c**, included from Fig. 4 for reference) and without permeabilization (**d**), where bars represent mean ± standard deviation of three independent experiments. **e** and **f**, Different lots of L-CIDE (teal) and D-CIDE (gray) show similar binding in cells with digitonin permeabilization (**e**) but not in cells without digitonin (**f**) suggesting variable permeabilities. Black bars represent mean ± standard deviation of at least two independent experiments.

We next assessed the cell permeability of the CIDEs by measuring BRD4 binding by NanoBRET in HEK293 cells in the absence of a cell permeabilization agent^28^. Surprisingly, despite their relatively large size, several CIDEs showed observable binding to BRD4 even in the absence of digitonin, indicating that they show some degree of cell permeability (Extended Data Fig. 8). One of the most permeable molecules (l-CIDE, Supplementary Table 4) was also synthesized using a macrocyclic building block composed entirely of d-amino acids. The resulting molecule (referred to here as d-CIDE) provided an enantiomeric control with theoretically equivalent physicochemical properties and conformational dynamics to l-CIDE. Because of the inability to knock down the essential gene *PSMD2* and the unpredictable and/or variable permeability of macrocyclic point mutants, an enantiomeric control was the best way to assess PSMD2 dependence of these CIDEs. As expected, reversing the stereochemistry for the d-CIDE abrogated PSMD2 binding, but because the BRD4 ligand is achiral, both compounds bound BRD4 identically in permeabilized cells. Furthermore, l-CIDE and d-CIDE demonstrated equivalent binding to BRD4 in non-permeabilized cells, albeit at *K*_i_ values ~1,000-fold greater than in the presence of digitonin (Fig. 6a), indicating that a nonspecific transport mechanism likely governed the cellular entry of these compounds. Additional synthetic lots of the enantiomers showed modest two- to tenfold variability in the degree of cell permeability (Extended Data Fig. 8), so care was taken to conduct all subsequent cellular experiments using l-CIDE and d-CIDE lot 1, which had comparable permeability.

**Extended Data Fig. 9:**
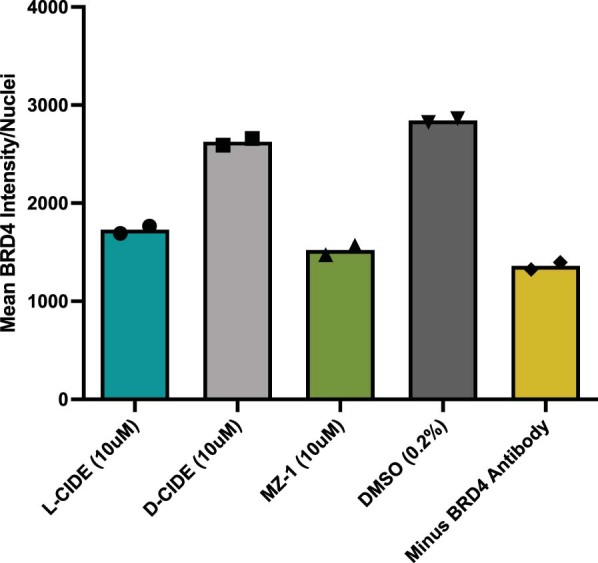
Controls for BRD4 degradation monitoring by immunofluorescence. Quantitated fluorescence signals form HEK 293 cells treated with L-CIDE, D-CIDE, bench mark degrader MZ1, DMSO. Control condition lacking BRD4 primary antibody (minus BRD4 Antibody, gold) shows the background fluorescence from secondary antibody. Bars represent mean of two technical replicates.

**Extended Data Fig. 10:**
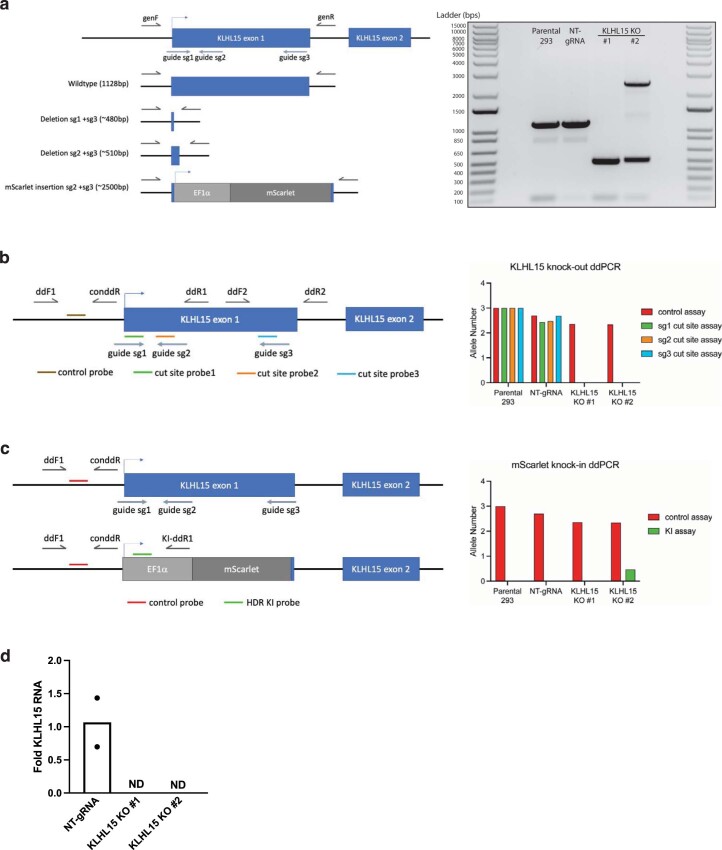
Confirmation of KLHL15 deletion in 293 s. **a**, Genomic PCR of control or KLHL15 KO clones. Approximate base pair sizes are shown based upon editing with the indicated guides. **b** and **c**, Schematic of the KHLH15 locus showing location of sgRNAs (blue arrows), primers (gray arrows) and probes (colored bars) for the ddPCR knock-out (**b**) and knock-in (**c**) assay designs. Copy number of each clone and non-targeting gRNA pool is shown relative to the triploid parental 293 cell line in the control or knockout assay (**b**) and control or knock-in assay (**c**). **d**, KLHL15 qPCR of RNA isolatedfrom non-targeting gRNA cells, KLHL15 KO clone #1, and KLHL15 KO clone #2. ND is not detected. Data are presented as mean values from two independent experiments. Source data

To further evaluate the degradation activity of these molecules, we treated HEK293 cells with a concentration range of both compounds and previously published compound MZ1 (ref. 9) as a benchmark and monitored BRD4 degradation by immunofluorescence (Fig. 6b and Extended Data Fig. 9). Here, we observed a loss of BRD4 after treatment with l-CIDE but not with the negative control. l-CIDE-mediated degradation was also dose dependent, with a half-maximum degradation concentration (DC_50_) of 0.73 µM (Fig. 6c). Western blotting analysis showed degradation of both long and short forms of BRD4 and moderate depletion of the BRD3 paralog (Fig. 6d and Supplementary Table 5) after treatment with the active l-CIDE. Observed degradation increased over sustained treatment with l-CIDE, showing robust degradation over 72 h (Supplementary Fig. 13). This degradation was abrogated after loss of PSMD2 binding with the enantiomeric d-CIDE control, with inhibition of the proteasome by bortezomib or with a competing excess of the BRD4 ligand, all congruent with a degrader mechanism using direct proteasome recruitment (Figs. 6e,f and Supplementary Tables 6 and 7). Finally, to investigate if l-CIDE-induced BRD4 degradation could be mediated in part by CUL3 ligase via binding to the KLHL15 adapter that we observed in AP–MS experiments, we generated *KLHL15*-knockout clones using CRISPR–Cas9 protocols. Editing of the *KLHL15* locus and loss of the *KLHL15* transcript were confirmed via genomic PCR, droplet digital PCR (ddPCR) and quantitative PCR (qPCR; Extended Data Fig. 10). Treatment with l-CIDE in *KLHL15*-knockout clones resulted in similar degradation of BRD4 as in control non-targeting guide RNA (gRNA) cells (Fig. 6g and Supplementary Table 8). Given that degradation still occurred in the absence of KLHL15 and the strong evidence for l-CIDE-induced ternary complex formation of PSMD and BRD4, these data collectively demonstrate proof of concept for targeted protein degradation through direct recruitment to the proteasome.

## Discussion

Most proteasomal substrates are targeted for degradation via ubiquitin modification; yet, a subset do not rely on this post-translational mark for proteolytic turnover. Well-studied examples of such ubiquitin-independent proteasomal substrates include ornithine decarboxylase and thymidylate synthase^29^. Detailed examination of the proteasome substrates and subsequent engineering studies have revealed that they contain two key degradative elements: a motif that can bind to the proteasome and an unstructured region of sufficient size to productively engage with the unfolding and/or proteolytic machinery^30,31^. Thus, many proteins could feasibly be degraded in the absence of ubiquitin if given sufficient tethering to the 26S proteasome^30–32^.

Here, we use mRNA display to rapidly identify peptidic macrocycles that bind PSMD2, an essential component of the 26S proteasome. PSMD2 serves as a docking site for a number of substrate remodeling factors on the 19S regulatory particle and interacts with the ring of ATPases that unfold substrates for entry into the proteolytic chamber of the 20S core particle^14,21^. The highest-affinity macrocyclic ligands associate with PSMD2, both alone and in the context of the 26S proteasome complex, using a solvent-accessible pocket that is distinct from partner-binding sites previously described for ubiquitin, RAD23b and USP14 (refs. 21,22). This newly identified binding groove is present in the non-liganded state of PSMD2 and is located ~70 Å from the entry pore of the ATPase assembly and is therefore well positioned for substrate delivery. Recognition of this site is primarily mediated by the HRYxGW peptide motif, with other positions in the macrocycle that point into solvent providing potential attachment vectors for the synthesis of chimeric molecules aimed at tethering target proteins to the proteasome for destruction.

As a proof of concept, we targeted BRD4 (ref. 33) for proteasomal degradation by generating heterobifunctional CIDE compounds composed of the macrocyclic PSMD2 ligand and a potent BET ligand attached through PEG-based linkers. The majority of the resulting CIDEs were competent to form ternary complexes between isolated PSMD2 and BRD4 bromodomains and to bind to the fully assembled proteasome. Furthermore, we measured CIDE-mediated degradation of endogenous BRD4 in cells, thus demonstrating engineered target turnover through direct recruitment to the proteasome using a new PSMD2 ligand. This cellular activity is striking given the limited permeability and large size of these tool compounds and suggests that a degrader molecule with improved chemical properties could promise great efficacy. Although currently not as potent as optimized ligase-based BRD4 degraders like MZ1, the CIDE molecules described here serve as an important proof of principle for targeted degradation via direct 26S proteasome recruitment.

Engineered degradation by direct targeting to the proteasome has a number of unique advantages over ligase-based degraders. CIDE-mediated recruitment to the 26S proteasome bypasses the need for available lysines for ubiquitination and subsequent proteasome binding, although substrates could still be remodeled on the proteasome by associated ubiquitination machinery. The abundant expression of the 26S proteasome complex makes it amenable for degradation of cytosolic and nuclear targets in all tissues. Moreover, recent work examining resistance mechanisms to heterobifunctional degraders involved mutations in the ubiquitination cascade rather than disruption of ligand binding to the target^34–36^. Targeting to PSMD2 could circumvent this resistance mechanism, given the 26S proteasome’s crucial function and, specifically, PSMD2’s critical role in 26S assembly and function^37–39^. Indeed, direct proteasome-targeting degraders are an underexplored and unique strategy for targeted protein degradation that could dramatically expand the number of protein targets amenable to inhibition by degradation.

## Methods

### Antibodies and reagents

Antibodies to the indicated proteins were purchased from the following vendors: BRD4 (ab128874), BRD2 (ab139690), PSMD14 (ab109123) and PSMD1 (ab140682; all Abcam); BRD3 (A302-368A; Bethyl); tubulin (926-42211; LICOR Biosciences); PSMD4 (3846), PSMD2 (25430), biotin (5597) and H2B (12364; Cell Signaling Technology) and PSMA1 (BML-PW8100-0100; ENZO Life Sciences). IRDye-800 anti-rabbit (926-32211) and IRDye-680RD anti-mouse (926-68072) secondary antibodies were purchased from LICOR Biosciences. Bortezomib (179324-69-7) was purchased from Calbiochem. Streptavidin magnetic beads (88816), NeutrAvidin agarose beads (29200), Lipofectamine RNAiMAX transfection reagent (13778075), RNA-to-*C*_t_ 1-Step kit (4392653) and TaqMan gene expression assays were purchased from Thermo Fisher. An RNeasy Plus mini RNA extraction kit (74134) was purchased from Qiagen.

### Protein purification

Recombinant PSMD2 was purified from BL21 gold (DE3) cells (Invitrogen) transformed with a pET 52b plasmid containing full-length human PSMD2 fused to an N-terminal His_6_ tag and TEV site or to N-terminal His_6_-TEV-Avi tags. For biotinylated PSMD2, cells were transformed with both the N-terminal His_6_-TEV-Avi-tagged PSMD2 plasmid and a plasmid containing untagged BirA enzyme with a chloramphenicol marker. Three liters of cells were cultured at 37 °C in TB medium supplemented with carbenicillin for His_6_-tagged PSMD2 and TB medium supplemented with carbenicillin, chloramphenicol and 50 µM biotin for Avi-tagged PSMD2 until an optical density at 600 nm (OD_600_) of 0.6–1.0 was reached; cells were induced with 0.4 mM IPTG overnight at 16 °C. Cell pellets were lysed with BPER (Thermo Fisher) supplemented with 150 mM NaCl, 5% (vol/vol) glycerol, 25 mM imidazole, 0.5 mM TCEP and complete EDTA-free protease inhibitor tablets (Roche). Lysates were clarified by centrifugation and incubated in batches with 5 ml of Ni-NTA agarose (Qiagen). Resin was loaded onto columns and washed and eluted with 250 mM imidazole. Eluates were then dialyzed into 50 mM HEPES (pH 7.5), 100 mM NaCl, 25 mM imidazole, 5% (vol/vol) glycerol and 0.5 mM TCEP with 1 µg ml^−1^ TEV protein to cleave the His_6_ tag. Dialysates were then passed over another 5 ml of Ni-NTA agarose (Qiagen) to remove any remaining tagged protein. The cleaved protein was then passed over a MonoQ 5/50 column (Cytiva) and eluted with a gradient of 0.05 to 1,000 mM NaCl over 50 column volumes (CV). Peak fractions were concentrated in a 30,000-molecular weight cutoff (MWCO) concentrator (EMD Millipore) and injected onto a Superdex 200 16/60 column (Cytiva), and the peak was concentrated in a 30,000-MWCO concentrator (EMD Millipore).

Recombinant USP14 was purified from BL21 gold (DE3) cells (Invitrogen) transformed with a pET 52b plasmid containing full-length human USP14 fused to a C-terminal His_6_ tag. USP14 was cultured in 1 liter of TB autoinduction medium for 3 h at 37 °C, followed by expression for 64 h at 16 °C. Cells were resuspended in 50 mM HEPES (pH 7.5), 100 mM KCl, 100 mM NaCl, 10% glycerol, 20 mM imidazole, 0.5 mM TCEP and complete EDTA-free protease inhibitor tablets (Roche). Lysates were clarified by centrifugation and incubated in batch with 5 ml of Ni-NTA agarose (Qiagen). Resin was loaded onto columns and washed and eluted with 250 mM imidazole. Eluate was concentrated in a 30,000-MWCO concentrator (EMD Millipore) and injected into a Superdex 200 (Cytiva) equilibrated in 25 mM HEPES (pH 7.5), 5% glycerol, 100 mM NaCl, 100 mM KCl and 0.5 mM TCEP. Peak fractions were concentrated in a 30,000-MWCO concentrator (EMD Millipore).

Recombinant linear tetraubiquitins (Ub4lin) with and without an N-terminal biotin were purified as previously described^40^. Briefly, BL21 (DE3) pLysS cells were transformed with pET 15b plasmid containing linear tetraubiquitin (M1-G304) with N-terminal His_6_-thrombin or His_6_-thrombin-Avi tags. For biotinylated tetraubiquitin, cells were cotransformed with both the tetraubiquitin plasmid and a plasmid containing untagged BirA enzyme with a chloramphenicol marker. Cells were cultured at 37 °C in TB medium buffered with 100 mM MOPS until an OD_600_ of 1.5 was reached. Cells were then cooled to 16 °C and induced with 0.5 mM IPTG. Cells were collected, frozen, resuspended in 40 mM Tris (pH 8.0), 0.3 M NaCl and EDTA-free complete protease inhibitor tablets (Roche) and lysed via microfluidizer. Lysates were clarified by centrifugation, passed over Ni-NTA agarose (Qiagen) and eluted with imidazole. Imidazole was dialyzed out, and His_6_ tags were cleaved by treatment with thrombin. Uncleaved protein was captured by subsequent pass through Ni-NTA agarose, and the cleaved fraction was then purified further by injecting on a HiLoad 26/60 Superdex 75 column (Cytiva) in 20 mM Tris (pH 7.5), 150 mM NaCl and 0.5 mM TCEP. Fractions were monitored by SDS–PAGE.

### Selection of PSMD2-binding linear peptide by phage display

Phage display using N-terminally expressed peptide libraries was conducted as previously published^41^. Briefly, a naive library of 8- to 16-amino-acid-long peptide sequences fused to the N terminus of M13 major coat protein (p8) was used for selection. Phage pools were cycled through four rounds of binding selections using 20 or 10 µg of biotinylated PSMD2 in a buffer consisting of PBS, 0.5% bovine serum albumin (BSA) and 0.1% Tween-20. After four rounds of selection, individual phage clones were picked and evaluated by phage ELISA using PSMD2 immobilized onto Maxisorp plates coated with NeutrAvidin (Nunc). Clones with the highest signal-to-noise ratio were then selected for peptide synthesis and evaluation by surface plasmon resonance (SPR). Highest-binding clones were then subjected to affinity maturation.

Affinity-matured libraries were generated via Kunkel mutagenesis and soft randomization of the parent sequence from naive panning. Phage binding selection was then repeated as described above except with 20, 2 and 0.02 µg of PSMD2 and up to 30-min wash times. Candidates were evaluated as described above.

### Macrocyclic peptide library design and selection of PSMD-binding macrocycles

A thioether-macrocyclic peptide library was constructed by using ClAc-F as an initiator in a genetically reprogrammed in vitro translation system^42^. The two genetic codes NNW and NNU were designed. The NNW code contained all 20 natural amino acids. The NNU code contained four *N*-methyl amino acids: *N*-methyl-l-phenylalanine (MeF; codon TTC), *N*-methyl-l-glycine (MeG; codon ATC), *N*-methyl-l-norleucine (MeNle; codon ACC) and *N*-methyl-l-alanine (MeA; codon GCC) in addition to 11 natural amino acids (asparagine, aspartic acid, arginine, glycine, histidine, leucine, proline, serine, tryptophan, tyrosine and valine). The two mRNA libraries, referred to as the NNW or NNU library, were designed to have an AUG (ClAc-F) initiator codon followed by 8 to 11 NNW or NNU codons, which encode random amino acid residues, followed by a fixed UGG codon that assigns cysteine and a sequence encoding a G4S2 peptide linker^15–18^.

After in vitro translation, a thioether bond formed spontaneously between the N-terminal ClAc group of the initiator l-phenylalanine residue and the sulfhydryl group of a downstream cysteine residue to generate the macrocyclic peptides.

### Selection of PSMD2-binding macrocycles

Affinity selection of peptidic macrocycles binding to PSMD2 was conducted using Avi-tagged PSMD2 purified as described above. Briefly, 10 µM mRNA library was hybridized with a peptide linker (11 mM) for 3 min at room temperature. The mRNA library was translated for 30 min at 37 °C using the reprogrammed in vitro translation system to produce the peptide–mRNA fusion library^15–18^. Each reaction contained 2 μM mRNA–peptide linker conjugate, 12.5 µM initiator tRNA (tRNA^fMet^ aminoacylated with ClAc-l-phenylalanine) and 25 µM of each elongator tRNA (EnAsn) aminoacylated with the specified non-canonical/canonical amino acids. In the first round of selections, translation was performed at a 100-μl scale. After translation, the reaction was quenched with 17 mM EDTA. The product was subsequently reverse transcribed using RNase H minus reverse transcriptase (Promega) at 42 °C for 30 min, and buffer was exchanged for HBS-T (25 mM HEPES-NaOH, 150 mM NaCl_2_ and 0.05% Tween-20). The peptide–mRNA/cDNA mixture was incubated with 250 nM biotinylated PSMD2 for 60 min at 4 °C, followed by incubation with streptavidin-coated beads (Dynabeads M-280 Streptavidin, Thermo Fisher) for 10 min for the selection of binders. The resulting beads were washed with cold HBS-T buffer three times, and the cDNA was eluted from the beads by heating for 5 min at 95 °C. Fractional recoveries from the affinity selection step were assessed by qPCR using SYBR Green I on a QuantStudio 5 thermal cycler (Thermo). After four rounds of selection, another two rounds of off-rate selections were performed by raising the wash stringency before elution to identify high-affinity binders. The final enriched cDNA was sequenced using a MiSeq next-generation sequencer (Illumina).

### Binding enzyme-linked immunosorbent assay

Biotinylated PSMD2 was immobilized on streptavidin-coated plates (Nunc) by incubating 60 nM protein solutions for 0.5 h at room temperature. After washing the plate, 1 μl of in vitro-translated FLAG-tagged peptides were incubated with 50 μl of HBS-T in the plate for 1 h. After washing with HBS-T (300 μl, three times), the plate was incubated with anti-FLAG-horseradish peroxidase (HRP; 1:5,000 dilution of monoclonal anti-FLAG M2-HRP produced in mouse; A8592, Sigma) for 0.5 h. Color development was achieved by adding TMB substrate (Sera Care), and the reaction was stopped by adding an equal volume of TMB stop solution (Sera Care). Absorbances were recorded at OD_450_ using a Multiskan Ascent plate reader and Ascent Software version 2.6. Peptides were run in technical triplicate from a single in vitro translation.

### Peptide synthesis

Thioether-macrocyclic peptides were synthesized using standard FMOC solid-phase peptide synthesis. After the peptide assembly was complete, the N terminus was capped with chloroacetyl in solid phase. The peptide was then released from the resin by treatment with a trifluoroacetic acid (TFA) cocktail, followed by precipitation with diethyl ether. The obtained crude peptide was dissolved in DMSO, and triethylamine was added for intramolecular cyclization via formation of a thioether bond between the thiol of the cysteine and N-terminal chloroacetyl group. After completion of cyclization, the reaction was quenched with acetic acid. The cyclized peptide was purified using standard reverse-phase high-performance liquid chromatography (HPLC) methods and characterized by liquid chromatography–MS (LC–MS; see Supporting Note for details).

### Surface plasmon resonance

PSMD2 was biotinylated via an Avi tag on the N terminus and immobilized on an SA Series S SPR chip (Cytiva) for measurements on a Biacore S200 (Cytiva). All four flow cells were blocked with saturating amounts of PEG-biotin (Thermo) after immobilization to prevent nonspecific binding. Experiments were run in 25 mM HEPES (pH 7.5), 100 mM NaCl, 90 mM KCl, 2% glycerol, 0.005% Tween-20 and 1% DMSO at 10 °C. Ligands were diluted three times in either a six- or ninefold dilution series and injected at 50 µl min^−1^ using single-cycle kinetics with sufficient off rates to determine kinetic rate constants of dissociation. A five-point solvent correction was run before and after the samples to account for DMSO-based bulk shifts. Binding kinetics were fit using a 1:1 binding model in Biacore BiaEvaluation (Cytiva) with an added parameter to account for drift over very long off rates.

### AlphaScreen

Assays were performed in white ProxiPlate Plus 384-well plates (PerkinElmer) in 25 mM HEPES (pH 7.5), 100 mM NaCl, 100 mM KCl, 0.005% Tween-20, 2 mM MgCl_2_, 0.1% BSA and 5 mM ATP. The 26S proteasome (Boston Biochem) was incubated at 2.5 nM with tracer compound MC2_biotin_ at 5 nM and 0–10 µM assayed compound for 90 min at room temperature. Then, 20 µg ml^−1^ AlphaScreen streptavidin donor, anti-rabbit acceptor beads (PerkinElmer) and anti-PSMD1 (1 nM; ab140682, Abcam) were added in the semidark, and plates were incubated for an additional 90 min in the dark at room temperature before reading on an Envision plate reader and Envision Manager software (PerkinElmer). Samples were run in at least three technical replicates.

### Lysate affinity purification with biotinylated macrocycle for tandem mass tag–mass spectrometry

HEK293 cell lysates (50–80 million cells per ml) were prepared in ice-cold lysis buffer (50 mM HEPES (pH 8.0), 50 mM NaCl, 5 mM MgCl_2_, 10% glycerol, 0.1% NP-40, 0.5 mM TCEP and 5 mM ATP) with 0.1 mM phenylmethylsulfonyl fluoride and 1× Roche cOmplete protease inhibitor cocktail. Cell homogenates were prepared by Dounce homogenization followed by removal of cell debris by centrifugation (20,000*g*, 15 min, 4 °C) and quantification of the protein content by 660-nm Protein Assay Reagent (Pierce, Thermo Fisher). For each replicate/condition, 80 µl of streptavidin magnetic beads was washed, suspended in 200 µl of ice-cold lysis buffer and used to capture 2 nmol (10 µM) of biotinylated compound (l-MC1 or d-MC1) for 60 min at 4 °C. Beads were washed twice to remove the unbound compounds. The washed compound-bound beads were incubated with 1.5 ml of cell lysate (4 mg) and allowed to bind cellular proteins for 3 h at 4 °C. The protein-bound beads were then isolated and washed three times with 4× bed volume of lysis buffer and three times with buffer without NP-40 and glycerol. One replicate of the final washed beads was used for ECL western blotting analyses, and the other replicates were submitted for LC–tandem MS (LC–MS/MS) analyses. Samples for TMT analyses were prepared similarly with 100 µl of NeutrAvidin agarose beads and 2.4 mg of cell lysate.

### On-bead digestion and tandem mass tag sample preparation

On-bead trypsin digestion was performed manually with the following protocol. Beads were washed twice with 25 mM ammonium bicarbonate. Protein samples were reduced with dithiothreitol (DTT; 10 mM, 1 h, 60 °C) and alkylated in the dark with iodoacetamide (15 mM, 30 min, 25 °C). Supernatant was removed, and beads/proteins were digested with 100 ng (10 μl × 10 ng μl^−1^) of trypsin (Promega) at 37 °C for 18 h. An additional 100 ng of trypsin was added and incubated at 37 °C for 4 h. Digestion was quenched with formic acid, and supernatants were desalted via solid-phase extraction (SPE) with Waters µHLB (Waters). Peptides were taken to dryness using a lyophilizer. TMT 10plex (Thermo Fisher Scientific) labeling of peptide was performed using the following protocol. Each TMT tag tube was mixed aggressively with 40 μl of acetonitrile and incubated for 15 min at room temperature. Each peptide sample was mixed aggressively with 15 μl of label and incubated in an Eppendorf Thermomixer for 1.5 h at 300 r.p.m. and 25 °C. Addition of 8 μl of fresh 5% hydroxylamine solution and 15-min incubation at room temperature was used to terminate each reaction. Each labeled sample was pooled, frozen, lyophilized and subjected to SPE on a high-density 3M Empore SDB-XC column (3M, 4340-HD). The eluent was lyophilized.

### Tandem mass tag peptide fractionation and mass spectrometry

A Pierce High pH Reversed-Phase Peptide Fractionation kit (84868) was used to fractionate peptides according to the manufacturer’s instructions. Briefly, columns were washed with acetonitrile and conditioned with 0.1% TFA. Peptides were loaded on column and washed with water (no acid) and eluted with 5, 7.5, 10, 12.5, 15, 17.5, 20 and 50% acetonitrile in 0.1% triethanolamine (TEA), and fractions were frozen, lyophilized and reconstituted in 0.1% TFA. Next, peptides (50% per fraction) were analyzed by nano-LC–MS/MS with a Waters NanoAcquity HPLC system interfaced to a Thermo Fisher Fusion Lumos mass spectrometer. Peptides were loaded on a trapping column and eluted over a 75-μm analytical column at 350 nl min^−1^; both columns were packed with Luna C18 resin (Phenomenex). A 90-min gradient was used per fraction (12 h total of LC–MS/MS time). The mass spectrometer was operated using a custom MS3 method. MS scans were acquired in the Orbitrap at 120,000-full-width at half-maximum (FWHM) resolution, MS2 scans were acquired in the ion trap using collision induced dissociation (CID) at 35% normalized collision energy (NCE), and product ions were isolated using synchronized precursor selection (SPS) and fragmented using higher-energy collision dissociation (HCD) at 55% NCE. The isolation window and number of ions for SPS-MS3 were adjusted based on the charge state of the precursor. MS3 scans were acquired in the Orbitrap at 50,000-FWHM resolution from *m*/*z* 100 to 200. A 2-s cycle time was used for all steps. Data were processed via in-house software and searched with the MASCOT algorithm (Matrix Science) against the UniProt human taxonomy database with appropriate modification parameters. Searched data were filtered with a 1% peptide false-discovery-rate (FDR) and 2% protein FDR. TMT quantitative results were processed with in-house software Mojave, and results were compiled and visualized with custom Spotfire Dashboard (TIBCO). TMT data analysis and statistics were performed using the MSStats package for R, and final volcano plots were made using the ggplot2 package for R^43^.

### In-gel digestion and liquid chromatography–tandem mass spectrometry

Immunoprecipitated proteins were boiled/eluted with SDS sample buffer and reduced with DTT (10 mM, 37 °C, 1 h). Cysteines were alkylated with iodoacetamide (20 mM, 25 °C, 30 min) and separated on a denaturing SDS–PAGE gel. Bands of interest were excised, and each gel slice was subjected to in-gel digestion with trypsin (Promega) at a 1:50 enzyme:substrate ratio in 25 mM ammonium bicarbonate (pH 8.0) overnight at 37 °C. Peptides were extracted with a solution containing 50% acetonitrile and 1% formic acid. Extracted samples were dried under vacuum and reconstituted in 2% acetonitrile and 0.1% formic acid. Samples were injected via an autosampler onto a 75 µm × 100 mm column (BEH, 1.7 µm; Waters) at a flow rate of 1 µl min^−1^ using a NanoAcquity UPLC (Waters). A gradient from 98% solvent A (water + 0.1% formic acid) to 80% solvent B (acetonitrile + 0.08% formic acid) was applied over 40 min. Samples were analyzed on line via nanospray ionization into a hybrid Thermo Fisher Orbitrap Fusion mass spectrometer. Data were acquired under data-dependent mode with 1-s duty cycle acquisition with ion-trap higher-energy collision dissociation (IT-HCD) fragmentation; the parent ion resolution was 120,000 FWHM, and the IT-HCD fragmentation resolution was 15,000 FWHM. Data were processed via in-house software and searched with the MASCOT algorithm (Matrix Science) against the UniProt human taxonomy database with appropriate modification parameters. Searched data were filtered with a 1% peptide FDR and 2% protein FDR. Results were compiled and visualized with custom Spotfire Dashboard (TIBCO). The experiment was performed on a single sample.

### Differential scanning fluorimetry

PSMD2 (5 µM (5 mg ml^−1^)) was incubated with either DMSO, 5, 10, or 20 µM macrocycle or peptide in buffer (25 mM HEPES (pH 7.5), 100 mM NaCl and 0.5 mM TCEP). Melting curves were conducted on a Prometheus NT48 (NanoTemper Technologies) by measuring the tryptophan fluorescence 330-nm/350-nm ratio of protein in a standard capillary. Standard deviations were calculated from three technical replicates performed with the same protein sample, and mean values were plotted using GraphPad Prism.

### Nuclear magnetic resonance experiments and assignment

NMR samples of unlabeled macrocyclic peptide MC1 were prepared in acetonitrile-d_3_/water (30/70) and DMSO-d_6_, with 4,4-dimethyl-4-silapentane-1-sulfonic acid as an internal reference. The concentration of peptide was 1–2 mM. NMR spectra were acquired at 298 K on a Bruker Avance 600-MHz spectrometer equipped with a 5-mm triple-resonance cryogenic probe. Chemical shift assignments were performed using 2D ^1^H-^1^H TOCSY and NOESY spectra with 70- and 500-ms mixing times, respectively, and DQF-COSY spectra. All spectra were processed using TOPSPIN (Bruker). NMR spectral analyses and assignments were performed using Mnova (Mestrelab Research) and NMRFAM-SPARKY software^44^.

### Library construction and selection for PSMD2 Fabs

The minimalist Fab library was constructed as described previously, which is the library D made by Fellouse et al.^45^ and contains greater than 10^10^ unique members. The library was sorted against biotinylated PSMD2 in complex with macrocycle MC1 in solution format following the standard phage display library screening protocol^41^. After five rounds of selection, phage was produced from individual clones grown in a 96-well format, and the culture supernatants were used in phage ELISA to detect specific binding. Clones that bound to antigen in phage ELISAs were subjected to DNA sequence analysis, and unique clones were aligned^46^.

### Fab chaperone screening and purification

Twenty clones were selected, and a stop codon was inserted between the Fab heavy chain and pIII proteins for each clone. The resulting phagemids were transformed into *Escherichia coli* strain 34B8. For screening, cells were grown in 500 ml of Complete C.R.A.P. at 30 °C for 24 h. Cell pellets were lysed with 5× BPER by weight, clarified by centrifugation at 18,500*g* for 60 min and filtered through a 0.2-µm PES membrane (Millipore Sigma). The supernatants were aliquoted into multiwell plates to allow for Fab affinity pulldown to be performed on a Hamilton Microlab STAR liquid handler using Phynexus tips packed with MabSelect SuRe resin (Cytiva). The resin-packed tips were first washed in buffer A (0.1 M glycine-HCl (pH 3.0) and 150 mM NaCl) and equilibrated in PBS. The protein was loaded onto tips by sequential binding from four plates containing clarified lysates. The resin-packed tips were then washed in 30 CV of PBS and blotted on blotting paper to remove excess wash buffer. The Fabs were eluted into 2.2 ml of buffer A and immediately neutralized with 0.2 ml of 1 M Tris (pH 8.0). The automated steps above involve pipetting up and down through the resin using flow rates of 8 µl s^−1^ for equilibration and wash steps and 4 µl s^−1^ for the binding and elution steps, while incorporating a 30-s pause after each aspirating and dispensing step. The quantity of purified Fabs was assessed by measuring the absorbance at 280 nm on a NanoDrop spectrophotometer (Thermo Fisher Scientific).

After screening, Fabs 8 and 14 were expressed at a 2-liter scale as described above. Cell pellets were reconstituted in PBS supplemented with an additional 15 mM NaCl and complete protease inhibitors (Roche) and clarified by centrifugation at 12,000*g* for 60 min. Lysates were then purified by passing over Protein G Sepharose 4 fast flow resin (Cytiva) and eluted with 10 mM glycine (pH 1.5), followed by neutralization with 1 M Tris (pH 8). Elutions were buffer exchanged by concentration in a 10,000-MWCO Amicon concentrator (Millipore Sigma).

### Cryo-electron microscopy sample preparation

PSMD2 (15 µM) and MC1 (100 µM) were mixed together in 25 mM HEPES and 200 mM NaCl and allowed to bind for 10 min before adding 45 µM each of Fab 14 and Fab 8. The complex was treated with 0.0125% glutaraldehyde for 20 min at 25 °C before quenching with Tris (pH 7.5) to a final concentration of 300 mM. The treated complex was injected on a Superdex 200 Increase 3.2/300 column on an AKTAmicro (Cytiva). Concentration of the peak fraction was determined by absorbance at 280 nm.

### Cryo-electron microscopy grid preparation and data collection

To prepare samples for cryo-EM, peak fractions from size exclusion were diluted to ~0.1 mg ml^–1^ in size exclusion buffer, and 4 μl of sample dilution was applied to holey gold grids (UltrAuFoil 25 nm R 1.2/1.3; Quantifoil) that had been glow discharged for 20 s using a Solarus plasma cleaner (Gatan). Grids were then plunge-frozen using a Vitrobot (Thermo Fisher Scientific) at 4 °C, 100% relative humidity, a blot force of 7 and a 4-s blot time.

Initial transmission electron microscopy data collection was performed using a Glacios (Thermo Fisher Scientific) operating at 200 kV and a K2 summit direct electron detector. Movies were collected in counting mode at a nominal magnification of ×36,000 (1.148 Å per pixel) and a defocus range of –1 to –3 μm, without stage tilt. For the final datasets, a Titan Krios G3i (Thermo Fisher Scientific) operating at 300 kV equipped with a BioQuantum energy filter was used. Movies were collected with a K3 Summit direct electron detector operating in super-resolution mode at a nominal magnification of ×105,000 (0.419 Å per pixel), an energy slit width of 20 eV, defocus range of –1 to –3 μm, 3-s exposure time, 60 frames per movie, a total electron fluence of ~64 e^–^ Å^–2^ and a stage tilt of 20°.

For all data collection, microscope and camera automation was accomplished by using SerialEM^47^ version 3.7.14.

### Cryo-electron microscopy data processing

Cryo-EM data were processed using RELION^48,49^ version 3.1.1 and cisTEM^50^ version 2.0.0-alpha.

The PSMD2/Fab/MC1 data were processed as described in Supplementary Fig. 10. An initial Glacios dataset collected at 0° stage tilt was processed using RELION. Ab initio particle picking, 2D classification with selection, three-dimensional (3D) classification with selection and 3D autorefine with Contrast Transfer Function (CTF) refinement and Bayesian particle polishing resulting in an initial map at ~4.1-Å resolution were used. Due to a preferred orientation, the final PSMD2/Fab/MC1 dataset was collected at a 20° tilt and processed using both RELION and cisTEM. Movies (3,281 in total) were motion corrected using RELION, and CTF estimation was performed by CTFFIND4 (ref. 51). The initial 4.1-Å map was used as a template for autopicking, resulting in 1,311,869 particle coordinates. These particles were extracted and classified/selected in 2D and 3D, ultimately resulting in a final selection of 105,705 particles. These particles were then subjected to RELION 3D autorefinement, per particle defocus refinement and Bayesian particle polishing. The resulting particles were then imported into cisTEM for the final refinement using a mask that encompassed the entire macromolecule, with the regions outside the mask multiplied by 0. Focused refinement using a mask including only PSMD2/MC1 and excluding the Fabs did not improve resolution (not shown). At a Fourier shell correlation (FSC) of 0.143, the masked full-molecule refinement reached a nominal resolution of 2.5 Å, with substantial variation in local resolution, as measured by RELION postprocessing.

The PSMD2/Fab/MC1 data were processed as described in Supplementary Fig. 11. The 3,619 movies from this dataset were motion corrected with RELION, and CTFFIND4 was used for CTF estimation. The initial PSMD2/Fab/MC1 map was used as a template for autopicking, resulting in 737,190 particle picks. These were extracted and classified/selected in 2D and 3D, and the final selection consisted of 82,738 particles. As before, these were further processed with RELION 3D autorefinement with per particle defocus refinement and Bayesian particle polishing before exporting to cisTEM for focused refinement using a mask that included only PSMD2 and excluded the Fabs. For this focused refinement, the region outside the mask was not downweighted but was low-pass filtered to 12 Å. At an FSC of 0.143, this map reached a nominal resolution of 3.4 Å.

Before modeling, the PSMD2/Fab/MC1 map was density modified using Phenix Resolve CryoEM^52^. The PSMD2/Fab/MC1 atomic models were built using Coot^53^ using PDB 6MSJ as a starting atomic coordinate into the PSMD2/Fab/MC1 map; the FSCref = 0.5 resolutions before and after density modification were 3.01 Å and 2.78 Å, respectively. Three cycles of Phenix refinement were used for model refinement^54^. Final data processing statistics are reported in Supplementary Table 2. Buried surface area was calculated using PISA^55^.

### Recombinant pulldowns

To assess ligand binding to the 26S proteasome,10 µl of washed Streptavidin T1 beads (Invitrogen) was coated with 20 µM biotinylated peptide in 25 mM HEPES-NaOH (pH 7.5), 50 mM NaCl, 0.5 mM TCEP, 5% glycerol, 5 mM MgCl_2_, 3 mM ATP and 0.005% Tween-20. Beads were washed with 3 CV of buffer three times before incubating with 200 nM 26S proteasome (Boston Biochem) complexed with either buffer or 20 µM MC1–MC3 or PP1 for competition. Beads and sample were incubated at room temperature for 30 min before placing the sample on a magnet, draining the sample and washing three times with 5 CV of ice-cold buffer before eluting by boiling into 4× Novex LDS sample buffer (Invitrogen) supplemented with 100 mM DTT for 5 min. Samples were run on a 4–12% Bis-Tris Novex gel with MOPS running buffer (Invitrogen), transferred to a nitrocellulose blot using the iBlot 2 (Invitrogen) and probed with antibodies to PSMD4 (1:200 dilution; Cell Signaling Technology, D17E4, 3336) and PSMA1 (1:4,000 dilution; BML-PW1800-0100, Enzo). Blots were imaged using LICOR 1:1,000 dilution secondary anti-rabbit 680 and 1:1,000 anti-mouse 800 on an Odyssey imager (LICOR). A 1:5,000 dilution of anti-Streptavidin HRP was used to detect loading amounts in the blot and was developed using West Femto ECL reagent (Thermo) and imaged on a PXi imager (Syngene). The experiment was run at least two separate times.

To assess competition between ligands and USP14 binding to PSMD2, 10 µl of washed Streptavidin T1 beads (Invitrogen) was coated with 20 µM biotinylated peptide in 25 mM HEPES-NaOH (pH 7.5), 50 mM NaCl, 0.5 mM TCEP, 5% glycerol, 5 mM MgCl_2_ and 0.005% Tween-20. Beads were washed with 3 CV of buffer three times before incubating with 200 nM PSMD2 complexed with either buffer or 20 µM USP14, Rad23_UBL_ or MC1–MC4. Beads and sample were incubated at room temperature for 30 min before placing the sample on a magnet, draining the sample and washing three times with 5 CV of ice-cold buffer before eluting by boiling into 4× Novex LDS sample buffer (Invitrogen) supplemented with 100 mM DTT for 5 min. Samples were run on a 4–12% Bis-Tris Novex gel with MOPS running buffer (Invitrogen) and stained overnight with Sypro Ruby (Invitrogen) before imaging on a Typhoon Trio (GE, Cytiva) with 488 excitation and a 526 SP emission filter. The experiment was run at least two separate times.

To assess ternary complex formation between PSMD2, BetCIDE 1–BetCIDE 16 and BRD4 domains BD1 or BD2, 2 µM biotinylated BD1 or BD2 was incubated with 10 µl of washed streptavidin M-280 Dynabeads (Invitrogen) for 15 min at room temperature in HEPES (pH 7.5), 150 mM NaCl, 5% glycerol, 0.5 mM TCEP, 0.05% Tween-20 and 0.5% BSA before loading onto a plate for pulldown using a King Fisher Duo Prime (Thermo). Biotinylated beads were washed with 200 µl of buffer and incubated in 200 µl of 2 µM PSMD2 complexed with 2 µM compound for 30 min, followed by three 200-µl washes for 90 s each. Beads were eluted into 4× LDS Novex sample loading buffer (Invitrogen) before running on a 3–12% Bis-Tris Novex SDS–PAGE gel (Invitrogen) and staining with Sypro Ruby (Thermo). Gels were imaged with a Typhoon Trio (GE, Cytiva) with 488-nm excitation and a 526 SP emission filter. The experiment was run at least two separate times.

To interrogate l-CIDE-induced ternary complexes in lysates, exponentially growing HEK293 cells were trypsinized, washed with ice-cold PBS and lysed in FLAG immunoprecipitation buffer (50 mM Tris-HCl (pH 7.4), 150 mM NaCl, 1 mM EDTA and 1% Triton X-100 supplemented with 1× Halt protease inhibitor cocktail (Thermo), 5 µM bortezomib (Selleck) and 0.5 mM ATP-γ-S (Sigma)) at a density of 2 × 10^7^ cells per ml. Lysates were vortexed, centrifuged at 9,300*g* at 4 °C and sonicated twice using an Active Motif EpiShear probe sonicator (30% amplitude, 10 s on and 2 s off for 20 s, spinning between sonication rounds). Sonicated lysates were centrifuged at 9,300*g* at 4 °C, and the supernatant was collected as the final lysate. Lysates were precleared with Dynabeads M-280 Streptavidin (Thermo; 30 µl per sample washed in FLAG immunoprecipitation buffer supplemented as described above) at room temperature for 20 min with rotation. Two milligrams of precleared lysate were then supplemented with or without 150 nM recombinant biotinylated BRD4_BD1_, and 2% was retained as input. Lysates were next treated with DMSO control or 50 nM l-CIDE and incubated at room temperature for 20 min with rotation. Pulldowns were performed using Dynabeads M-280 Streptavidin (Thermo; 50 µl per sample washed in FLAG immunoprecipitation buffer supplemented as described above) at 4 °C overnight and washed three times with supplemented FLAG immunoprecipitation buffer. Elution was performed using 2× NuPAGE LDS sample buffer (Invitrogen) supplemented with 1× sample reducing agent (Invitrogen), followed by boiling and analysis using standard western blotting procedures as described below. Data are representative of three independent experiments.

### Hydroxyl radical footprinting labeling procedure with fast photochemical oxidation of protein

PSMD2 (5 µM) was complexed with either buffer or 10 µM MC1 or USP14 and dialyzed overnight in PBS at 4 °C to ensure removal of any potential radical-scavenging components. PSMD2 apo, MC1-bound and USP14-bound complexes were subjected to laser-induced HRF as previously described in detail^56,57^. This work uses our previously described dosimetry strategy in which leucine enkephalin was spiked into each sample to monitor the scavenging potential during sample irradiation^58^.

### Liquid chromatography–tandem mass spectrometry analysis of hydroxyl radical footprinting samples

LC–MS/MS analysis was performed as previously published^58^. In brief, samples for LC–MS/MS analysis were denatured with guanidine, reduced with DTT and *s*-carboxymethylated with isodacitate. Samples were then desalted using NAP-5 columns and subjected to trypsinization followed by deglycosylation using PNGaseF. After quenching with formic acid, the tryptic peptides (10 µg) were separated using a Waters H-Class UPLC with a Waters Acquity UPLC CSH130 C18 column (1.7 µm, 2.1 × 150 mm), with a column temperature of 77 °C. Peptide separation occurred across a gradient from 100% solvent A (water and 0.1% formic acid) to 35% solvent B (acetonitrile and 0.1% formic acid) over 60 min at a flow rate of 0.3 ml min^−1^. MS analysis was performed with a Thermo Fisher Q Exactive operating in positive mode, performing MS2 on the top ten most abundant peaks in data-dependent mode.

### Data analysis of hydroxyl radical footprinting samples

Peak identification and quantitation of percent oxidation for each peptide were performed using the Biologic Software Suite (Protein Metric). All samples were analyzed in triplicate after background subtraction from a corresponding ‘no laser’ control sample. The extracted ion chromatograms for the oxidized peptide species and the parent peptide (in bound and unbound states) were used to calculate the percent oxidation with the equation as previously described^55,57,58^. Percent oxidation is presented as average of triplicate runs after subtraction of the ‘no laser’ background oxidation control. Error bars represent a 95% confidence interval for the background-adjusted mean percent oxidation for each peptide, based on statistical analysis of the three values by a single-sample *t*-test. For each peptide, the percent oxidation level of a sample was classified heuristically as different from the level of another sample if the confidence intervals for the two means do not overlap. Non-overlapping error bars were considered notable differences^56,58,59^.

### Fluorescence microscopy and internalization experiments

KPL-4 cells from the Genentech cell repository (short tandem repeat validated) were grown in RPMI supplemented with 10% fetal bovine serum (FBS) and 1% glycine. MC1_Cy5.5_ was dissolved in DMSO at 200 µM and used at 2 µM in continuous uptake experiments. Capped free Cy5.5 (custom capped Cy5.5 azide A4030; Lumiprobe Batch 7888; Supporting Information) was dissolved in DMSO at 500 µM and used at 2 µM.

Endocytic inhibitors included Dynasore (synthesized in-house, dissolved at 80 mM in DMSO and used at 80 µM in serum-free medium), Filipin (Sigma, F9765; 5 mg ml^−1^ stock in methanol; used at 5 µg ml^−1^) and dimethyl amiloride (Sigma, A5642; 9 mg ml^−1^ stock in methanol; used at 200 µg ml^−1^).

#### Internalization experiments

KPL-4 cells were grown on LabTek-II eight-well slides to approximately 70% confluency and incubated with 2 µM MC1_Cy5.5_ and the micropinocytic probe Oregon Green Dextran (Molecular Probes, D7171; 200 µg ml^−1^) and/or the clathrin-dependent recycling marker Alexa 555–transferrin (12.5 µg ml^–1^; Molecular Probes, T35352) for different lengths of time. Cells were then washed four times in cold medium, fixed for 20 min in 3% paraformaldehyde, washed twice for 5 min each in PBS and mounted in Prolong Gold plus DAPI (Invitrogen, P36931). Mitochondria were stained with 200 nM Mitotracker Red FM (Molecular Probes, M22425) for at least 20 min before live imaging.

Spinning disk confocal microscopy was performed using a CSU-W1 (Yokogawa) spinning disk on a Zeiss AxioObserver M1 microscope with a ×63/1.4-NA objective equipped with 405-, 488-, 561- and 640-nm lasers. Images were acquired using SlideBook v6 (Intelligent Imaging Innovations) and a Photometrics Prime BSI (Teledyne Photometrics). Figures were assembled in Adobe Photoshop 2021, and any gamma adjustments to the contrast were applied across the whole image.

### NanoBRET

The NanoBRET assay is a tracer competition BRET assay that can detect binding of a fluorescent tracer that binds to BRD4. The assay without digitonin is a slightly modified version of the NanoBRET target engagement BET BRD assay (Promega, N2131) described in the manufacturer’s protocol. Deviations from the manufacturer’s 384-well protocol include use of an HEK293 BRD4–NanoLuc stable cell line instead of transient transfection of DNA provided in the kit. Additionally, compounds and tracer solubilized in DMSO were dispensed into the plate using an Echo 555 liquid handler (Labcyte, Beckman) for an assay condition of 1.5% DMSO from compound and tracer (0.25 μM) in 6,800 cells per well (40-μl well volume) for a 2-h incubation. For the digitonin version of the NanoBRET assay, all conditions were the same as those for the without digitonin assay with the following exceptions: digitonin was added to the cells right before dispensing into the plate for a digitonin concentration of 50 μg ml^−1^ in the 40-μl well. Compound incubation time was 30 min, and the Extracellular NanoLuc Inhibitor was excluded from this assay. Raw data were normalized and scaled to a DMSO control column on a per row basis to account for decreases of substrate during plate read time; data were then background corrected with a ‘no tracer’ condition before fitting normalized BRET (%) compound dose–response with software such as GraphPad Prism. The IC_50_ was then converted to an apparent compound *K*_i_ using the Cheng–Prusoff correction by dividing the IC_50_ by a constant specific to each digitonin condition that was based on tracer titrations for each experiment^28^. Samples were run in replicates as described in the figure legends.

### Quantitation of BRD4 degradation by CIDEs in HEK293 cells

HEK293 cells were seeded on day 1 at a density of 22,500 cells per well in CellCarrier-384 Ultra microplates and tissue culture treated (PerkinElmer, 6057300) in 45 μl per well of assay medium (RPMI, 10% FBS and 1% GlutaMAX). On day 2, compounds were serially diluted 1:2 in DMSO to create 20-point dilutions across a 384-well v-bottom polypropylene microplate (Greiner, 781091). Two microliters of each well of compound serial dilution was transferred to 98 μl of assay medium for an intermediate dilution. Five microliters of compound intermediate dilution was then transferred and mixed in 45 μl in the cell plate. Columns 1, 2, 23 and 24 were treated with DMSO only (0.2% final) for data normalization as ‘neutral controls’. After compound treatment, cell plates were incubated at 37 °C for 24 h. Cells were then fixed in 3.7% final concentration of paraformaldehyde by addition of 15 μl of 16% paraformaldehyde (Electron Microscopy Sciences, 15710-S) directly to the 50 μl of medium and compound in the cell plate. Cell plates were incubated at room temperature for 20 min. Well contents were aspirated and washed with 100 μl per well of PBS three times. PBS (50 μl per well) containing 0.5% (wt/vol) BSA and 0.5% (wt/vol) Triton X-100 (antibody dilution buffer) was added to each well. Samples were incubated for 20 min. Well contents were aspirated and washed three times with 100 μl per well of PBS. Immunofluorescence staining of BRD4 was performed by diluting monoclonal anti-BRD4 (EPR5150; Abcam, 128874) 1:500 in antibody dilution buffer (PBS, 0.5% Triton X-100 and 0.5% BSA). BRD4 antibody (25 μl) diluted in buffer was added and incubated overnight at 4 °C.

On day 3, samples were washed three times with 100 μl per well of PBS. Secondary antibody solution (25 μl per well; goat anti-rabbit IgG, DyLight 488-conjugated highly cross-adsorbed (Thermo Fisher, 35553) and Hoechst 33342 (1 μg ml^−1^) diluted in antibody dilution buffer) was dispensed into each well. Hoechst 33342 and secondary antibody only were added to the bottom three columns for data normalization as ‘inhibitor controls’. Samples were incubated for 2 h at room temperature and washed three times with 100 μl of PBS. Quantitative fluorescence imaging of BRD4 was performed using an Opera Phenix High-Content Screening System. Fluorescent images of the samples were captured using 488-nm and 405-nm channels. The Hoechst channel was used to identify nuclear regions. Mean 488 intensity of BRD4 was quantitated in nuclear regions. Data analysis was performed using Genedata Screener, with DMSO- and no primary antibody control-treated samples used to define the 0% and 100% changes in BRD4. Dose–response log (inhibitor) versus response was used to define the inflexion point of the curve (half-maximum effective concentration (EC_50_)) and the plateau of the maximal effect. Samples were run with two technical replicates.

### Cell culture, cell treatments and western blotting for BRD4 degradation

HEK293 cells were obtained from Genentech’s repository and were cultured under standard conditions in RPMI medium containing 10% FBS. For evaluating the cellular effects of the degrader compounds, 0.4 million cells were seeded in 1 well of a 12-well plate. The following day, the medium was diluted twofold with fresh medium containing vehicle (DMSO) or compounds. For degradation rescue studies, cells were co-treated with the proteasome inhibitor bortezomib (62.5 nM) or BET ligand (0.1, 1 or 10 µM). Cells were cultured with the compounds for an additional 20 h and collected. Complete cell lysates were prepared in urea lysis buffer (50 mM Tris-HCl (pH 7.4), 120 mM NaCl, 1 mM EDTA, 1 % NP-40, 6 M urea and 1× Roche cOmplete protease inhibitor cocktail). Ten micrograms of total lysate was resolved by NuPAGE 3–8% Tris-acetate gels (Thermo Fisher) and transferred to nitrocellulose membranes (Bio-Rad Trans-Blot transfer system) followed by western blotting analysis. Primary antibodies were diluted 1:3,000 and incubated for 1 h at room temperature or 4 °C overnight. Secondary antibodies were diluted 1:10,000 and incubated for 60 min at room temperature. Blots were imaged by scanning using LICOR Odyssey CLx.

### CRISPR–Cas9-mediated deletion of *KLHL15* in HEK293 cells

*KLHL15* was deleted in HEK293 cells using a CRISPR–Cas9 approach with two single guide RNAs (sgRNAs; Synthego) within exon 1. An mScarlet-expressing single-stranded donor DNA (Genscript) containing homology arms (sgRNA and donor sequences provided in the Supplementary Information) was used to replace exon 1 by homology-directed repair (HDR) and enable enrichment of the knockout population by fluorescence-activated cell sorting (FACS; schematic shown in Extended Data Fig. 10). Briefly, two sgRNAs (sg1 and sg3 or sg2 and sg3) were mixed with purified TrueCut Cas9 V2 protein (Thermo Fisher Scientific, A36498) at a 3:1 molar ratio and incubated for 20 min to generate complexes. The Cas9–sgRNA mix was nucleofected into cells along with the donor DNA using a Lonza 4D nucleofector system with a predetermined protocol for HEK293 cells. A non-targeting control guide was included in parallel. After brief expansion, mScarlet^+^ cells were isolated as pools by FACS. Overall, mScarlet expression and percentage of mScarlet^+^ cells was low likely due to low HDR efficiency, and a relaxed gating strategy was used to isolate positive cells. For the non-targeting guide cells, mScarlet^–^ cells (as defined by parental HEK293 cells) were also isolated (as a control cell line). The mScarlet^+^ pools, labeled A1 (guides sg1 and sg3) and C1 (guides sg2 and sg3), were expanded, and single-cell clones were generated by infinite dilution. Genomic DNA was isolated using the Zymo Research Quick-DNA kit (D3024), and clones were analyzed by genomic PCR (specific PCR oligonucleotides are provided in the Supplementary Information) using the NEB Q5 high-fidelity polymerase (M0492). Two clone profiles were further analyzed: (1) clones that showed a complete loss of the wild-type band and the presence of an mScarlet knock-in band and (2) clones that showed complete loss of the wild-type band. The clones that showed no mScarlet knock-in were likely enriched in the initial FACS analysis due to residual mScarlet expression from the unintegrated donor, which contains an *Eif1a* promoter driving mScarlet expression. The genotype of the clones was further confirmed by ddPCR using the Bio-Rad QX200 AutoDG ddPCR system with specific primers (Integrated DNA Technologies) and FAM- or HEX-conjugated locked nucleic acid-modified probes (Integrated DNA Technologies; sequences are provided in the Supplementary Information). Probes included (1) cut site overlapping probes (FAM), (2) HDR knock-in probe (FAM), (3) control HEX probe that binds both wild-type and edited genomic DNA and (4) FAM- or HEX-labeled PlekHF1 centromeric copy number reference assays (Bio-Rad). Each reaction mix contained 40 ng of genomic DNA, 900 nM primers, 250 nM target probe, 1.25 µl of 20× reference probe and ddPCR Supermix for Probes (no dUTP; Bio-Rad, 186-3024). Copy number was determined using the QuantaSoft Analysis Pro Software (Bio-Rad). Clones that were confirmed by ddPCR were further analyzed using real-time qPCR. RNA from clones was extracted using the Qiagen RNeasy Plus Mini kit (74136), and 2 µg of RNA was reverse transcribed using a Thermo Fisher high-capacity cDNA reverse transcription kit (4368813). qPCR was performed using 20 ng of cDNA per well on the QuantStudio 7 Flex real-time PCR system (Thermo Fisher) with TaqMan probes for *KLHL15* (Hs01072769_m1, Thermo Fisher) and *RPL13A* (housekeeping gene; Hs04194366_g1, Thermo Fisher). Final cell lines that were used in these studies included clone A1-14 (*KLHL15*-KO 1), which has a long deletion generated in all three *KLHL15* alleles, clone C1-15 (*KLHL15*-KO 2), which has a long deletion generated in all three *KLHL15* alleles and mScarlet integrated into one *KLHL15* allele, and pool E1 (NT-gRNA), which has unaltered *KLHL15* alleles generated from the non-targeting gRNA.

### Reporting summary

Further information on research design is available in the Nature Portfolio Reporting Summary linked to this article.

## Online content

Any methods, additional references, Nature Portfolio reporting summaries, source data, extended data, supplementary information, acknowledgements, peer review information; details of author contributions and competing interests; and statements of data and code availability are available at 10.1038/s41589-022-01218-w.

## Supplementary information

Supplementary Information Supplementary Figs. 1–13, Tables 1–11, Figs. 14–17 (unprocessed gels or blots) and note (synthesis of MC1_Cy5.5_ and CIDEs).

Reporting Summary

Supplementary Table 1 Table of oligonucleotides and reagents for *KLHL15*-knockout experiments.

## Data Availability

The PSMD2/Fab/MC1 and PSMD2/Fab maps have been submitted to the EMD (https://www.ebi.ac.uk/pdbe/emdb/) under accession numbers 24742 and 24743, respectively. The PSMD2/MC1 and PSMD2 apo models have been deposited to the PDB (rcsb.org) under accession numbers 7UJD and 7UJH. Source data are provided with this paper. All other data generated or analyzed during this study are included in this published article and its Supplementary Information files.

## Source data

Source Data Fig. 4 Unprocessed western blots and/or gels.
Source Data Fig. 6 Unprocessed western blots and/or gels.
Source Data Extended Data Fig. 2 Unprocessed western blots and/or gels.
Source Data Extended Data Fig. 3 Unprocessed western blots and/or gels.
Source Data Extended Data Fig. 10 Unprocessed western blots and/or gels.
