# Supplementary material for: Targeted degradation via direct 26S proteasome recruitment
Source: Nat Chem Biol. 2022 Dec 28;19(1):55–63. doi: 10.1038/s41589-022-01218-w (PMC9797404; doi:10.1038/s41589-022-01218-w)
Supplement: Supplementary file 1 — Supplementary Figs. 1–13, Tables 1–11, Figs. 14–17 (unprocessed gels or blots) and note (synthesis of MC1Cy5.5 and CIDEs). [file 41589_2022_1218_MOESM1_ESM.pdf]

nature chemical biology

Article

https://doi.org/10.1038/s41589-022-01218-w

Targeted degradation via direct 26S proteasome recruitment

In the format provided by the authors and unedited

Supplementary Information

Supplementary Figures 1-13
Supplementary Figures 14-17 (unprocessed gels and blots from Supplementary Figures 1-13)
Supplementary Tables 1-8
Supplementary Tables 9-11 (KLHL15 KO reagents)
Supplementary Note (synthesis of MC1-Cy5.5 and CIDEs)

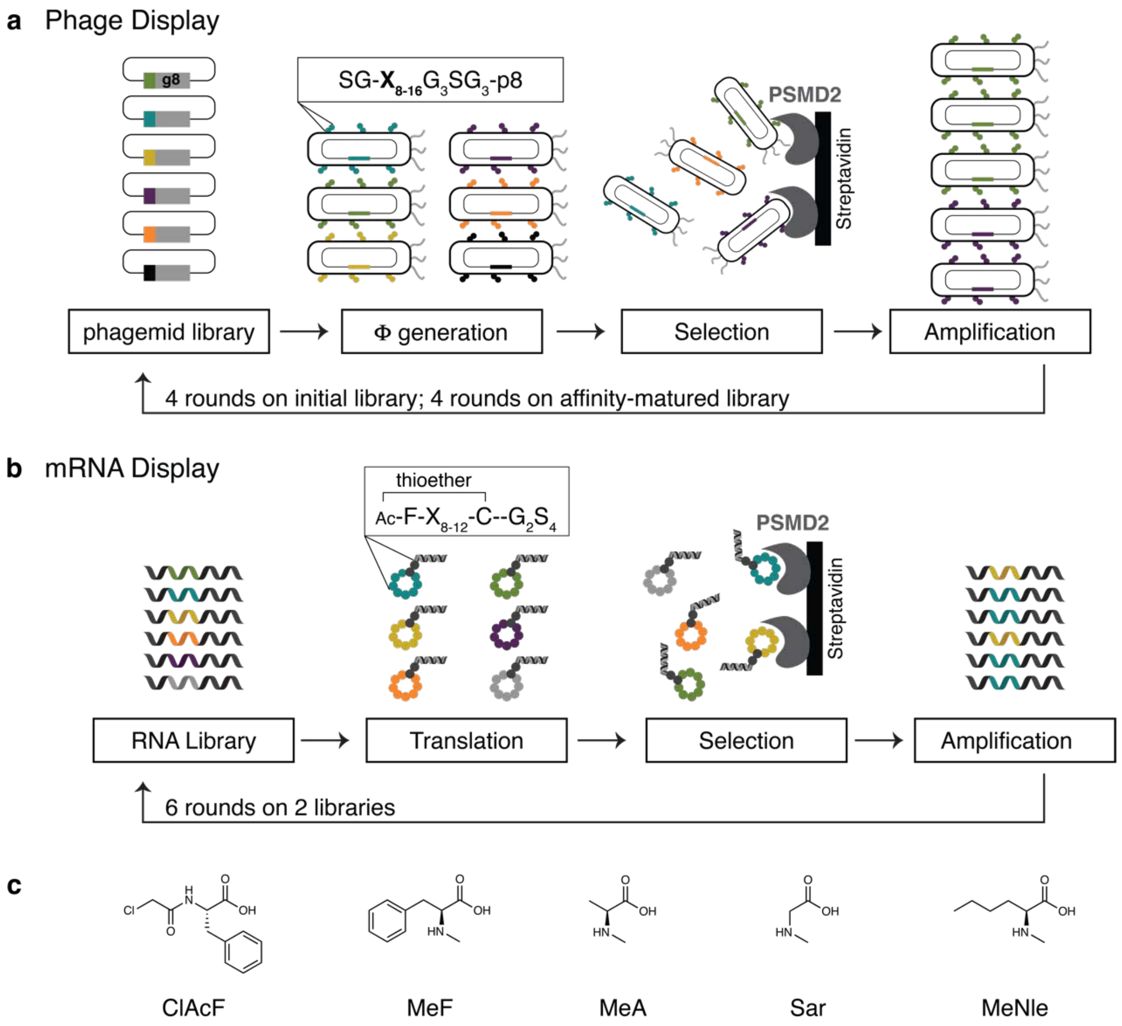

Supplementary Fig. 1: Ligands discovered from display efforts.
a, Representation of phage display selection. Libraries containing variable peptide sequences fused to gene 8 were selected against PSMD2 through iterative rounds of phage generation, selection, and amplification. Top candidates were then used to generate soft-randomized library for affinity maturation, resulting in the best linear peptide hit, PP1. **b**, Representation of mRNA display selection. Macrocycle peptide libraries varying from 8-16 amino acids in length are encoded in an mRNA library, and sequences that bind to PSMD2 are enriched over iterative cycles of in-vitro translation/transcription, selection, and sequence amplification. **c**, Structures of 5 non-natural amino acids used in libraries, including the initiator ClAcF (chloroacetyl-phenylalanine), MeF (N-methyl-L-phenylalanine), Sar (Sarcosine or N-methyl glycine), MeAla (N-methyl-L-alanine), and MeNle (N-methyl-L-norleucine).

library	selection	name		sequence														% sequenced K1	K2
natural amino acid library	solution selection		ClAc-	F	Y	F	E	R	F	R	R	P	A	R	C			12.47%	21.50%
			ClAc-	F	V	L	K	S	F	R	R	P	P	R	C			5.62%	12.92%
			ClAc-	F	R	F	R	Y	L	R	Y	E	R	C				3.71%	4.42%
			ClAc-	F	I	F	Q	S	H	F	R	R	P	N	R	C		1.36%	2.24%
			ClAc-	F	F	L	K	H	F	Q	R	P	S	R	C			1.14%	1.97%
			ClAc-	F	Y	L	D	G	F	R	R	P	P	R	C			1.38%	1.02%
			ClAc-	F	A	L	R	Y	F	R	R	P	A	R	C			0.66%	1.02%
			ClAc-	F	L	L	D	G	F	R	R	P	P	R	C			0.59%	0.70%
	bead selection	**MC2**	**ClAc-**	**F**	**S**	**D**	**K**	**P**	**L**	**H**	**R**	**Y**	**V**	**G**	**F**	**Q**	**C**	51.46%	56.55%
			ClAc-	F	R	K	N	P	R	F	W	A	L	A	R	C		4.09%	9.39%
			ClAc-	F	L	T	S	Y	Y	Y	R	V	F	I	F	G	C	7.10%	7.27%
			ClAc-	F	H	S	A	F	R	Y	L	R	Y	E	S	F	C	2.85%	3.50%
		MC4	ClAc-	F	S	R	N	P	R	F	W	A	A	Y	C			1.12%	1.43%
			ClAc-	F	H	R	L	D	R	F	W	H	L	P	R	C		0.16%	0.20%
n-methylated amino acid library	solution selection		ClAc-	F	R	R	L	S	W	W	S	H	H	D	C			15.19%	59.57%
			ClAc-	F	R	R	L	S	W	S	W	S	C					4.91%	8.51%
			ClAc-	F	R	R	L	S	W	W	S	V	H	C				2.09%	5.16%
			ClAc-	F	R	R	L	S	W	R	W	S	C					0.69%	0.84%
			ClAc-	F	R	R	L	S	W	S	W	R	C					0.36%	0.59%
			ClAc-	F	R	Y	Nle	F	S	W	L	D	C					0.34%	0.17%
	bead selection	**MC3**	**ClAc-**	**F**	**S**	**N**	**W**	**P**	**S**	**W**	**L**	**H**	**Y**	**L**	**C**			21.79%	18.10%
			ClAc-	F	Y	S	S	Y	Y	W	D	V	Y	Y	Y	W	C	10.02%	13.47%
			ClAc-	F	V	Y	R	W	Y	G	L	P	P	L	H	R	C	4.51%	12.21%
			ClAc-	F	R	G	V	S	W	F	R	Y	L	C				8.96%	11.42%
			ClAc-	F	F	L	Y	A	H	R	S	W	R	V	L	C		1.43%	1.36%
		**MC1**	**ClAc-**	**F**	**P**	**D**	**V**	**G**	**L**	**H**	**R**	**Y**	**W**	**G**	**W**	**D**	**C**	0.17%	1.34%
			ClAc-	F	R	R	L	S	W	N	W	S	C					0.35%	1.17%
			ClAc-	F	R	P	F	R	Y	L	Y	Y	H	S	C			0.24%	0.98%
			ClAc-	F	Y	G	V	S	W	F	R	Y	L	C				1.80%	0.85%
			ClAc-	F	L	R	Y	L	Y	P	H	Y	W	N	C			0.49%	0.85%
			ClAc-	F	L	Y	F	R	Y	W	Y	V	G	W	C			0.93%	0.63%
			ClAc-	F	P	D	V	G	F	H	R	Y	W	G	W	D	C	0.45%	0.57%

Supplementary Fig. 2: Sequences from mRNA display.

Sequencing results from 6 rounds of selections. Two libraries, one with natural amino acids and one including 4 n-methylated amino acids were selected against PSMD2 in solution or on beads. Sequence enrichment after the final two rounds of most stringent washing (K1 and K2) are shown on the right. Top candidates are labeled in bold underlined text. Red text represents an n-methylated amino acid: MeF (N-methyl-L-phenylalanine), Sar (Sarcosine or N-methyl glycine), MeAla (N-methyl-L-alanine), and MeNle (N-methyl-L-norleucine). All sequences begin with ClAcF (chloroacetyl-phenylalanine) and end with C (cysteine) to enable cyclization.

26S pulldown

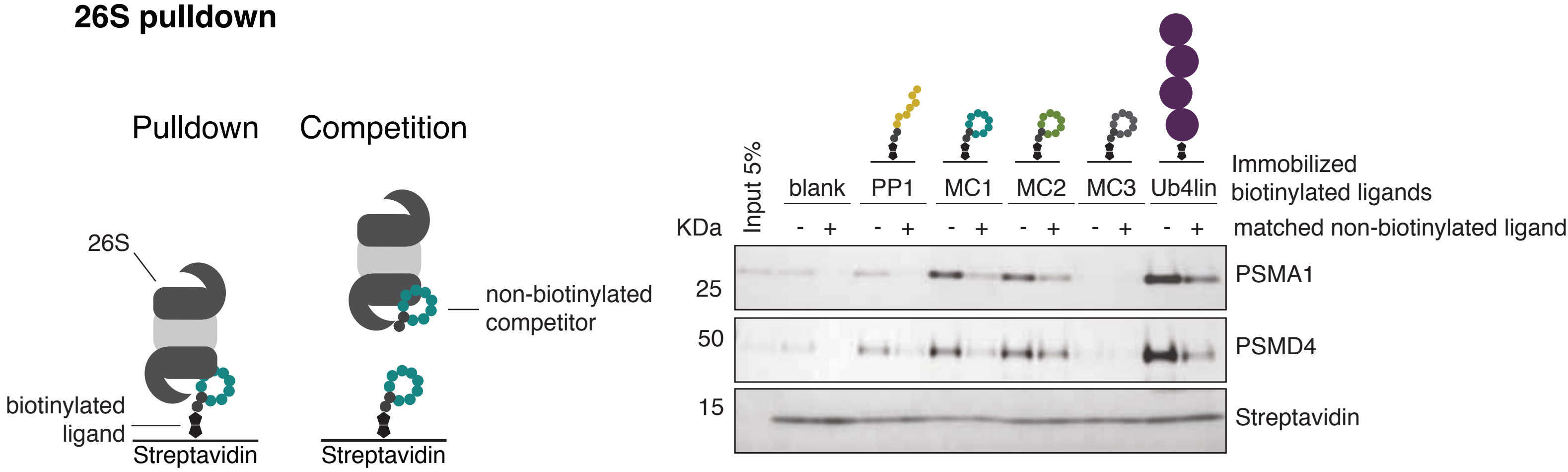

Supplementary Fig. 3: Biotinylated macrocycles can pulldown 26S proteasome. Biotinylated versions of MC1, MC2, MC3, and PP1 ($MC1_{biotin}$ $MC2_{biotin}$, $MC3_{biotin}$, $PP1_{biotin}$) were incubated with 26S proteasome *in vitro* in the presence or absence of excess corresponding non-biotinylated ligand. Linear ubiquitin was included as a positive control. Both PSMD4 (19S) and PSMA1 (20S) subunits were pulled down to some extent by 3 of the 4 ligands. Data were reproduced in 2 independent experiments.

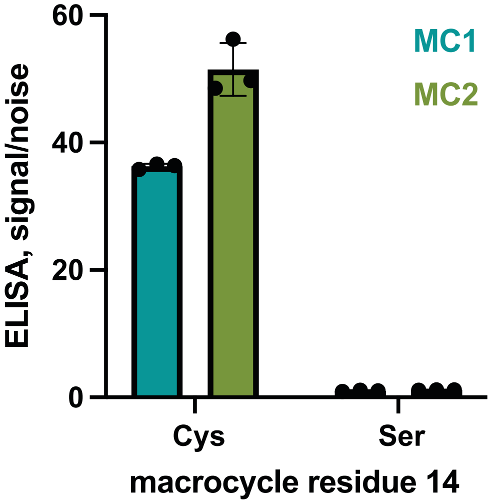

Supplementary Fig. 4: Cyclization is required for binding to PSMD2.
ELISA signal to noise for MC1 and MC2 containing the original cysteine residue or a serine mutation at position 14. Data are shown as the ratio of PSMD2 signal to Streptavidin signal alone. Bars represent the mean ± standard deviation of 3 independent experiments.

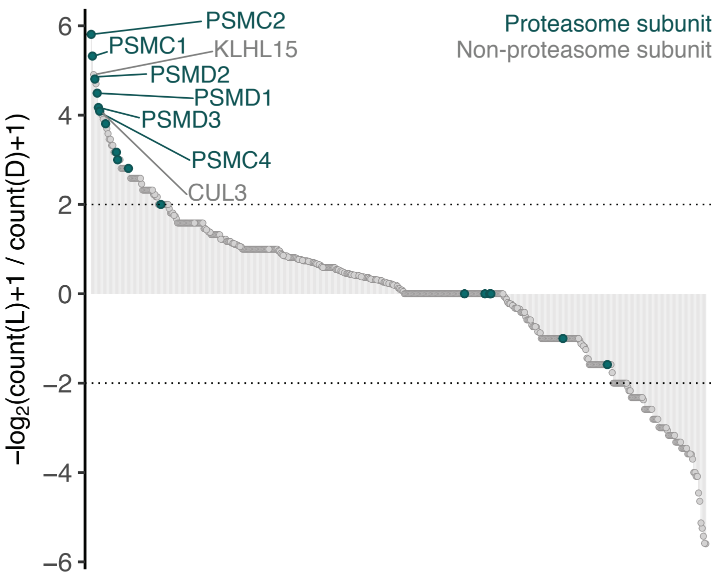

Supplementary Fig. 5: Identification of KLHL15: macrocycle interaction from label-free AP-MS.

MC1$_{biotin}$ and D-MC1$_{biotin}$, synthesized from L- and D- amino acids respectively, were incubated with lysates from HEK 293 cells, and co-purifying proteins were identified by mass spectrometry. As expected, the active L- version did co-purify with several subunits of the 26S proteasome, while the inactive D-version did not. Non proteasomal interacting partners include CUL3 and adapter KLHL15. Data represent a single experiment.

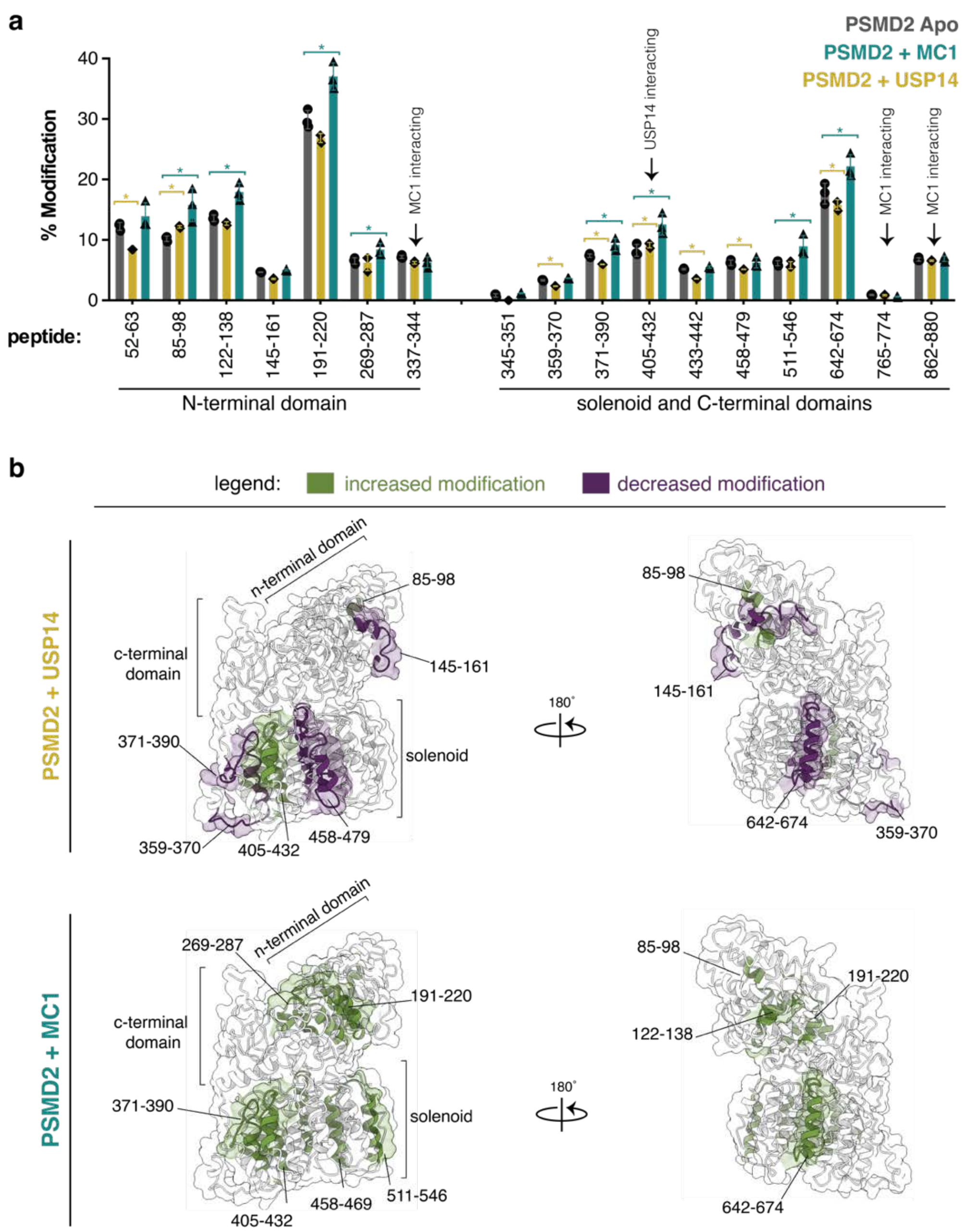

Supplementary Fig. 6: Macrocyclic ligand MC1 shows differential impact on PSMD2 dynamics compared to USP14 by hydroxyl-radical footprinting.

a, Oxidation profiles for selected PSMD2 tryptic peptides shown for apo PSMD2 (gray) and PSMD2 bound to MC1 (teal) or USP14 (gold). Sequences with color-coded asterisks were classified as having different modification levels compared to apo (* = notable difference based on heuristic classification using individual-sample percent oxidation means and confidence intervals). Percent oxidation is presented as the mean of triplicate runs after subtraction of the "no laser" background oxidation control. Error bars for each mean represent a 95% confidence interval for that mean based on the three replicates. The tryptic peptide that contains the homologous sequence to the published USP14 binding site, as well as regions that interact with MC1 in the EM structure are labeled with an arrow. **b**, Regions showing increased or decreased oxidation are mapped onto PSMD2 model PDB 6MSE chain f.

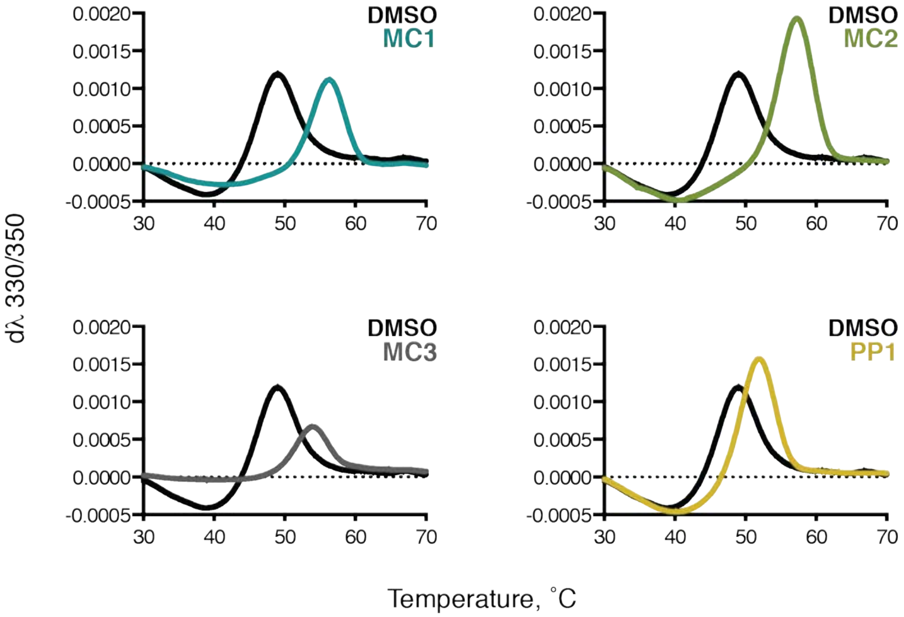

Supplementary Fig. 7: Macrocycles differentially stabilize PSMD2.
PSMD2 treated with DMSO (black), or a 10-fold excess of MC1 (teal), MC2 (green), MC3 (gray), or PP1 (gold) and thermal stability was assayed by nanoDSF. Folded state was measured by tryptophan fluorescence at 330 and 350 nm; the ratio each is plotted as a function of temperature. Curves represent mean of three technical replicates.

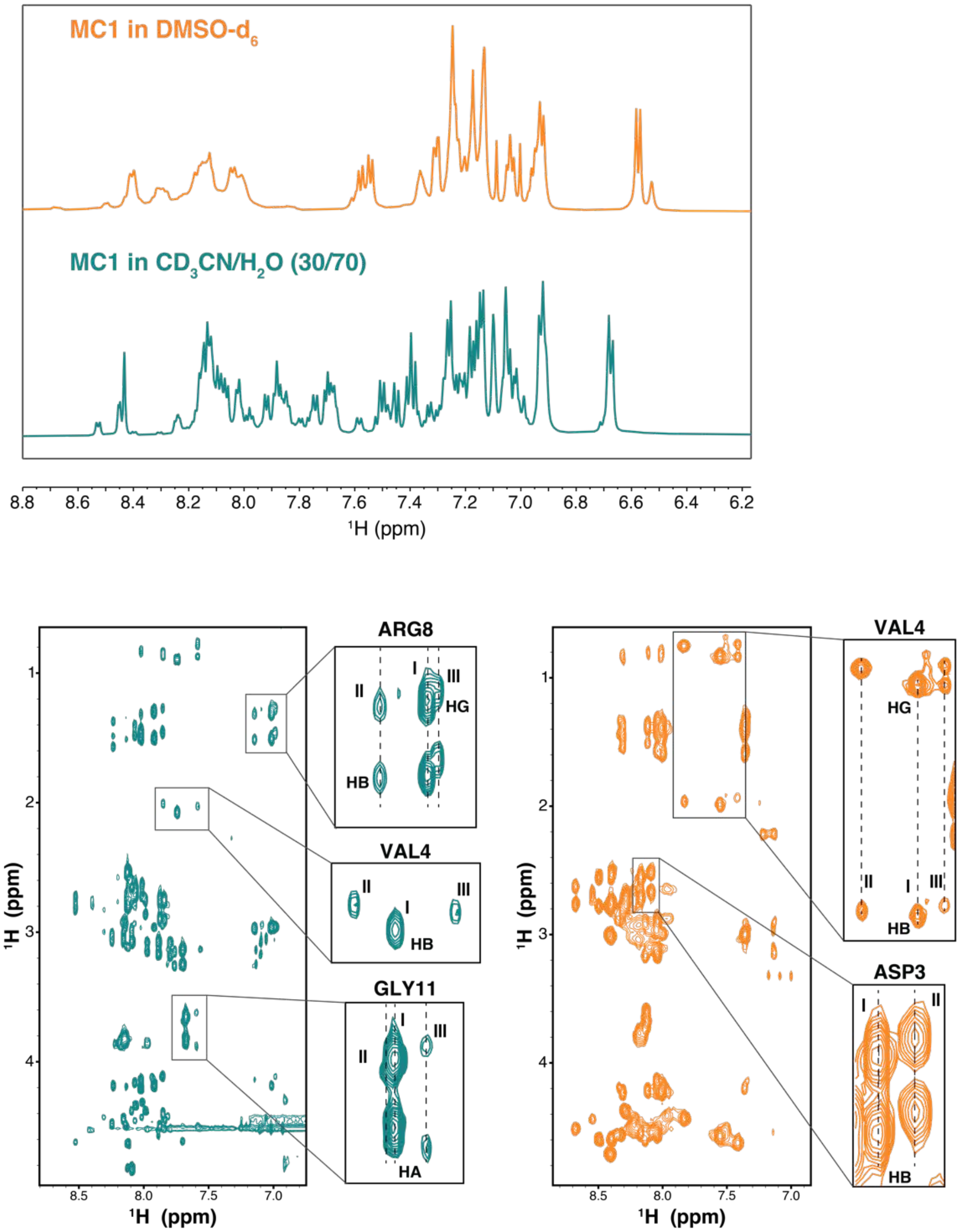

Supplementary Fig. 8: Solution NMR analysis shows that MC1 macrocyclic peptide exists in multiple conformations.

Top: aromatic and amide regions of 1D ^1H NMR spectra of macrocyclic peptide in DMSO-d_6 (orange) and CD_3CN/H_2O in (30/70) (teal) at 298K. Multiple conformers were identified in both solvents. Significant signal broadening was also observed in DMSO-d_6 suggesting conformational exchange in the intermediate exchange regime. Bottom: select regions of 2D TOCSY spectra in CD_3CN/H_2O (30/70) (left panel) and DMSO-d_6 (right panel) highlighting the distinct populations of conformers observed for several amino acids in MC1. One major population (I) and two minor populations (II and III, respectively) were observed in CD_3CN/H_2O (left panel).

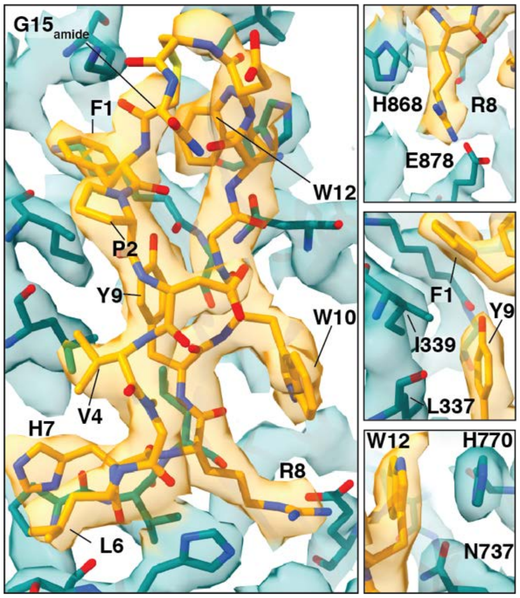

Supplementary Fig. 9: PSMD2-MC1 binding site.
Structure of PSMD2-MC1 (sticks, PSMD2 in teal and MC1 in orange) fit into the electron density map (transparent surface in corresponding colors). Images show residues within 4 Å and density within 7 Å of MC1 ligand, with nearby PSMD2 residues that do not make any contacts hidden for visual clarity. Main panel shows overview of MC1 ligand with close-ups highlighting various interactions on the right.

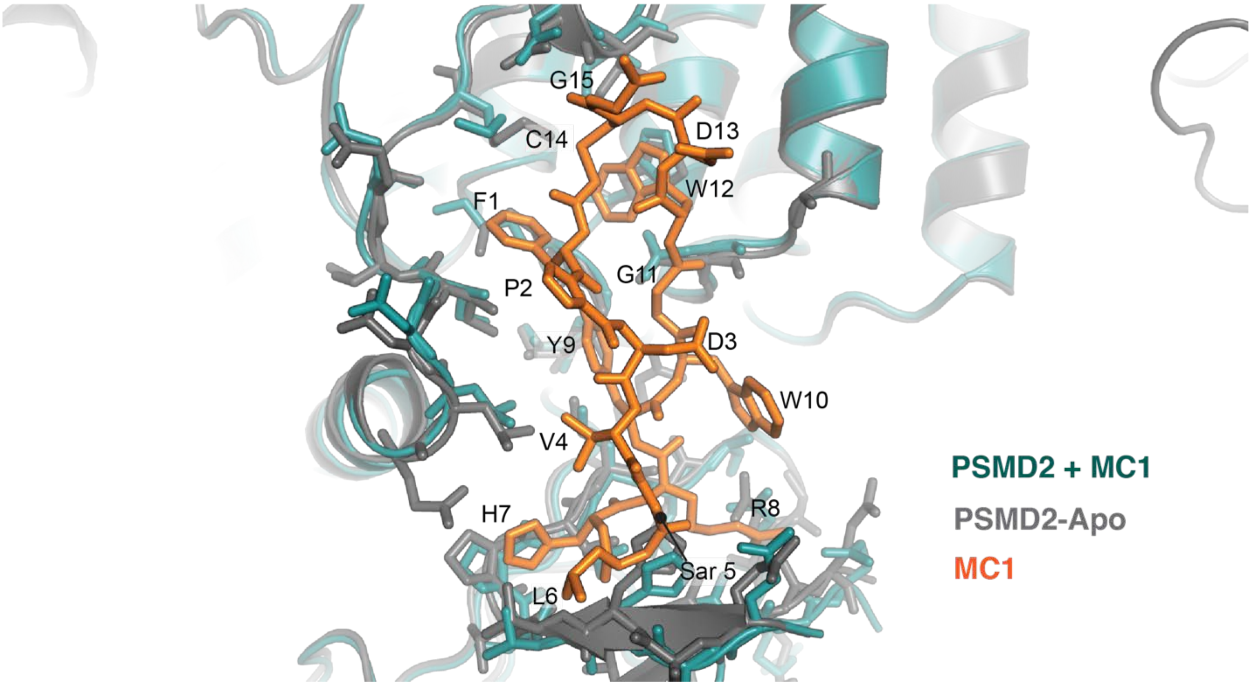

Supplementary Fig. 10: MC1 binding does not affect PSMD2 conformation at binding site.

Comparison between PSMD2-MC1 complex (in teal) and PSMD2 apo (in gray) show minimal differences in PSMD2 upon MC1 binding.

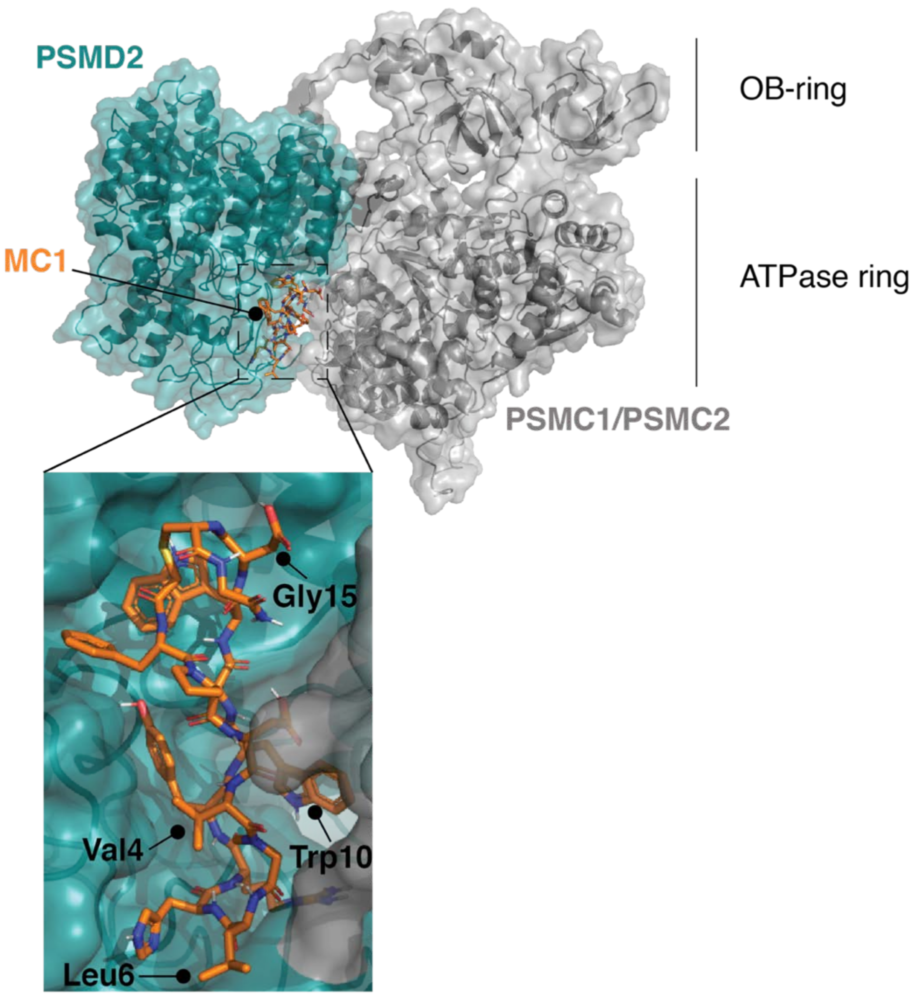

Supplementary Fig. 11: MC1 has solvent-accessible residues ideal for conjugation. Top panel: PSMD2:MC1 structure aligned to 6MSJ showing PSMD2 (teal) and the nearest AAA+ subunits PSMC1 and PSMC2 (gray). MC1 is between PSMD2 and PSMC1/2 AAA+ domains. Bottom panel: Zoom-in of binding site shows several solvent exposed residues for potential conjugation to make heterobifunctional degraders.

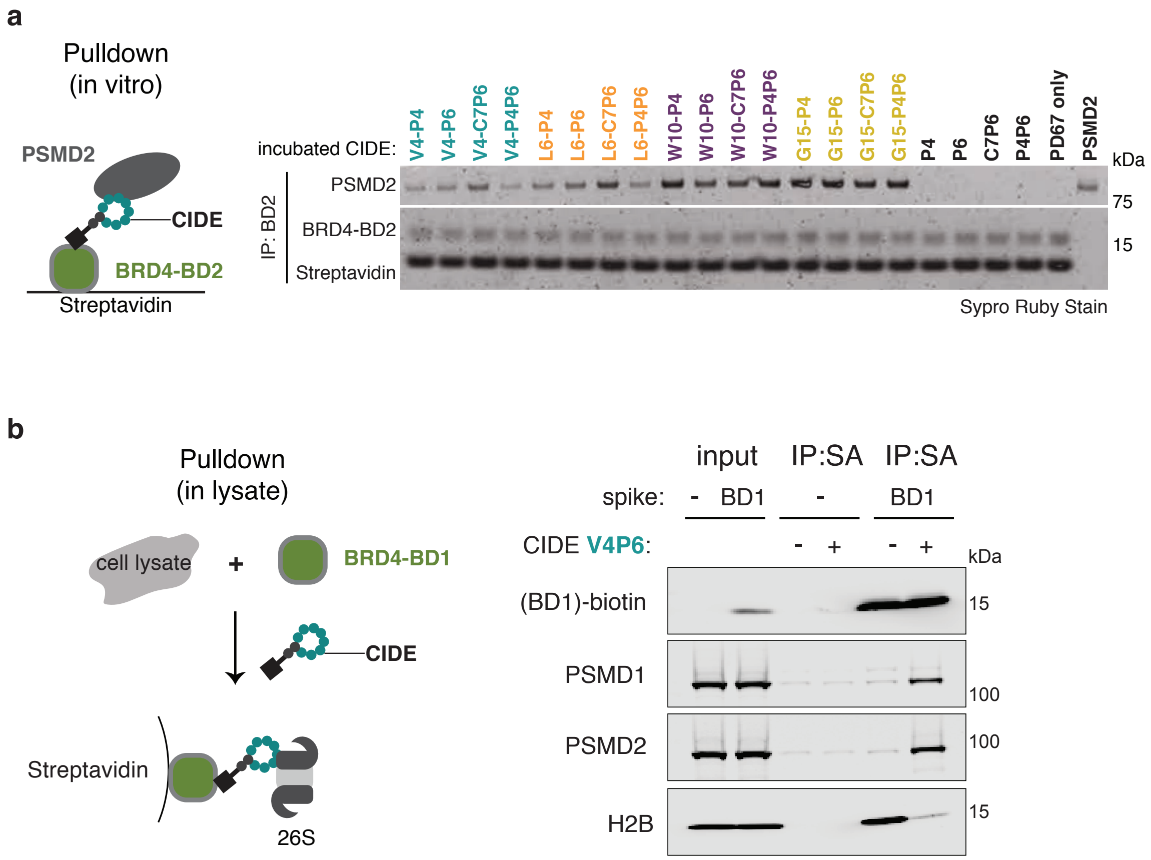

Supplementary Fig. 12: Ternary complex formation between PSMD2 and BRD4. **a**, PSMD2 pulls down with biotinylated BRD4 BD2 domain in the presence of all 16 CIDEs, but not in the presence of the BET-L+linker compounds alone, or macrocycle alone as shown by SDS-PAGE. Lanes are color coded by CIDE conjugation point as described in Figure 3a. **b**, HEK293 lysates supplemented with bortezomib, ATPγS and recombinant biotinylated BD1 interact with 26S subunits PSMD2 and PSMD1 when incubated with CIDE V4-P6 from (a), as shown my western blot. Note biotinylated BD1 protein is functional and interacts with histone protein H2B. Data in (**a**) and (**b)** were reproduced in two and three independent experiments, respectively.

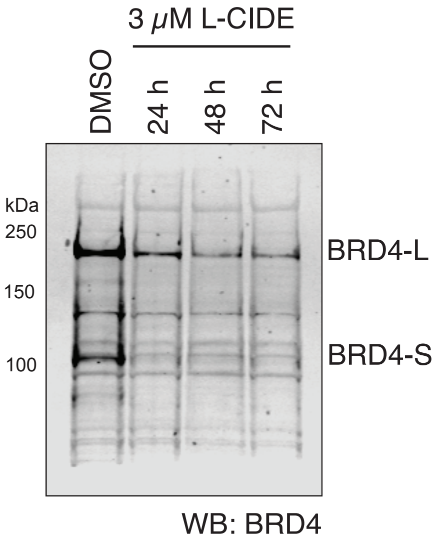

Supplementary Fig. 13: BRD4 degradation is sustained over 72 hours.
HEK293 cells treated with L-CIDE show increasing degradation of both long and short (BRD4-L and BRD4-S) forms of BRD4 over the course of 72 hours. Data were reproduced in 2 independent experiments.

Supplementary Figures 14-17
Unprocessed gels and blots from Supplementary Figures 1-13

Supplementary Fig. 14
(unprocessed blots from Supplementary Fig. 3)

Blot 1

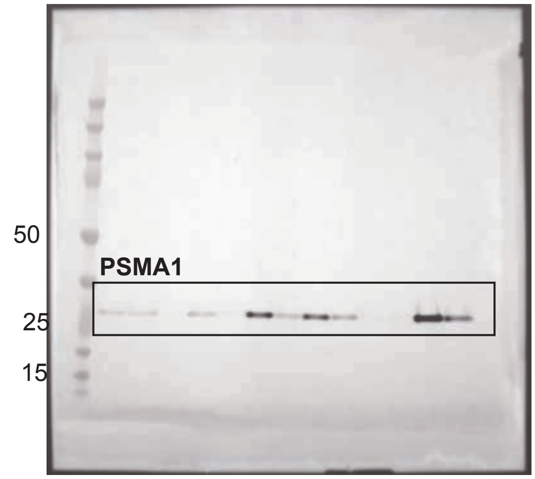

Blot 2 (Blot 1 stripped and re-probed)

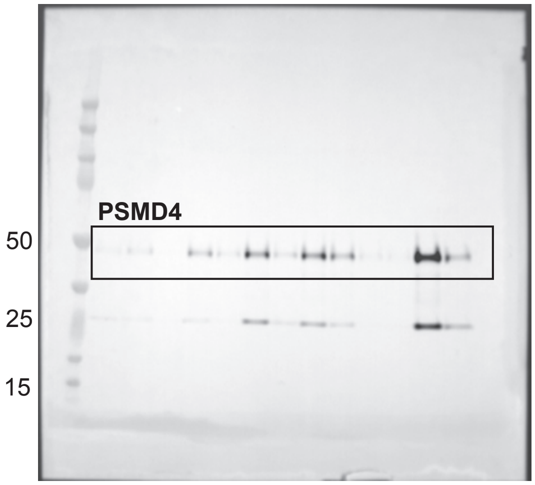

Blot 3 (Blot 2 stripped and re-probed)

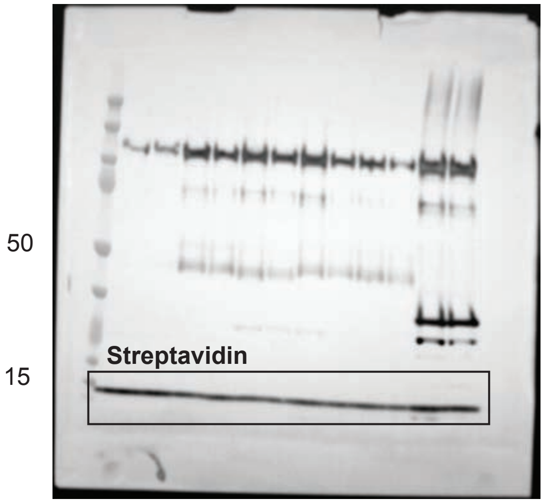

Supplementary Fig. 15
(unprocessed gel from Supplementary Fig. 12a)

Gel

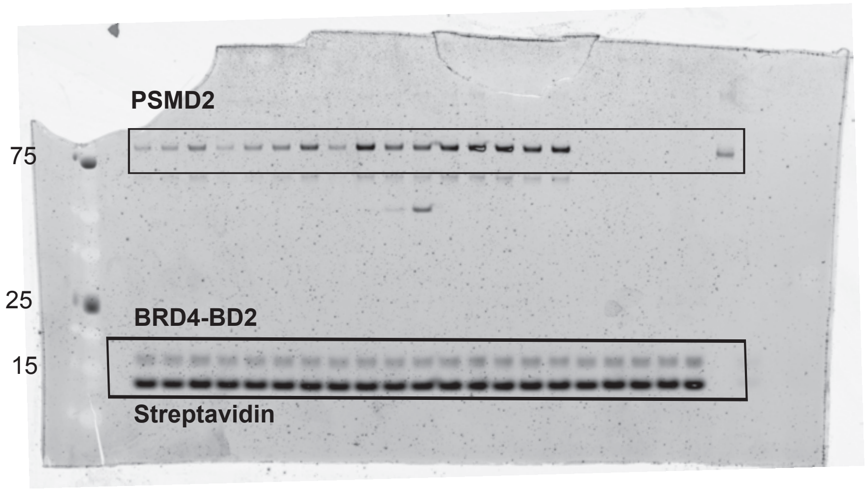

Supplementary Fig. 16
(unprocessed blots from Supplementary Fig. 12b)

Blot 1

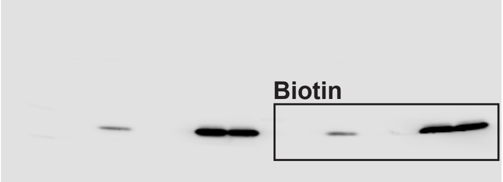

Blot 1 (with markers)

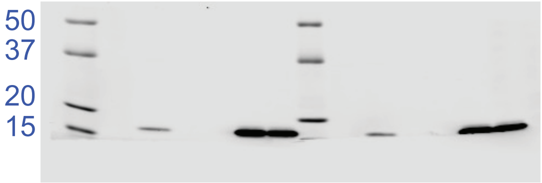

Blot 2

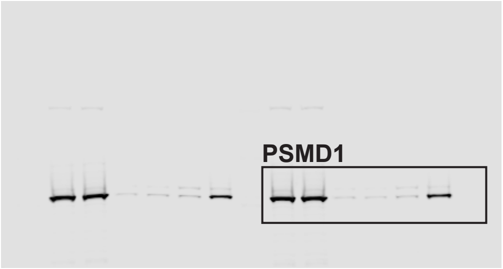

Blot 2 (with markers)

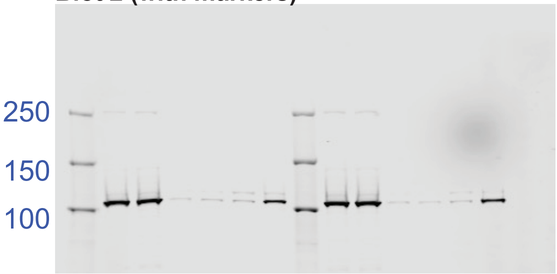

Blot 3

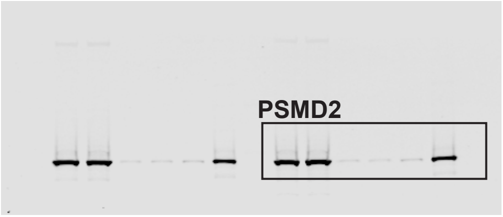

Blot 3 (with markers)

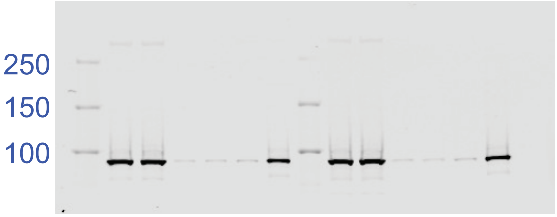

Blot 4

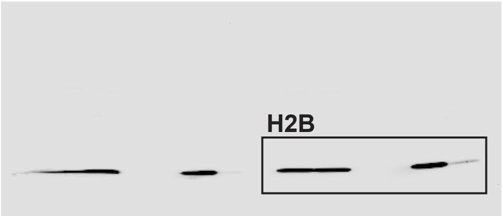

Blot 4 (with markers)

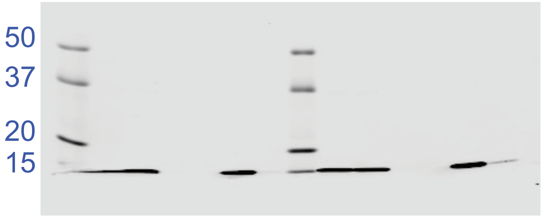

Supplementary Fig. 17
(unprocessed blots from Supplementary Fig. 13)

Blot

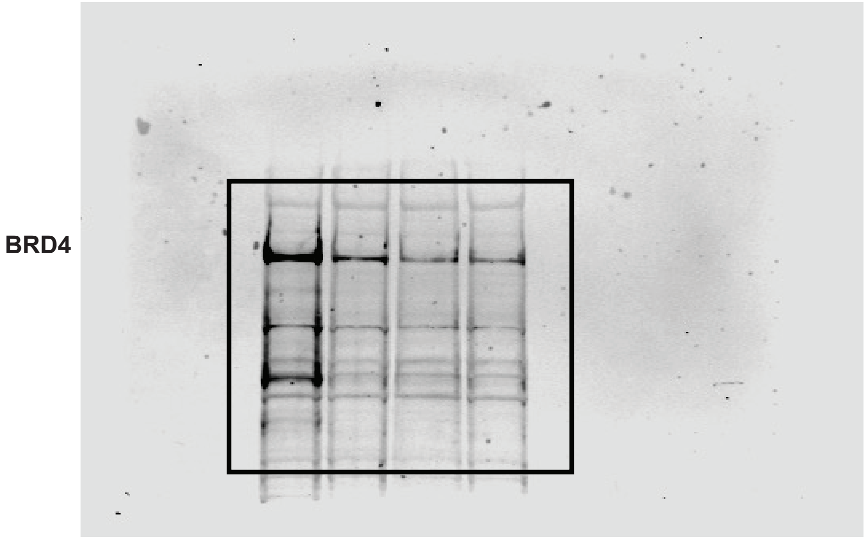

Blot (with markers)

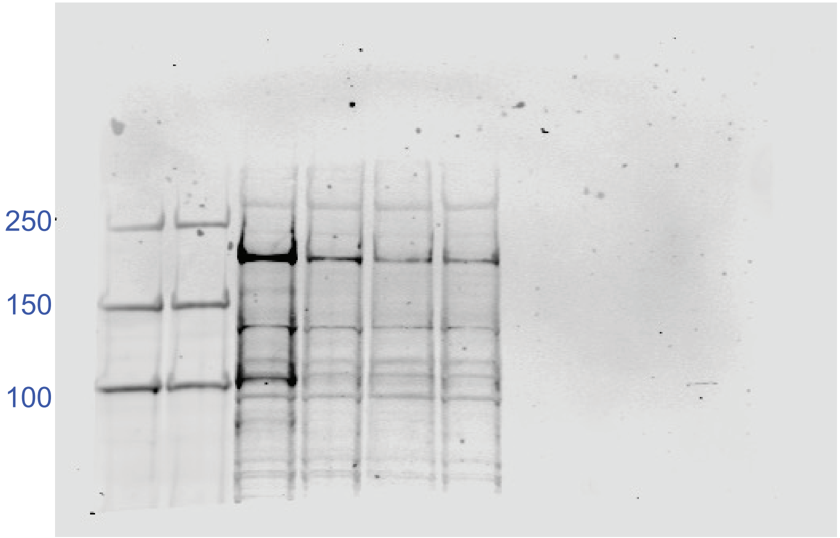

Peptides used in this study				
**full peptide name**	**nickname**	**peptide type**	**c-term modification**	**amino acid stereochemistry**
PSMD2-MC1	MC1	macrocycle		L
PSMD2-MC2	MC2	macrocycle		L
PSMD2-MC3	MC3	macrocycle		L
PSMD2-MC4	MC4	macrocycle		L
PSMD2-PP1	PP1	linear peptide		L
PSMD2-MC1$_{biotin}$	MC1$_{biotin}$	macrocycle	PEG3-Lys biotin	L
PSMD2-MC2$_{biotin}$	MC2$_{biotin}$	macrocycle	PEG3-Lys biotin	L
PSMD2-MC3$_{biotin}$	MC3$_{biotin}$	macrocycle	PEG3-Lys biotin	L
PSMD2-PP1$_{biotin}$	PP1$_{biotin}$	macrocycle	PEG3-Lys biotin	L
PSMD2-D-MC$_{biotin}$	D-MC1$_{biotin}$	macrocycle	PEG3-Lys biotin	D

Supplementary Table 1: List of peptides used in this study.
Peptides that were ordered and custom synthesized commercially for use in this study. Molecules sequences are described in Figure 1a. Macrocycles are cyclized via thioether bond as described in text and in Supplementary Figure 1. Peptides contain an N-acetyl and C-amide groups.

Sample	ka (1/Ms)	kd (1/s)	KD (M)	Rmax (RU)	Chi² (RU²)	MW	Model
MC1	60800	0.00008	1.32E-09	34.2	0.186	1500	1:1 Binding
MC1	62200	8.22E-05	1.32E-09	34.1	0.259	1500	1:1 Binding
MC1	60000	8.31E-05	1.39E-09	32.3	0.32	1500	1:1 Binding
MC2	85200	0.000132	1.55E-09	33.1	0.103	1500	1:1 Binding
MC2	85100	0.000139	1.63E-09	33.9	0.214	1500	1:1 Binding
MC2	83200	0.000146	1.75E-09	32.2	0.102	1500	1:1 Binding
MC3	92200	0.00481	5.22E-08	17.7	0.916	1100	1:1 Binding
MC3	95400	0.00452	4.73E-08	17.4	1.47	1100	1:1 Binding
MC3	90900	0.00498	5.48E-08	17.1	1.37	1100	1:1 Binding
PP1	73600	0.00917	1.24E-07	21.1	2.27	1293	1:1 Binding
PP1	78000	0.00975	1.25E-07	21.9	2.56	1293	1:1 Binding
PP1	70400	0.00772	1.1E-07	20.1	2.91	1293	1:1 Binding

Supplementary Table 2: SPR measurements and kinetic fit parameters of macrocycle and peptide ligands binding to PSMD2.

	PSMD2-PSMD2-MC1-Fabs	PSMD2-Fabs
**Data Collection**		
Magnification	165,000x	165,000x
Voltage (kV)	300	300
Electron exposure (e/Å^2)	58	64
Defocus range (μm)	0.5-1.5	0.5-1.5
Pixel size (Å)	0.824	0.824
Symmetry imposed	C1	C1
Initial particle images	1,311,869	737,190
Final particle images	105,705	82,738
Map resolution (Å) overall	2.5	3.3
FSC threshold	0.143	0.143
Map resolution range (Å)	2.7-47.6	3.1-50.0
**Refinement**		
Initial models used (PDB code)	PSMD2: 6MSJ	PSMD2: 6MSJ
Model resolution (Å)	2.9	3.5
FSC threshold	0.5	0.5
Model resolution range (Å)	2.5-47.6	3.3-50.0
Map-sharpening B-factors (Å^2)	-90	-90
Model composition		
Non-hydrogen atoms	5269	5114
Protein residues	665	662
Waters	0	0
Ligands	1	0
B factors (Å^2)		
Protein	35.36	83.38
Ligand	31.87	0
Validation		
MolProbity score	1.46	1.56
Clashscore	4.44	6.38
Poor rotamers (%)	0.00	0.00
Ramachandran Plot		
Favored (%)	96.37	96.67
Allowed (%)	3.63	3.33
Disallowed (%)	0.00	0.00
**EMDB accession #**	24742	24743
**PDB accession #**	7UJD	7UIH

Supplementary Table 3: Cryo-EM data collection, refinement and validation statistics.

MC1 - derived conjugates used in this study

Compound name	Nickname	Cmpd type	Conjugation point	Linker*	amino acid stereo-chemistry
BETcide1 G15-P4	G15-P4	CIDE	Gly15	P4	L
BETcide2 G15-P6	G15-P6	CIDE	Gly15	P6	L
BETcide3 G15-C7P6	G15-C7P6	CIDE	Gly15	C7P6	L
BETcide4 G15-P4P6	G15-P4P6	CIDE	Gly15	P4P6	L
BETcide5 V4-P4	V4-P4	CIDE	Val4	P4	L
BETcide6 V4-C7P6	V4-C7P6	CIDE	Val4	C7P6	L
BETcide7 V4-P4P6	V4-P4P6	CIDE	Val4	P4P6	L
BETcide8 V4-P6	L-CIDE	CIDE	Val4	P6	L
BETcide9 L6-P4	L6-P4	CIDE	Leu6	P4	L
BETcide10 L6-C7P6	L6-C7P6	CIDE	Leu6	C7P6	L
BETcide11 L6-P4P6	L6-P4P6	CIDE	Leu6	P4P6	L
BETcide12 L6-P6	L6-P6	CIDE	Leu6	P6	L
BETcide13 W10-P4	W10-P4	CIDE	Trp10	P4	L
BETcide14 W10-C7P6	W10-C7P6	CIDE	Trp10	C7P6	L
BETcide15 W10-P4P6	W10-P4P6	CIDE	Trp10	P4P6	L
BETcide16 W10-P6	W10-P6	CIDE	Trp10	P6	L
D-BETcide8 V4-P6	D-CIDE	CIDE	val4	P6	D
PSMD2-$MC1_{Cy5.5}$	$MC1_{Cy5.5}$	Fluorescent macrocycle	Val4		L
BETi P4	P4	BET ligand		P4	
BETi P6	P6	BET ligand		P6	
BETi C7P6	C7P6	BET ligand		C7P6	
BETi P4P6	P4P6	BET ligand		P4P6	

* P4 = PEG4, P6 = PEG6, C7P6 = -$(CH_2)_6$CONH- (C7) + PEG6, P4P6 = PEG4+PEG6. See Supporting Information for more details

Supplementary Table 4: Summary of conjugated macrocycles synthesized by click chemistry.
MC1 was conjugated to BETi ligand or Cy5.5 fluorophore as described above and in the Supporting Information.

[Compound] uM	BRD4L	BRD4S	BRD3	BRD2	Treatment
0	1	1	1	1	DMSO
0.1	0.92	0.77	0.97	1.31	L-CIDE
0.3	0.65	0.66	0.93	1.32	L-CIDE
0.6	0.38	0.55	0.77	1.45	L-CIDE
1.25	0.25	0.34	0.73	1.35	L-CIDE
2.5	0.22	0.27	0.55	1.59	L-CIDE
5	0.21	0.21	0.49	1.84	L-CIDE
0.1	1.14	0.91	1.15	1.32	D-CIDE
0.3	1.17	0.90	1.05	1.54	D-CIDE
0.6	1.55	0.98	1.10	1.28	D-CIDE
1.25	1.34	0.87	0.93	1.26	D-CIDE
2.5	0.93	0.61	0.78	1.15	D-CIDE
5	0.63	0.50	0.58	1.00	D-CIDE

Supplementary Table 5: Quantification of bands in Figure 6d western blots.
Tubulin background-corrected signal intensity was used to normalize for loading and reported band intensities normalized to DMSO control.

Treatment	BRD4L	BRD4S	BRD3	BRD2
DMSO	1	1	1	1
L-CIDE	0.28	0.25	0.49	1.03
D-CIDE	0.93	0.67	0.58	0.86
DMSO + Btz	1.99	0.93	0.79	1.30
L-CIDE + Btz	2.05	0.98	0.80	1.45
D-CIDE + Btz	2.38	1.07	0.92	1.39

Supplementary Table 6: Quantification of bands in Figure 6e western blots.
Tubulin background-corrected signal intensity was used to normalize for loading and reported band intensities normalized to DMSO control.

[BETi], uM	BRD4L	BRD4S	BRD3	BRD2	Treatment
0	1	1	1	1	DMSO
0.1	1.55	1.58	0.99	1.97	DMSO
1	1.37	1.38	1.18	2.10	DMSO
10	1.51	1.47	1.04	1.76	DMSO
0	0.24	0.23	0.40	0.96	L-CIDE
0.1	0.67	0.98	0.62	1.55	L-CIDE
1	0.79	1.06	0.68	1.93	L-CIDE
10	1.25	1.48	0.60	1.62	L-CIDE
0	1.25	0.83	0.76	0.75	D-CIDE
0.1	2.26	2.18	0.97	1.55	D-CIDE
1	2.08	2.04	0.86	1.56	D-CIDE
10	1.94	2.10	0.86	1.32	D-CIDE

Supplementary Table 7: Quantification of bands in Figure 6f western blots.
Tubulin background-corrected signal intensity was used to normalize for loading and reported band intensities normalized to DMSO control.

cell line	BRD4L	PSMD2	Treatment
NT-gRNA	1	1	D-CIDE
NT-gRNA	0.53	0.95	L-CIDE
KLHL15 KO #1	1	1	D-CIDE
KLHL15 KO #1	0.36	0.82	L-CIDE
KLHL15 KO #2	1	1	D-CIDE
KLHL15 KO #2	0.46	1.01	L-CIDE

Supplementary Table 8. Quantification of bands in Figure 6g western blots.
Histone H2B background-corrected signal intensity was used to normalize for loading and reported band intensities normalized to DMSO control.

Supplementary Tables 9-11

KLHL15 KO reagents

sgRNA	Sequence
hKLHL15_sg1	TTCATGGCAGGGGACGTGGA
hKLHL15_sg2	GACACTGGTGTCGTGGATGG
hKLHL15_sg3	GATATTCTGAATGATGGTAT
control guide RNA	GCTTATGGATTAGCCAATC
**Donor**	**Sequence**
mScarletKI long ssDNA donor	TCCCAAAGGACTTGTACATTTTGGACTTAGTCAGTAGAATTGAGATAATAGCAAAAATTGTTAAAACTCTAA<br>GTTAATATTTTCTTCTGAATGCATGGAAGTGTTTAACATTGTTGTCTTTGATTTTTTTCCAGGTGATTCATGGC<br>AGGGgctccggtgcccgtcagtgggcagagcgcacatcgcccacagtccccgagaagttggggggaggggtcggcaattgaaccggtgcc<br>tagagaaggtggcgcggggtaaactgggaaagtgatgtcgtgtactggctccgcctttttcccgagggtgggggagaaccgtatataagtgc<br>agtagtcgccgtgaacgttctttttcgcaacgggtttgccgccagaacacaggtaagtgccgtgtgtggttcccgcgggcctggcctctttacggg<br>ttatggcccttgcgtgccttgaattacttccacgcccctggctgcagtacgtgattcttgatcccgagcttcgggttggaagtgggtgggagagttc<br>gaggccttgcgcttaaggagccccttcgcctcgtgcttgagttgaggcctggcttgggcgctggggccgccgcgtgcgaatctggtggcaccttc<br>gcgcctgtctcgctgctttcgataagtctctagccatttaaaatttttgatgacctgctgcgacgctttttttctggcaagatagtcttgtaaatgcgg<br>gccaagatctgcacactggtatttcggtttttggggccgcgggcggcgacggggcccgtgcgtcccagcgcacatgttcggcgaggcggggcc<br>tgcgagcgcggccaccgagaatcggacgggggtagtctcaagctggccggcctgctctggtgcctggcctcgcgccgccgtgtatcgccccgcc<br>ctgggcggcaaggctggcccggtcggcaccagttgcgtgagcggaaagatggccgcttcccggccctgctgcagggagctcaaaatggagg<br>acgcggcgctcgggagagcgggcgggtgagtcacccacacaaaggaaaagggcctttccgtcctcagccgtcgcttcatgtgactccacgga<br>gtaccgggcgccgtccaggcacctcgattagttctcaagcttttggagtacgtcgtctttaggttggggggaggggttttatgcgatggagtttcc<br>ccacactgagtgggtggagactgaagttaggccagcttggcacttgatgtaattctccttggaatttgccctttttgagtttggatcttggttcattct<br>caagcctcagacagtggttcaaagtttttttcttccatttcaggtgtcgtgaccctagcgctacctctagagccaccatggtgagcaaaggcgaag<br>cggtgattaaagaatttatgcgctttaaagtgcatatggaaggcagcatgaacggccatgaatttgaaattgaaggcgaaggcgaaggccg<br>cccgtatgaaggcacccagaccgcgaaactgaaagtgaccaaaggcggcccgctgccgtttagctgggatattctgagcccgcagtttatgta<br>tggcagccgcgcgtttaccaaacatccggcggatattccggattattataaacagagctttccggaaggctttaaatgggaacgcgtgatgaac<br>tttgaagatggcggcgcggtgaccgtgacccaggataccagcctggaagatggcaccctgatttataaagtgaaactgcgcggcaccaacttt<br>ccgccggatggcccggtgatgcagaaaaaaaccatgggctgggaagcgagcaccgaacgcctgtatccggaagatggcgtgctgaaaggc<br>gatattaaaatggcgctgcgcctgaaagatggcggccgctatctggcggattttaaaaccacctataaagcgaaaaaaccggtgcagatgcc<br>gggcgcgtataacgtggatcgcaaactggatattaccagccataacgaagattataccgtggtggaacagtatgaacgcagcgaaggccgc<br>catagcaccggcggcatggatgaactgtataaataaGCTAGCaacttgtttattgcagcttataatggttacaaataaagcaatagcatcac<br>aaatttcacaaataaagcatttttttcactgcattctagttgtggtttgtccaaactcatcaatgtatcttaAGCGTTTTTGAGAAGGTTAG<br>TGCATTTGAAAGCAATAGACAGTACTCATCATTTAGTTAAGGCAGTGTATGTCTTTATAGATAAGATTCACAG<br>ACTTAAGAGCCAAGAATGAATAACTTCATTTTGTTATTTCTTATGTTATTCAAGAAT

Supplementary Table 9. sgRNA and donors

Primer	Sequence
hKLHL15_genF	TCCTTAGATAGGGAAGCCAGAAA
hKLHL15_genR	ACGTGGTTTATCTTTCACCCACA

Supplementary Table 10. Genomic PCR oligos

Primer	Sequence	Notes
hKLHL15-ddF1	GATAGGGAAGCCAGAAATATTAGTC	control, sg1, sg2 and mScarlet knock-in assays
hKLHL15_conddR	ACACTTCCATGCATTCAGAAGAA	control assay
hKLHL15-ddR1	AAGCAATCCCTCCTCATACA	sg1 and sg2 assays
hKLHL15KI-ddR1	CGACATCACTTTCCCAGTTTAC	mScarlet knock-in assay
hKLHL15-ddF2	AGCAGGTTCCCAGAGATAGA	sg3 assay
hKLHL15-ddR2	TAAATGCACAACAAATCAGGCT	sg3 assay
**Probe**	**Sequence**	
hKLHL15-WTcon-Hex	AA+C+CTTT+A+G+ATT+GTCT	control assay probe
hKLHL15-sg1FAM	CTT+C+C+A+CGT+CC	sg1 cutsite probe
hKLHL15-sg2FAM	CTC+C+A+T+CC+A+CGA	sg2 cutsite probe
hKLHL15-sg3FAM	CC+GA+T+A+C+CA+TCA	sg3 cutsite probe
hKLHL15-KI-FAM	CG+GT+T+CAA+T+T+GC	HDR knock-in probe
**Centromeric copy number reference assay**	**Assay ID**	
PLEKHF1 FAM	dHsaCP2506315	Bio-Rad
PLEKHF1 HEX	dHsaCP2506723	Bio-Rad

Supplementary Table 11. ddPCR sequences

Supplementary Note

(synthesis of MC1-Cy5.5 and CIDEs)

General Methods

A. Materials

All chemical reagents for the synthesis of BET ligands were purchased form Sigma Aldrich. Fmoc amino acids where purchased from commercial sources namely, EMD Millipore, Chem-Impex, CEM and Bachem. OxymaPure® (ethyl (2*E*)-2-cyano-2-hydroxyiminoacetate) and Rink amide ProTide™ resin were purchased from CEM. *N*,*N*-Diisopropylcarbodiimide (DIC), 4-methylpiperidine and Chelex 100 resin were purchased from Chem-Impex, Beantown Chemical and Bio-Rad, respectively. All solvents and other chemical reagents for peptide synthesis were purchased from Millipore Sigma.

B. Methods and General experimental procedures

I. Synthesis of BET Ligands

Synthetic Scheme for compound 16

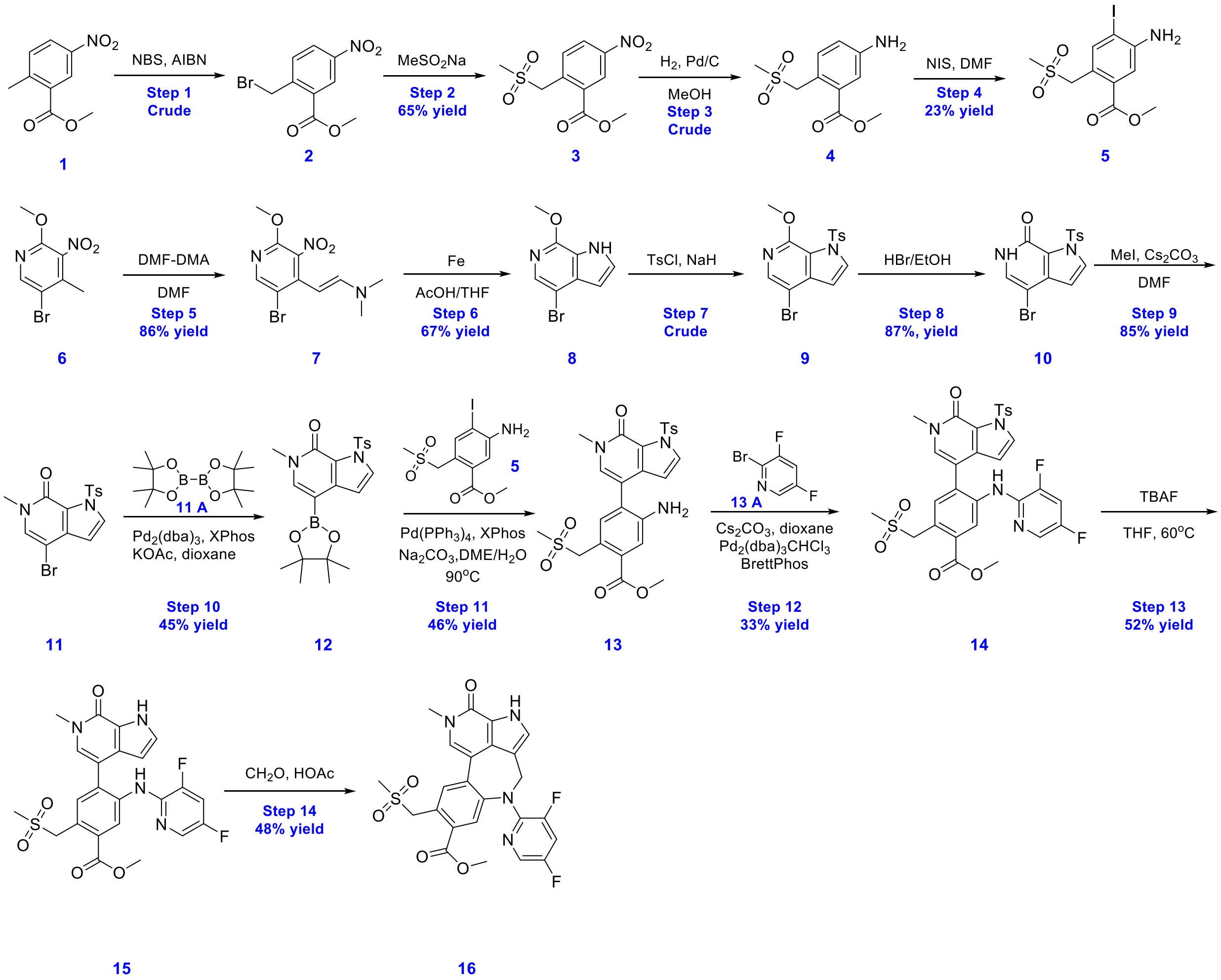

Experimental Procedure for BET Ligands:

1. *Preparation of Compound 2*

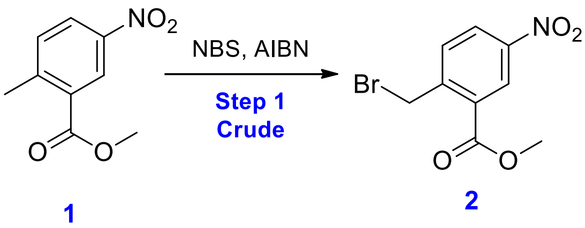

To a solution of Compound **1** (370 g, 1.90 mol) in ACN (2.50 L) was added AIBN (156 g, 948 mmol) at 25°C, and the reaction was heated to 80°C. NBS (506 g, 2.84 mol) was added in batches at 80°C and the resulting solution was stirred for 1 hour at 80°C. The reaction mixture

was concentrated to remove solvent and dissolved in ethyl acetate (2.00 L). The mixture was then washed with water (500 mL x 3). The aqueous was extracted with ethyl acetate (500 mL x 3)and the combined the organics were washed with brine (200 mL) and concentrated to give a residue. The crude product, compound **2** (550 g, crude), was obtained as a brown solid and used without purification.

2. *Preparation of Compound 3*

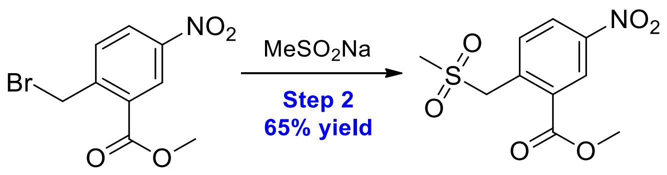

2 **3**

A solution of **2** (277 g, 1.01 mol) in DMF (1.50 L) was treated with $MeSO_2Na$ (155 g, 1.52 mol) at 25°C. The resulting solution was heated to 60°C and stirred for 2 hours. Two reactions of the same scale were combined for work-up. The combined reaction mixtures were diluted with 15.0 L of water and filtered. The resultant solid was dissolved in ethyl acetate (8.00 L), extracted with water (2.00 L x 3) and brine (2.00 L) and the organic dried over Na_2SO_4. Concentration and trituration with Petroleum ether/ethyl acetate = 5/1 (3.00 L) delivered compound **3** (380 g, 1.32 mol, 65.2% yield, 94.7% purity) as a brown solid. ^1H NMR (400 MHz, $CDCl_3$): δ 8.88 (s, 1 H), 8.41 (dd, *J* = 8, 2 Hz, 1 H), 7.78 (d, *J* = 8 Hz, 1 H), 5.03 (s, 1 H), 4.00 (s, 3 H), 2.91 (s, 3 H). LCMS (ESI): *m/z* 273 $(M+H)^+$.

3. *Preparation of Compound 4*

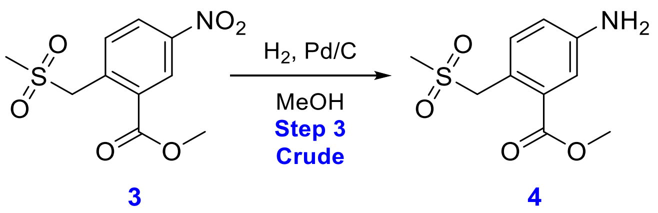

A solution of **3** (98.0 g, 359 mmol) in MeOH (1.00 L) was treated with Pd/C (20.0 g, 10% purity) under N_2. The suspension was degassed under vacuum and purged with H_2 several times. The mixture was stirred under H_2 (50 psi) at 30°C for 2 hours. Five reaction mixtures of this scale were combined for work-up. The combined reaction mixtures were filtered and concentrated. The crude product was triturated with MTBE/MeOH = 3/1 (1 L) to give **4** (448 g) as a white crude solid that was used without further purification.

4. General procedure for preparation of Compound 5

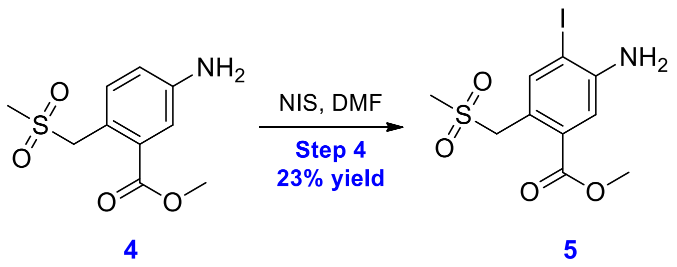

A solution of **4** (308 g, 1.27 mol) in DMF (2.10 L) was treated with NIS (308 g, 1.37 mol) at 25°C and stirred for 2 hours. The reaction mixture was diluted with 5.00 L of saturated Na_2SO_3 solution and 5.00 L of saturated $NaHCO_3$ solution. The solid was collected by filtration and purified by column chromatography (SiO_2, Petroleum ether/Ethyl acetate = 100/1 to 0/1) followed by trituration with ethyl acetate (1.00 L) to give **5** (110 g, 298 mmol, 23.5% yield) as a brown solid. ^{1}H NMR (400 MHz, DMSO-d6): δ 7.72 (s, 1 H), 7.25 (s, 1 H), 5.69 (s, 2H), 4.70 (s, 2H), 3.78 (s, 3 H), 2.81 (s, 3 H).

5. Preparation of Compound 7

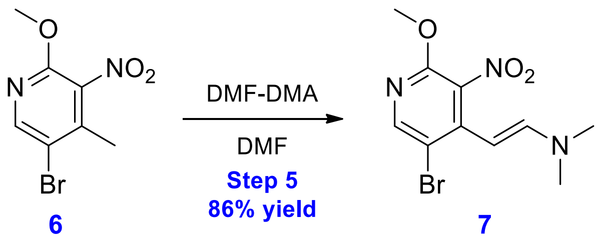

A solution of **6** (150 g, 607 mmol) in DMF (1.20 L) at 25°C and was treated with DMF-DMA (606 g, 5.08 mol, 675 mL) at 80° C in portions over 30 min. The reaction mixture was stirred at 95° C for 2.5 hours. Three reaction of the same scale were combined for work-up. The combined reaction mixtures were dissolved in ethyl acetate (10.0 L), washed with brine (200 mL x 3), dried over Na_2SO_4 and concentrated give crude **7** (530 g, 1.75 mol, 96.3% yield) reddish solid that was used without further purification. ^{1}H NMR (400 MHz, $CDCl_3$): δ 8.11 (s, 1 H), 6.99 (d, *J* = 14 Hz, 1 H), 4.92 (d, *J* = 14 Hz, 1 H), 3.94 (s, 3 H), 2.92 (s, 6H).

6. Preparation of Compound 8

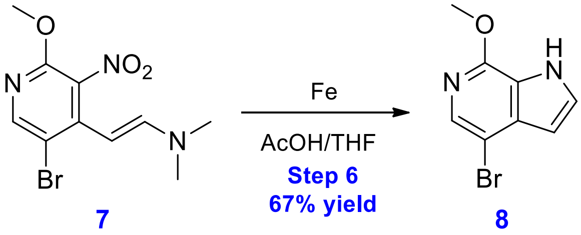

A solution of **7** (167 g, 554 mmol) in THF (1.67 L) was treated with AcOH (800 mL) at 25°C. The mixture was heated to 60° C, treated with iron powder (170 g, 3.05 mol) and stirred for 8 hours at 60°C. Two reactions were combined for work-up. The combined reaction mixtures were concentrated, dissolved in ethyl acetate (5.00 L), washed with saturated $NaHCO_3$ solution

(1.00 L x 3), water (400 mL x 3) and brine (500 mL). The organic was concentrated and purified by column chromatography (SiO_2, Petroleum ether/Ethyl acetate = 100/1 to 0/1) to deliver **8** (230g, 1.01 mol, 67.3% yield) as light yellow solid. ^{1}H NMR (400 MHz, DMSO- d6): δ 12.16 (s, 1 H), 7.75 (s, 1 H), 7.56-7.55 (m, 1 H), 6.43-6.42 (m, 1 H), 4.00 (s, 3 H).

7. Preparation of Compound 9

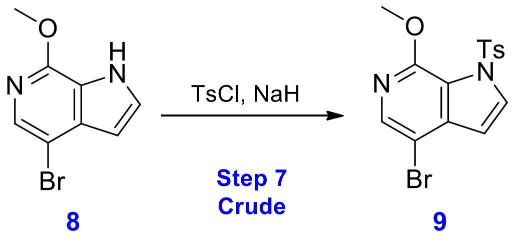

A 5.00 L 3-necked round-bottom flask purged with nitrogen and charged with NaH (41.66 g, 1.04 mol, 60% purity) and THF (1.00 L). Compound **8** (215 g, 947 mmol) was dissolved in THF (400 mL) and added dropwise with stirring at 0 °C. TsCl (217 g, 1.14 mol) was added in portions. The resulting solution was stirred for 1 hour at 0 °C. Two reactions were combined for workup. The mixture was diluted with NH_4Cl solution (500 mL), extracted with 3 x 500 mL of ethyl acetate. The organic layers were combined and washed with brine (100 mL x 2), dried over Na_2SO_4 and concentrated under vacuum to deliver **9** (380 g, crude) as a white solid. ^{1}H NMR (400 MHz, DMSO- d6): δ 8.18 (d, *J* = 4 Hz, 1 H), 7.99 (s,1 H), 7.85 (d, *J* = 8 Hz, 2 H), 7.46 (d, *J* = 8 Hz, 2 H), 6.81 (d, *J* = 4 Hz, 1 H), 3.82 (s, 3 H), 2.38 (s, 3 H).

8. Preparation of Compound 10

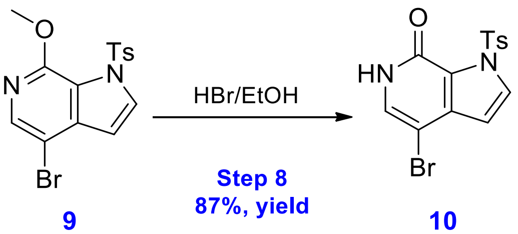

9 10

A solution of **9** (380 g, 997 mmol) in EtOH (1.00 L) was treated with HBr (1.79 kg, 8.84 mol, 1.20 L, 40% purity) at 25°C then warmed to 90°C to stir for 2 hoursThe reaction mixture was adjusted to pH = 7-8 with saturated $NaHCO_3$ solution and extracted with 2-Me-THF (2.00 L x 4). The layers were separated and the organic layer was washed with water (1.00 L x 3) and brine (1.00 L), dried over Na_2SO_4 and concentrated to give a residue. The crude product was triturated with MTBE (600 mL), and filtration delivered **10** (320 g, 871 mmol, 87.4% yield) as a light brown solid. ^{1}H NMR (400 MHz, DMSO- d6): δ 11.50 (s, 1 H), 8.04 (d, *J* = 3 Hz, 1 H), 7.93 (d, *J* = 8 Hz, 2 H), 7.41 (d, *J* = 8 Hz, 2 H), 7.36 (s, 1 H), 6.60 (d, *J* = 3 Hz, 1 H), 2.37 (s, 3 H).

9. Preparation of Compound 11

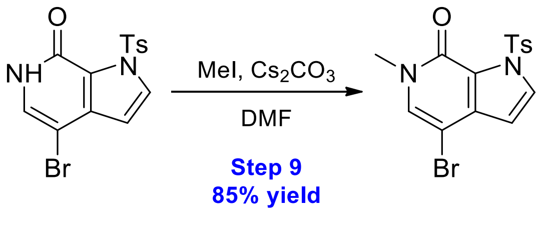

10 11

A solution of **10** (225 g, 613 mmol) in DMF (1.57 L) was treated with Cs_2CO_3 (240 g, 735 mmol) and MeI (153 g, 1.08 mol, 67.2 mL and stirred at 25°C for 3 hours. Trituration of the reaction mixture with petroleum ether/ethyl acetate (5/1, 300mL) gave **11** (204 g, 525 mmol, 85.7%

yield, 98.1% purity) as a light brown solid. ^{1}H NMR (400 MHz, DMSO- d6): δ 8.06 (d, *J* = 4 Hz, 1 H), 7.95 (d, *J* = 8 Hz, 2 H), 7.80 (s, 1 H), 7.42 (d, *J* = 8 Hz, 2 H), 6.60 (d, *J* = 4 Hz, 1 H), 3.39 (s, 3 H), 2.38 (s, 3 H). LCMS (ESI): *m/z* 382 (M+H)$^+$.

10. Preparation of Compound 12

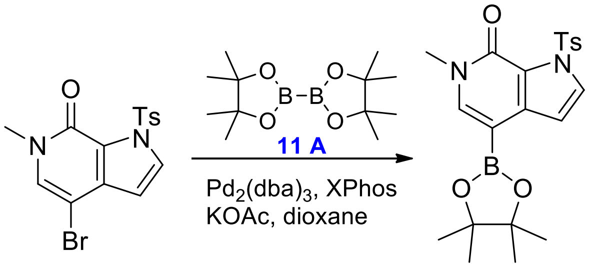

Step 10
45% yield

11 **12**

Compound **11** (84.0 g, 220 mmol) and KOAc (54.1 g, 551 mmol) were dissolved in dioxane (1.30 L) under N_2 atmosphere. Then the mixture was treated with (112 g, 441 mmol), $Pd_2(dba)_3$ (10.1 g, 11.0 mmol) and XPhos (10.5 g, 22.0 mmol). The reaction mixture was purged with argon and stirred at 90°C for 2 hours. Three reactions were combined for work-up. The combined reaction mixtures were concentrated, dissolved in ethyl acetate (1.00 L) and washed with H_2O (200 mL x 3). The aqueous phase was extracted with ethyl acetate (200 mL x 2), then the combined organics were washed with brine (100 mL), dried over Na_2SO_4, concentrated and the resultant solid purified by column chromatography (ether/Ethyl acetate = 100/1 to 0/1). Trituration with n-heptane (200 mL) gave **12** (140 g, 299 mmol, 45.3% yield, 91.5% purity) as a grey solid. NMR (400 MHz, $CDCl_3$): δ 7.97 (d, *J* = 8 Hz, 2 H), 7.88 (d, *J* = 3 Hz, 1 H), 7.51 (s, 1 H), 7.29~7.26 (m, 2 H), 6.90 (d, *J* = 3 Hz, 1 H), 3.52 (s, 3 H), 2.39 (s, 3 H), 1.33 (s, 12H). LCMS (ESI): *m/z* 429 (M+H)$^+$.

11. Preparation of Compound 13

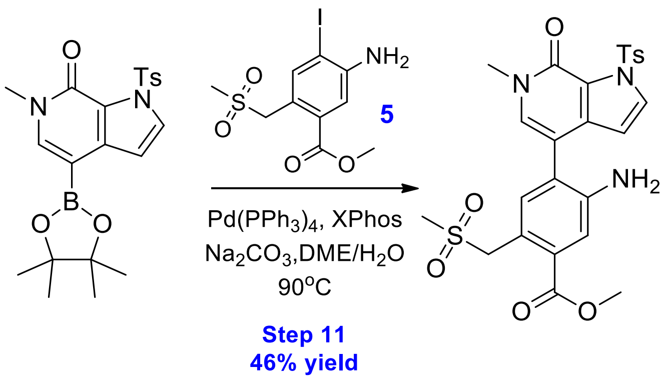

12 13

A 3.00 L 3-necked round-bottom was purged with argon and charged with **12** (70.0 g, 163 mmol), **5** (60.3 g, 163 mmol), XPhos (15.6 g, 32.7 mmol), DME (700 mL), Na_2CO_3 (34.6 g, 327 mmol), $Pd(PPh_3)_4$ (18.9 g, 16.3 mmol) and H_2O (700 mL). The resulting solution was stirred for 4 hours at 90 °C. Two reactions of this scale were combined for work-up. The mixtures were concentrated, and the resultant solid triturated with DCM (200 mL) to give **13** (90.0 g, 151 mmol, 46.2% yield, 91.2% purity) was obtained as yellow solid. NMR (400 MHz, $CDCl_3$): δ 8.05~8.03 (d, *J* = 8 Hz, 2 H), 7.91 (d, *J* = 4 Hz, 1 H), 7.42 (s, 2 H), 7.33 (d, *J* = 8 Hz, 2 H), 7.31 (s, 1 H), 7.00 (s, 1 H), 6.34 (d, *J* = 4 Hz, 1 H), 4.77 (s, 2 H), 3.92 (s, 3 H), 3.55 (s, 3 H), 2.82 (s, 3 H), 2.42 (s, 3 H). LCMS (ESI): *m/z* 544 $(M+H)^+$.

12. Preparation of Compound 14

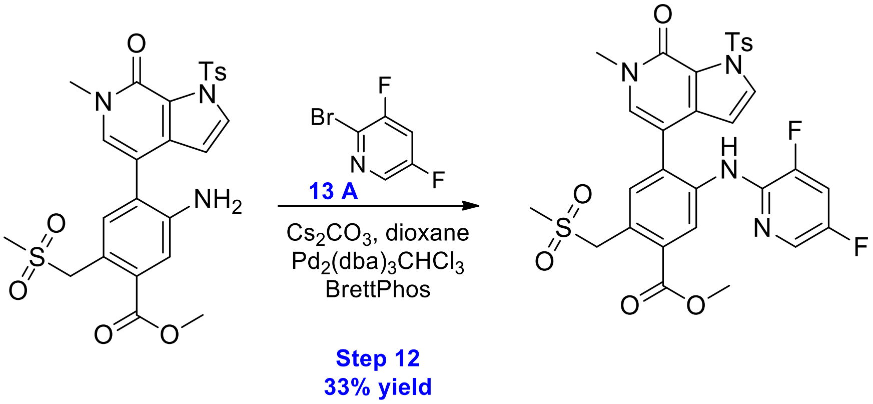

13 14

A 2.00 L 3-necked round-bottom was purged with nitrogen and charged with **13** (84.5 g, 155 mmol), **13 A** (60.3 g, 311 mmol), Cs_2CO_3 (101 g, 311mmol), dioxane (720 mL), BrettPhos (66.8 g, 124 mmol) and $Pd_2(dba)_3CHCl_3$ (56.9 g, 62.2 mmol). The reaction mixture was heated to 90°C for 4 hours, cooled to 25°C and dissolved into DCM (500 mL). The mixture was filtered through celite (DCM wash) and the combined organics were washed with water (200 mL x 3), brine (100 mL) and then dried over Na_2SO_4. Concentration followed by trituration with ethyl acetate (300 mL) at yielded **14** (35.0 g, 51.9 mmol, 33.3% yield, 97.3% purity) as a pale green solid. ^{1}H NMR (400 MHz, DMSO- d6): δ 8.29 (s, 1 H), 8.15 (s, 1 H), 7.91~7.89 (m, 3 H), 7.85~7.84 (m, 1 H), 7.73~7.70 (m, 1 H), 7.48 (d, *J* = 8 Hz, 2 H), 7.43 (d, *J* = 8 Hz, 2 H), 6.31 (d, *J* = 3 Hz, 1 H), 4.93 (s, 2 H), 3.84 (s, 3 H), 3.40 (s, 3 H), 2.93 (s, 3 H), 2.39 (s, 3 H). LCMS (ESI): *m/z* 657 $(M+H)^+$.

13. Preparation of Compound 15

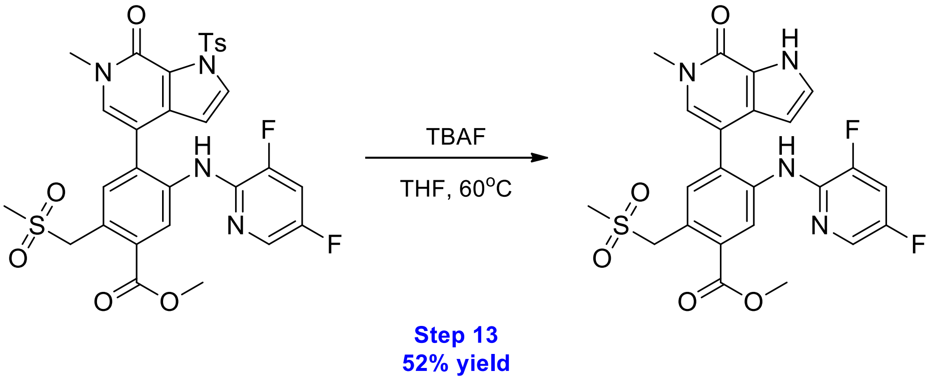

14

15

A solution of **14** (30.0 g, 45.7 mmol) in THF (100 mL) was treated with TBAF (1 M, 137 mL) at 25°C and the mixture was stirred to 60°C for 3 hours. The reaction mixture was diluted with DCM (100 mL), filtered through a pad of celite and the filtrate was washed with H_2O (50.0 mL x 3) and brine (50.0 mL). Trituration of the concentrated organics with ethyl acetate (50.0 mL) at gave **15** (13.0 g, 24.0 mmol, 52.6% yield, 92.8% purity) a pale green solid. ^{1}H NMR (400 MHz, DMSO- d6: δ 12.08 (s, 1 H), 8.40 (s, 1 H), 7.96~7.93 (m, 1 H), 7.74~7.73 (m, 1 H), 7.73~7.71 (m, 1 H), 7.55 (s, 1 H), 7.33 (s, 1 H), 7.26~7.25 (m, 1 H), 6.06 (t, *J* = 2 Hz, 1 H), 4.95 (s, 2 H), 3.85 (s, 3 H), 3.53 (s, 3 H), 2.94 (s, 3 H). LCMS (ESI): *m/z* 503 $(M+H)^+$.

14. Preparation of Compound 16

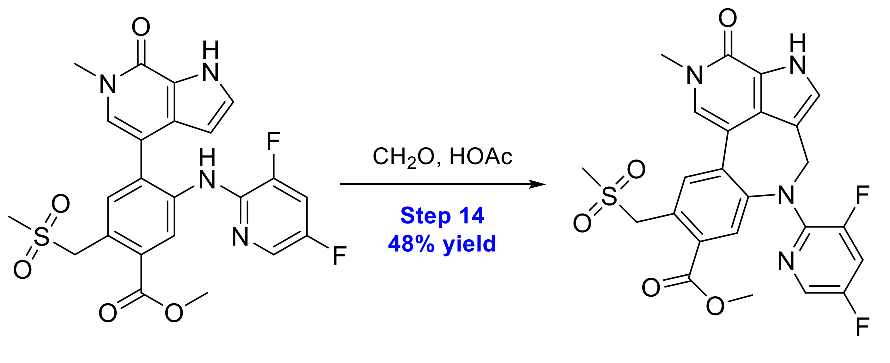

A 500-mL 3-necked round-bottom flask was purged with nitrogen and charged with **15** (13.0 g, 25.9 mmol), AcOH (130 mL) and HCHO (6.30 g, 77.6 mmol, 5.78 mL, 37% purity). The resulting solution was stirred for 2 h at 75 ^{o}C. The reaction was filtered and the solid washed with petroleum ether. The solid was dissolved in DCM (200 mL) and the pH was adjusted to pH = 8 with saturated $NaHCO_3$ solution. The organic phase was washed with H_2O (100 mL) and brine (50.0 mL), dried over Na_2SO_4, filtered and concentrated to give a residue. Two reactions were combined and the crude solid was triturated with MeOH (200 mL) to give **16** (10.5 g, 0.02 mol, 48.8% yield, 98.2% purity) as a pale yellow solid. ^{1}H NMR (400 MHz, DMSO- d6: δ 11.96 (d, *J* = 2 Hz, 1 H), 8.09 (d, *J* = 2 Hz, 1 H), 8.00 (s, 1 H), 7.83 (s, 1 H), 7.68~7.62 (m, 1 H), 7.57 (s, 1 H), 7.28 (d, *J* = 2 Hz, 1 H), 5.92 (br, 1 H), 5.12~4.86 (m, 2 H), 4.29 (br, 1 H), 3.78 (s, 3H), 3.63 (s, 3 H), 2.95 (s, 3 H). LCMS (ESI): *m/z* 515 $(M+H)^{+}$.

Synthetic scheme for preparation of compound 18 (BETi P4)

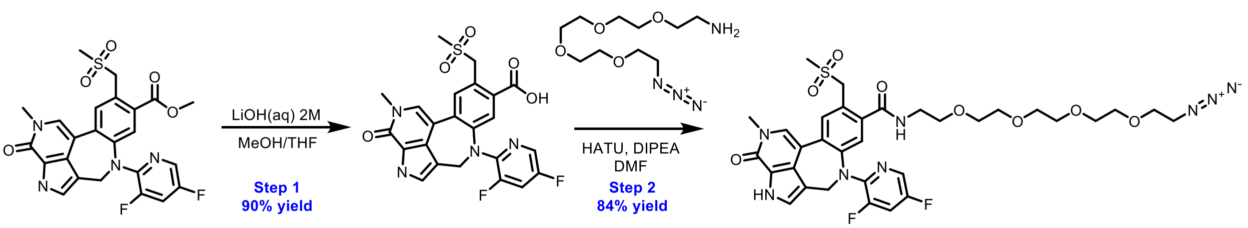

16 **17** **18**

Experimental Procedure:

1. Preparation of Compound 17

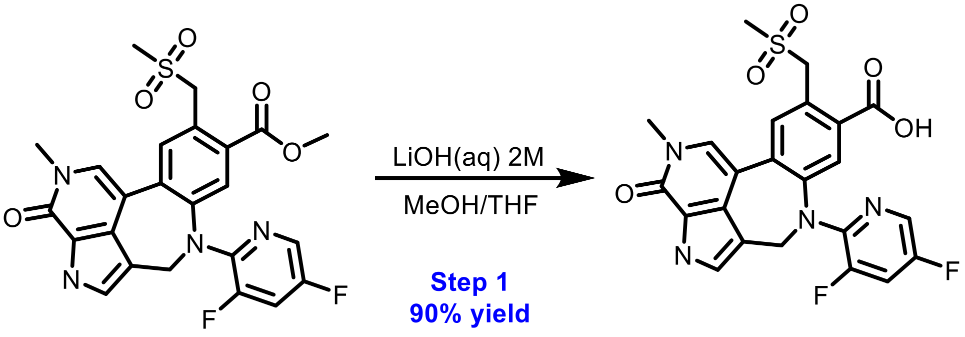

16 **17**

A mixture of compound **16** (950 mg, 1.85 mmol) and LiOH 2M (3.7 mL, 7.38 mmol, 4 equiv.) in THF (18 mL, 0.1 M) / MeOH (6.2 mL, 0.3 M) was stirred at room temperature for 4h. The volatiles were removed under vacuum. The residue was taken up in THF and HCl was added (2M, 3.7 mL, 7.38 mmol, 4 equiv.). The mixture was concentrated under vacuum and purified by reverse phase chromatography (20-80% ACN/0.05% TFA in water). The fractions containing the desired product were combined, concentrated and lyophilized to obtained **17** (831 mg, 1.66 mmol, 90% Yield) as a yellow solid. 1H NMR (400 MHz, DMSO-d6) δ 11.93 (s, 1H), 8.09 (d, J = 2.6 Hz, 1H), 7.97 (s, 1H), 7.81 (s, 1H), 7.70 – 7.60 (m, 1H), 7.58 (s, 1H), 7.28 (d, J = 2.6 Hz, 1H), 3.63 (s, 3H), 2.93 (s, 3H). LCMS (ESI): *m/z* 501 $(M+H)^+$.

Preparation of compound 18

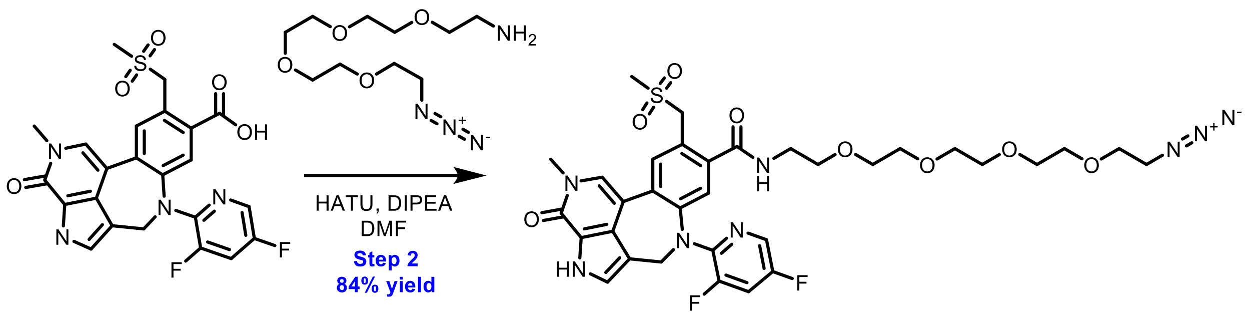

17 18

A mixture of compound 17 (150 mg, 0.299 mmol), 14-azido-3,6,9,12-tetraoxatetradecan-1-amine (113 µL, 0.449 mmol, 1.5 equiv.), HATU (141 mg, 0.359 mmol, 1.2 equiv.) and DIPEA (105 µL, 0.599 mmol, 2 equiv.) in DMF (1.5 mL, 0.2 M) was stirred at room temperature for 45 min. The mixture was purified by silica gel chromatography using 0-10% MeOH/DCM. Lyophilization gave **18** (187 mg, 0.251 mmol, 84% Yield) as an off-white solid. 1H NMR (400 MHz, DMSO-d6) δ 11.92 (d, J = 2.7 Hz, 1H), 8.46 (t, J = 5.7 Hz, 1H), 8.07 (d, J = 2.5 Hz, 1H), 7.92 (s, 1H), 7.75 (s, 1H), 7.61 (ddd, J = 12.1, 8.2, 2.5 Hz, 1H), 7.31 – 7.24 (m, 2H), 5.94 (s, 1H), 5.16 (s, 1H), 4.61 (s, 1H), 4.26 (s, 1H), 3.63 (s, 3H), 3.61 – 3.57 (m, 2H), 3.56 – 3.45 (m, 16H), 3.37 (dd, J = 5.6, 4.4 Hz, 2H), 2.90 (s, 3H), 1.25 (d, J = 6.9 Hz, 1H). LCMS (ESI): *m/z* 745 $(M+H)^+$.

Synthetic scheme for preparation of compound 19 (BETi P6)

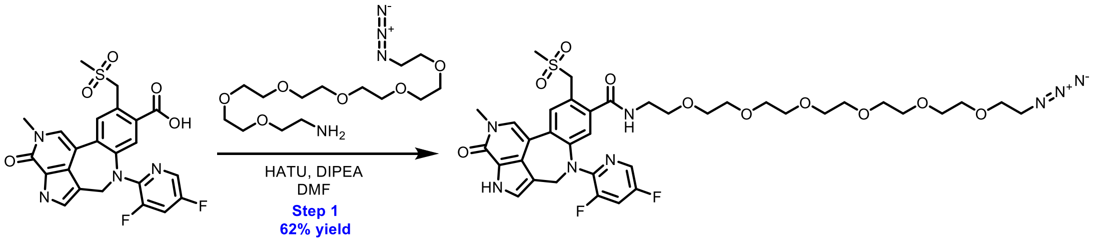

17 19

Experimental Procedure:

1. Preparation of compound 19 (BETi P6)

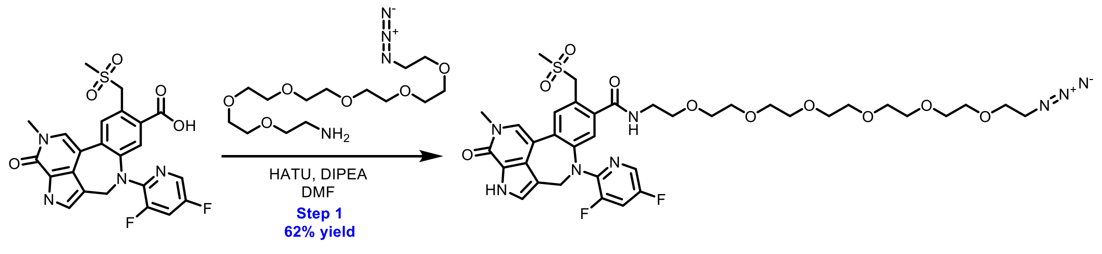

17 19

A mixture of compound **17** (150 mg, 0.299 mmol), 20-azido-3,6,9,12,15,18-hexaoxaicosan-1-amine (175.0 mg, 0.449 mmol, 1.5 equiv.), HATU (141.0 mg, 0.359 mmol, 1.2 equiv.) and DIPEA (105 µL, 0.599 mmol, 2 equiv.) in DMF (1.5 mL, 0.2 M) was stirred at room temperature for 4h. The mixture was purified by silica gel chromatography using 0-10% MeOH/DCM. Lyophilization gave **19** (155 mg, 0.186 mmol, 62% yield) as a yellow solid. 1H NMR (400 MHz, DMSO-d6) δ 11.92 (d, J = 2.8 Hz, 1H), 8.46 (t, J = 5.7 Hz, 1H), 8.07 (d, J = 2.5 Hz, 1H), 7.92 (s, 1H), 7.75 (s, 1H), 7.61 (ddd, J = 12.5, 8.3, 2.6 Hz, 1H), 7.31 – 7.24 (m, 2H), 6.06 – 5.83 (m, 1H), 5.16 (s, 1H), 4.59 (s, 1H), 4.26 (s, 1H), 3.63 (d, J = 3.5 Hz, 3H), 3.59 (dd, J = 5.6, 4.3 Hz, 2H), 3.56 – 3.45 (m, 24H), 3.41 – 3.35 (m, 2H), 2.91 (d, J = 9.5 Hz, 3H). LCMS (ESI): *m/z* 833 $(M+H)^+$.

Synthetic scheme for compound 22 (BETi C7P6)

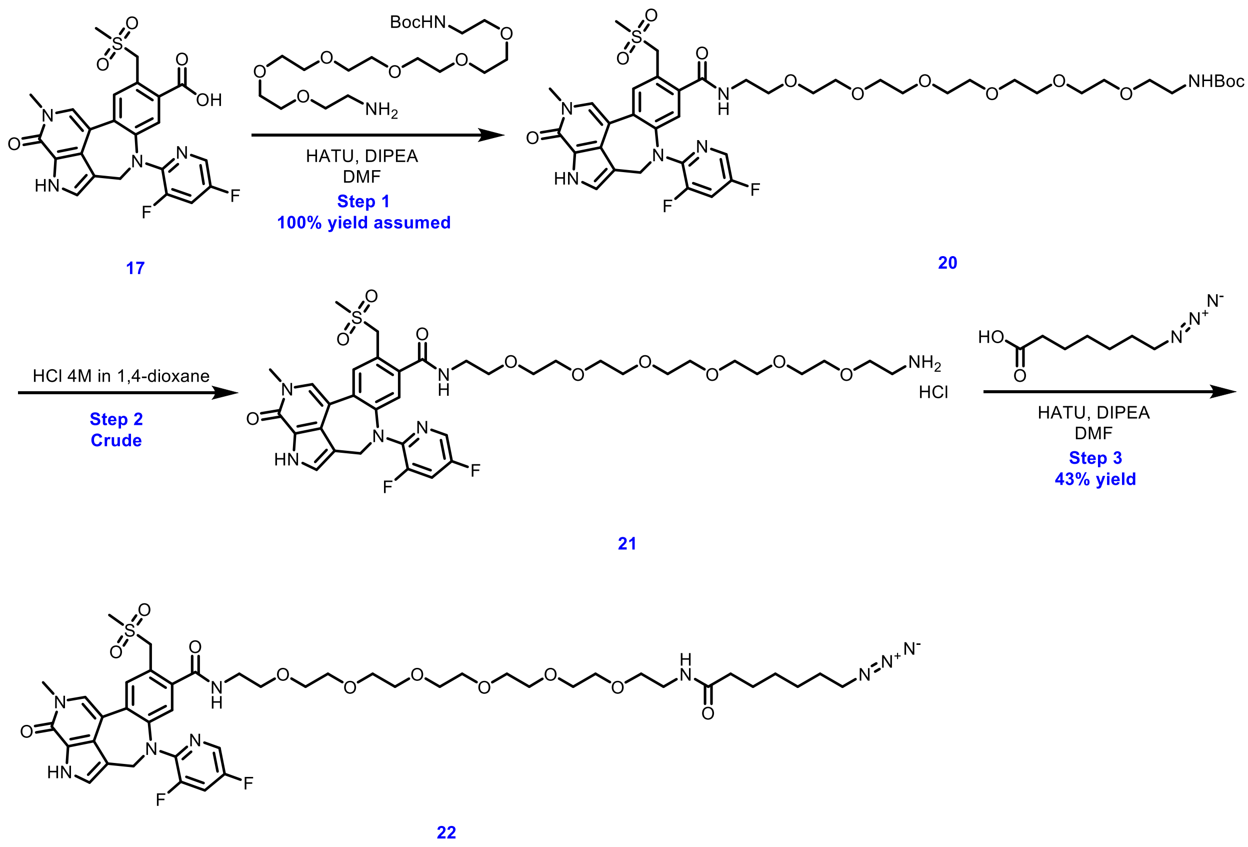

Experimental Procedure:

1. Preparation of compound 20

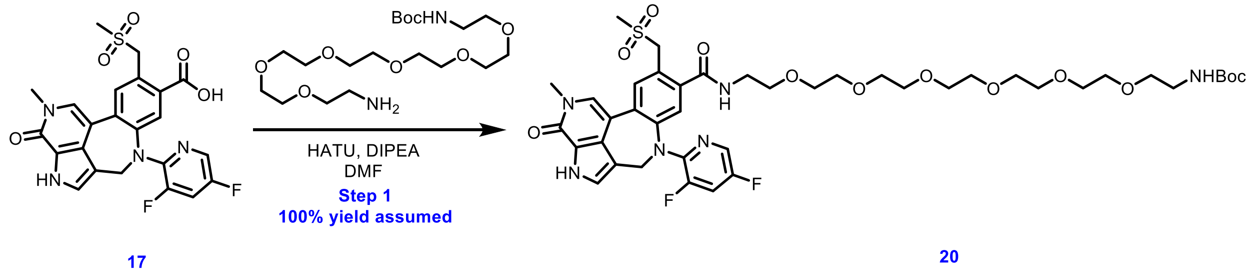

A mixture of compound **17** (400 mg, 0.799 mmol), Boc-N-amino-PEG6-amine (480 mg, 1.12 mmol, 1.4 equiv.), HATU (407 mg, 1.04 mmol, 1.3 equiv.) and DIPEA (279 µL, 1.60 mmol, 2 equiv.) in DMF (4.0 mL, 0.2 M) was stirred at room temperature for 20h. The reaction mixture was partitioned in water/DCM and extracted with DCM (3x). The combined organic extracts were washed with brine, dried over $MgSO_4$, filtered and concentrated. The crude mixture was

adsorbed on silica gel and purified by silica gel chromatography using 0-10% MeOH/DCM to give **20** (1.02 g, 1.12 mmol) as a yellow residue. NMR showed residual DMF. 1H NMR (400 MHz, DMSO-*d*6) d 11.91 (s, 1H), 8.46 (t, *J* = 5.6 Hz, 1H), 8.06 (d, *J* = 2.7 Hz, 1H), 7.92 (s, 1H), 7.75 (s, 1H), 7.67 – 7.54 (m, 1H), 7.30 – 7.24 (m, 2H), 6.73 (s, 2H), 3.62 (s, 3H), 3.49 (dq, *J* = 5.1, 2.4 Hz, 37H), 3.06 (t, *J* = 5.9 Hz, 3H), 2.90 (s, 4H), 2.69 (s, 2H), 1.37 (d, *J* = 3.6 Hz, 17H), 1.24 (s, 12H). LCMS (ESI): *m/z* 907 $(M+H)^+$.

2. *Preparation of Compound 21*

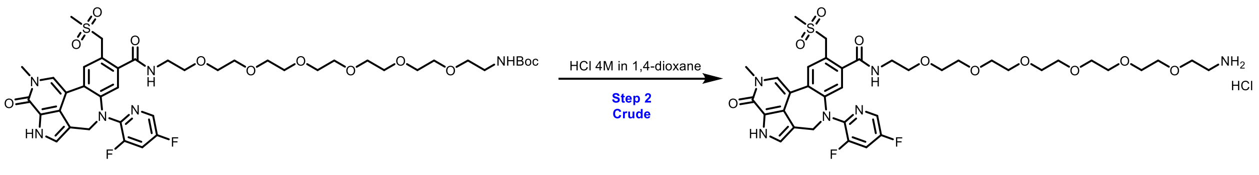

20 21

Compound **20** (725 mg, 0.799 mmol) was stirred with HCl (4 M in 1,4-dioxane) (4.0 mL, 16.0 mmol, 0.2 M) at room temperature for 4h. The reaction mixture was concentrated to dryness and diluted with toluene and concentrated (2x). Crude product **21** was carried over to the next step without purification.

3. *Preparation of compound 22 (BETi C7P6)*

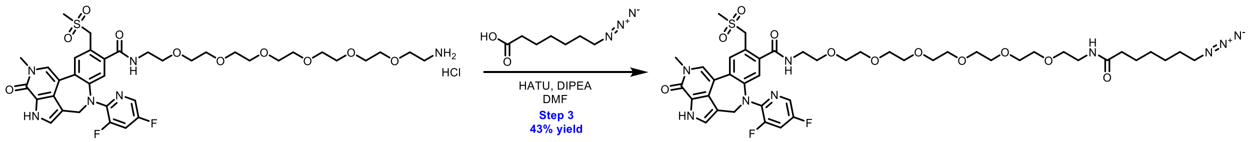

21 22

A mixture of compound **21** (337 mg, 0.399 mmol), 7-azidoheptanoic acid (103 mg, 0.599 mmol, 1.5 equiv.), HATU (313 mg, 0.799 mmol, 2 equiv.) and DIPEA (698 µL, 3.99 mmol, 10 equiv.) in DMF (2.0 mL, 0.2 M) was stirred at room temperature for 3h. The crude product was adsorbed on silica gel and purified by silica gel chromatography using 0-10% MeOH/DCM. The compound was further purified by prep-HPLC (20-60 CAN/0.1% NH_4OH in water, XSelect CSH Prep C18, 50 x 30mm, 5µm, run time: 10 min) to afford compound **22** (164 mg, 0.171 mmol, 43% Yield) as a

yellow residue. 1H NMR (400 MHz, DMSO-d6) δ 11.91 (d, J = 2.8 Hz, 1H), 8.46 (t, J = 5.6 Hz, 1H), 8.07 (d, J = 2.5 Hz, 1H), 7.92 (s, 1H), 7.80 (t, J = 5.7 Hz, 1H), 7.75 (s, 1H), 7.61 (ddd, J = 12.3, 8.3, 2.5 Hz, 1H), 7.29 (s, 1H), 7.27 (d, J = 2.7 Hz, 1H), 5.95 (s, 1H), 5.16 (s, 1H), 4.61 (s, 1H), 4.26 (s, 1H), 3.62 (s, 3H), 3.49 (d, J = 2.5 Hz, 22H), 3.42 – 3.33 (m, 4H), 3.28 (d, J = 6.8 Hz, 2H), 3.17 (q, J = 5.9 Hz, 2H), 2.90 (s, 3H), 2.05 (t, J = 7.4 Hz, 2H), 1.49 (tq, J = 14.4, 7.4, 7.0 Hz, 4H), 1.35 – 1.17 (m, 4H). LCMS (ESI): *m/z* 960 (M+H)⁺.

Synthetic scheme for preparation of compound 23 (BETi P4P6)

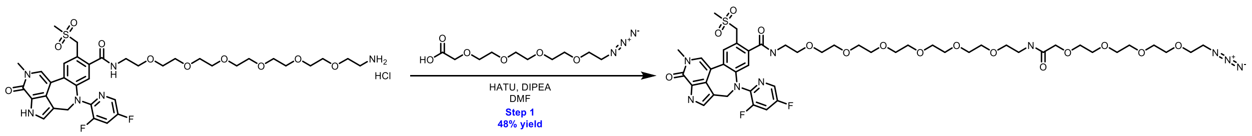

1. Preparation of compound 23 (BETi P4P6)

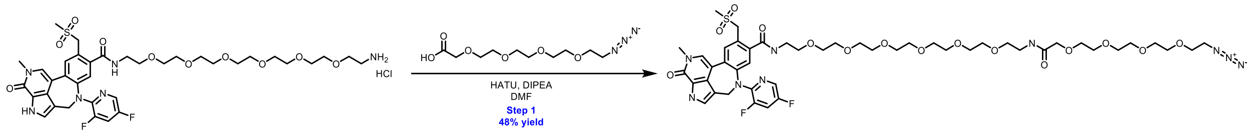

Compound **21** (337 mg, 0.399 mmol), 2-[2-[2-[2-(2-azidoethoxy)ethoxy]ethoxy]ethoxy]acetic acid 0.5M in MTBE (1 mL, 0.599 mmol, 1.5 equiv.), HATU (313 mg, 0.799 mmol, 2 equiv.) and DIPEA (698 µL, 3.99 mmol, 10 equiv.) in DMF (2.0 mL, 0.2 M) was stirred at room temperature for 3h. The crude product was adsorbed on silica gel and purified by silica gel chromatography using 0-10% MeOH/DCM. The compound was further purified by prep-HPLC (20-60 CAN/0.1% Ammonium hydroxide in water, XSelect CSH Prep C18, 50 x 30mm, 5µm, run time: 10 min) to afford compound **23** (199 mg, 0.187 mmol, 48% Yield) as a yellow residue. 1H NMR (400 MHz, DMSO-d6) δ 11.92 (s, 1H), 8.46 (t, J = 5.6 Hz, 1H), 8.07 (d, J = 2.5 Hz, 1H), 7.92 (s, 1H), 7.75 (s, 1H), 7.66 – 7.56 (m, 2H), 7.29 (s, 1H), 7.27 (s, 1H), 5.94 (s, 1H), 5.16 (s, 1H), 4.61 (s, 1H), 4.26 (s, 1H), 3.87 (s, 2H), 3.62 (s, 3H), 3.61 – 3.57 (m, 2H), 3.57 – 3.52 (m, 11H), 3.49 (d, J = 3.2 Hz, 21H), 3.46 – 3.34 (m, 8H), 3.27 (d, J = 6.7 Hz, 2H), 2.90 (s, 3H). LCMS (ESI): *m/z* 1066 (M+H)⁺.

1. General Experimental Procedure for Synthesis of BETcides

II. Peptide Synthesis

All peptides were synthesized using standard Fmoc (9-fluorenylmethoxycarbonyl) solid-phase peptide synthesis (SPPS) [1]. A combination of both manual and automated synthesis was employed where appropriate. Automated microwave-assisted SPPS was performed on a Liberty Blue™ Automated Microwave Peptide Synthesizer (CEM) using a 0.5 mmol-scale protocol. Rink amide ProTide™ resin (loading 0.52 mmol/g) was used as the solid support, and DIC/ OxymaPure® as the activating system. In the coupling steps, a solution of an Fmoc-protected amino acid (0.4 M, 10 mL) in *N*,*N*-dimethylformamide (DMF) was added to the resin (0.7 g, 0.36 mmol), followed by the addition of OxymaPure® solution (1 M, 4 mL) and DIC solution (1 M, 4 mL) in DMF. The resulting mixture was purged with nitrogen under microwave irradiation at 90 °C for 2 min with the exceptions of L-and D-cysteine (75 °C, 2 min), and L- and D-histidine (50 °C, 10 min). The supernatant was drained, and the process was repeated once for all amino acids except L- and D-histidine. The supernatant was drained, and the resin was washed with DMF (20 mL × 3). For Fmoc removal, a 20% solution of 4-methylpiperidine in DMF (10 mL) was added to the resin. The resulting mixture was purged with nitrogen under microwave irradiation at 90 °C for 1 min. The supernatant was drained, and the process was repeated once. The resin was then washed thoroughly with DMF (10 mL × 5). The coupling-deprotection cycle was repeated until the peptide assembly was complete.

The coupling of Fmoc-Ser(propargyl)-OH was done manually. A solution of Fmoc-Ser(Propargyl)-OH (0.4 g, 1.08 mmol, 3.0 equiv) in DMF was added to the resin (0.7 g, 0.36 mmol, 1.0 equiv), followed by the addition of 2-(1*H*-7-azabenzotriazol-1-yl)-1,1,3,3-tetramethyl uronium hexafluorophosphate (HATU, 411 mg, 1.08 mmol, 3 equiv) and *N*,*N*-diisopropylethylamine (DIEA, 0.188 mL, 1.08 mmol, 3 equiv). The resulting mixture was agitated at room temperature overnight. Ninhydrin test indicated completion of reaction. The supernatant was drained, and the resin was washed with DMF (20 mL × 3).

For the synthesis of thioether-cyclic peptides, the N-terminus of the peptide was capped with chloroacetyl by agitating the resin (0.7 g, 0.36 mmol, 1.0 equiv) with a solution of chloroacetic anhydride (0.65 g, 3.6 mmol, 10.0 equiv) and DIEA (0.63 mL, 3.6 mmol, 10.0 equiv) in 20 mL DMF at room temperature overnight. Ninhydrin test indicated complete reaction. The supernatant was drained, and the resin was washed with DMF (20 mL × 3).

For peptide cleavage, the resin (0.7 g, 0.36 mmol) was washed thoroughly with (DMF ,15 mL × 3) and dichloromethane (DCM, 20 mL × 3), and then dried in vacuo. A mixture of trifluoroacetic acid/phenol/water/thioanisole/triisopropylsilane (v/w/v/v/v 90:2.5:1.5:3.5:2.5, 15 mL) was added to the resin at 0 °C. The resulting mixture was slowly warmed to room temperature and allowed to mix for 3 h. The supernatant was collected by filtration, and the resin was washed with the cleavage mixture (8 mL × 3). The combined filtrate was concentrated under reduced pressure to a small volume, which was diluted with anhydrous diethyl ether (90 mL). The resulting mixture was allowed to stand at 0 °C for 30 min and then centrifuged. The precipitate was washed with anhydrous diethyl ether (90 mL × 3) and dried in vacuo to afford the crude linear peptide as a white powder. Crude yield 45-58%.

For cyclization via a thioether bond, the crude linear peptide was dissolved in 50% acetonitrile (ACN) in water at a concentration of 10 mg/mL, and the pH was adjusted to 8 – 9 by dropwise addition of DIEA. The reaction was stirred overnight and was monitored with liquid chromatography-mass spectrometry (LC-MS). Upon completion of cyclization, the solution was lyophilized to dryness. The obtained crude cyclic peptides were purified by reverse-phase high-performance chromatography (RP-HPLC, C18 column, 21 mm × 250 mm, 5 µm, 100 Å, 10 mL/min, 60-min gradient from 30 to 60% ACN/water containing 0.1% TFA). Purification yield 10-15%.

General Synthetic Scheme for Microwave assisted and Manual SPPS

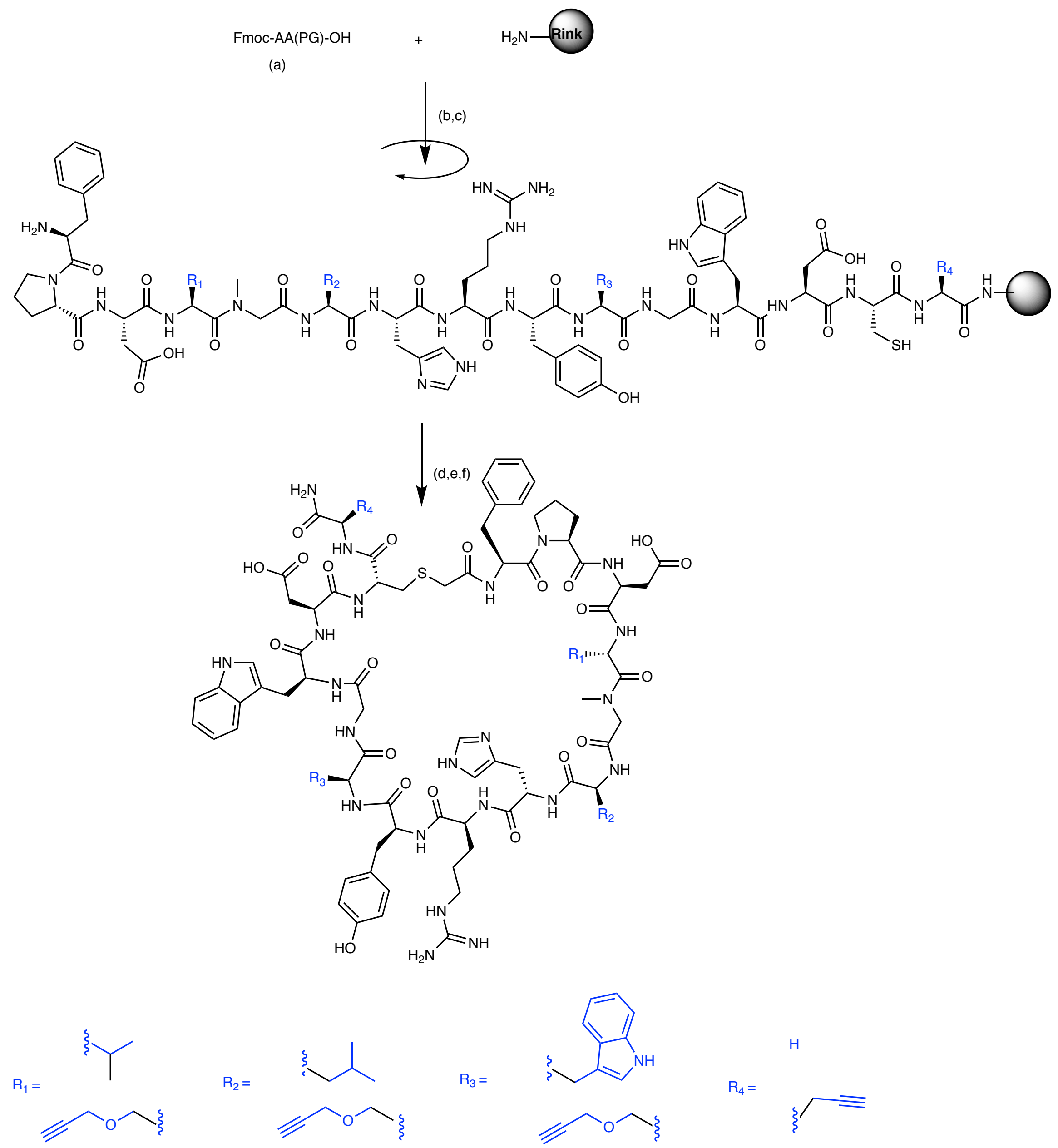

Peptide Sequence modified accordingly with alkyne structures represented above at the Gly, Val, Leu and Trp positions: cyc(Ac) FPDV(Sar)LHRYWGWDCG amide

Scheme for SPPS: a) 0.25 mmol Fmoc amino acid/DMF b) 1M DIC/ 1M Oxyma pure activating reagent/ DMF c) 20% Piperidine /DMF d) Chlroacetyl anhydride/DMF, DIEA e) TFA cocktail f) DIEA

Table (1) Representation of Macrocyclic Peptide Alkynes

Side chain / Compound Number	R1	R2	R3	R4	Yield %
24	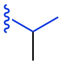	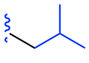	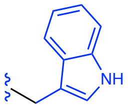	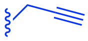	15
25	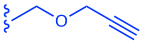	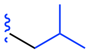	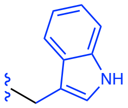	H	12
26	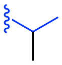	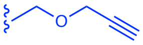	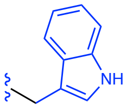	H	14
27	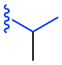	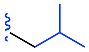	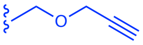	H	15
28D*	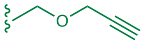	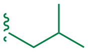	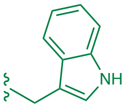	H	10

D* = D isomer macrocyclic peptide, where all amino acids in the macrocycle are Fmco-D-AA-OH

Conjugation of Peptides to BRD4 Ligands

The alkyne-bearing peptides were conjugated to BRD4 ligands containing an azido group on a polyethylene glycol (PEG) linker via click chemistry. In a typical procedure, the alkyne-bearing peptide (40 mg, 1.0 equiv) was dissolved in degassed DMF (3 mL) under nitrogen atmosphere, and the pH was adjusted to 8 with DIEA by adding excess DIEA. Tris(hydroxypropyltriazolylmethyl)amine

(THPTA) ligand (10.0 equiv) was added. The resulting mixture was agitated. A stochiometric amount of tetrakis(acetonitrile)copper(I) hexafluorophosphate (Cu (I)) (3.0 equiv) was added to the solution under nitrogen atmosphere, followed by addition of the azide-derivatized BRD4 ligand (4.0 equiv). The resulting mixture was purged with nitrogen at room temperature overnight, and the reaction was monitored by LC-MS. Upon completion of reaction, Chelex 100 resin (1.0 g) was added. The resulting mixture was agitated for 5 min and then filtered. The

filtrate was used directly for RP-HPLC purification (C18 column, 21 mm × 250 mm, 5 µm, 100 Å, 10 mL/min, 60-min gradient from 30 to 60% ACN/water containing 0.1% TFA). BETCIDEs were obtained as white powder with purification yields 10-20%.

Fluorescent labeling of peptides with Cyanine 5.5

The fluorescently labeled PSMD2 Macrocyclic peptide PSMD2-MC1$_{Cy5.5,}$ was obtained following the click chemistry protocol under similar reaction conditions as described above for BRD4 conjugation. The PSMD2-MC1$_{Cy5.5}$ was obtained as a blue powder with purification yield of 23%. The control compound triazole capped Cyanine5.5 (**55**) dye was commercially sourced from Lumiprobe Corporation.

General Synthetic Scheme for C-Terminal Conjugation Compounds (28 to 31) (BETcide1-4 X*)

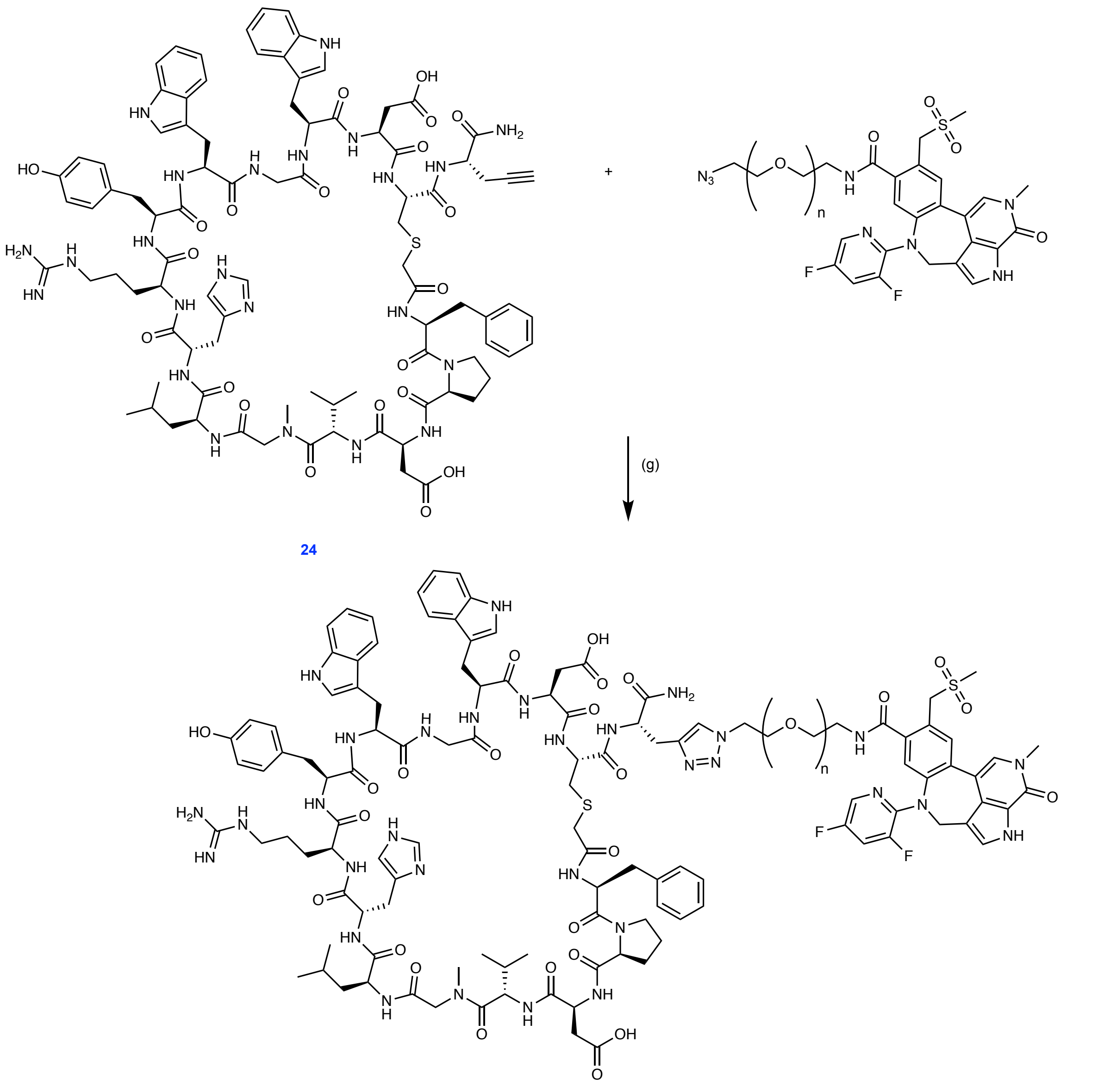

24

28 to 31 = BETcide1- 4 X*

Scheme for Conjugation: g) THPTA, Cu(I), DIEA, DMF

*X represents linkers P4, P6, C7P6, P4P6 refer to table.

General Synthetic Scheme for on-the-ring Macrocycle Conjugation, Compounds (32 to 35) (BETcide5 -8 X*)

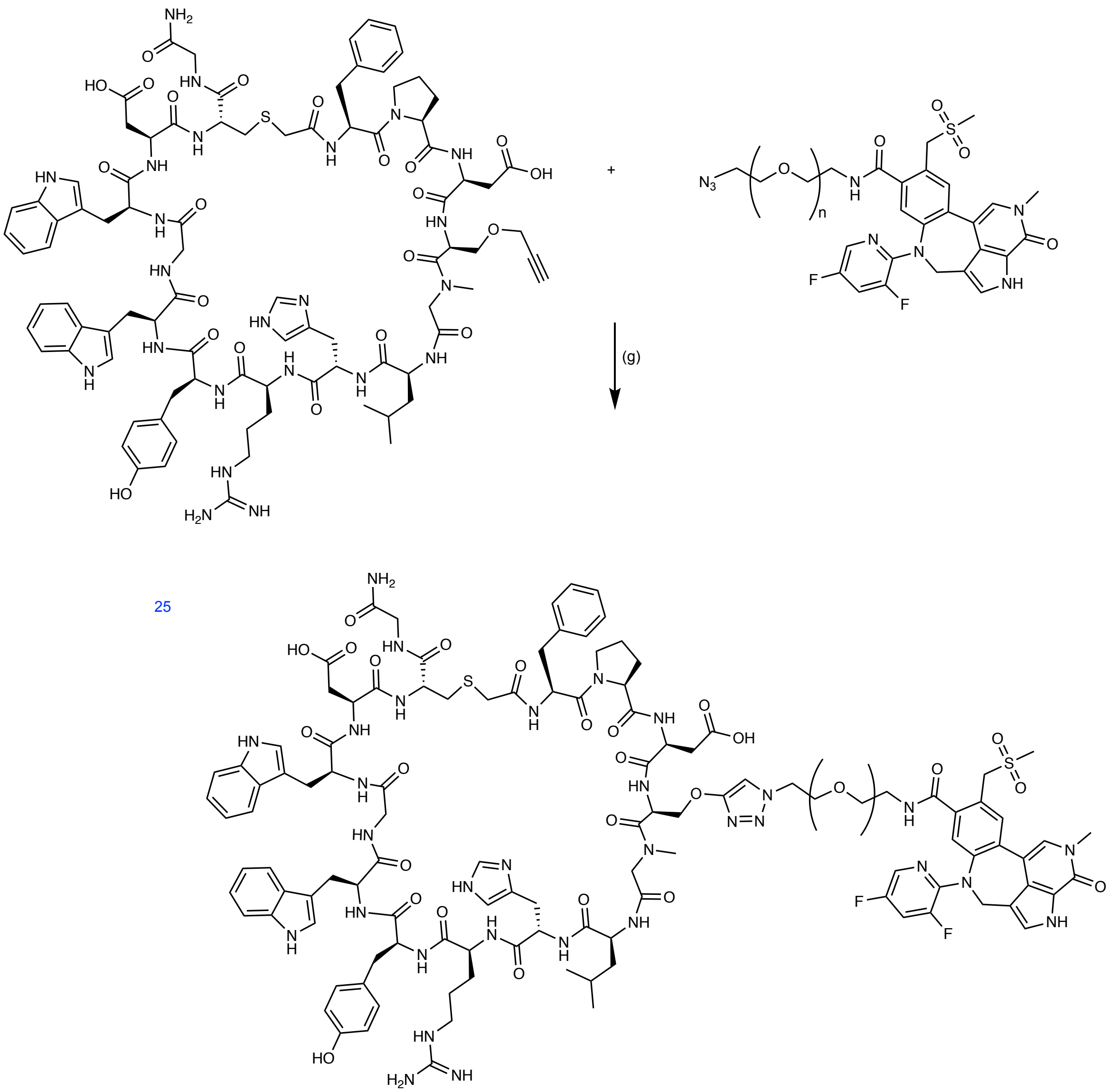

25

32

Scheme for Conjugation: g) THPTA, Cu(I), DIEA, DMF

Table (2) Representation of BETcide Peptide Macrocyclic conjugates with their Respective Abbreviations of Compound Names and Numbers

PSMD2-MC1 - derived CIDEs used in this study						
Compound name/ or Number	Yeild %	Nickname	Compound type	Conjugation point	X= Linker*	amino acid stereochemistry
(28) BETcide1 G15-P4	18	G15-P4	CIDE	Gly15	P4	L
(29) BETcide2 G15-P6	20	G15-P6	CIDE	Gly15	P6	L
(30) BETcide3 G15-C7P6	10	G15-C7P6	CIDE	Gly15	C7P6	L
(31) BETcide4 G15-P4P6	15	G15-P4P6	CIDE	Gly15	P4P6	L
(32) BETcide5 V4-P4	20	V4-P4	CIDE	Val4	P4	L
(33) BETcide6 V4-C7P6	10	V4-C7P6	CIDE	Val4	C7P6	L
(34) BETcide7 V4-P4P6	16	V4-P4P6	CIDE	Val4	P4P6	L
(35) BETcide8 V4-P6	17	L-CIDE	CIDE	Val4	P6	L
(36) BETcide9 L6-P4	19	L6-P4	CIDE	Leu6	P4	L
(37) BETcide10 L6-C7P6	12	L6-C7P6	CIDE	Leu6	C7P6	L
(38) BETcide11 L6-P4P6	12	L6-P4P6	CIDE	Leu6	P4P6	L
(39) BETcide12 L6-P6	18	L6-P6	CIDE	Leu6	P6	L
(40) BETcide13 W10-P4	20	W10-P4	CIDE	Trp10	P4	L
(41) BETcide14 W10-C7P6	14	W10-C7P6	CIDE	Trp10	C7P6	L
(42) BETcide15 W10-P4P6	18	W10-P4P6	CIDE	Trp10	P4P6	L
(43) BETcide16 W10-P6	15	W10-P6	CIDE	Trp10	P6	L
(44) D-BETcide8 V4-P6	20	D-CIDE	CIDE	val4	P6	D
*P4 = PEG4, P6 = PEG6, C7P6= 7alkyl-PEG6, P4P6 = PEG4+PEG6						

1. Chemical Structures and LC-MS Data for BRD4 Conjugates

LC-MS data for compound (28) BETcide1 G15-P4 - G03428399

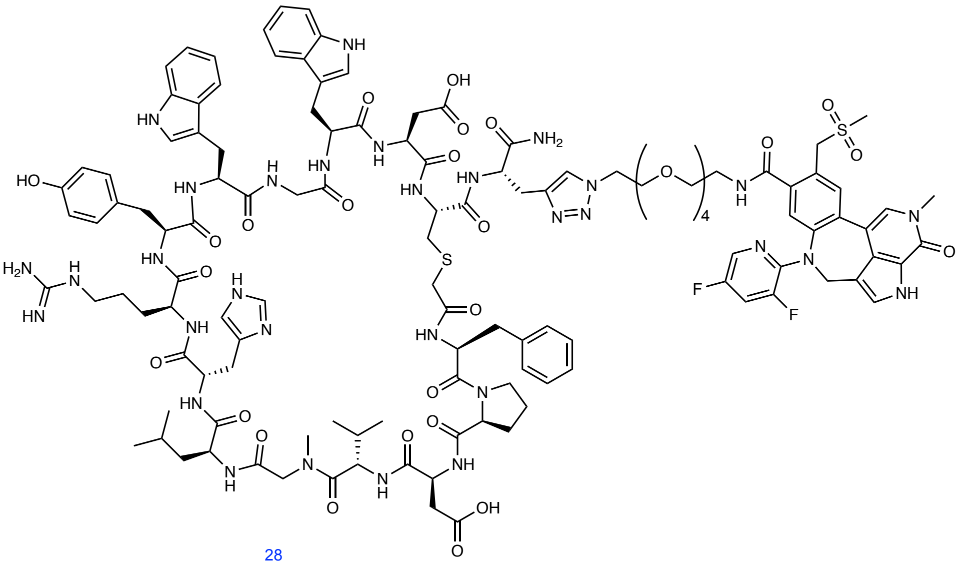

28

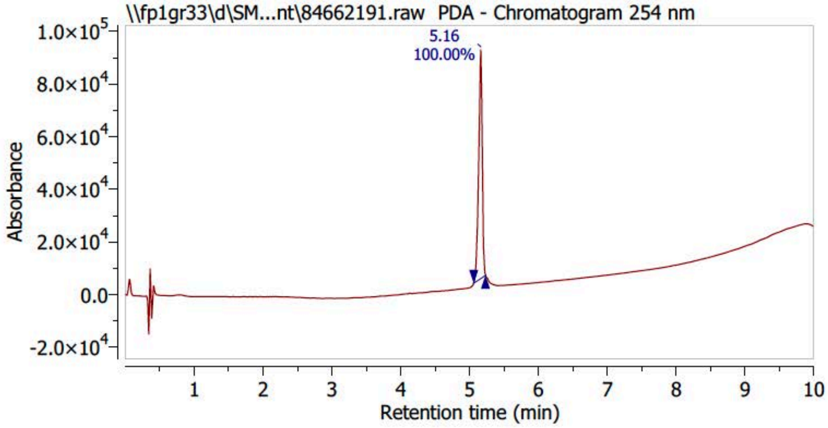

DAD, Sig=254 (>=1.5%)

RT	Area	Area %	MS+
5.16	1540910.00	100.00	930.40

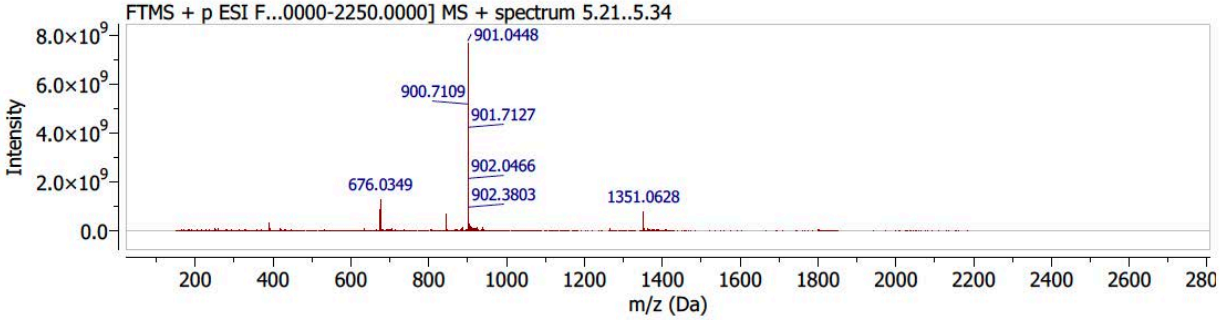

LC-MS data for compound (29) BETcide2 G15-P6 - G03428400

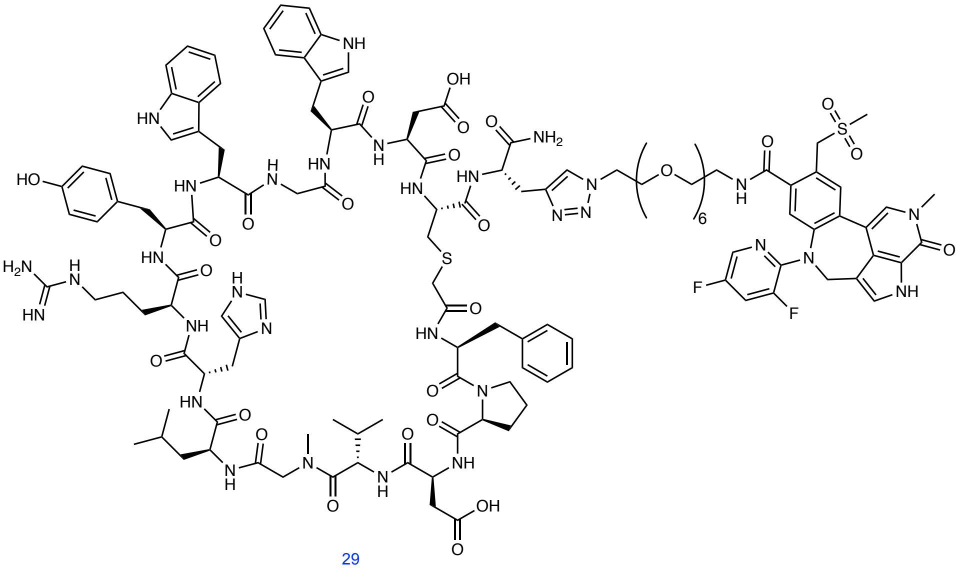

29

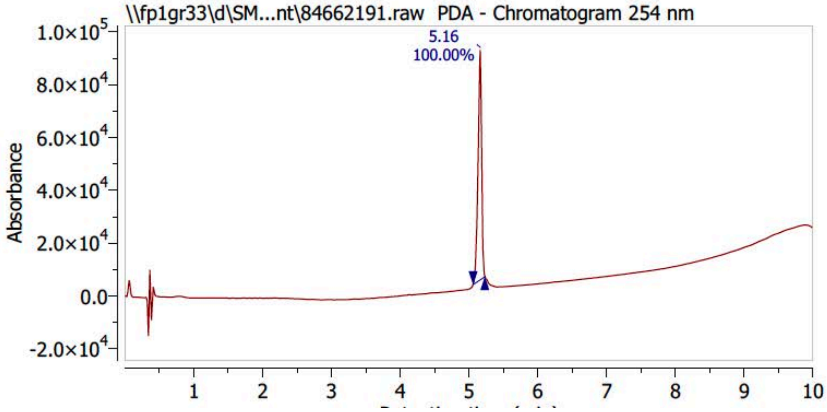

DAD, Sig=254 (>=1.5%)

RT	Area	Area %	MS+
5.16	1540910.00	100.00	930.40

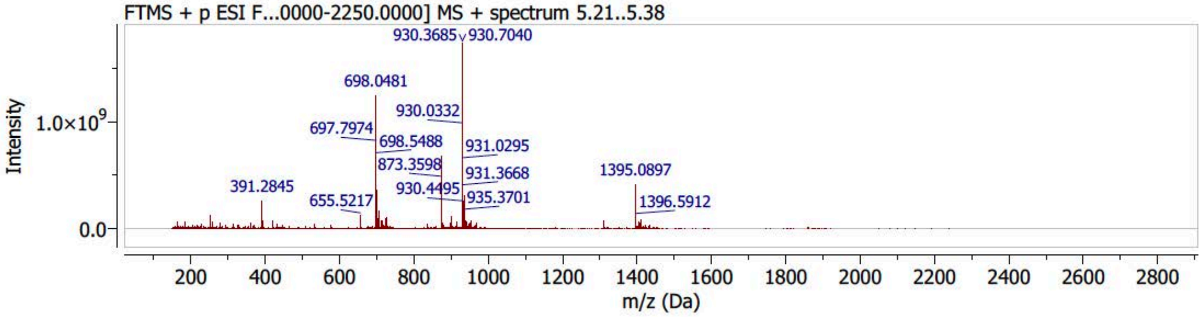

LC-MS data for compound (30) BETcide3 G15-C7P6 - G03428401

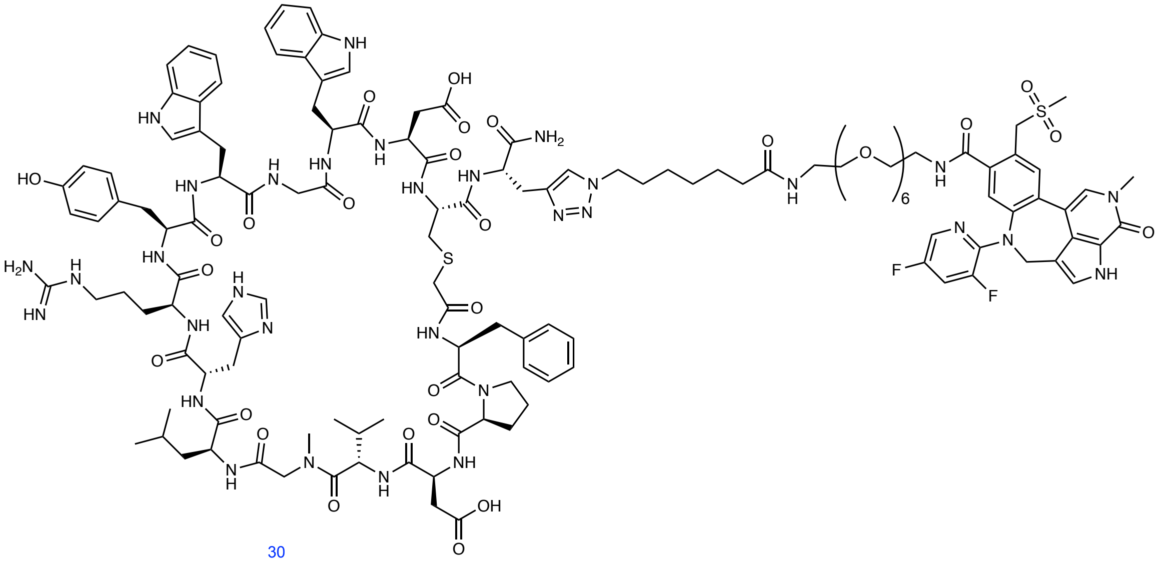

30

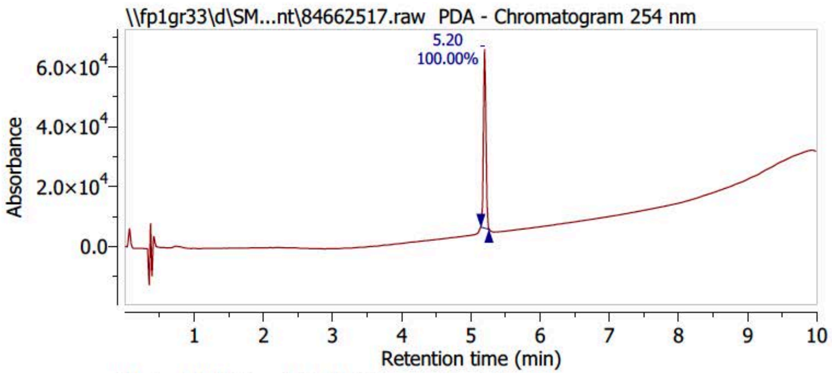

DAD, Sig=254 (>=1.5%)

RT	Area	Area %	MS+
5.20	734132.00	100.00	972.76

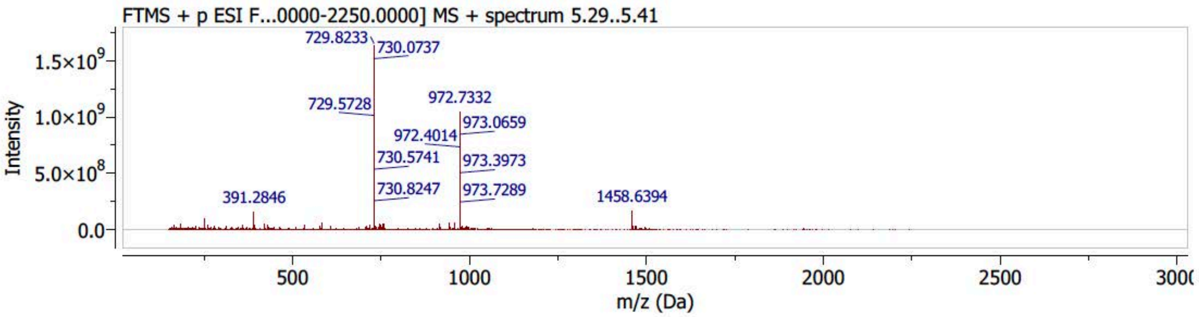

LC-MS data for compound (31) BETcide4 G15-P4P6 - G03428402

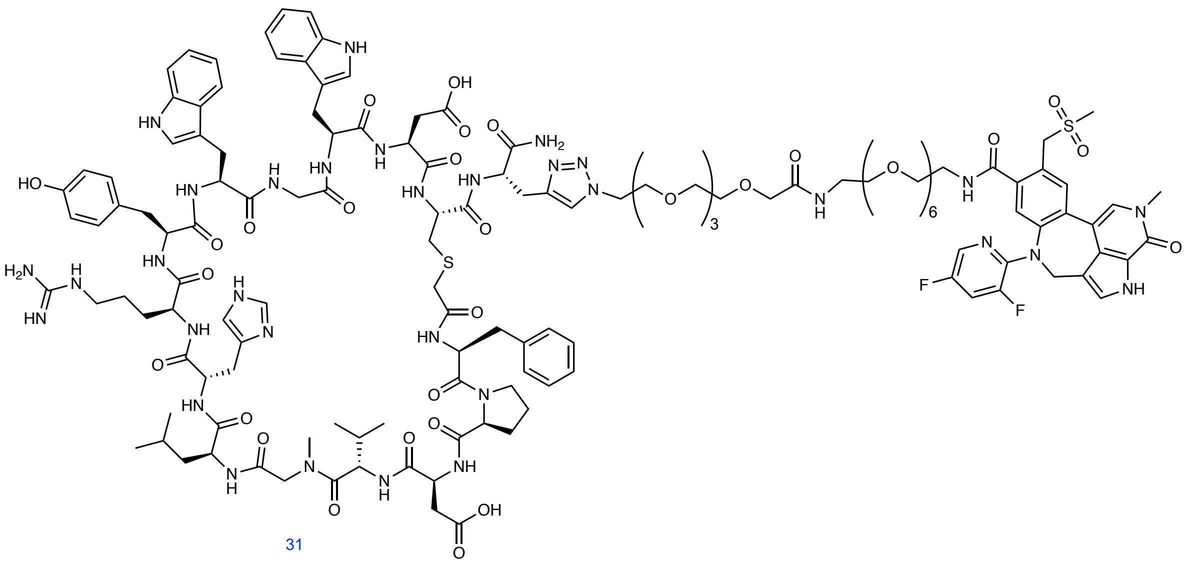

31

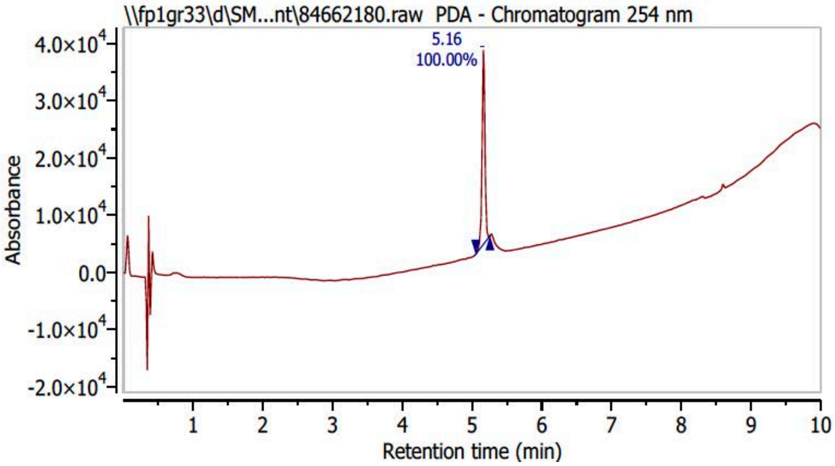

DAD, Sig=254 (>=1.5%)

RT	Area	Area %	MS+
5.16	471208.47	100.00	756.33

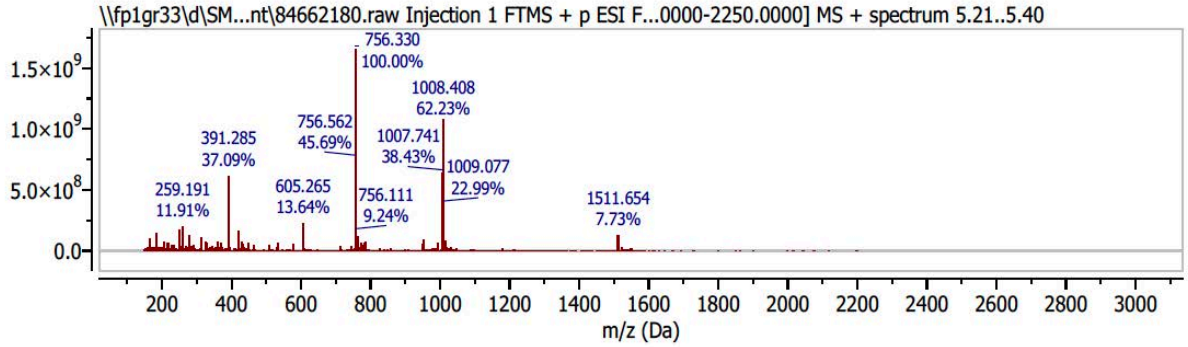

LC-MS data for compound (32) BETcide5 V4-P4 - G03409111

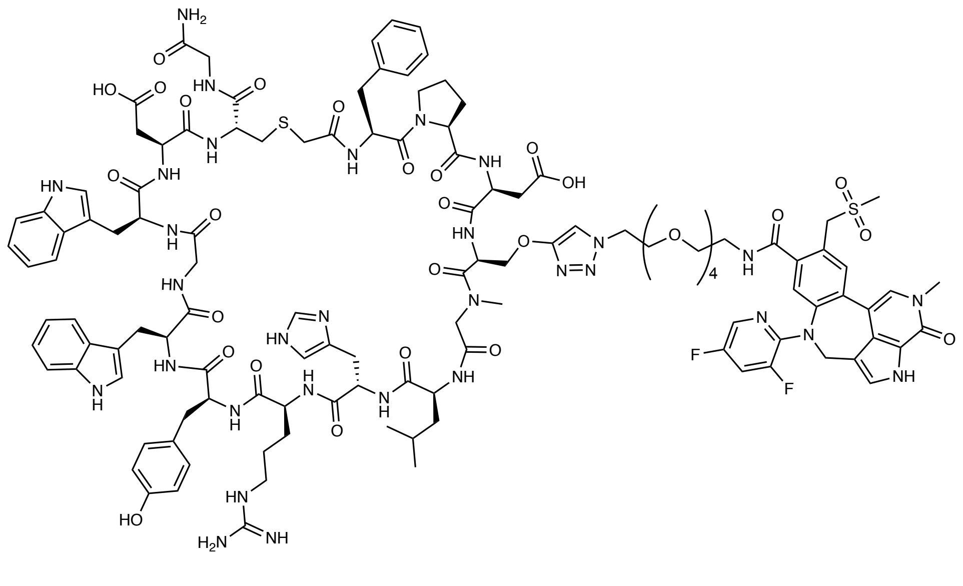

32

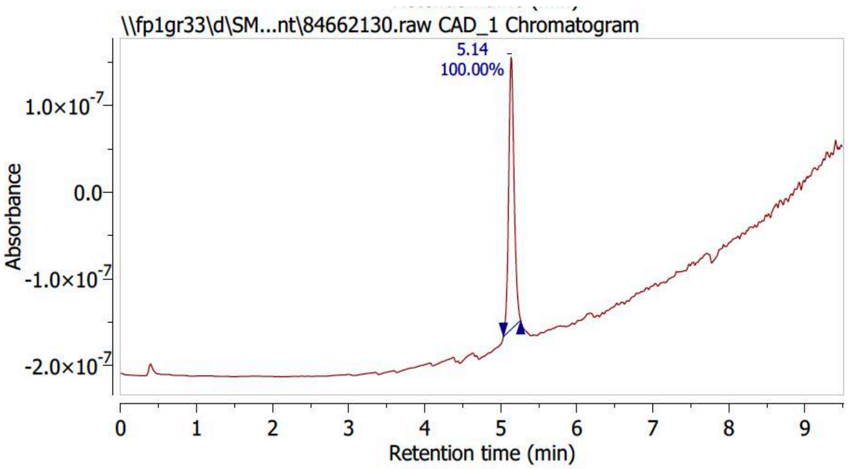

CAD_1 (>=5%)

RT	Area	Area %	MS+
5.14	30771.87	100.00	878.03

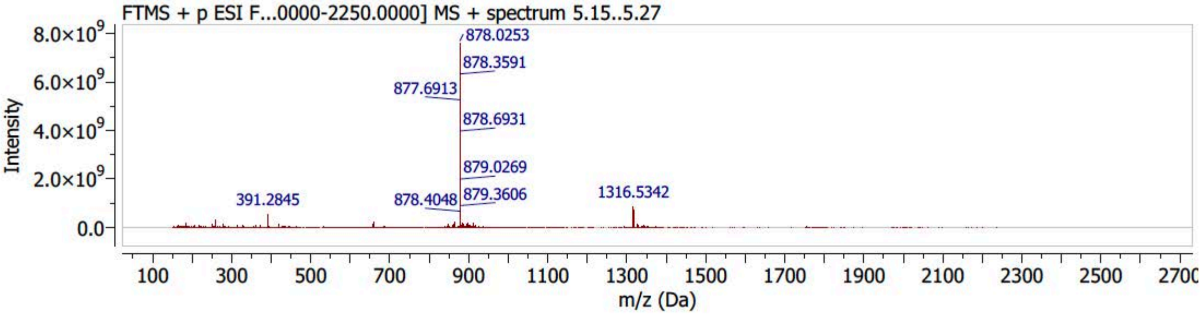

LC-MS data for compound (33) BETcide6 V4-C7P6 - G03409112

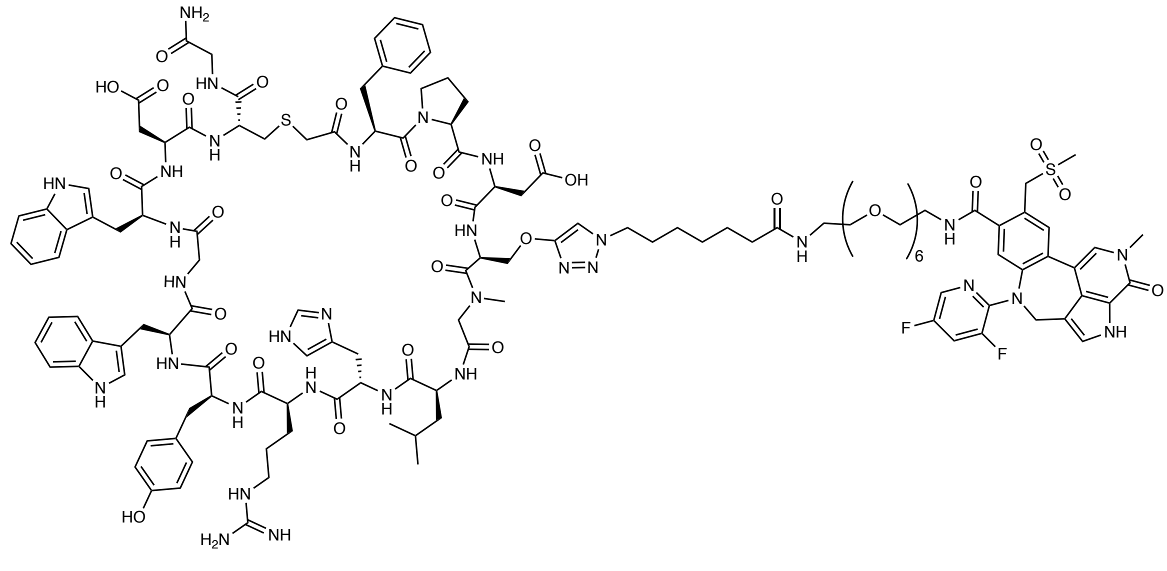

33

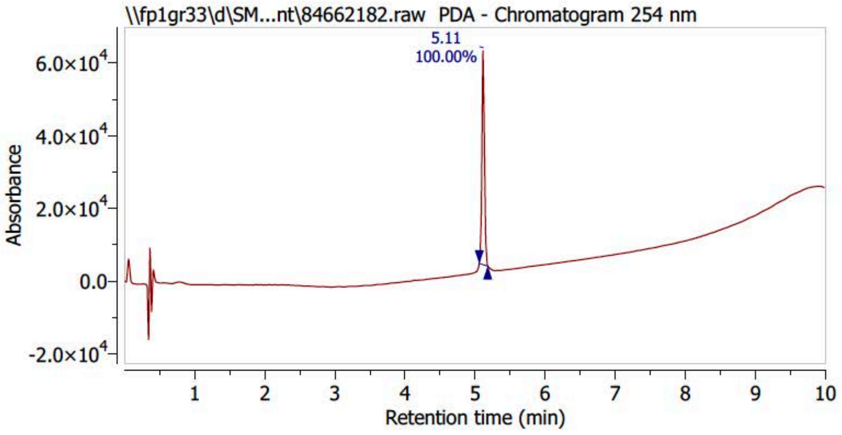

DAD, Sig=254 (>=1.5%)

RT	Area	Area %	MS+
5.11	739878.50	100.00	949.74

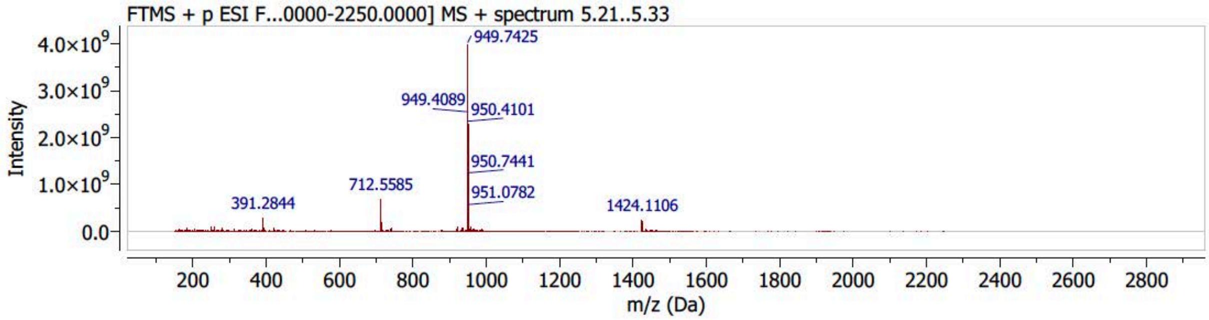

LC-MS data for compound (34) BETcide7 V4-P4P6 - G03409113

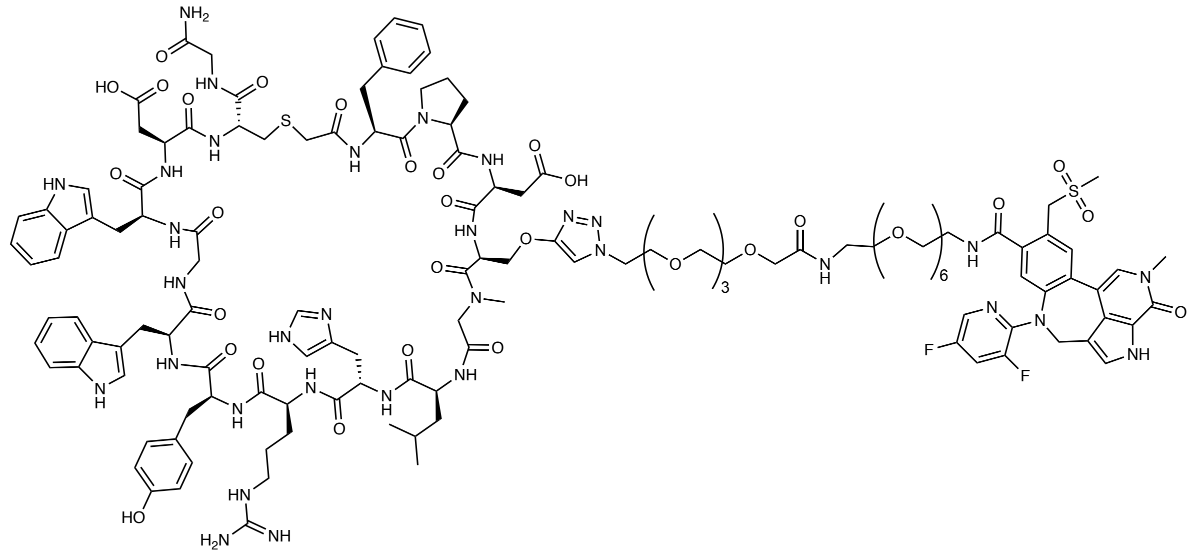

34

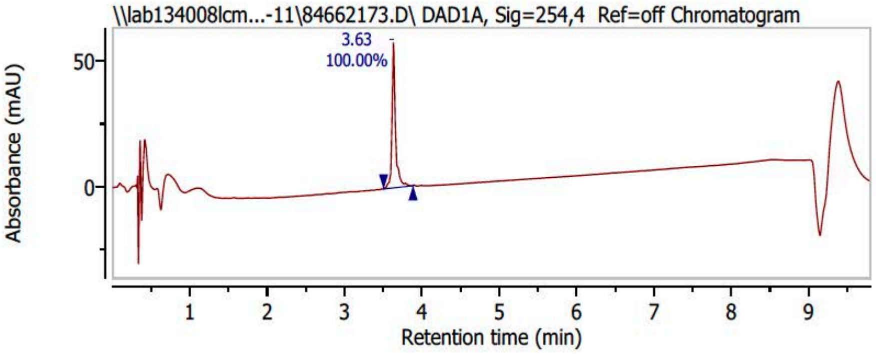

DAD1A, Sig=254,4 Ref=off (>=1.5%)

RT	Area	Area %	MS+
3.63	2271.66	100.00	985.20

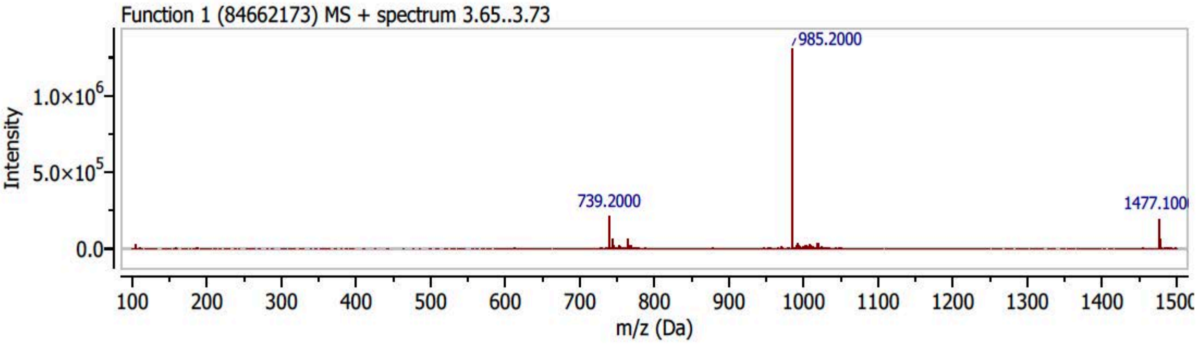

LC-MS data for compound (35) BETcide8 V4-P6 - G03409115

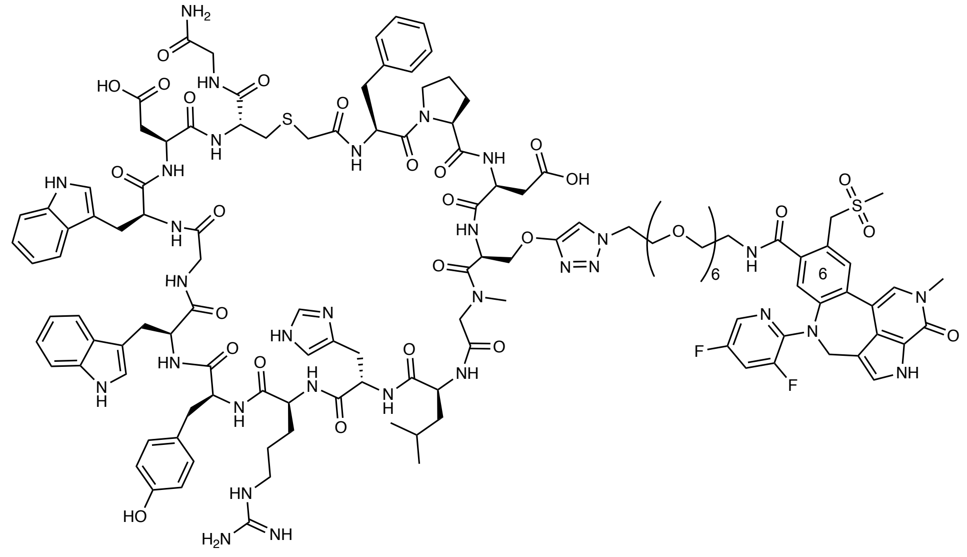

35

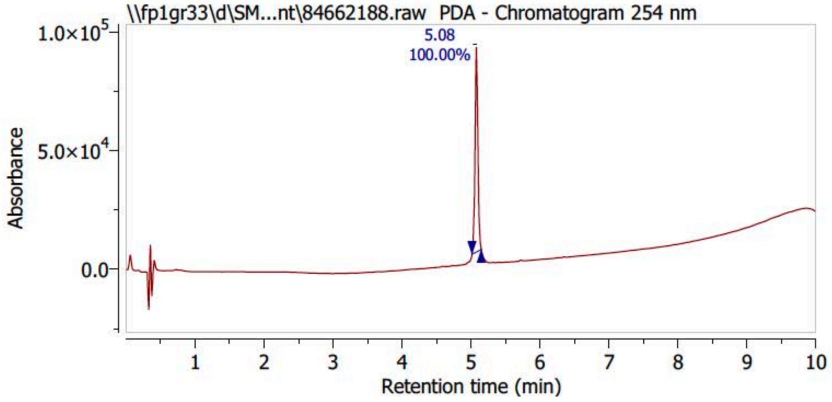

DAD, Sig=254 (>=1.5%)

RT	Area	Area %	MS+
5.08	1224960.00	100.00	907.38

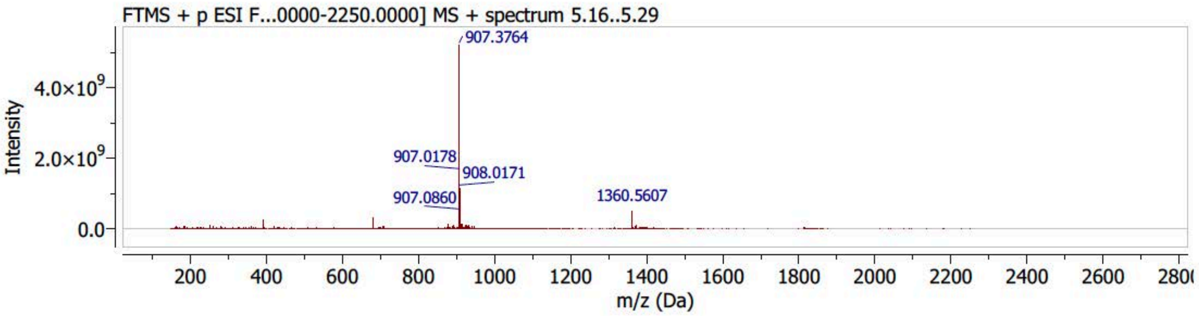

LC-MS data for compound (36) BETcide9 L6-P4 - G03409124

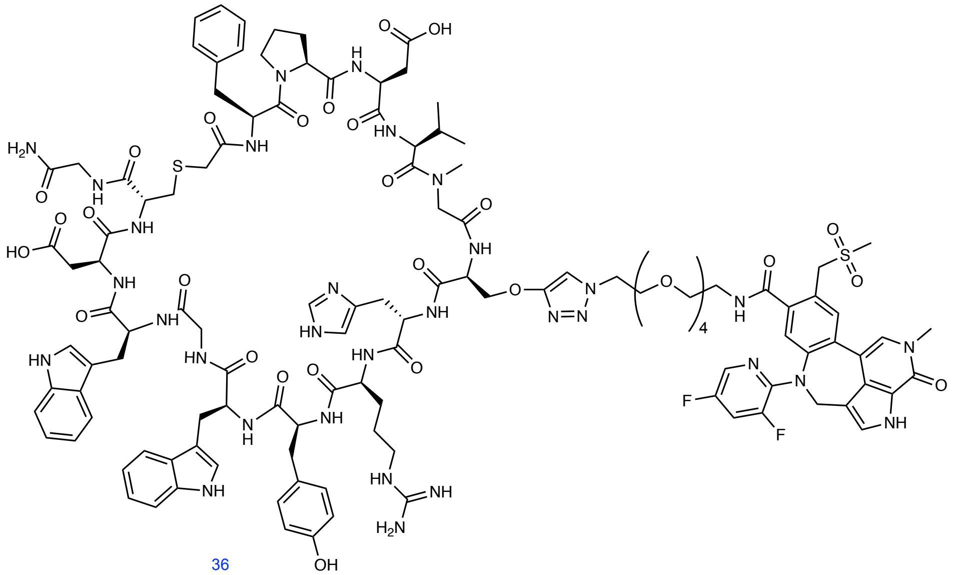

36

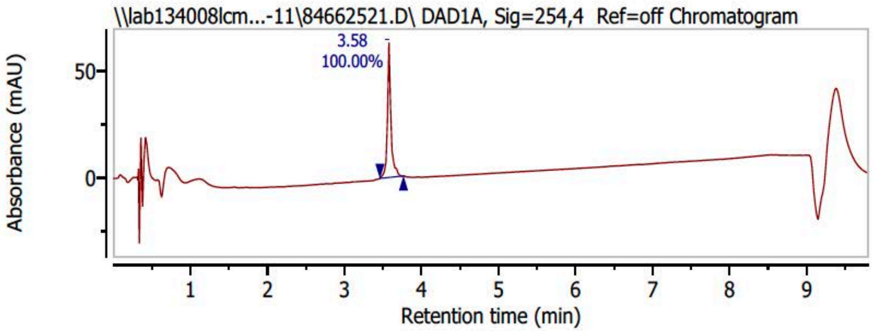

DAD1A, Sig=254,4 Ref=off (>=1.5%)

RT	Area	Area %	MS+
3.58	3956.25	100.00	873.30

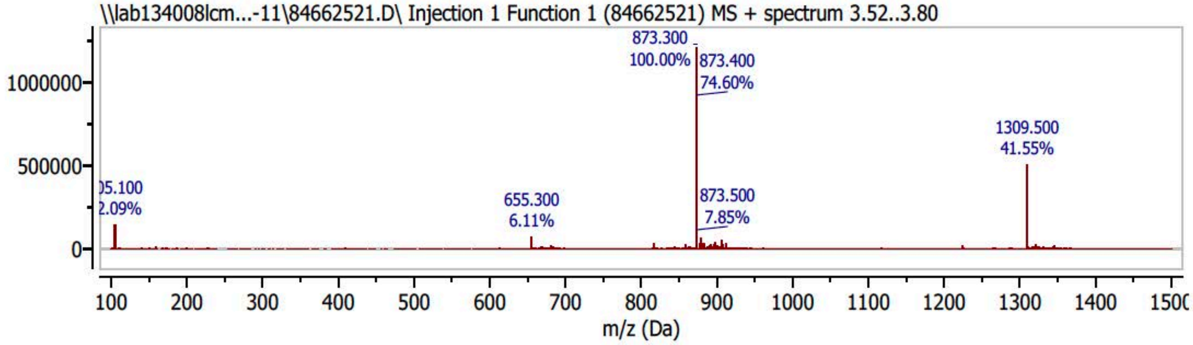

LC-MS data for compound (37) BETcide10 L6-C7P6 - G03409125

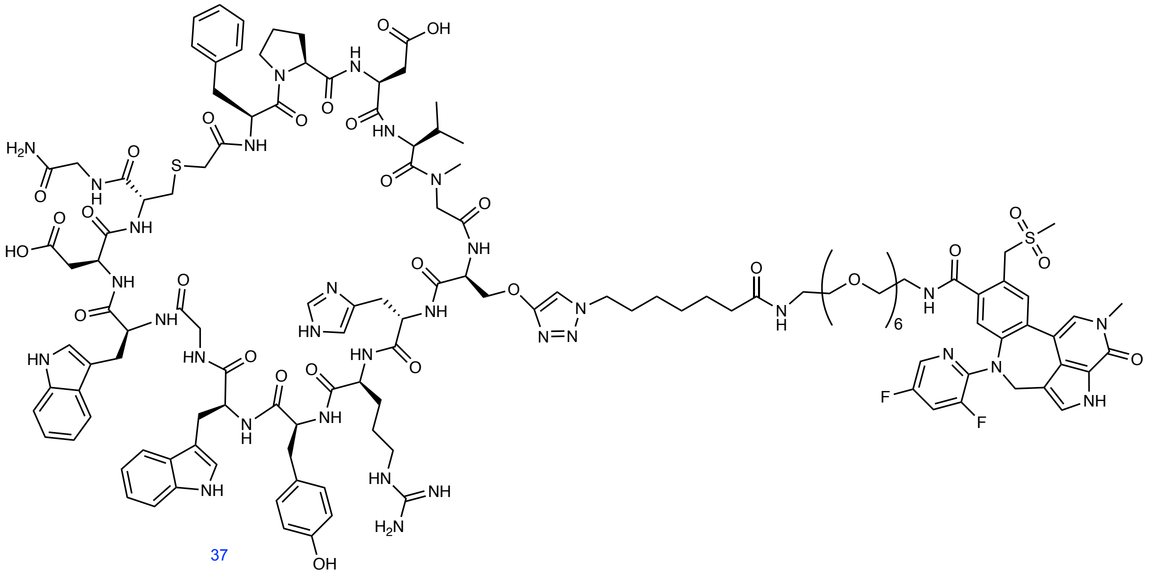

37

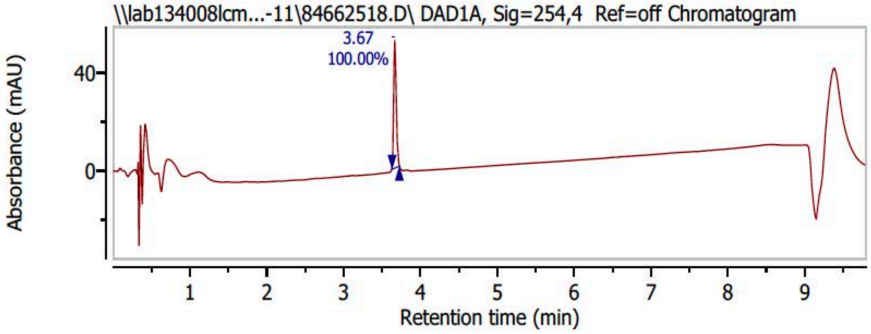

DAD1A, Sig=254,4 Ref=off (>=1.5%)

RT	Area	Area %	MS+
3.67	2355.05	100.00	945.10

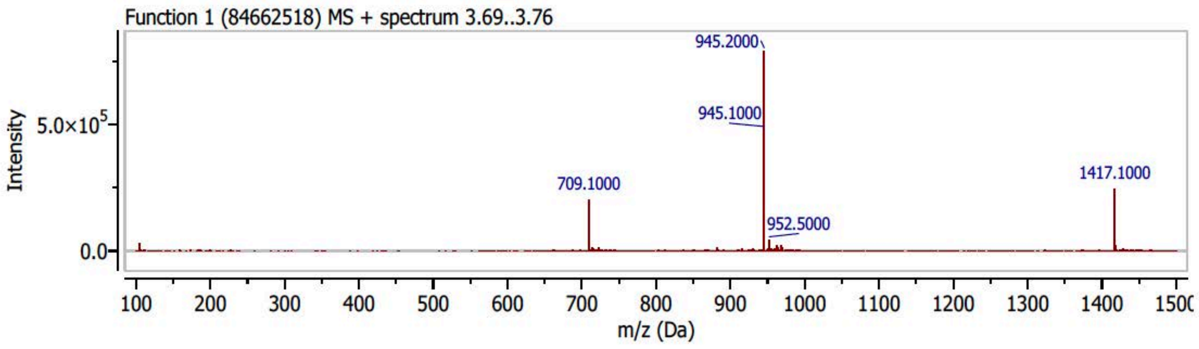

LC-MS data for compound (38) BETcide11 L6-P4P6 - G03409126

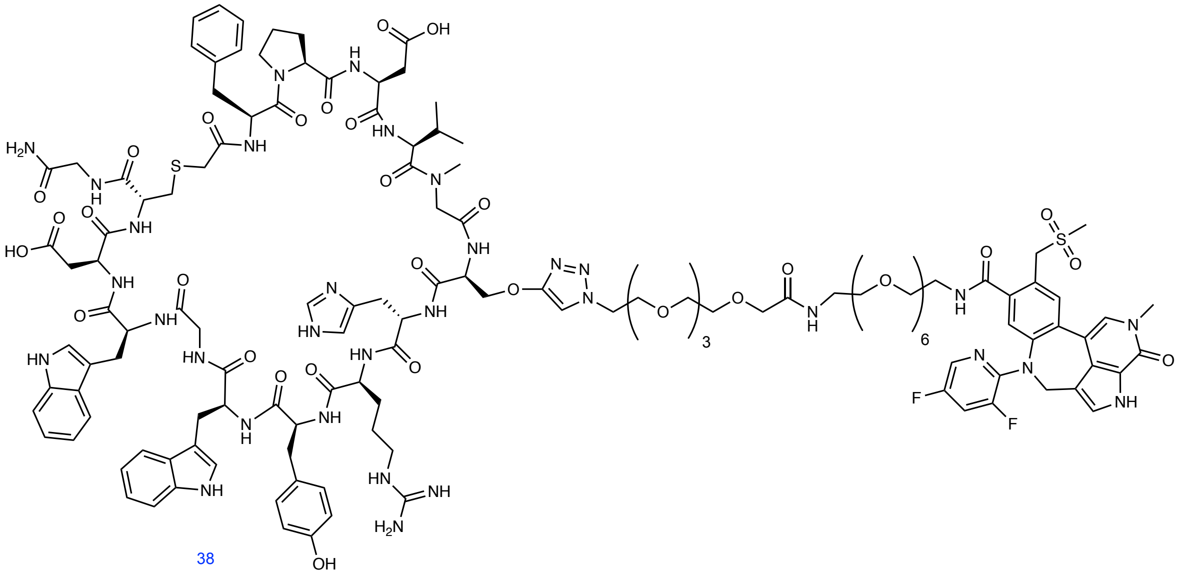

38

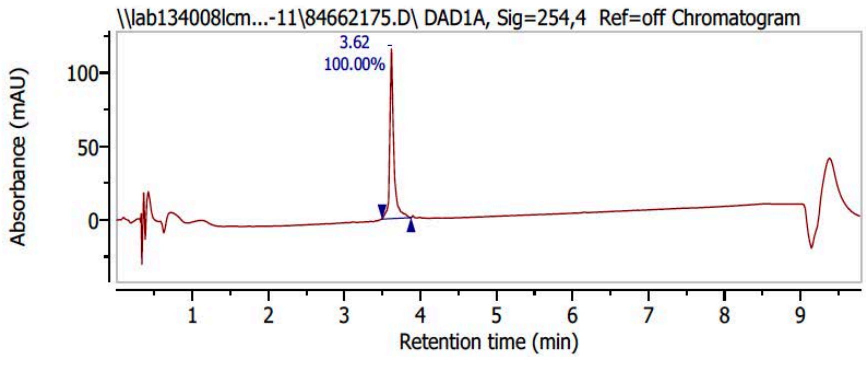

DAD1A, Sig=254,4 Ref=off (>=1.5%)

RT	Area	Area %	MS+
3.62	8293.42	100.00	980.50

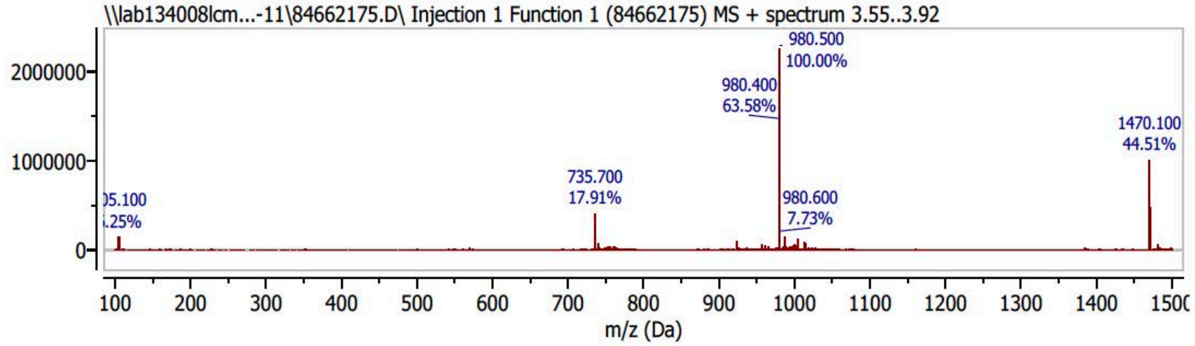

LC-MS data for compound (39) BETcide12 L6-P6 - G03409127

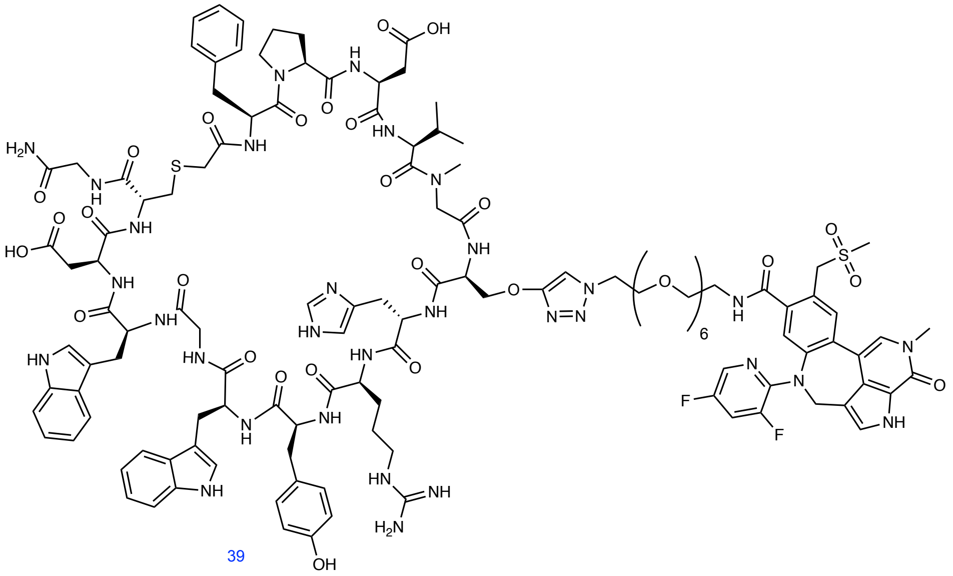

39

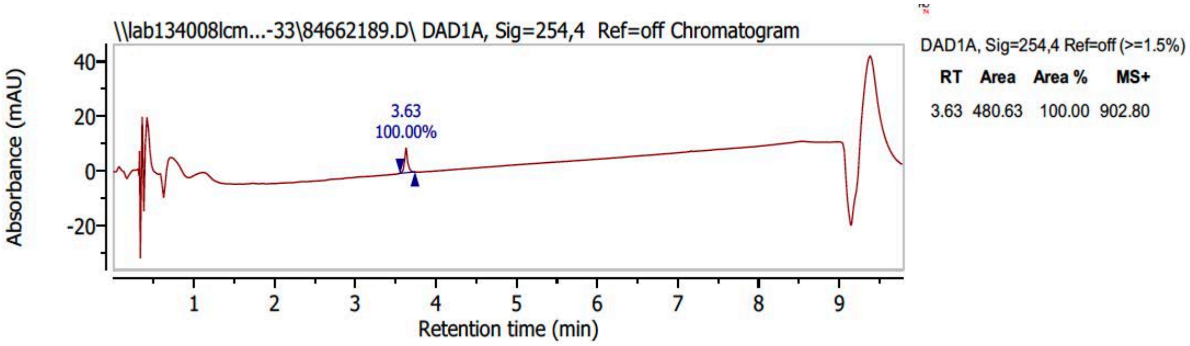

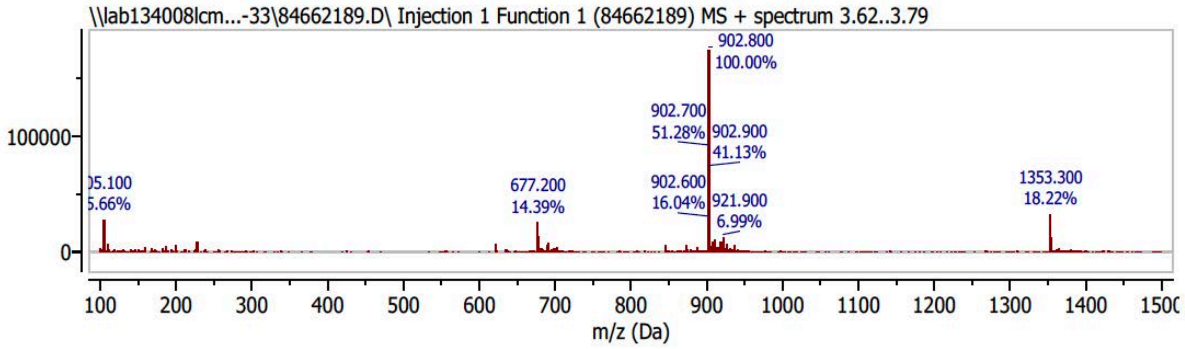

LC-MS data for compound (40) BETcide13 W10-P4 - G03409128

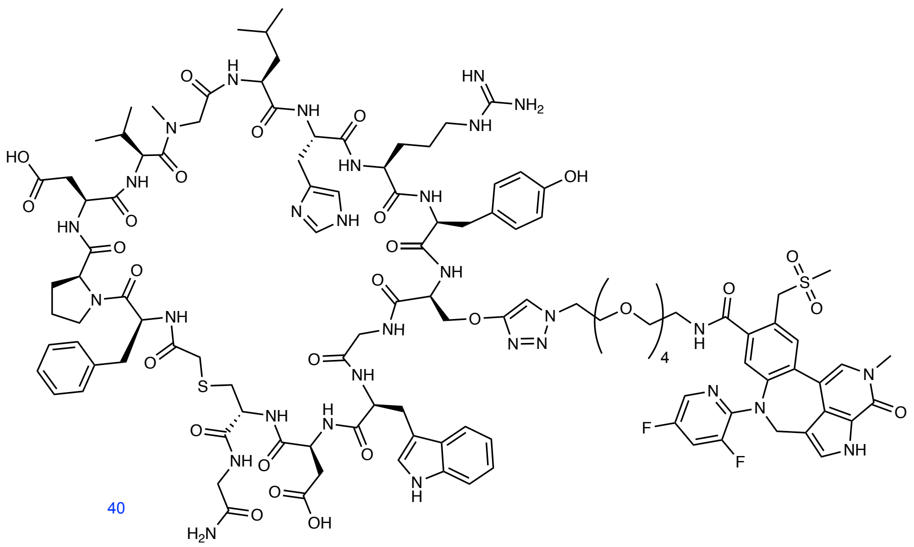

40

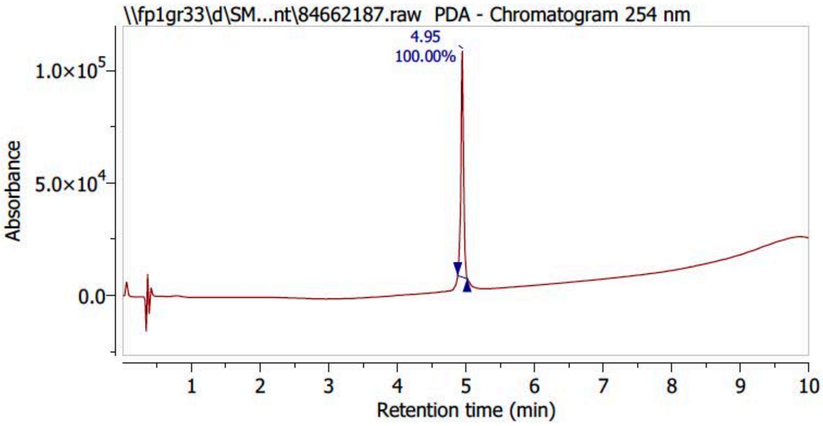

DAD, Sig=254 (>=1.5%)

RT	Area	Area %	MS+
4.95	1328994.00	100.00	849.02

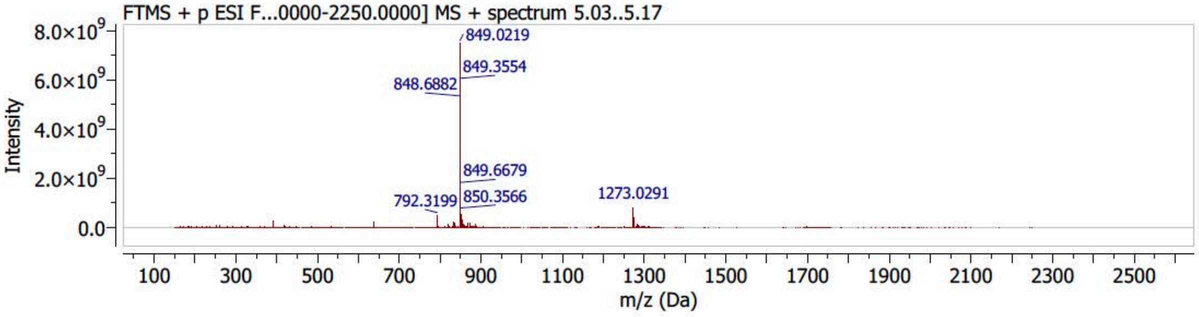

LC-MS data for compound (41) BETcide14 W10-C7P6 - G03409129

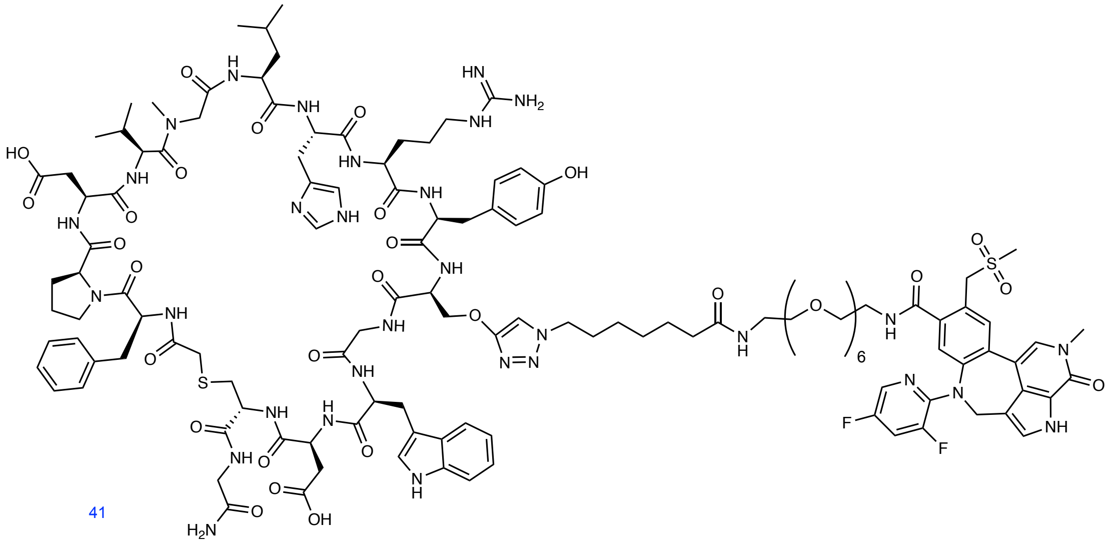

41

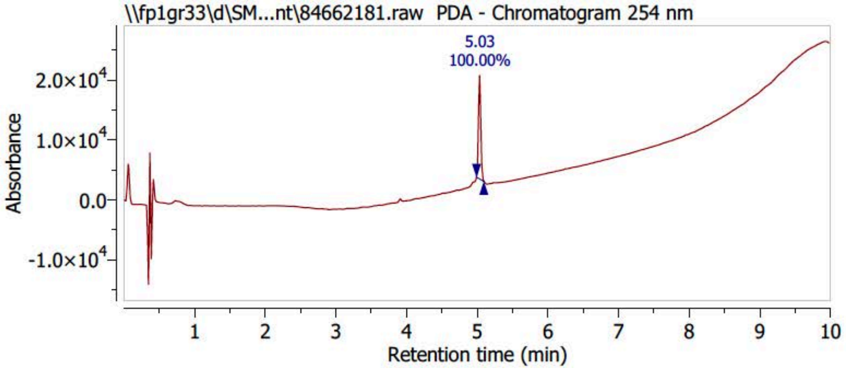

DAD, Sig=254 (>=1.5%)

RT	Area	Area %	MS+
5.03	195567.00	100.00	920.74

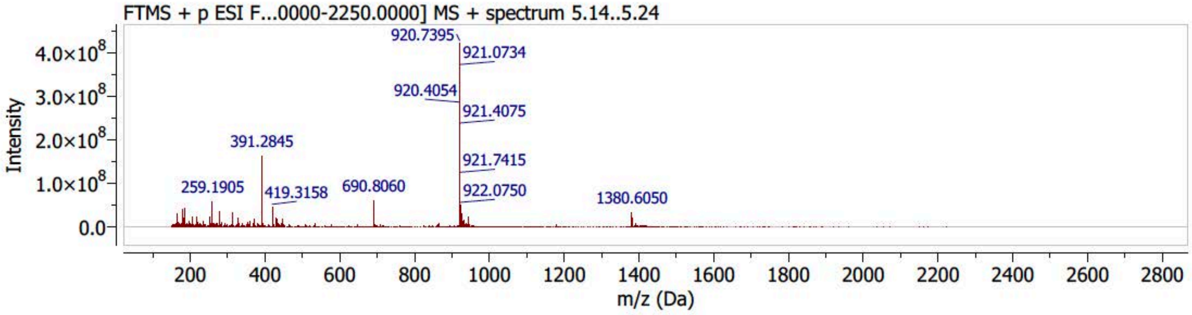

LC-MS data for compound (42) BETcide15 W10-P4P6 - G03409130

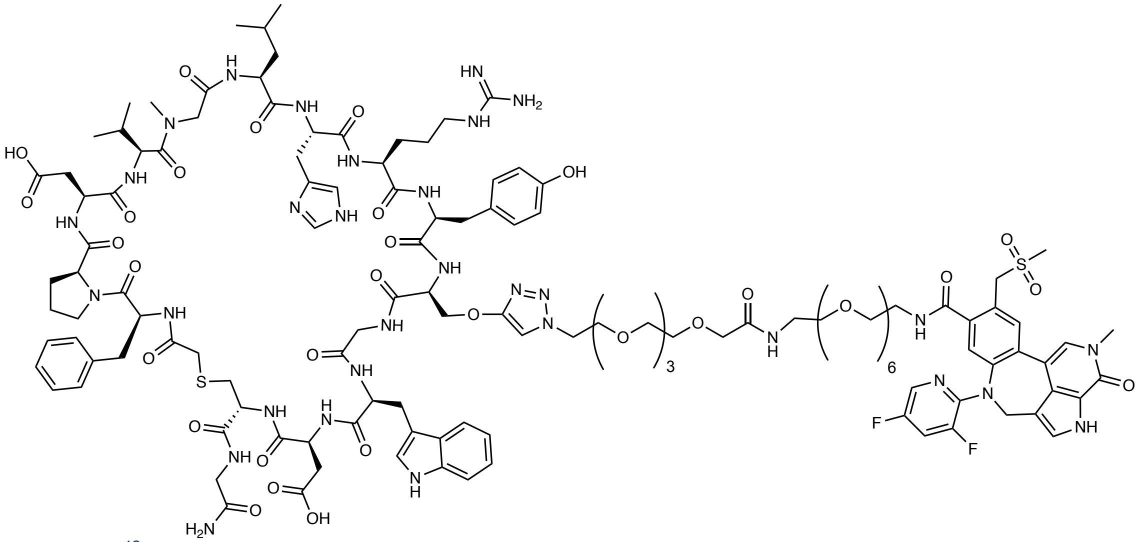

42

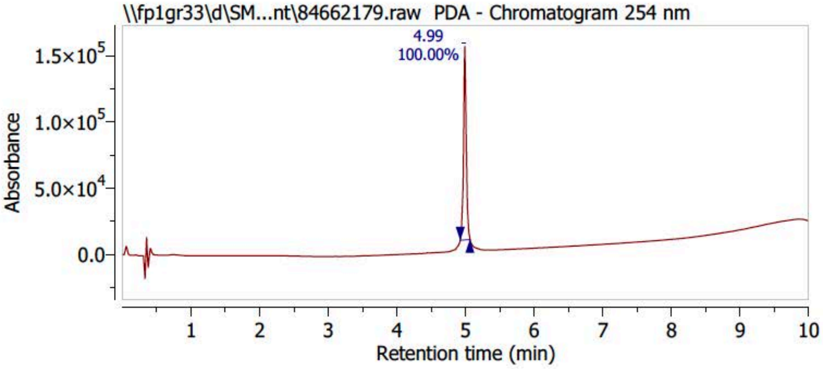

DAD, Sig=254 (>=1.5%)

RT	Area	Area %	MS+
4.99	1997127.50	100.00	956.08

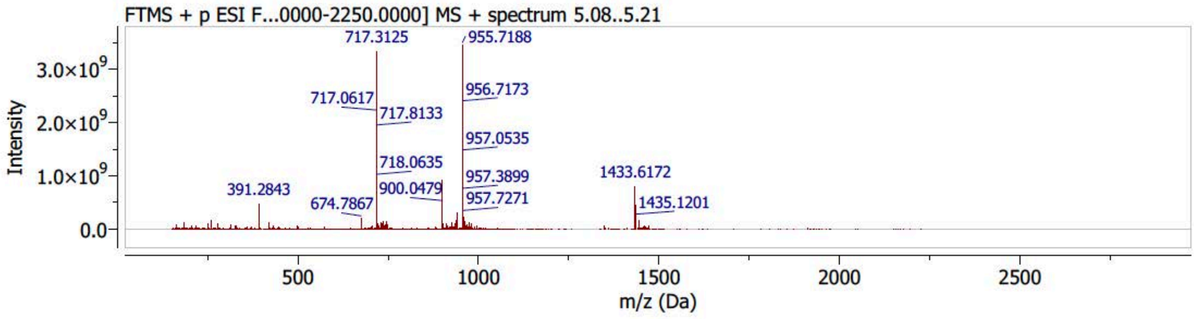

LC-MS data for compound (43) BETcide16 W10-P6 - G03409131

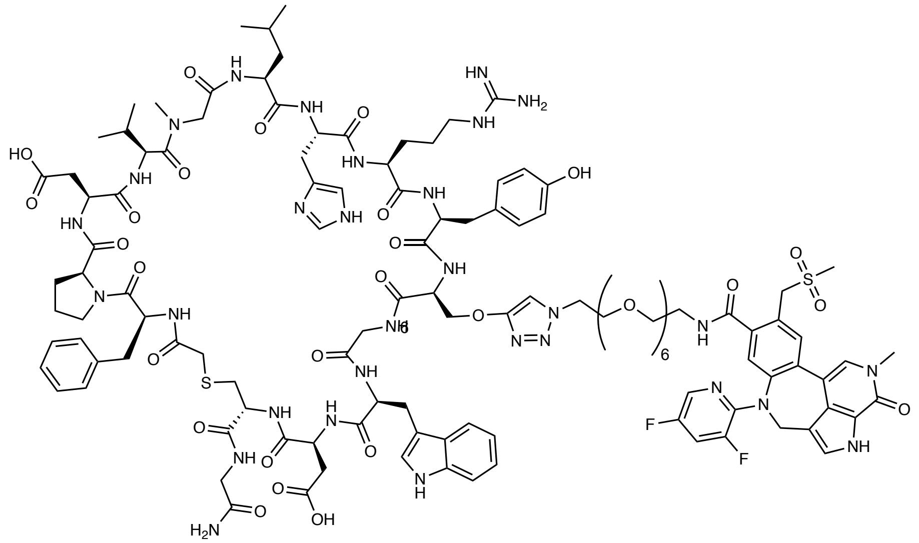
43

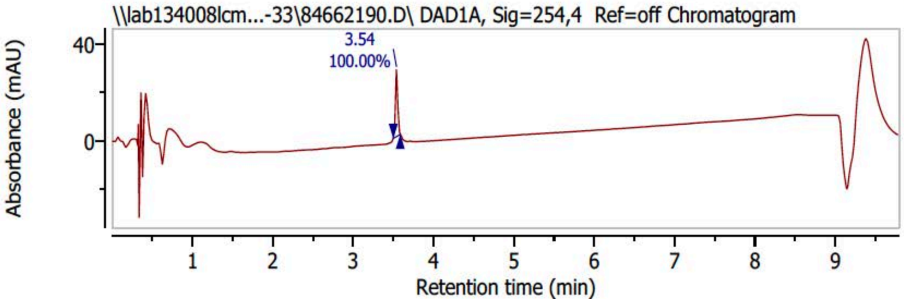

DAD1A, Sig=254,4 Ref=off (>=1.5%)

RT	Area	Area %	MS+
3.54	1096.57	100.00	878.40

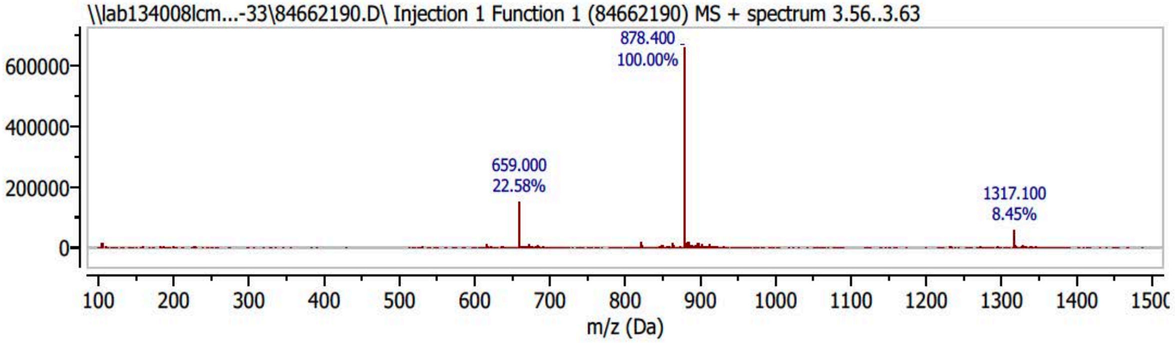

LC-MS data for compound (44) D-BETcide8 V4-P6 - G03438264

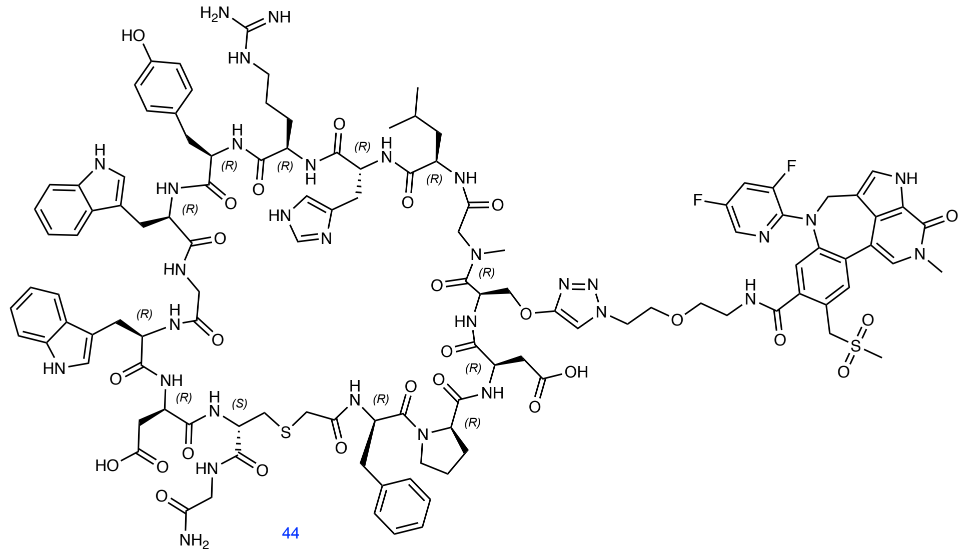

44

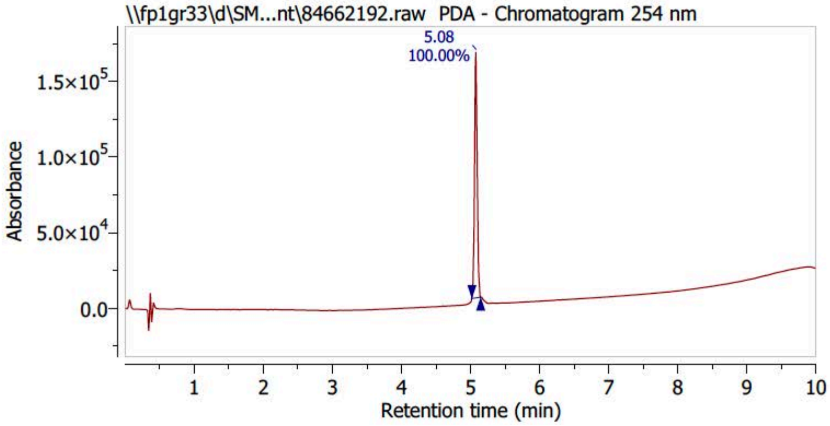

DAD, Sig=254 (>=1.5%)

RT	Area	Area %	MS+
5.08	2131509.50	100.00	907.38

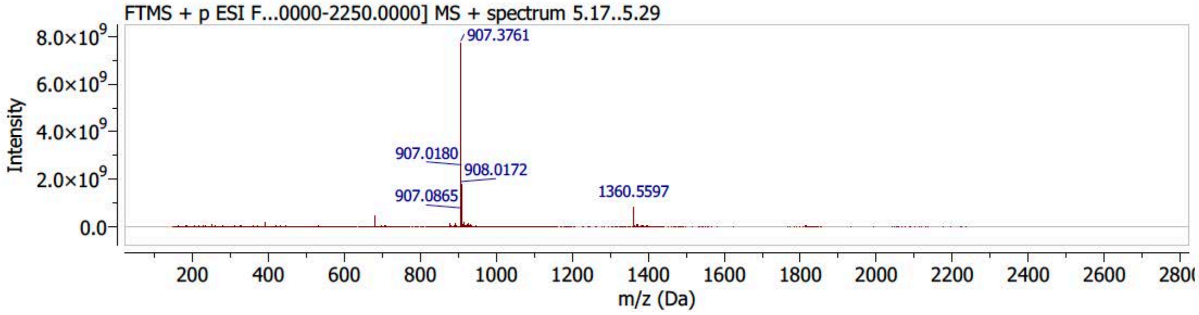

2. Chemical structures and LC-Ms Data for Biotin Conjugates

Table (3) Representation of Macrocyclic Peptides and their respective Biotin conjugates showing Abbreviations of Compound Names

Peptides used in this study					
**peptide name**	**Peptide Sequence**	**nickname**	**peptide type**	**c-term modification**	**amino acid stereochemistry**
PSMD2-MC1	cyc(Ac) FPDV(Sar)LHRYWGWDCG amide	MC1	macrocycle	-	L
PSMD2-MC2	cyc(Ac) FSDKPLHRYVGFQCG amide	MC2	macrocycle	-	L
PSMD2-MC3	cyc(Ac) FSNWPSWLHYLCG amide	MC3	macrocycle	-	L
PSMD2-pp1	Ac SHHSQVLAFAR amide	PP1	linear peptide	-	L
PSMD2-$MC1_{biotin}$	cyc(Ac) FPDV(Sar)LHRYWGWDCG(Peg3)(K(B)) amide	$MC1_{biotin}$	macrocycle	PEG3-Lys biotin	L
PSMD2-$MC2_{biotin}$	cyc(Ac) FSDKPLHRYVGFQCG(Peg3)(K(B)) amide	$MC2_{biotin}$	macrocycle	PEG3-Lys biotin	L
PSMD2-$MC3_{biotin}$	cyc(Ac) FSNWPSWLHYLCG(Peg3)(K(B)) amide	$MC3_{biotin}$	macrocycle	PEG3-Lys biotin	L
PSMD2-$PP1_{biotin}$	Ac SHHSQVLAFARG(Peg3)(K(B)) amide	$PP1_{biotin}$	macrocycle	PEG3-Lys biotin	L
PSMD2-D-MC_{biotin}	cyc(Ac) fpdv(Sar)lhrywGwdcG(Peg3)(K(B)) amide	D-$MC1_{biotin}$	macrocycle	PEG3-Lys biotin	D

LC-MS data for compound (45) PSMD2-MC1

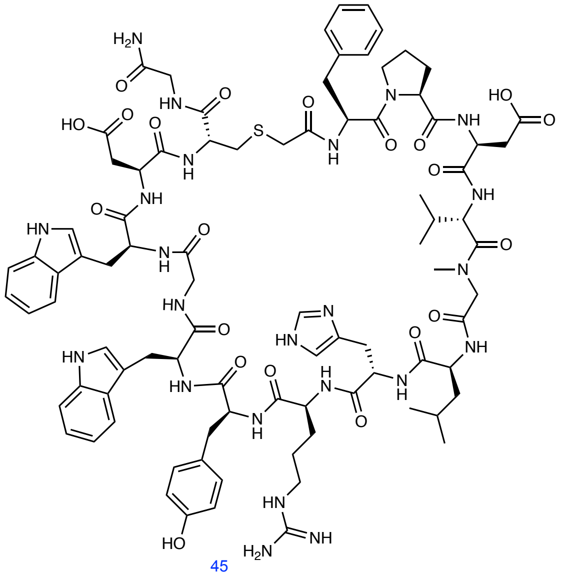

45

Chromatogram

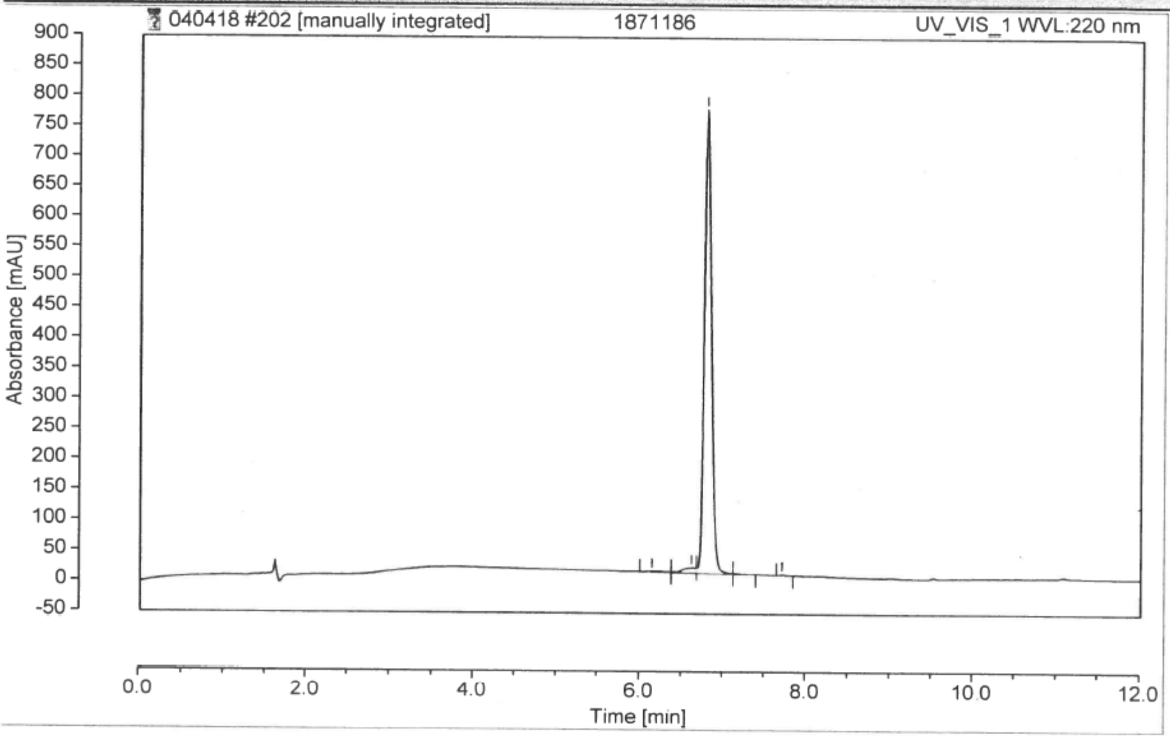

C:\Xcalibur\Data\1871186 04/13/18 09:46:12

1871186 #38 RT: 0.24 AV: 1 NL: 1.18E6
F: ITMS + c ESI E Full ms [50.00-2000.00]

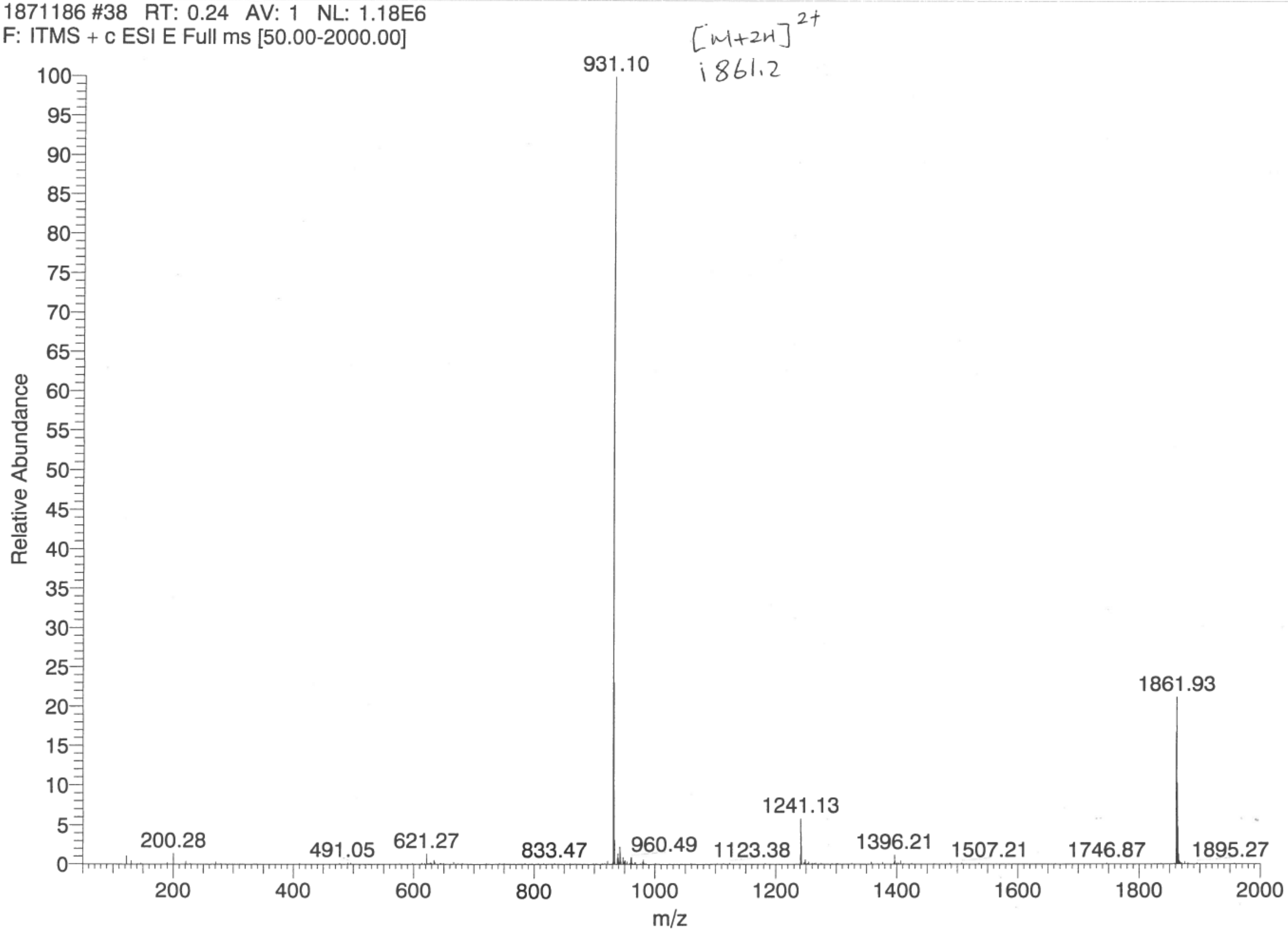

LC-MS data for compound (46) PSMD2-MC2

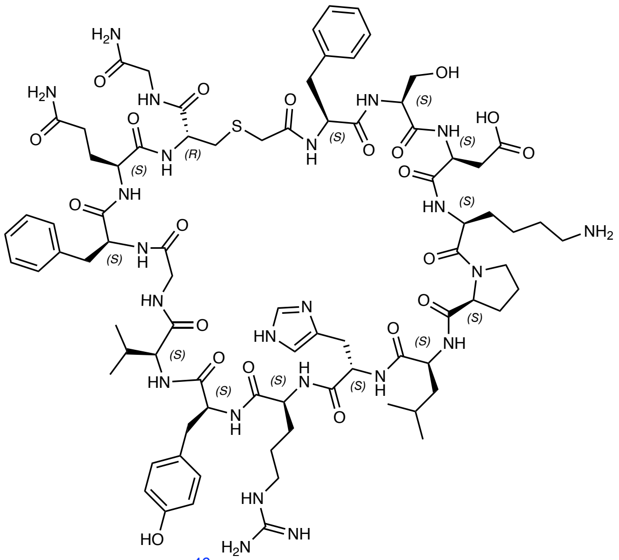

46

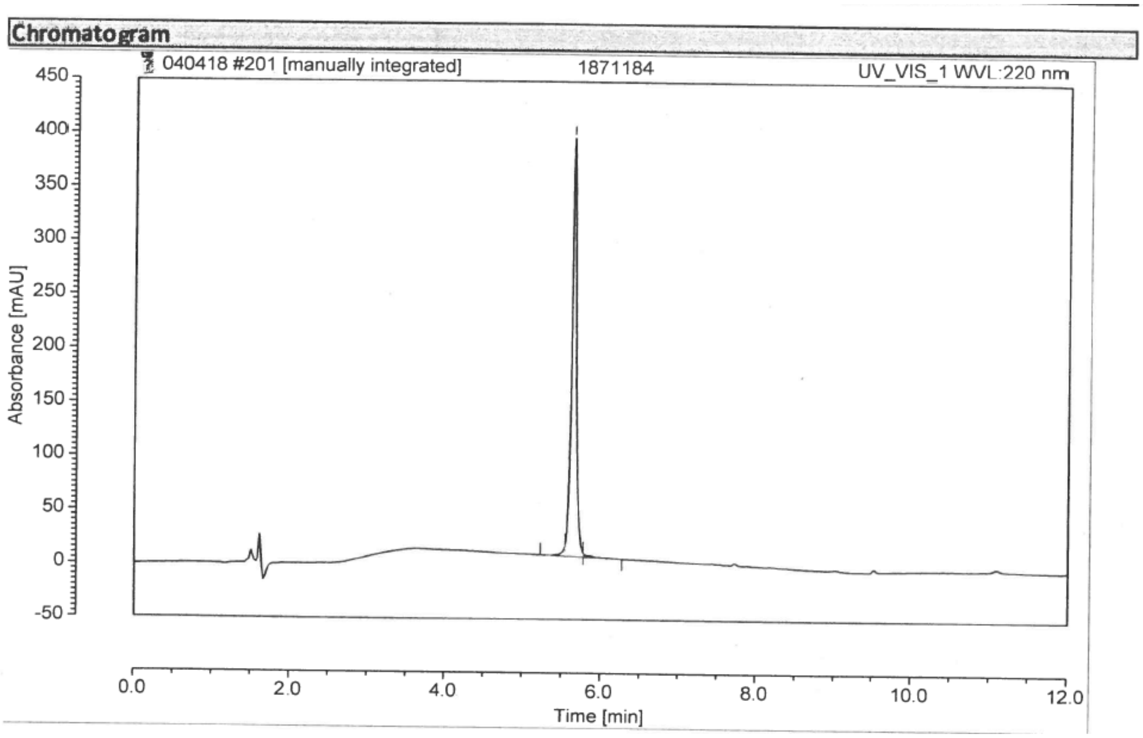

C:\Xcalibur\Data\1871184 04/13/18 09:43:17

1871184 #38 RT: 0.24 AV: 1 NL: 3.73E5
F: ITMS + c ESI E Full ms [50.00-2000.00]

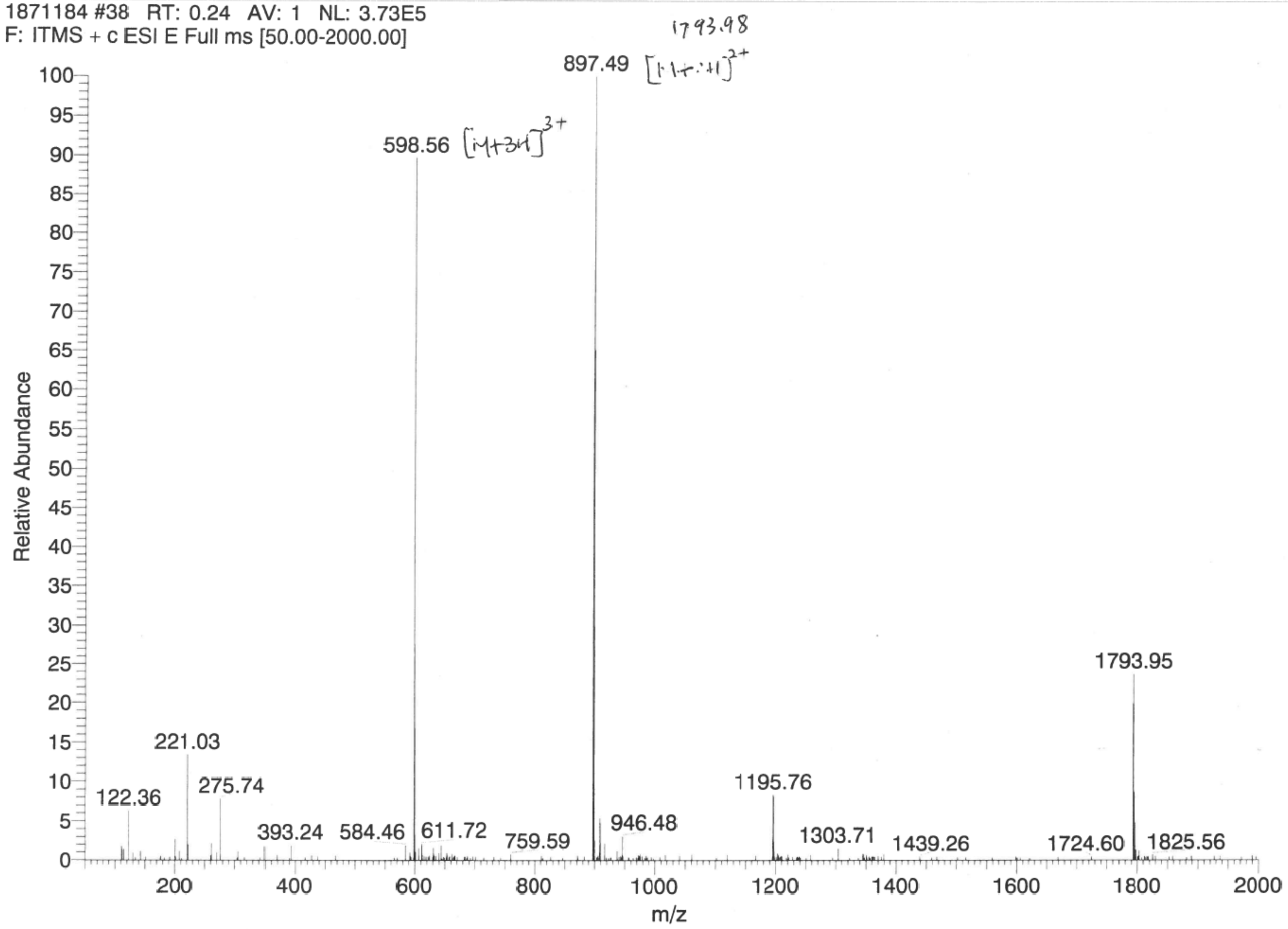

LC-MS data for compound (47) PSMD2-MC3

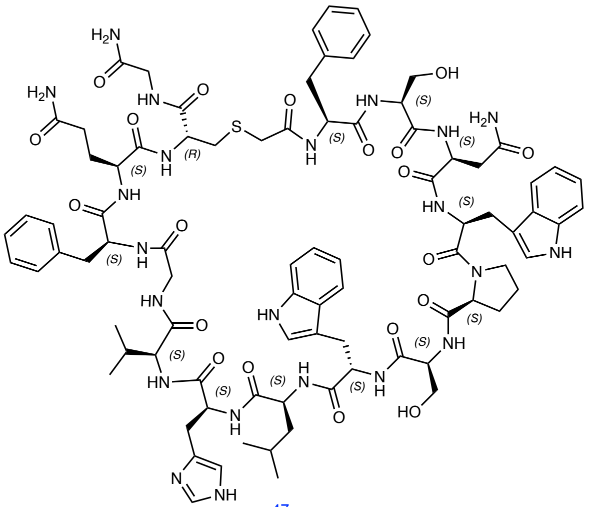

47

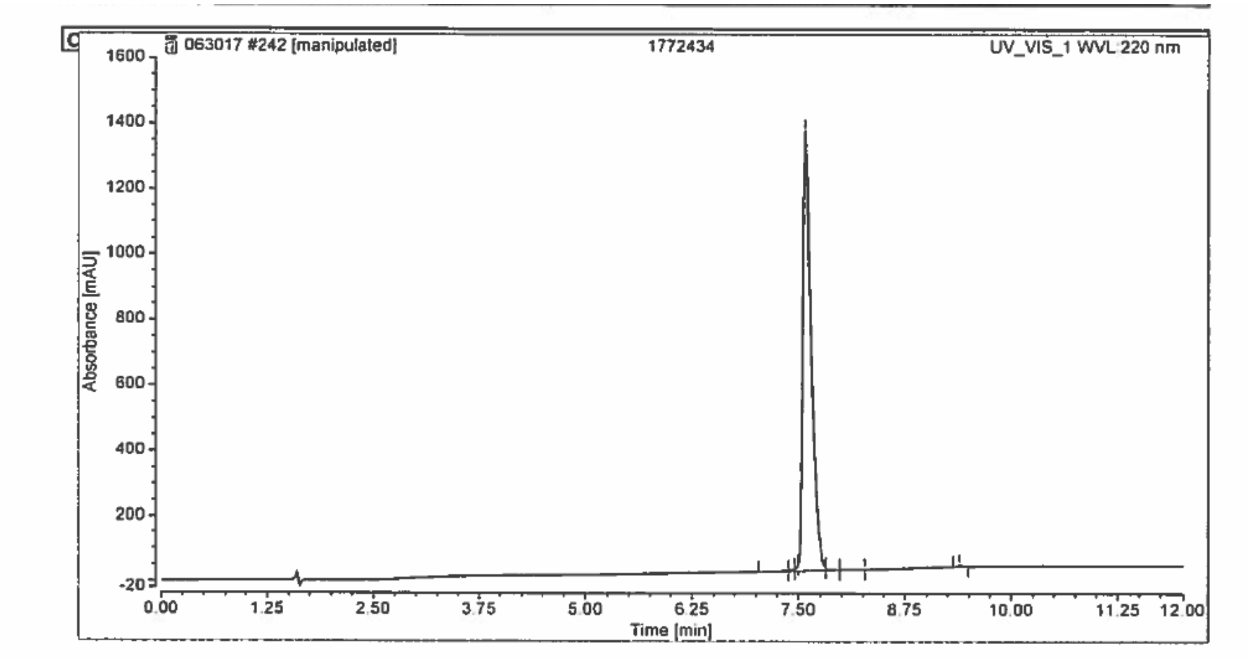

C:\Xcalibur\data\1772434_170721143208 7/21/2017 2:32:08 PM

07/21/17

1772434_170721143208 #27-55 RT: 0.18-0.33 AV: 9 NL: 3.47E6
F: ITMS + c ESI E Full ms [50.00-2000.00]

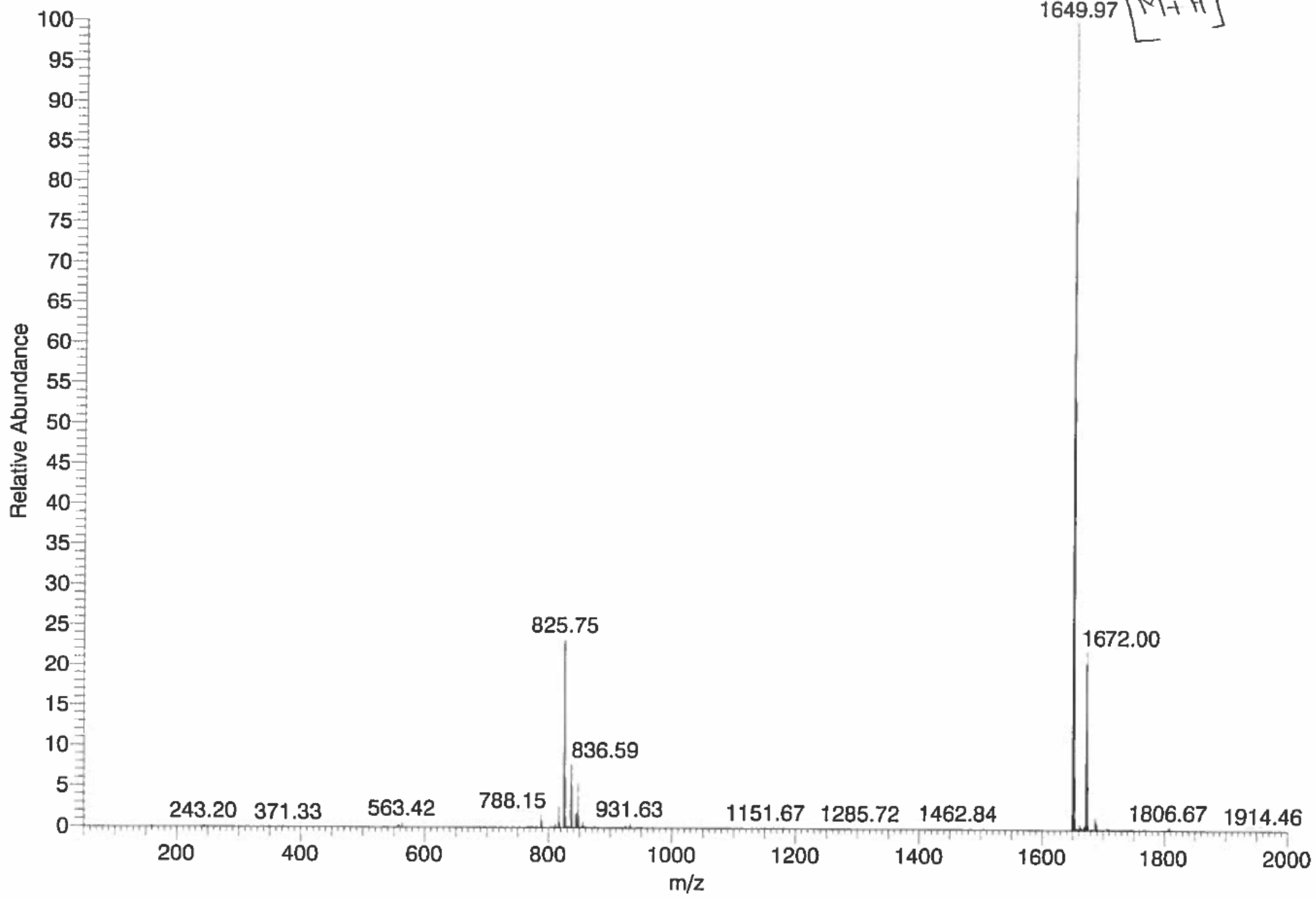

LC-MS data for compound (48) PSMD2-pp1

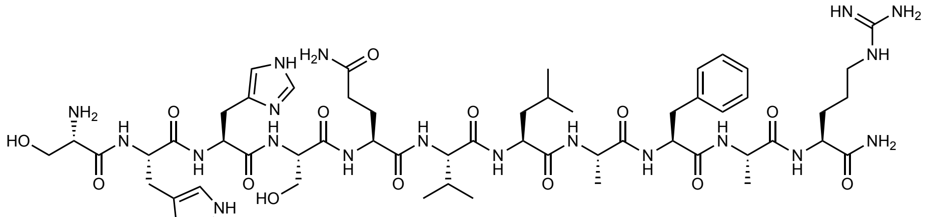

48

Chromatogram

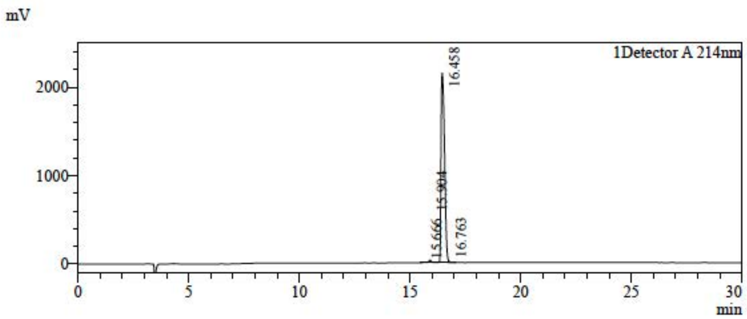

MS Spectrum

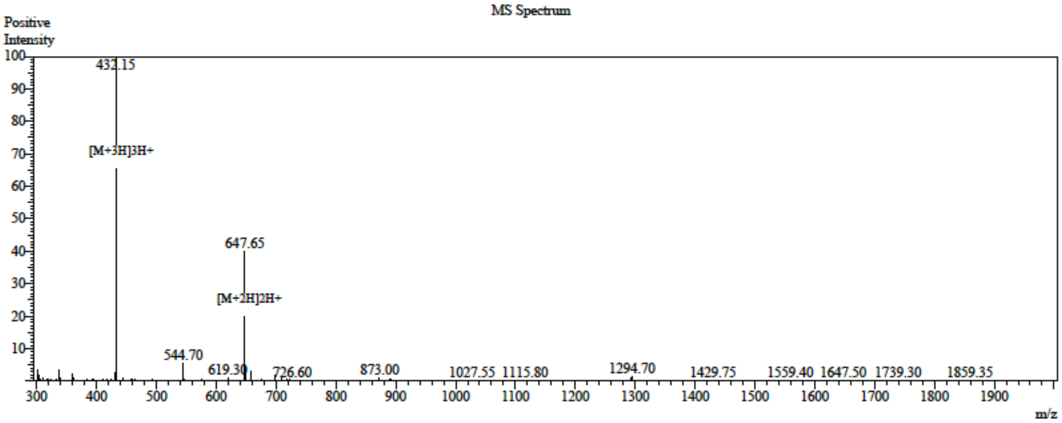

Sample Information

LC-MS data for compound (49) PSMD2-MC1biotin

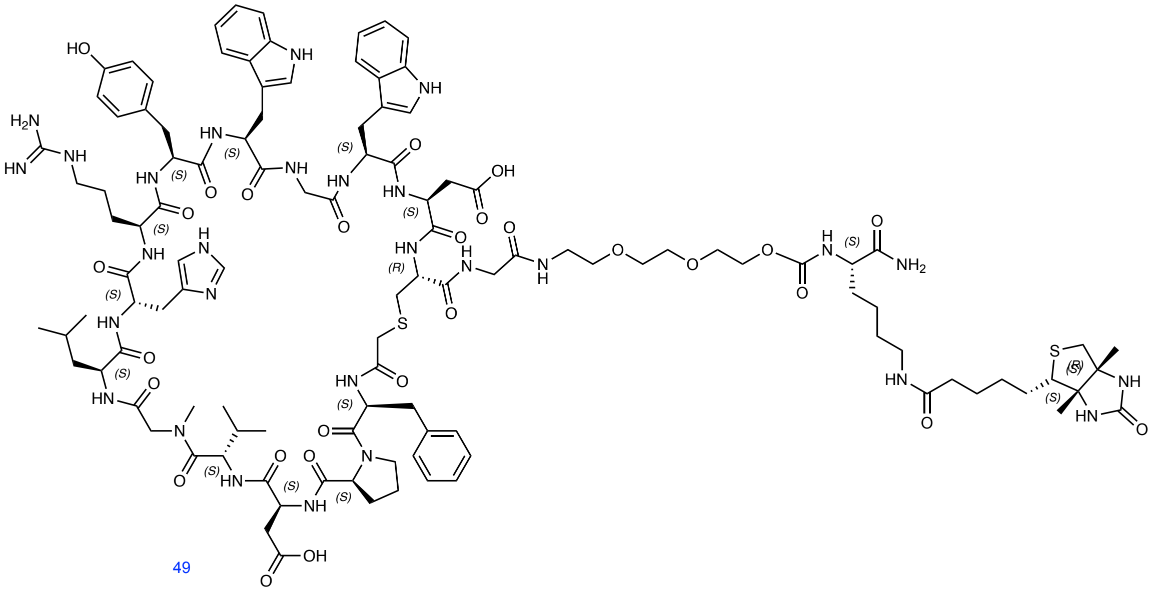

49

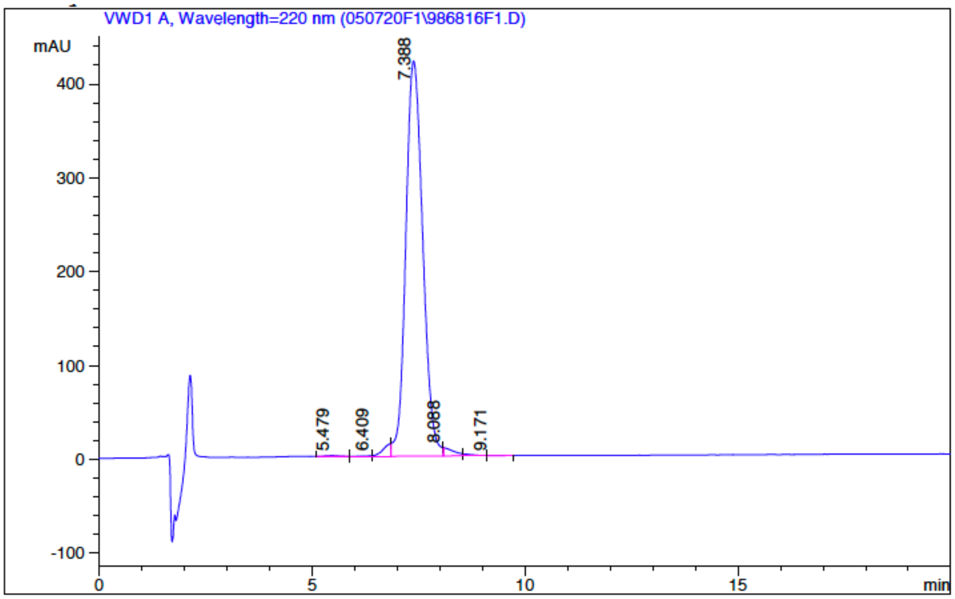

===>

Signal 1:VWD1 A, Wavelength=220 nm

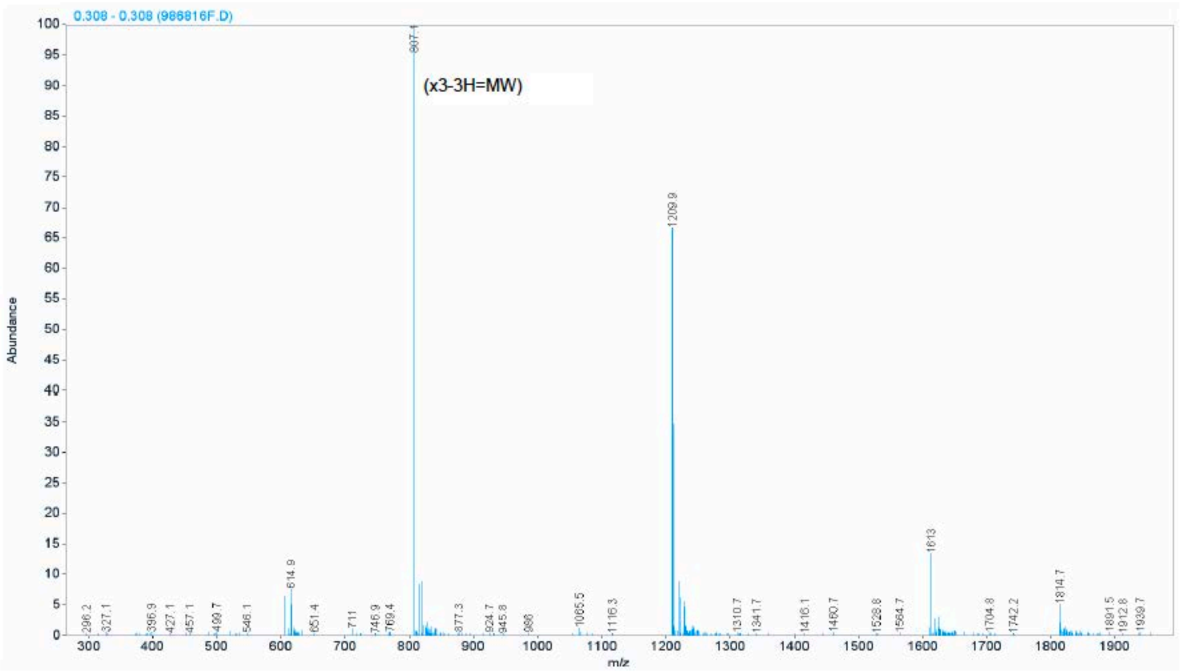

LC-MS data for compound (50) PSMD2-MC2biotin

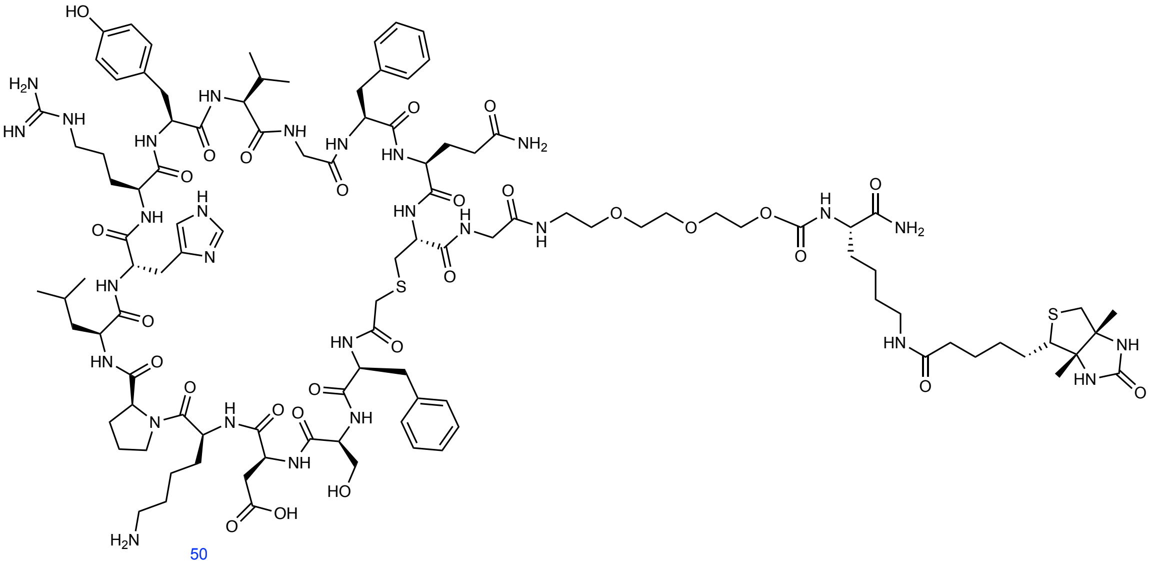

50

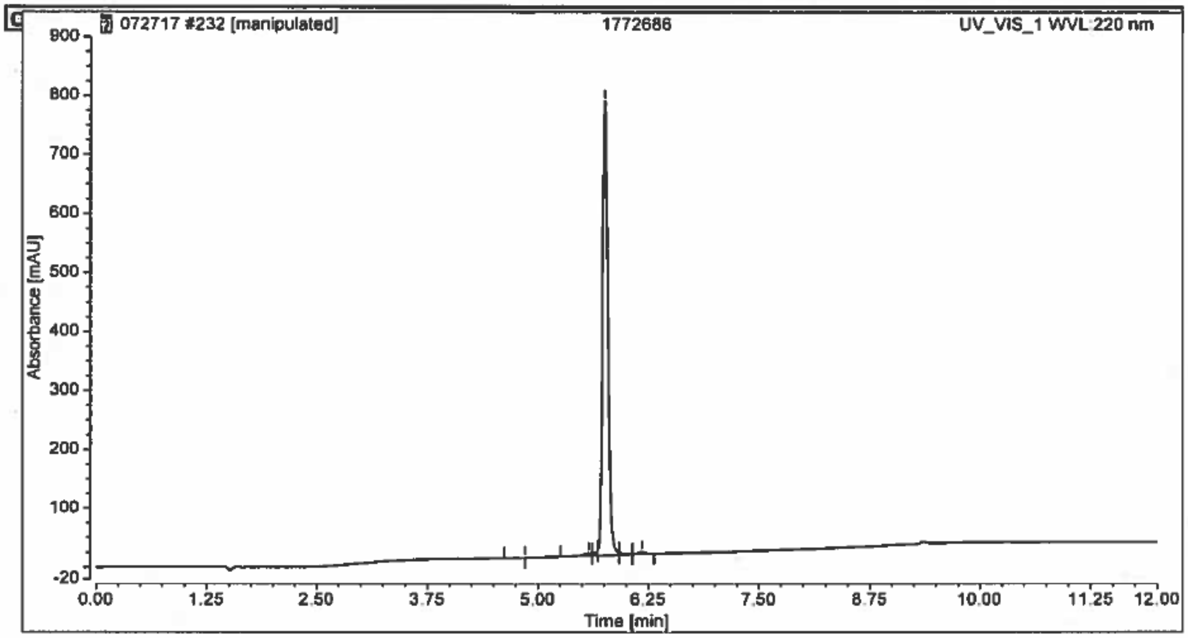
072717 #232 [manipulated]
1772686
UV_VIS_1 WVL:220 nm
900
800
700
600
500
400
300
200
100
-20
Absorbance [mAU]
0.00
1.25
2.50
3.75
5.00
6.25
7.50
8.75
10.00
11.25
12.00
Time [min]

C:\Xcalibur\data\1772686 8/11/2017 10:12:41 AM

1772686 #23-57 RT: 0.14-0.35 AV: 12 NL: 2.88E6
F: ITMS + c ESI E Full ms [50.00-2000.00]

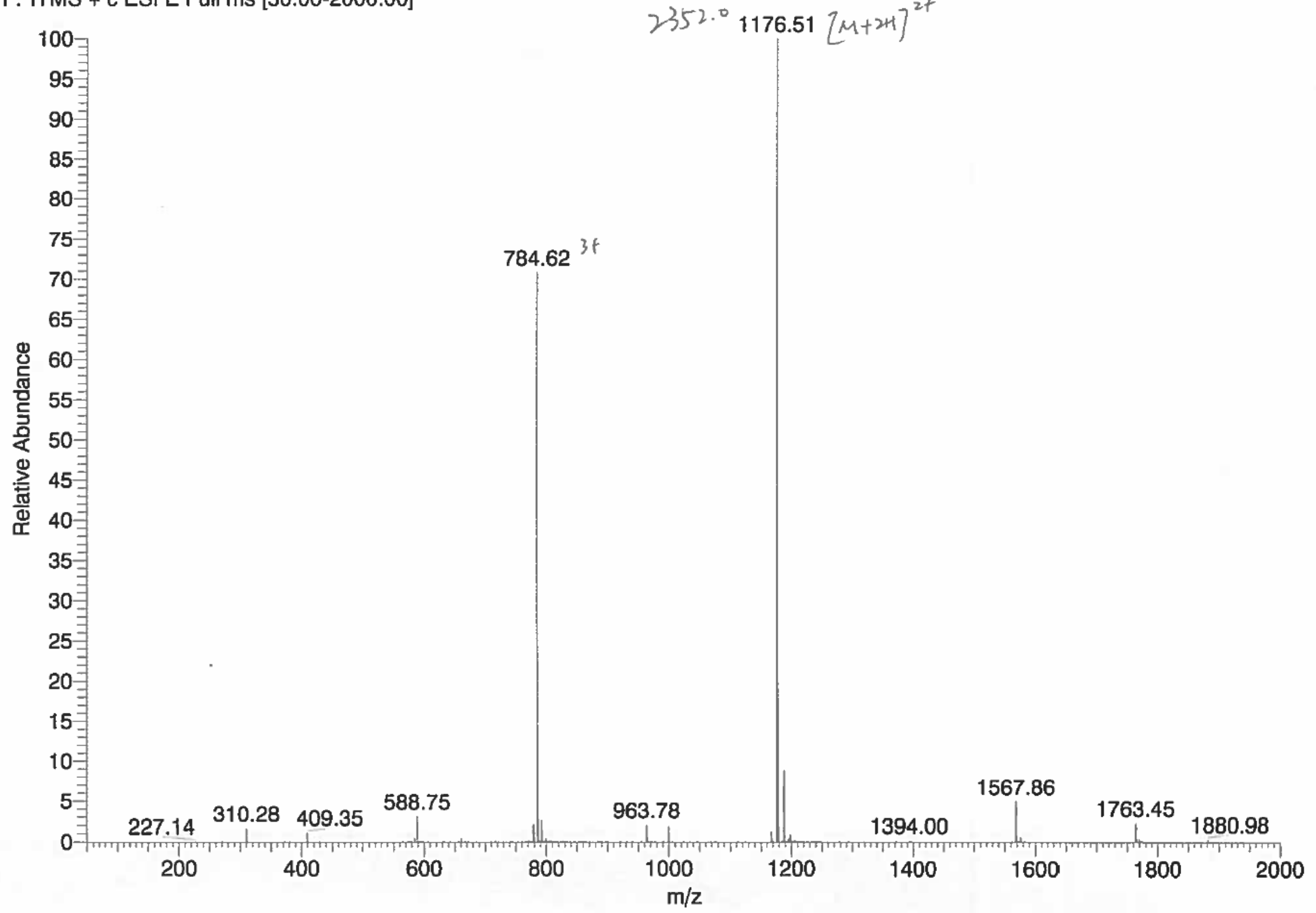

LC-MS data for compound (51) PSMD2-MC3biotin

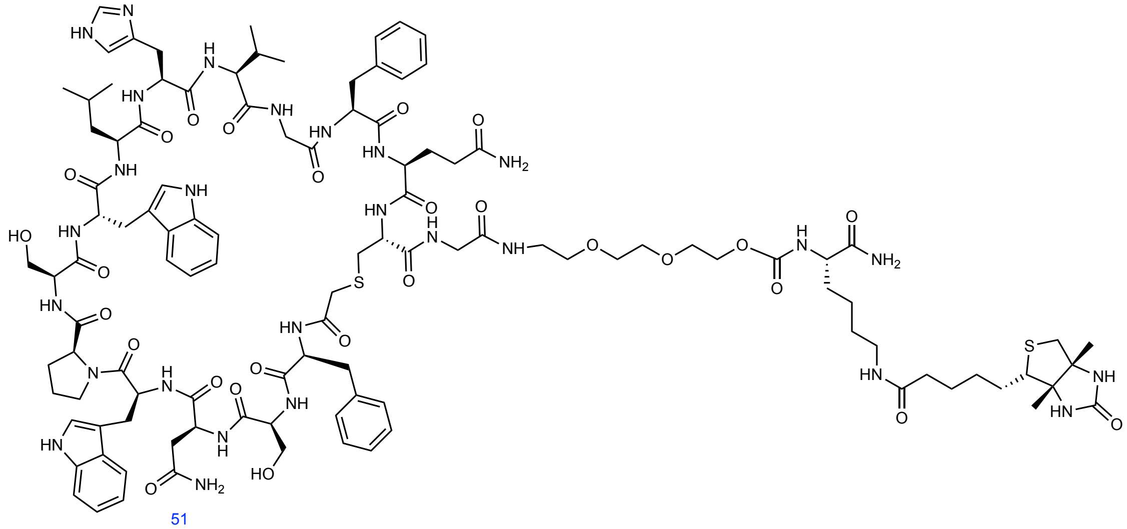

51

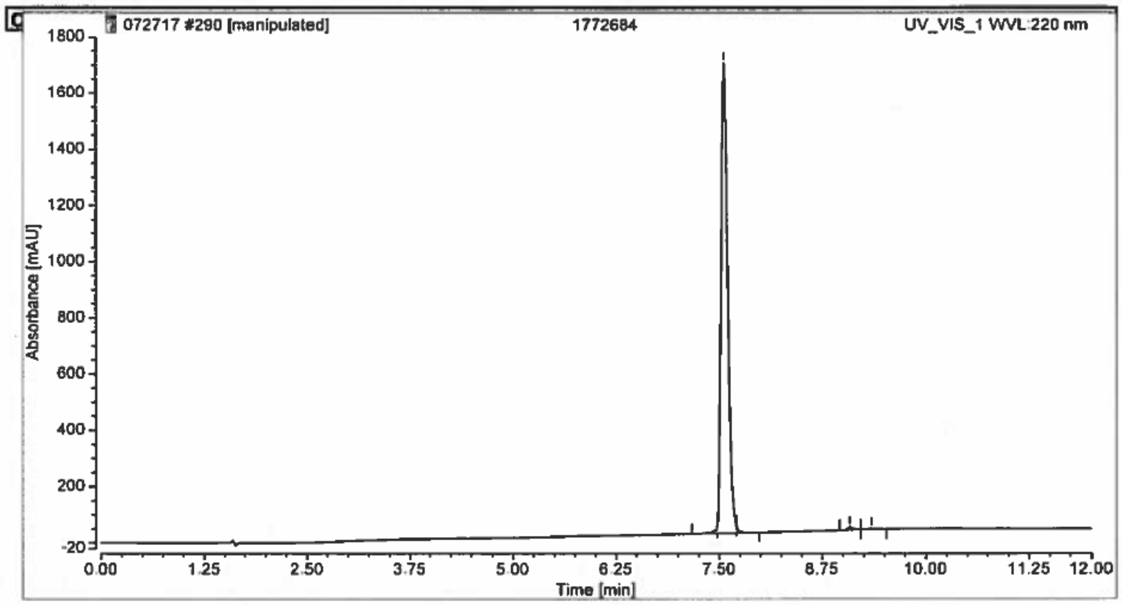

C:\Xcalibur\Data\1772684 8/16/2017 1:45:00 PM

08/16/17

1772684 #19-54 RT: 0.17-0.46 AV: 12 NL: 6.41E5
F: ITMS - c ESI Full ms [50.00-2000.00]

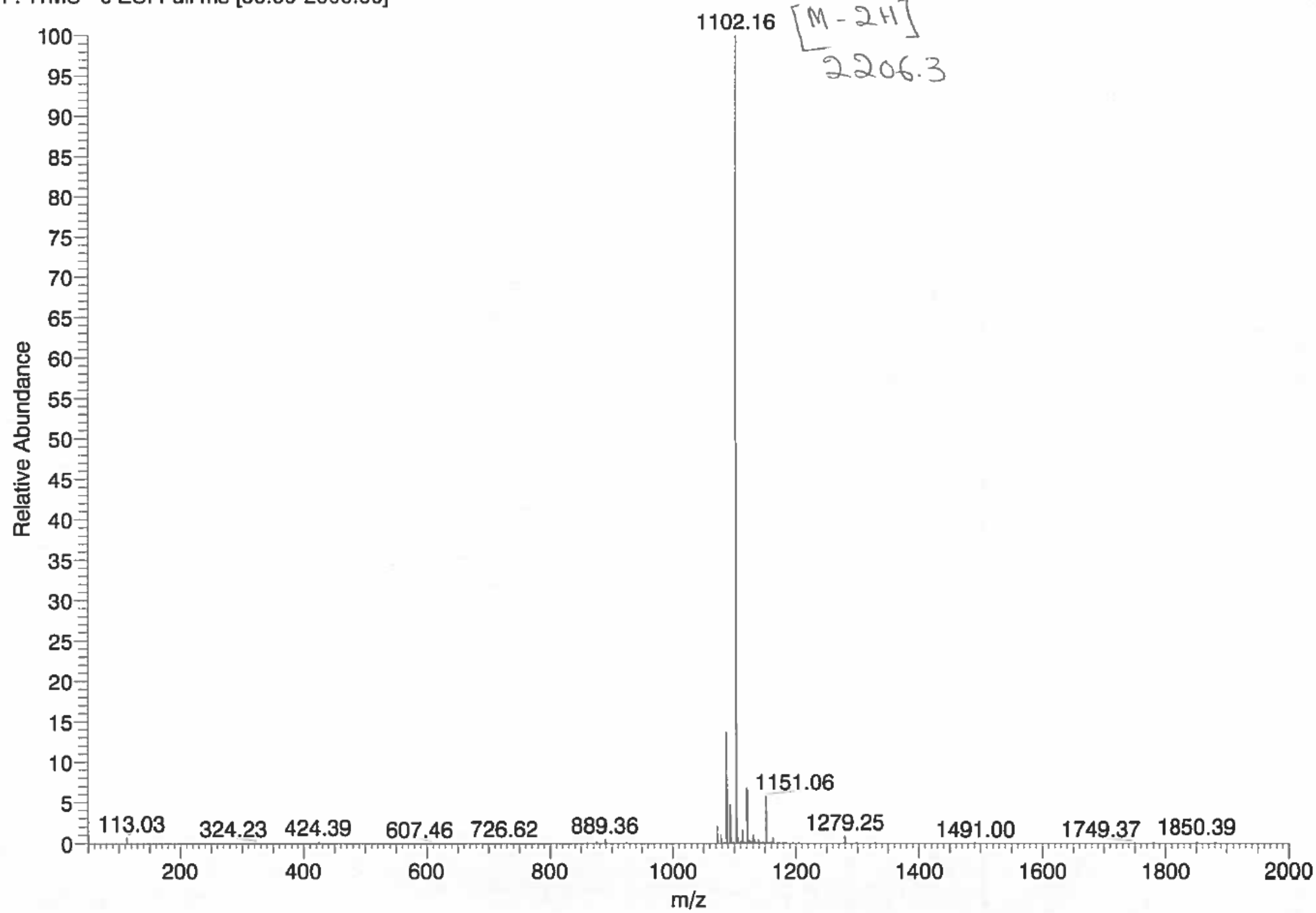

LC-MS data for compound (52) PSMD2-PP1biotin

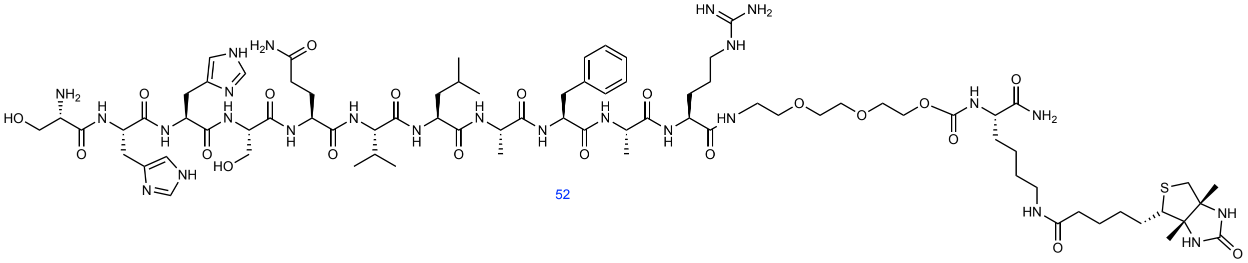

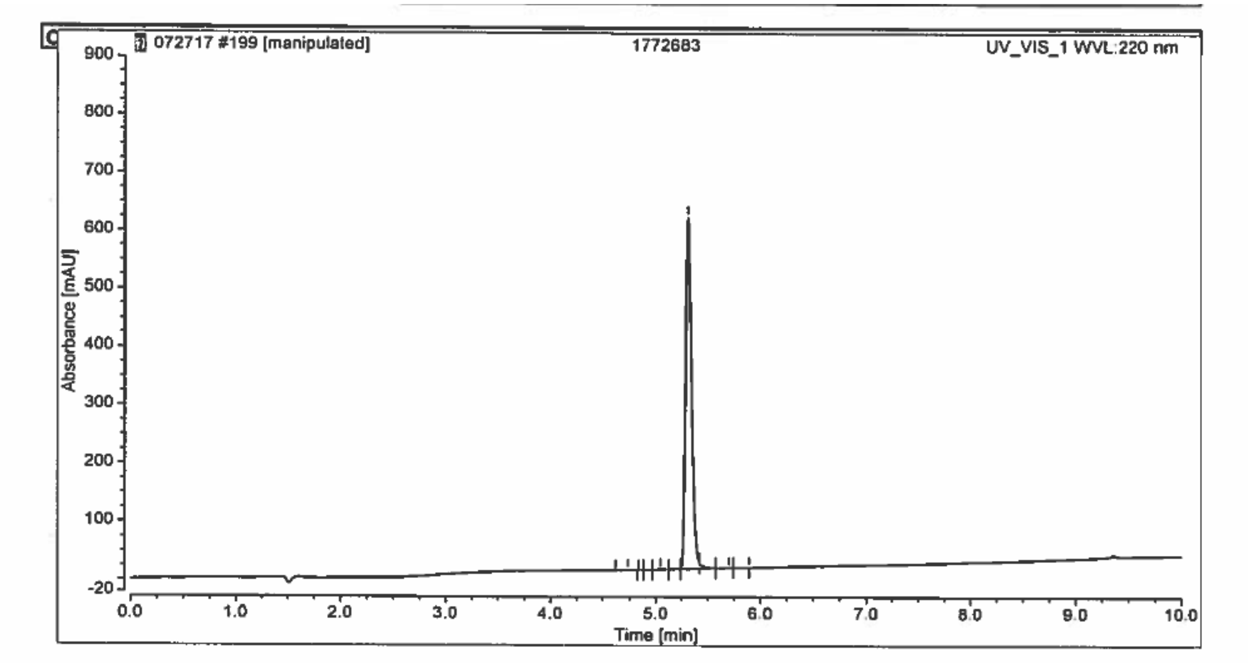

C:\Xcalibur\data\1772683_170809151405 8/9/2017 3:14:05 PM

08/09/17

1772683_170809151405 #19-55 RT: 0.17-0.48 AV: 13 NL: 3.21E5
F: ITMS - c ESI Full ms [50.00-2000.00]

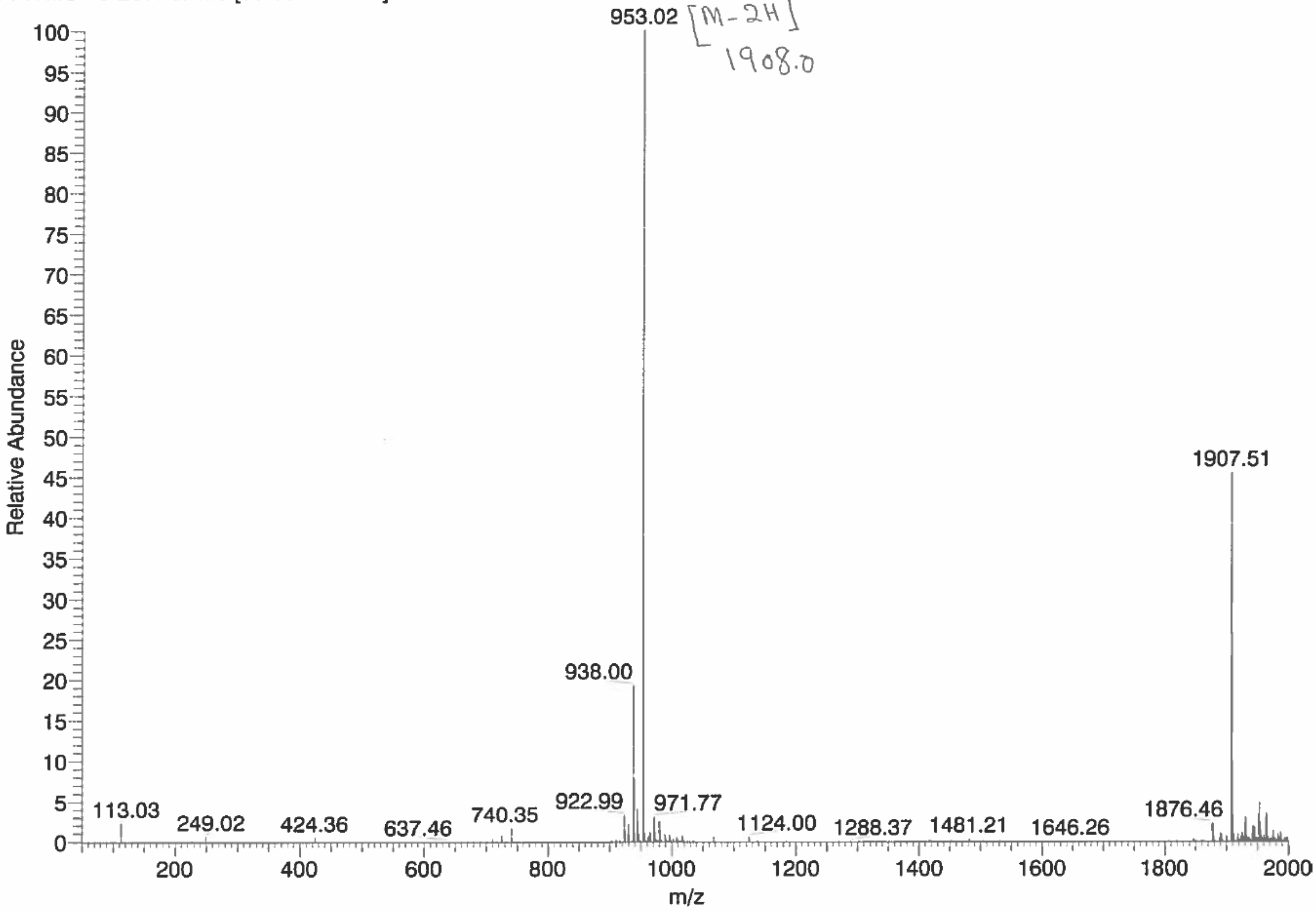

LC-MS data for compound (53) PSMD2-D-MCbiotin

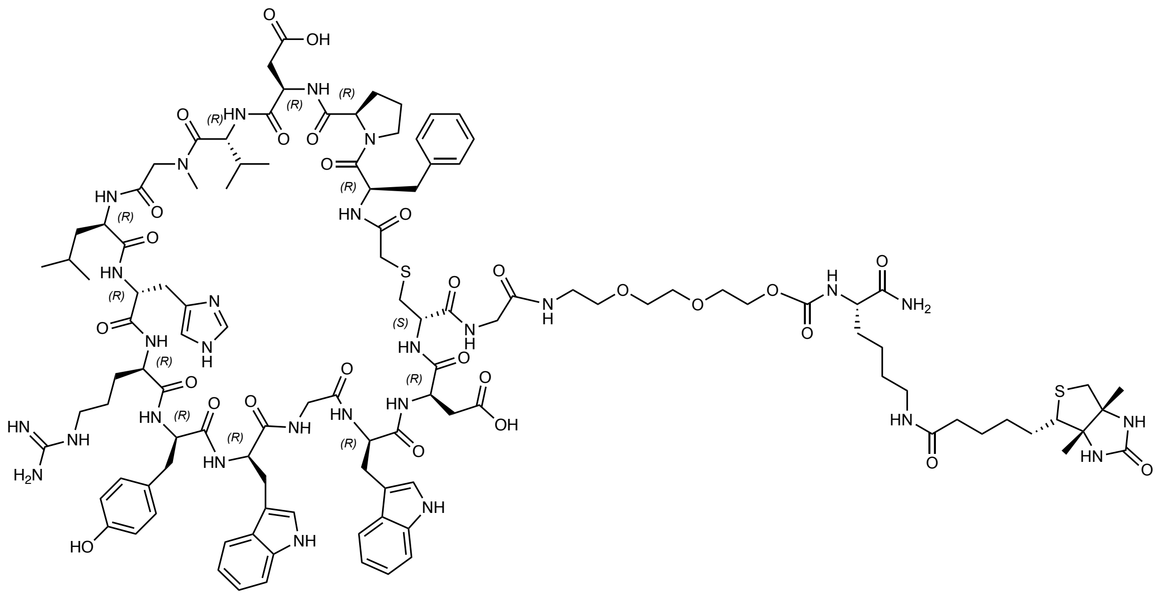

53

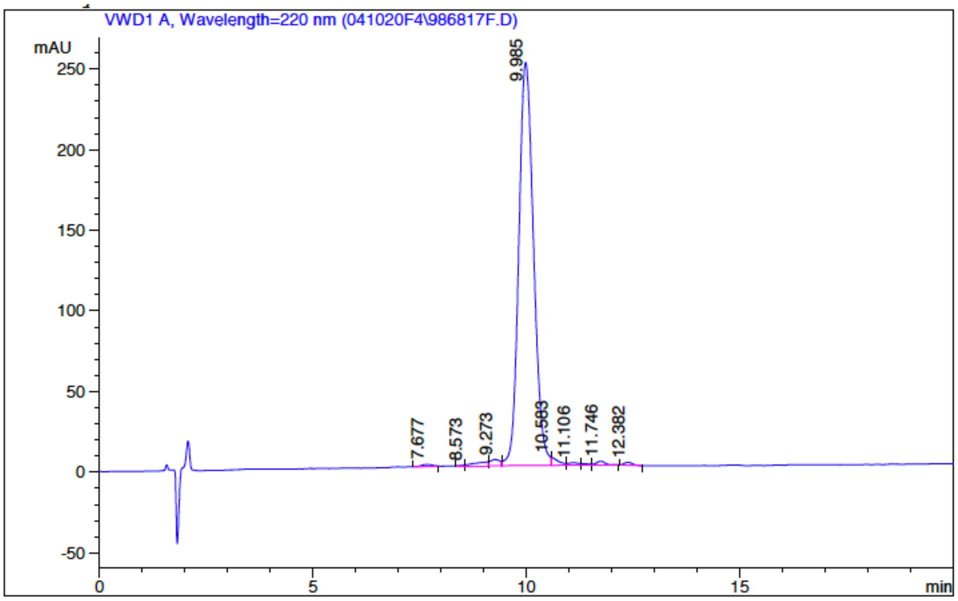

===>

Signal 1:VWD1 A, Wavelength=220 nm

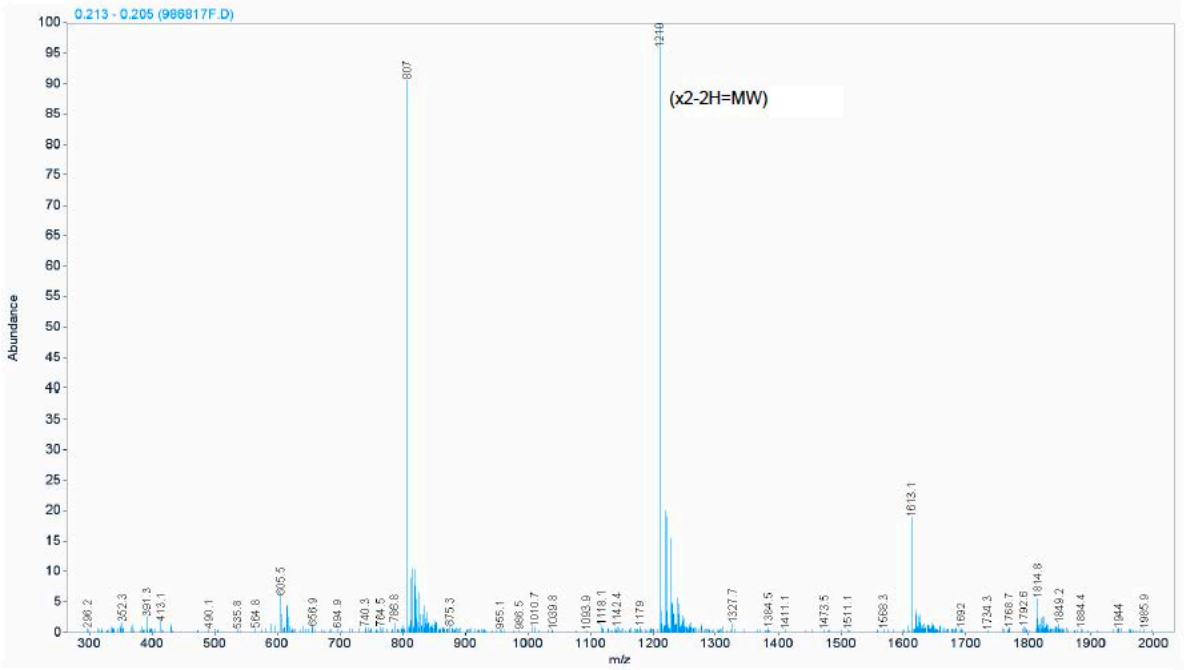

LC-MS data for compound (54) fluorescently labeled peptide $MC1_{Cy5.5}$

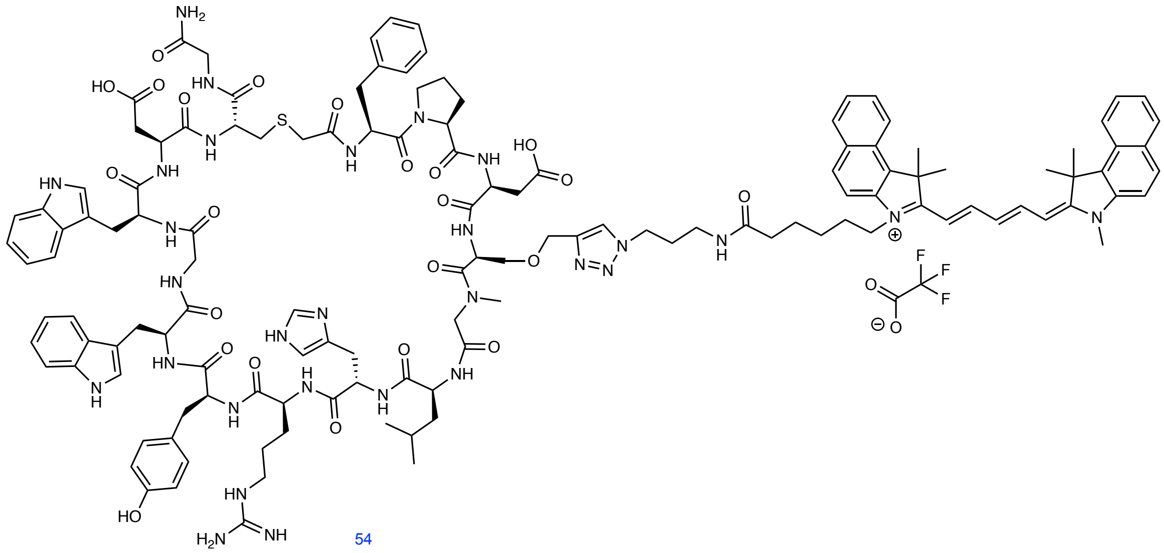

54

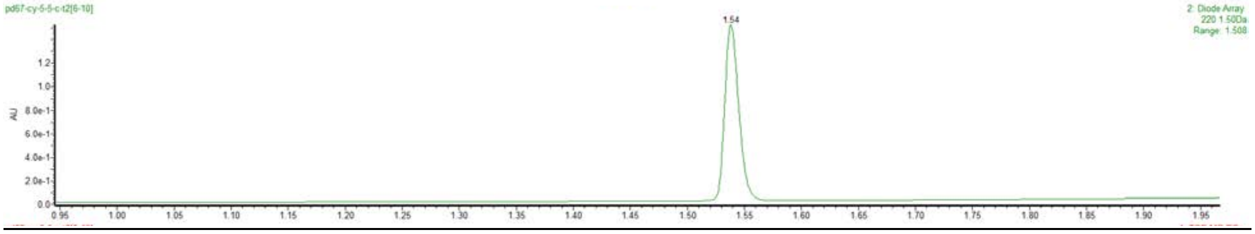

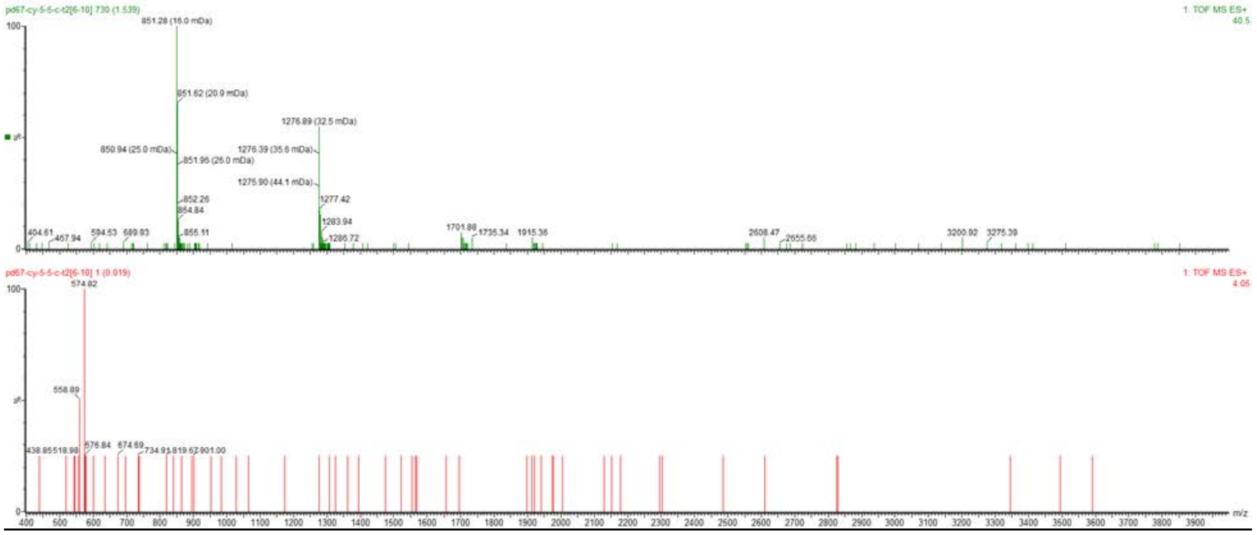

pd67-cy-5-5-c-t2[6-10] 730 (1.539)
1: TOF MS ES+
40.5
851.28 (16.0 mDa)
851.62 (20.9 mDa)
1276.89 (32.5 mDa)
850.94 (25.0 mDa)
851.95 (26.0 mDa)
1276.39 (35.6 mDa)
1275.90 (44.1 mDa)
852.26
854.84
1277.42
1283.94
404.61
467.94
594.53
689.93
855.11
1286.72
1701.88
1735.34
1915.36
2608.47
2655.66
3200.92
3275.39
pd67-cy-5-5-c-t2[6-10] 1 (0.019)
1: TOF MS ES+
4.05
574.82
558.89
438.85
518.98
576.84
674.69
734.91
819.67
901.00
m/z
