# Supplementary material for: Targeted degradation via direct 26S proteasome recruitment
Source: Nat Chem Biol. 2022 Dec 28;19(1):55–63. doi: 10.1038/s41589-022-01218-w (PMC9797404; doi:10.1038/s41589-022-01218-w)
Supplement: Supplementary file 2 — Reporting Summary [file 41589_2022_1218_MOESM2_ESM.pdf]

nature research

Corresponding author(s): Erin C. Dueber, Claudio Ciferri, Ingrid E. Wertz

Last updated by author(s): Aug 26, 2021

Reporting Summary

Nature Research wishes to improve the reproducibility of the work that we publish. This form provides structure for consistency and transparency in reporting. For further information on Nature Research policies, see our Editorial Policies and the Editorial Policy Checklist.

Statistics

For all statistical analyses, confirm that the following items are present in the figure legend, table legend, main text, or Methods section.

n/a	Confirmed	
☐	☒	The exact sample size (*n*) for each experimental group/condition, given as a discrete number and unit of measurement
☐	☒	A statement on whether measurements were taken from distinct samples or whether the same sample was measured repeatedly
☐	☒	The statistical test(s) used AND whether they are one- or two-sided<br>*Only common tests should be described solely by name; describe more complex techniques in the Methods section.*
☒	☐	A description of all covariates tested
☐	☒	A description of any assumptions or corrections, such as tests of normality and adjustment for multiple comparisons
☐	☒	A full description of the statistical parameters including central tendency (e.g. means) or other basic estimates (e.g. regression coefficient) AND variation (e.g. standard deviation) or associated estimates of uncertainty (e.g. confidence intervals)
☒	☐	For null hypothesis testing, the test statistic (e.g. *F*, *t*, *r*) with confidence intervals, effect sizes, degrees of freedom and *P* value noted<br>*Give P values as exact values whenever suitable.*
☒	☐	For Bayesian analysis, information on the choice of priors and Markov chain Monte Carlo settings
☒	☐	For hierarchical and complex designs, identification of the appropriate level for tests and full reporting of outcomes
☐	☒	Estimates of effect sizes (e.g. Cohen's *d*, Pearson's *r*), indicating how they were calculated

Our web collection on statistics for biologists contains articles on many of the points above.

Software and code

Policy information about availability of computer code

Data collection

All software information is detailed, with relevant citations, in the Materials and Methods section.

For mRNA display selections, qPCR data were collected on a QuantStudio 5 thermal cycler (Thermo) with corresponding software. Next-generation sequencing was carried out using a MiSeq next generation sequencer (Illumina). SPR data was collected using BiaControl (Biacore, Cytiva). ELISA data were collected on a Multiskan Ascent V1.24 and using Ascent Software V 2.6, with data exported to Excel. AlphaScreen data were collected on an Envision Plate Reader using Envision Manager software (Perkin Elmer) with data exported to Excel. TMT data was collected by nano LC/MS/MS with a Waters NanoAcquity HPLC system interfaced to a ThermoFisher Fusion Lumos mass spectrometer. The mass spectrometer was operated using a custom MS3 method. In-gel digestion and LC/MS/MS utilized a hybrid ThermoFisher Orbitrap Fusion mass spectrometer. NMR spectra were acquired at 298 K on a Bruker Advance 600 MHz spectrometer equipped with a 5-mm triple-resonance cryogenic probe. EM data collection, microscope and camera automation was acomplished by SerialEM version 3.7.14. Western blots were imaged using Odyssey imager (Licor) for fluorescence and a PXi imager (Syngene) for chemiluminescence. Sypro Ruby stained gels were imaged on a Typhoon Trio (Cytiva) using ImageQuant software. Pulldowns and mRNA Display selections were done using BindIt software on a KingFisher Duo Prime (Thermo). HRF experiments were done on a Thermo Fisher Q Exactive. Fluorescence microscopy and internalization experiments were done using a spinning disk confocal microscopy was performed using a CSU-W1 (Yokogawa) spinning disk on a Zeiss AxioObserver M1 microscope. Images were acquired using SlideBook v6 (Intelligent Imaging Innovations, Denver CO) and a Photometrics Prime BSI (Teledyne Photometrics, Tucson AZ). Figures were assembled in Adobe Photoshop 2021 and any gamma adjustments to the contrast were applied across the whole image. Quantitative fluorescence imaging of BRD4 was carried out using an Opera Phenix High-Content Screening System. qPCR was measured on QuantStudio 7 Flex RT-PCR system.

Data analysis

All software information is detailed, with relevant citations, in the Materials and Methods section.

Microsoft Excel and GraphPad Prism were used for data analysis across experiments. TMT-MS and in-gel digestion LC/MS/MS data were analyzed via in-house software and searched MASCOT algorithm (Matrix Science Inc), against UniProt.org Human taxonomy database with appropriate modification parameters. TMT quantitative results were processed with in-house software Mojave then results were compiled

and visualized with custom Spotfire® Dashboard (TIBCO). TMT analysis and statistics were done using the MSStats package for R, and final volcano plots and the waterfall plot were made using R/ggplot2. NMR spectra were processed using TOPSPIN (Bruker). NMR spectral analyses and assignments were performed using Mnova (Mestrelab Research) and NMRFAM-SPARKY software. EM data were processed using RELION version 3.1.1 and cisTEM version 2.0.0-alpha. Atomic models were built using Coot and refined with Phenix. Buried surface area was calculated using PISA. HRF data analysis was performed using Biologic Softawre Suite (Protein Metric Inc). Degradation of BRD4 measured by immunofluorescence was carried out using Genedata Screener.

For manuscripts utilizing custom algorithms or software that are central to the research but not yet described in published literature, software must be made available to editors and reviewers. We strongly encourage code deposition in a community repository (e.g. GitHub). See the Nature Research guidelines for submitting code & software for further information.

Data

Policy information about availability of data

All manuscripts must include a data availability statement. This statement should provide the following information, where applicable:

- Accession codes, unique identifiers, or web links for publicly available datasets
- A list of figures that have associated raw data
- A description of any restrictions on data availability

The PSMD2/MC1/fab and PSMD2/fab maps have been submitted to the Electron Microscopy Databank (https://www.ebi.ac.uk/pdbe/emdb/) under accession #s 24742 and 24743, respectively. The PSMD2/MC1 model has been deposited to the Protein Databank (rcsb.org) under accession # ZZZZ. All other data generated or analyzed during this study are included in this published article and its supplementary information files.

Field-specific reporting

Please select the one below that is the best fit for your research. If you are not sure, read the appropriate sections before making your selection.

☒ Life sciences ☐ Behavioural & social sciences ☐ Ecological, evolutionary & environmental sciences

For a reference copy of the document with all sections, see nature.com/documents/nr-reporting-summary-flat.pdf

Life sciences study design

All studies must disclose on these points even when the disclosure is negative.

Sample size	No sample size calculation was performed. Experiments were generally performed in triplicate to show standard deviation of the mean, with sample size depending on variability in experiments and availability of compound lots, when relevant.
Data exclusions	No data were excluded from the analyses
Replication	All biological experiments were done with replicates as detailed in the methods and figure legends.
Randomization	No sample randomization was done.
Blinding	No blinding was done

Reporting for specific materials, systems and methods

We require information from authors about some types of materials, experimental systems and methods used in many studies. Here, indicate whether each material, system or method listed is relevant to your study. If you are not sure if a list item applies to your research, read the appropriate section before selecting a response.

Materials & experimental systems

n/a	Involved in the study	
☐	☒	Antibodies
☐	☒	Eukaryotic cell lines
☒	☐	Palaeontology and archaeology
☒	☐	Animals and other organisms
☒	☐	Human research participants
☒	☐	Clinical data
☒	☐	Dual use research of concern

Methods

n/a	Involved in the study	
☒	☐	ChIP-seq
☒	☐	Flow cytometry
☒	☐	MRI-based neuroimaging

Antibodies

Antibodies used	BET degradation was assayed using antibodies to the indicated proteins were purchased by the following vendors: BRD4 (ab128874), BRD2 (ab139690), PSMD14 (ab109123), PSMD1 (ab140682) [AbCam]; BRD3 (A302-368A) [Bethyl]; Tubulin (926-42211) [LICOR

	Biosciences]; PSMD4 (3846) [Cell Signaling Technology]; PSMA1 (BML-PW8100-0100) [ENZO Life Sciences]. IRDye-800 anti-rabbit (926-32211) and IRDye-680RD anti-mouse (926-68072) secondary antibodies were purchased from LICOR Biosciences. ELISAs with in vitro translated macrocycles was probed using Monoclonal ANTI-FLAG® M2-Peroxidase (HRP) antibody produced in mouse, (A8592) Sigma. AlphaScreen assay uses anti PSMD1 antibody (ab140682; Abcam). Western Blotting of recombinant proteasome pulldowns used antibodies for PSMD4 (CST D17E4, #3336 Cell Signalling) and PSMA1 (BML-PW8100-0100, Enzo). Blots were imaged using Licor secondary anti-rabbit 680 and anti-mouse 800 antibodies (see previous).Anti-Streptavidin HRP was used to detect loading amounts in the blot and was developed using West Femto ECL reagent (Thermo). Immunofluorescence quantitation of BRD4 degradation used mAB Anti-BRD4 [EPR5150] antibody (Abcam 128874) and Goat Anti-Rabbit IgG, DyLight 488 Conjugated Highly Cross-adsorbed Thermo Fisher #35553.
Validation	Antibodies were used for applications validated by antibody suppliers per quality assurance by each supplier<br><br>BRD4 (ab128874), BRD2 (ab139690), antibodies are validated from the manufacturers' websites and citations therein and are also additionally KO validated; PSMD14 (ab109123), PSMD1 (ab140682), BRD3 (A302-368A), PSMD4 (3846), PSMA1 (BML-PW8100-0100) PSMD4 (D17E4 #3336), Goat Anti-Rabbit IgG, DyLight 488 Conjugated Highly Cross-adsorbed Thermo Fisher #35553 antibodies are validated from the manufacturers' websites and citations therein; Tubulin (926-42211), IRDye-800 anti-rabbit (926-32211), IRDye-680RD anti-mouse (926-68072), and anti-FLAG M2 (A8592) antibodies are validated from the manufacturers' websites.

Eukaryotic cell lines

Policy information about cell lines

Cell line source(s)	HEK 293 and KPL-4 are from Genentech's in-house cell repository and were authenticated and mycoplasma tested before distribution.
Authentication	All cell lines are STR-validated in-house prior to distribution.
Mycoplasma contamination	Mycoplasma is tested regularly and all lines should be mycoplasma-free.
Commonly misidentified lines<br>(See ICLAC register)	No commonly misidentified lines were used in this study.
