# Supplementary figures and images for: Targeted degradation via direct 26S proteasome recruitment
Source: Nat Chem Biol. 2022 Dec 28;19(1):55–63. doi: 10.1038/s41589-022-01218-w (PMC9797404; doi:10.1038/s41589-022-01218-w)

Fig. 4e

Gel 1

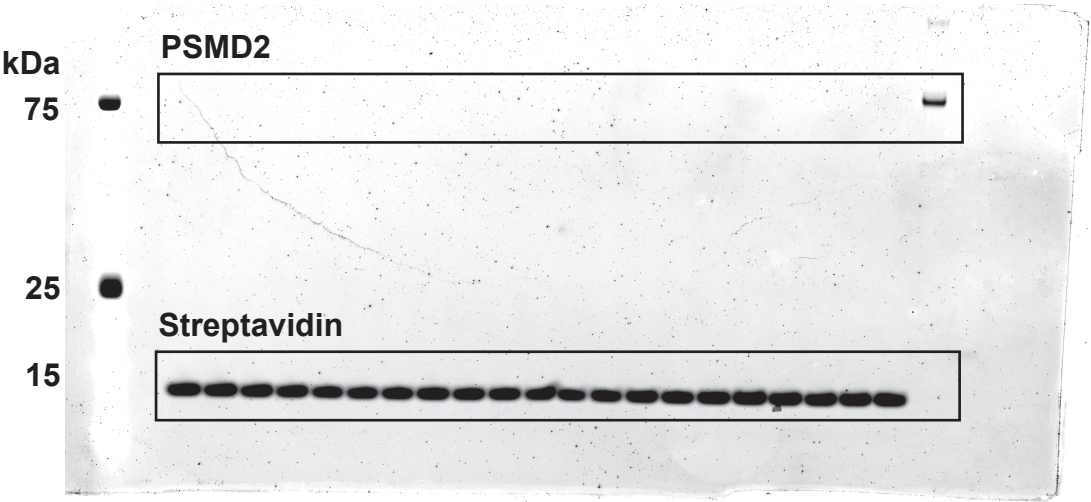

Gel 2

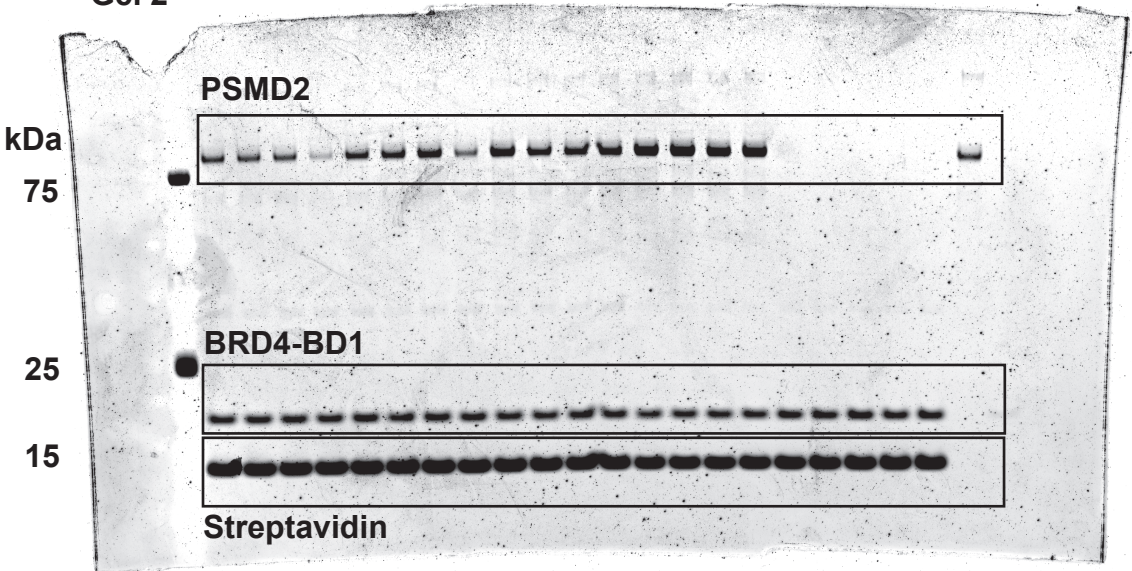

Supplement: Source Data Fig. 4 — Unprocessed western blots and/or gels. [file 41589_2022_1218_MOESM4_ESM.pdf]

Extended Dat Fig. 3, unprocessed gel

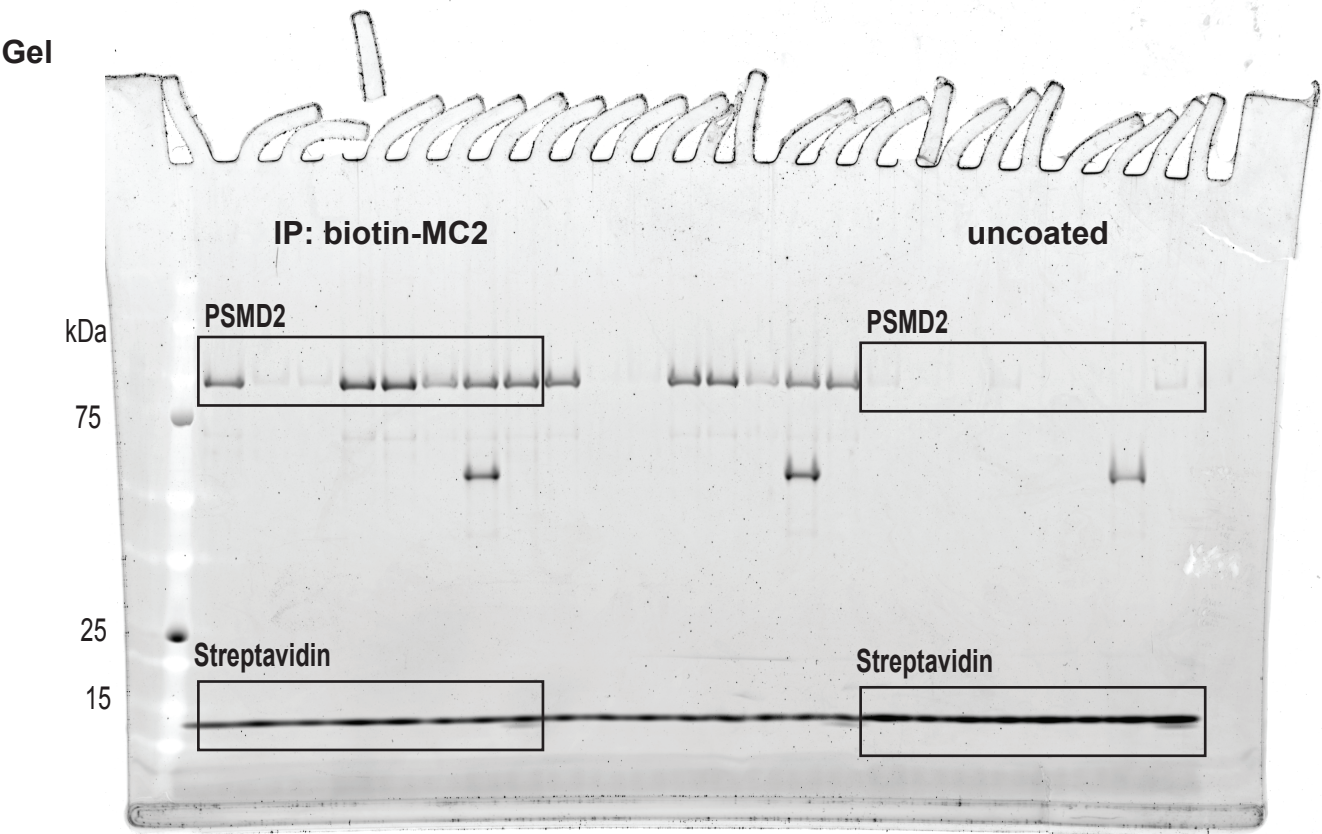

Supplement: Source Data Extended Data Fig. 3 — Unprocessed western blots and/or gels. [file 41589_2022_1218_MOESM7_ESM.pdf]

Extended Data Fig. 10a, unprocessed gel

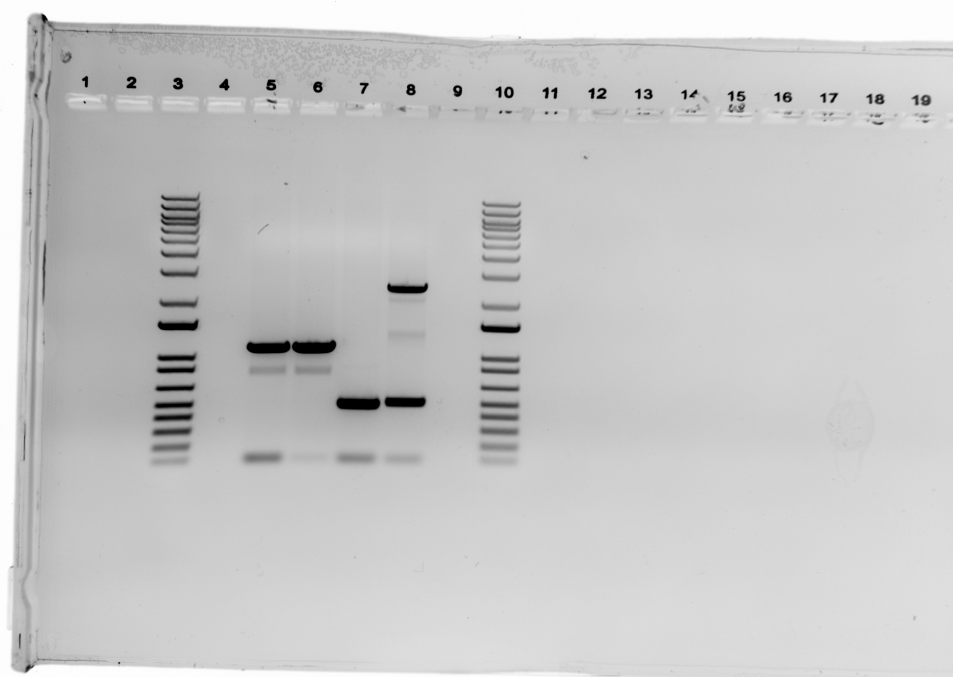

Supplement: Source Data Extended Data Fig. 10 — Unprocessed western blots and/or gels. [file 41589_2022_1218_MOESM8_ESM.pdf]
