# Supplementary material for: Targeted degradation via direct 26S proteasome recruitment
Source: Nat Chem Biol. 2022 Dec 28;19(1):55–63. doi: 10.1038/s41589-022-01218-w (PMC9797404; doi:10.1038/s41589-022-01218-w)

Fig. 6d

Blot 1

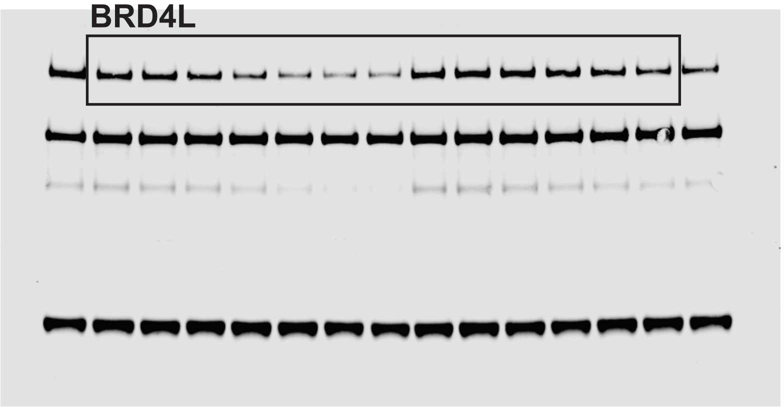

Blot 1 (contrast adusted-1)

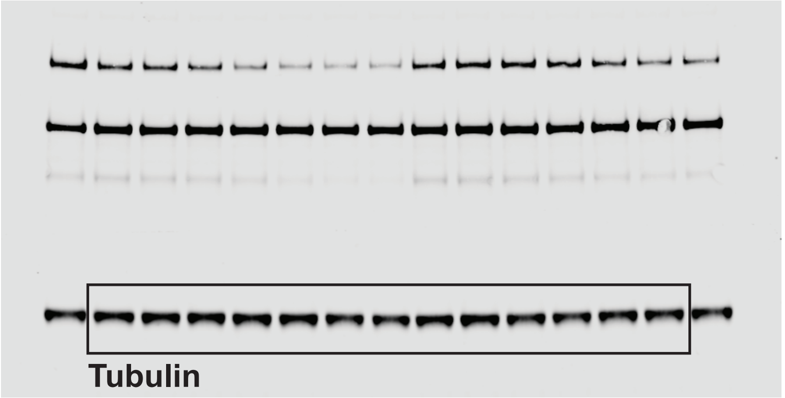

Blot 1 (contrast adusted-2)

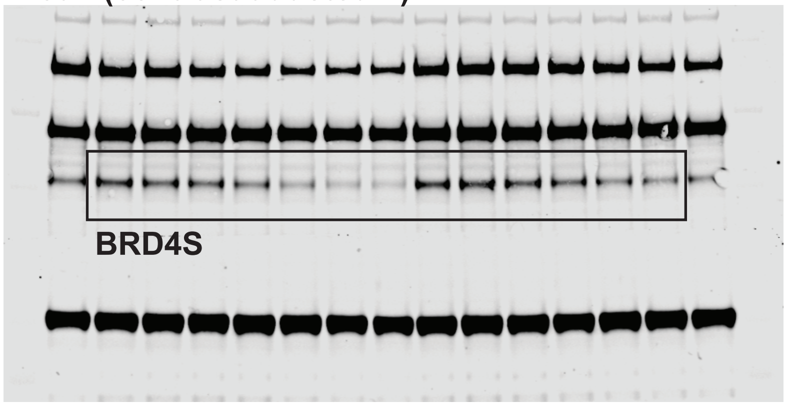

Blot 1 (with markers)

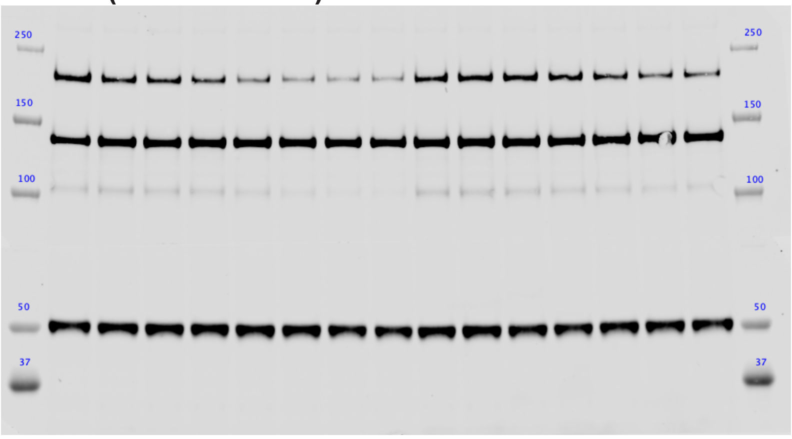

Blot 2

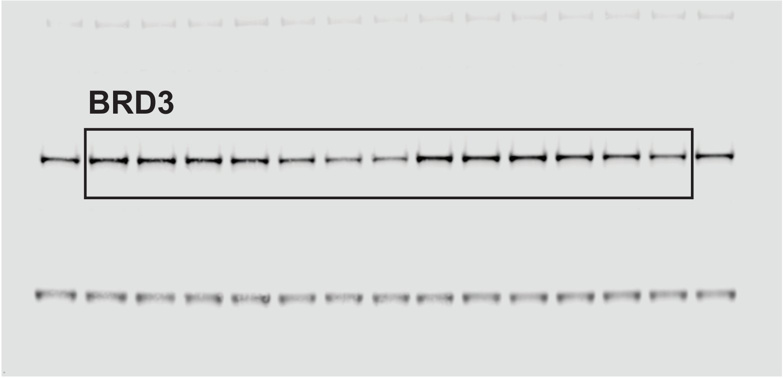

Blot 3

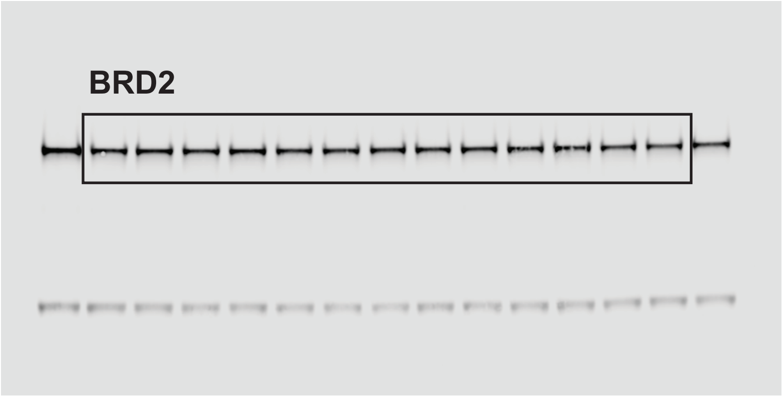

Blot 2 (with markers)

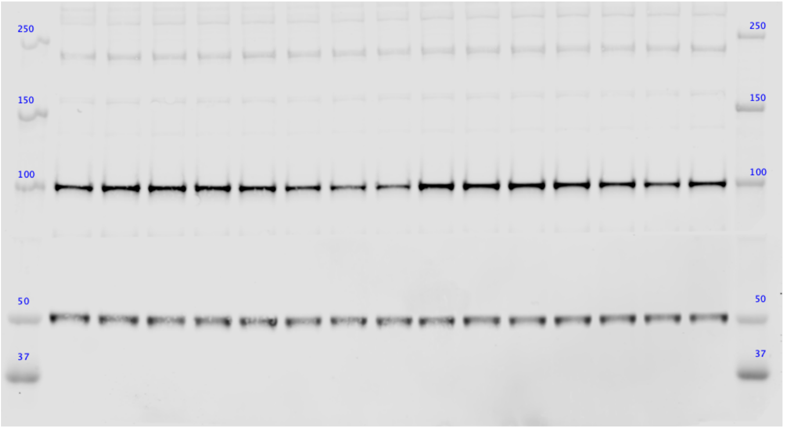

Blot 3 (with markers)

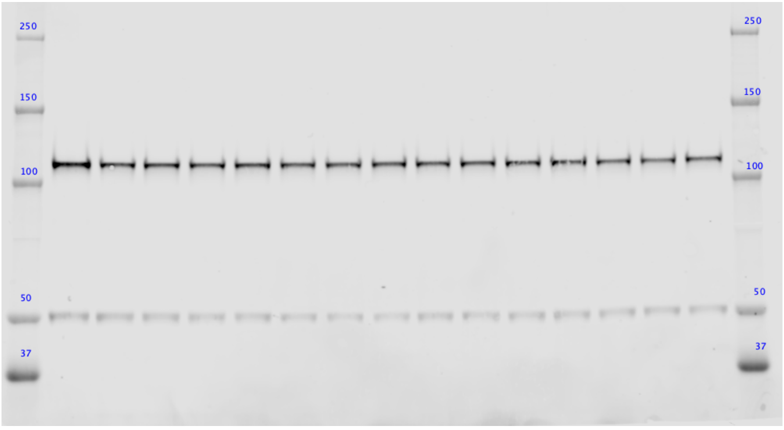

Fig. 6e

Blot 1

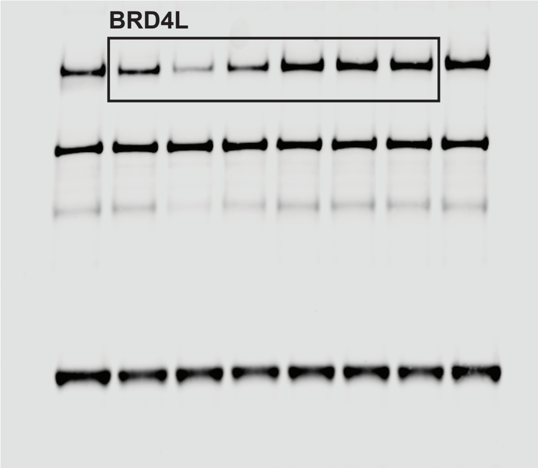

Blot 1 (contrast adusted-1)

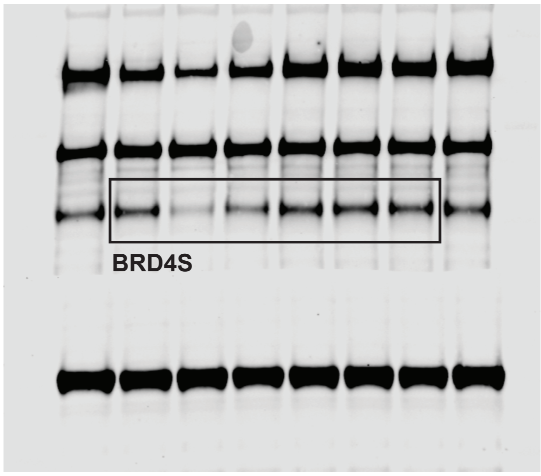

Blot 1 (contrast adusted-2)

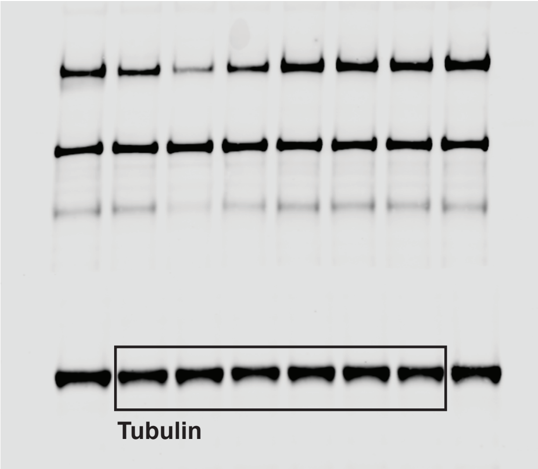

Blot 1 (with markers)

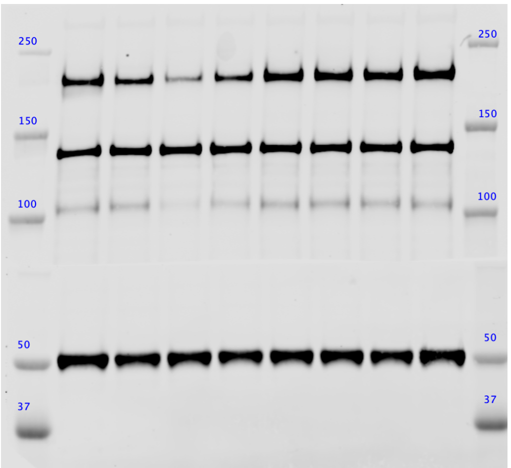

Blot 2

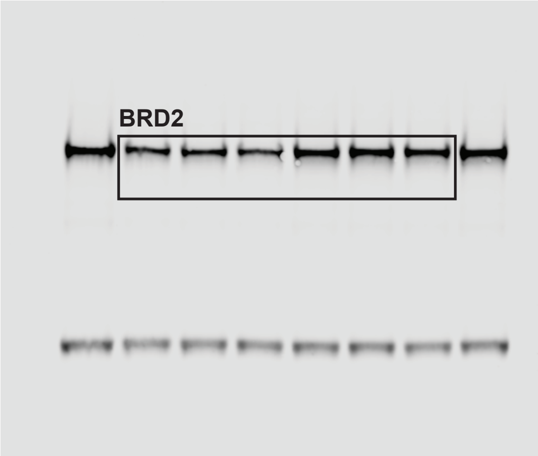

Blot 3

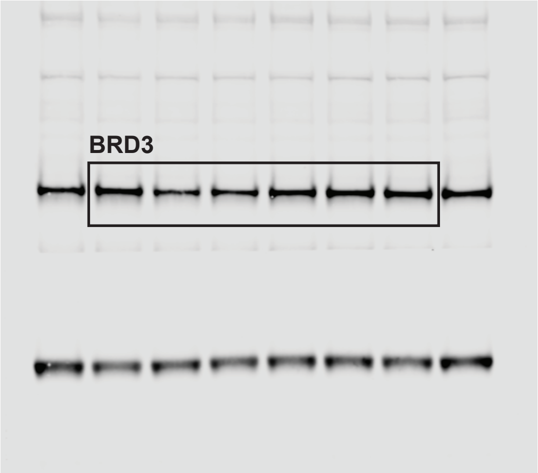

Blot 2 (with markers)

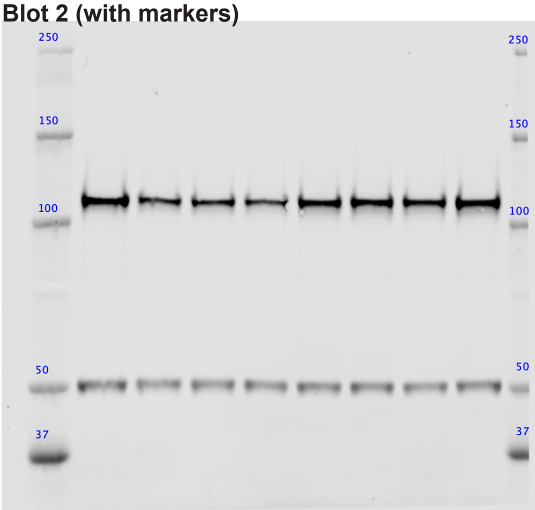

Blot 3 (with markers)

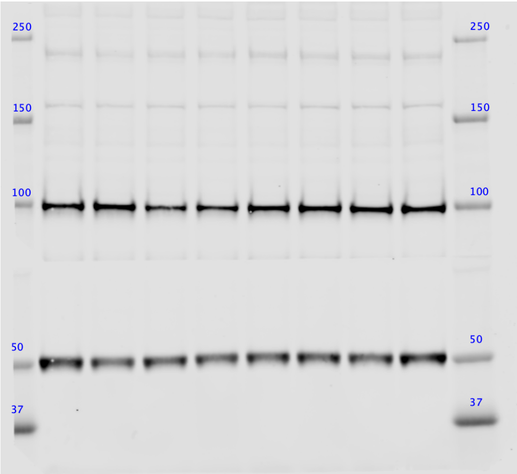

Fig. 6f

Blot 1

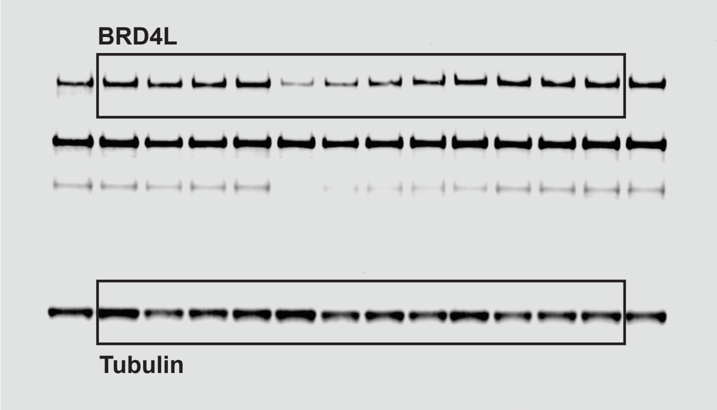

Blot 1 (with markers)

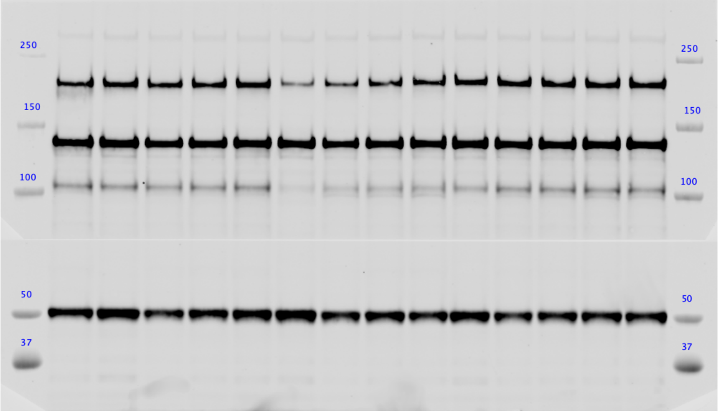

Blot 1 (contrast adusted-1)

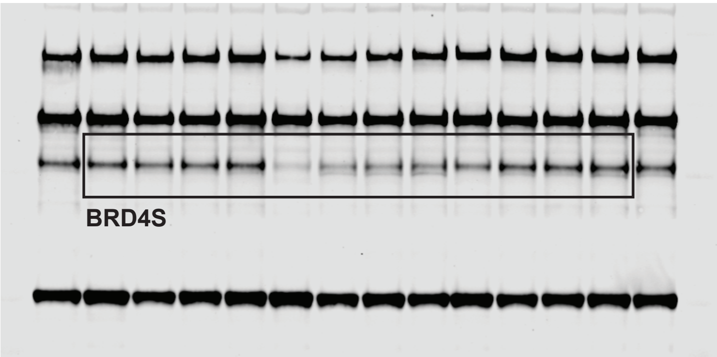

Blot 2

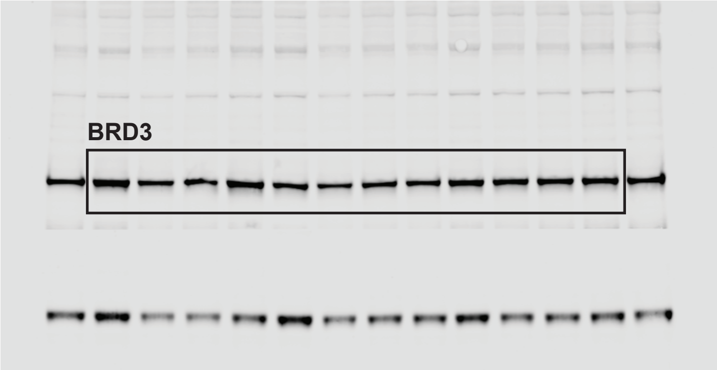

Blot 2 (with markers)

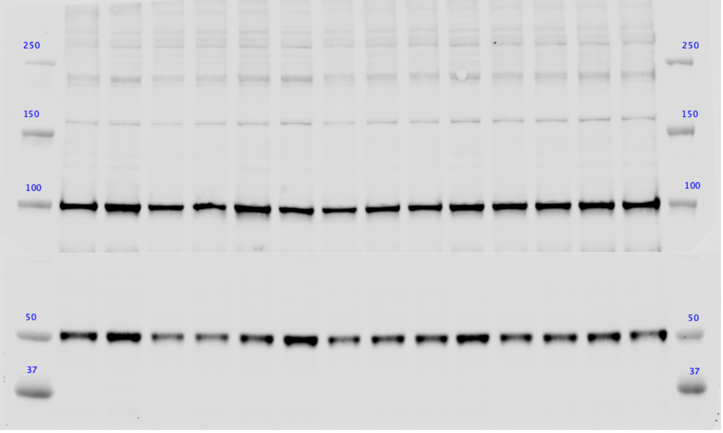

Blot 3

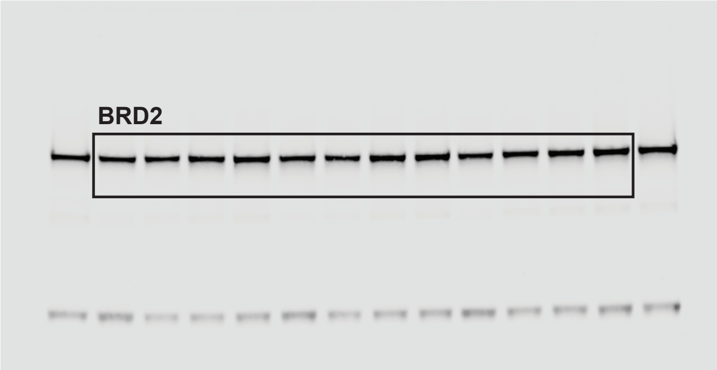

Blot 3 (with markers)

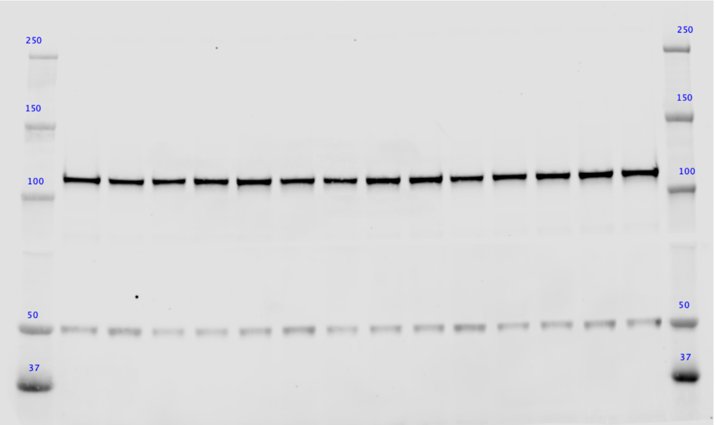

Fig 6g

Blot 1

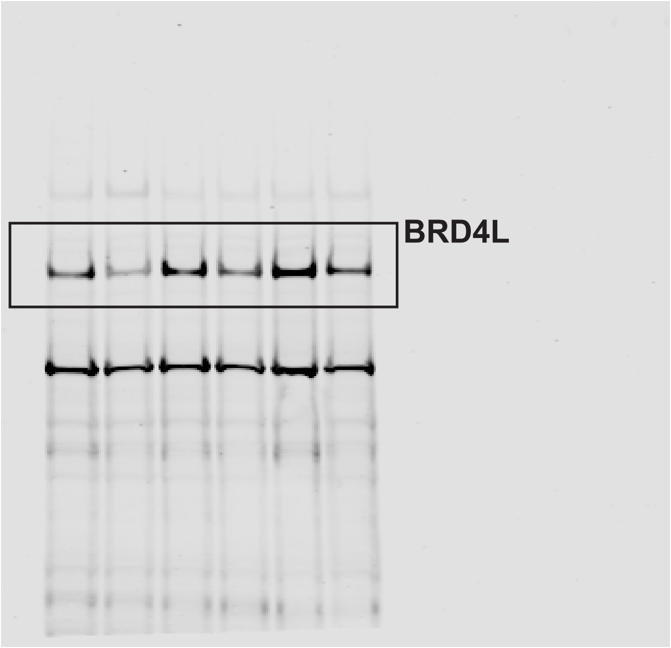

Blot 1 (with markers)

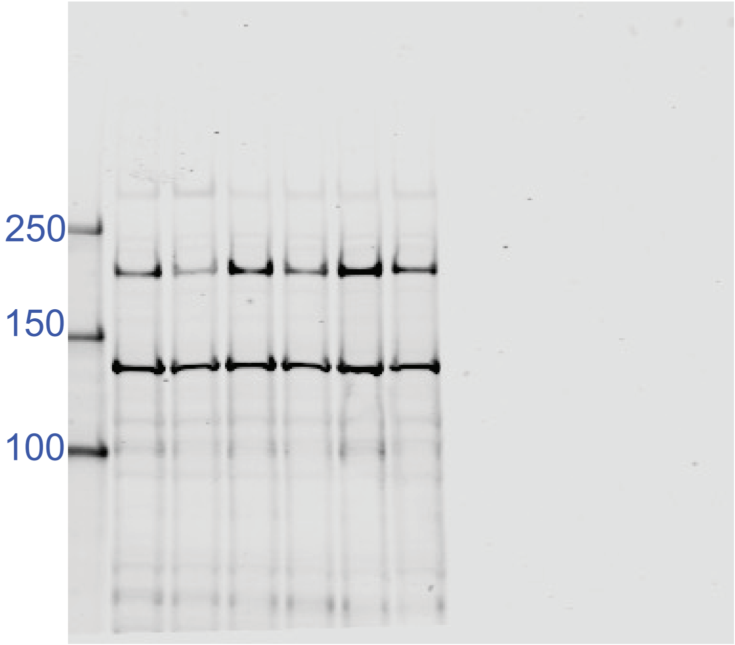

Blot 2

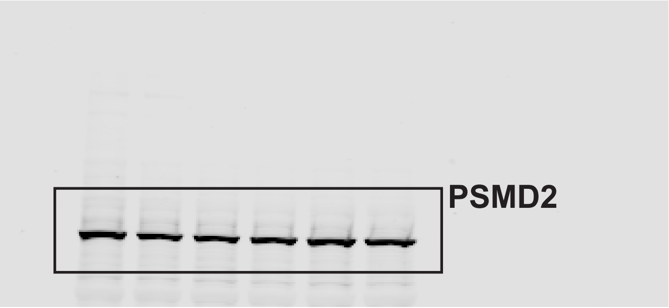

Blot 2 (with markers)

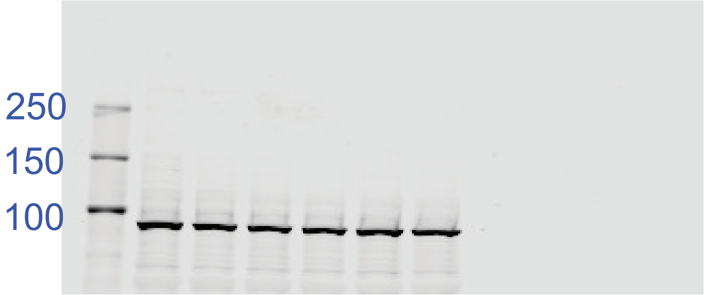

Blot 3

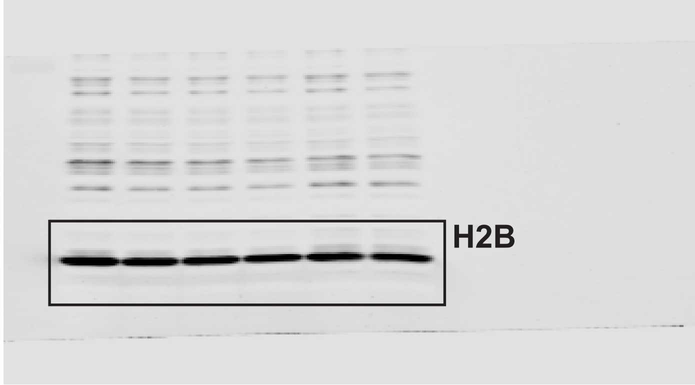

Blot 3 (with markers)

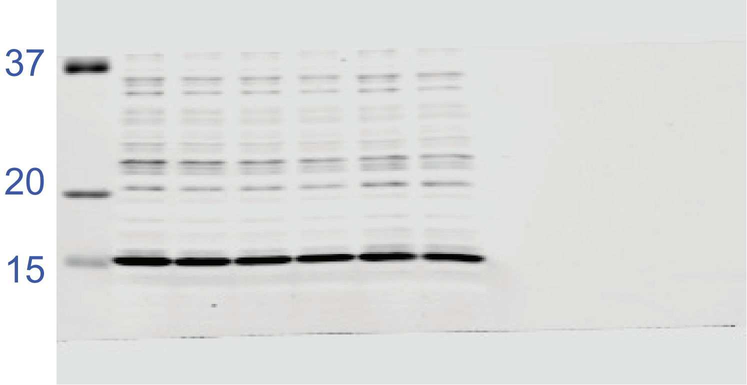

Supplement: Source Data Fig. 6 — Unprocessed western blots and/or gels. [file 41589_2022_1218_MOESM5_ESM.pdf]
