# Supplementary material for: Targeted degradation via direct 26S proteasome recruitment
Source: Nat Chem Biol. 2022 Dec 28;19(1):55–63. doi: 10.1038/s41589-022-01218-w (PMC9797404; doi:10.1038/s41589-022-01218-w)

Extended Data Fig. 2a, unprocessed gel

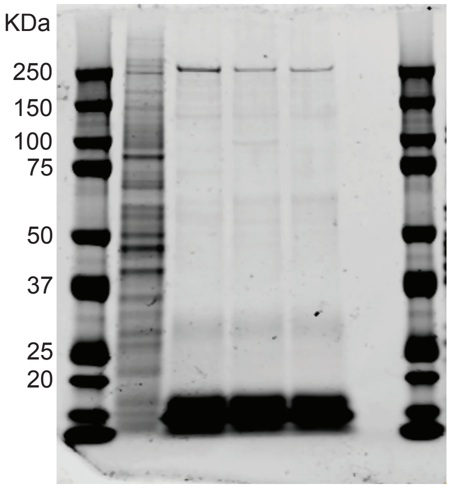

Extended Data Fig. 2b, unprocessed blots

Blot

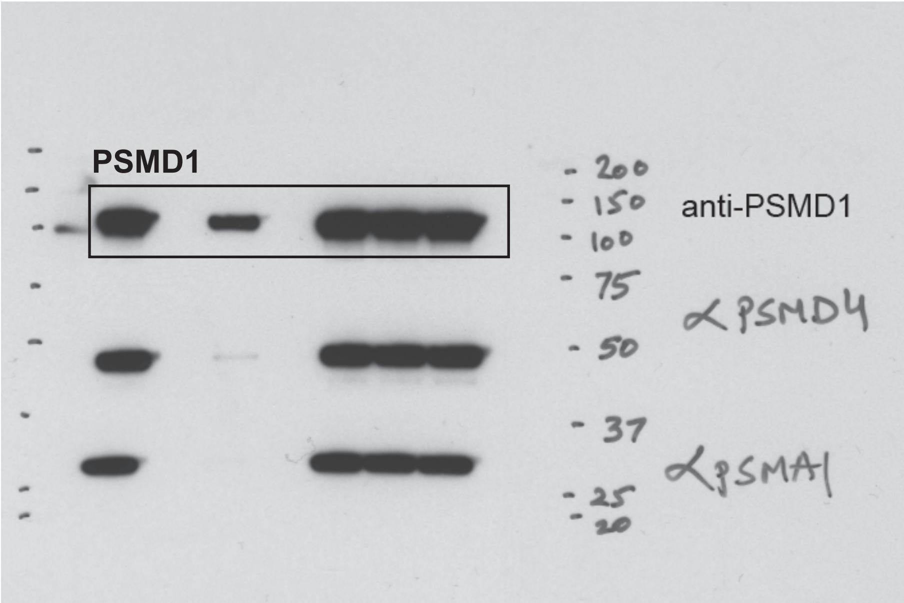

Blot
longer exposure

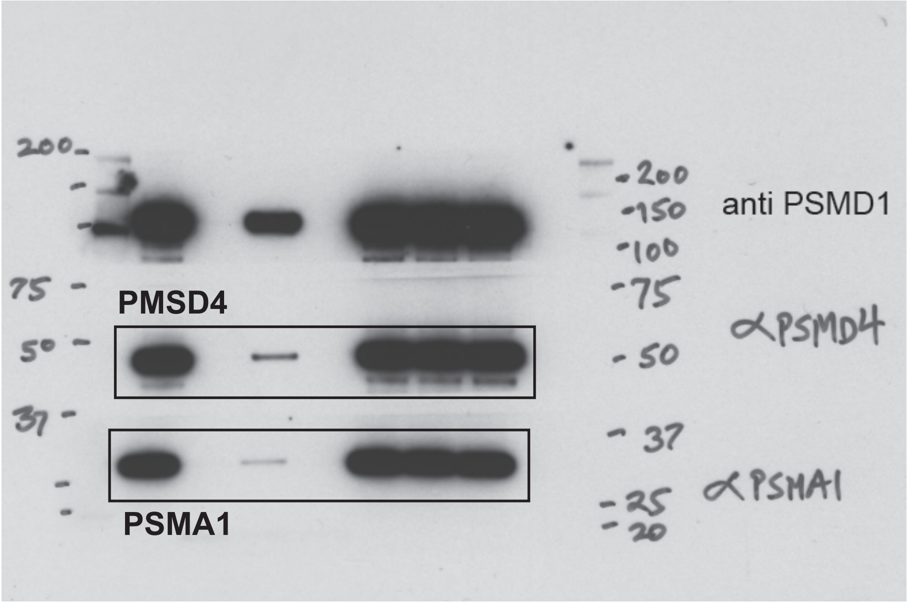

Supplement: Source Data Extended Data Fig. 2 — Unprocessed western blots and/or gels. [file 41589_2022_1218_MOESM6_ESM.pdf]
